# Implementation of a COVID-19 Genomic Surveillance Regional Network for Latin America and Caribbean region

**DOI:** 10.1371/journal.pone.0252526

**Published:** 2022-03-03

**Authors:** Juliana Almeida Leite, Andrea Vicari, Enrique Perez, Marilda Siqueira, Paola Resende, Fernando Couto Motta, Lucas Freitas, Jorge Fernandez, Barbara Parra, Andrés Castillo, Rodrigo Fasce, Alexander Augusto Martinez Caballero, COVID-19 Genomic Surveillance Regional Network, Lionel Gresh, Sylvain Aldighieri, Jean-Marc Gabastou, Leticia Franco, Jairo Mendez-Rico

**Affiliations:** 1 Health Emergencies Department, Pan American Health Organization, Washington, DC, United States of America; 2 Laboratorio de Virus Respiratorio, Fundação Oswaldo Cruz, Rio de Janeiro, RJ, Brazil; 3 Subdepartamento Genética Molecular, Instituto de Salud Pública de Chile, Santiago, Chile; 4 Subdepartamento Enfermidades Virales, Instituto de Salud Pública de Chile, Santiago, Chile; 5 Departamento de Investigación en Genómica y Proteómica, Instituto Conmemorativo Gorgas de Estudio de la Salud, Cuidad de Panamá, Panamá; Defense Threat Reduction Agency, UNITED STATES

## Abstract

The timely release of SARS-CoV-2 first genomic sequences allowed the identification of the etiologic agent and development of diagnostic protocols. Genomic sequencing was a crucial step in generating data for driving laboratory response and detections of SARS-CoV-2 since the start of the COVID-19 pandemic. Because of all the progression and achievements that timely release of genetic sequence data represents in the public health response, the Pan American Health Organization (PAHO) in collaboration with countries’ public health laboratories, started implementation of a network for strengthening the Latin America and Caribbean (LAC) region on timely generation of SARS-CoV-2 genomic data. Here we describe the implementation of the COVID-19 Genomic Surveillance Regional Network in the Americas region during the beginning of the pandemic. The establishment of this network has strengthened laboratory response capacity at the country level, as well as facilitated timely release of SARS-CoV-2 genomic information to be used to complement the multiple response strategies for COVID-19 pandemic mitigation. As genomic epidemiology is useful for guiding public health decisions on outbreak and response, we also analysed the first SARS-CoV-2 genomic sequence data from countries of the Latin America and Caribbean Region.

## Introduction

The first report of the coronavirus disease 2019 (COVID-19) cluster in Wuhan, China to the World Health Organization (WHO) was made in late December 2019, at that time it was known as a cluster of unusual severe pneumonia cases [1]. Due to rapid and collaborative work, in 10 days after notification, the first genome sequence was available at the Global Initiative on Sharing All Influenza Data (GISAID) [2, 3]. Timely public release of this novel coronavirus genomic data allowed the development of the first molecular diagnostic protocol released and published by the Charité –Universitätsmedizin Berlin Institute of Virology, Germany on the WHO webpage in less than 15 days after the cluster notification to WHO [4, 5].

The timely release of the first genomic sequences not only allowed the development of diagnostic protocols, but also the identification of the etiologic agent causing this outbreak. This novel coronavirus showed to be different from the severe acute respiratory syndrome coronavirus, and was eventually named severe acute respiratory syndrome coronavirus 2 (SARS-CoV-2) by the International Committee on Taxonomy of Viruses (ICTV) [6]. Genomic sequencing was a crucial step for generating data for driving laboratory response and laboratory case detections of SARS-CoV-2 since the first notifications of the novel coronavirus outbreak, now COVID-19 pandemic [7, 8].

Deeper genetic characterization of viruses has been the basis for developing diagnostic protocols, vaccines, and antiviral drugs. The use of this strategy has also been a useful tool for public health response regarding disease control. Among respiratory viruses, influenza virus is a classic example on how genetic characterization and phylogenetic analysis have been combined for influenza mitigation by providing information for vaccine compositions, molecular diagnostic protocols, antiviral development, monitoring of antiviral resistance and studies on viral lineage and molecular epidemiology [9–13].

Because of all progression and achievements that the timely release of genetic sequence information in open database represents for public health response to this viral outbreak, the Pan American Health Organization (PAHO), which is the WHO Regional Office for the Americas, in collaboration with countries’ public health laboratories, started the implementation of a regional network for SARS-CoV-2 genomic sequencing. This initiative was based on strengthening the Latin America and Caribbean (LAC) countries in the region for generating and timely making available SARS-CoV-2 genomic sequence data in GISAID.

Likewise, considering the rapid evolution of this virus in different subclades and the emergence of variants that are currently distributed around the globe, the need of generating SARS-CoV-2 genomic data should be a priority. Here we describe the implementation of the COVID-19 Genomic Surveillance Regional Network in the Region of the Americas. This network was created not only to enhance the laboratory response capacity at country level, but also to generate timely information of SARS-CoV-2 genomic sequencing data to be used as part of multiple response strategies for mitigation of the COVID-19 pandemic.

## Materials and methods

### Implementation of COVID-19 Genomic Surveillance Regional Network in the Americas

**Fig 1:**
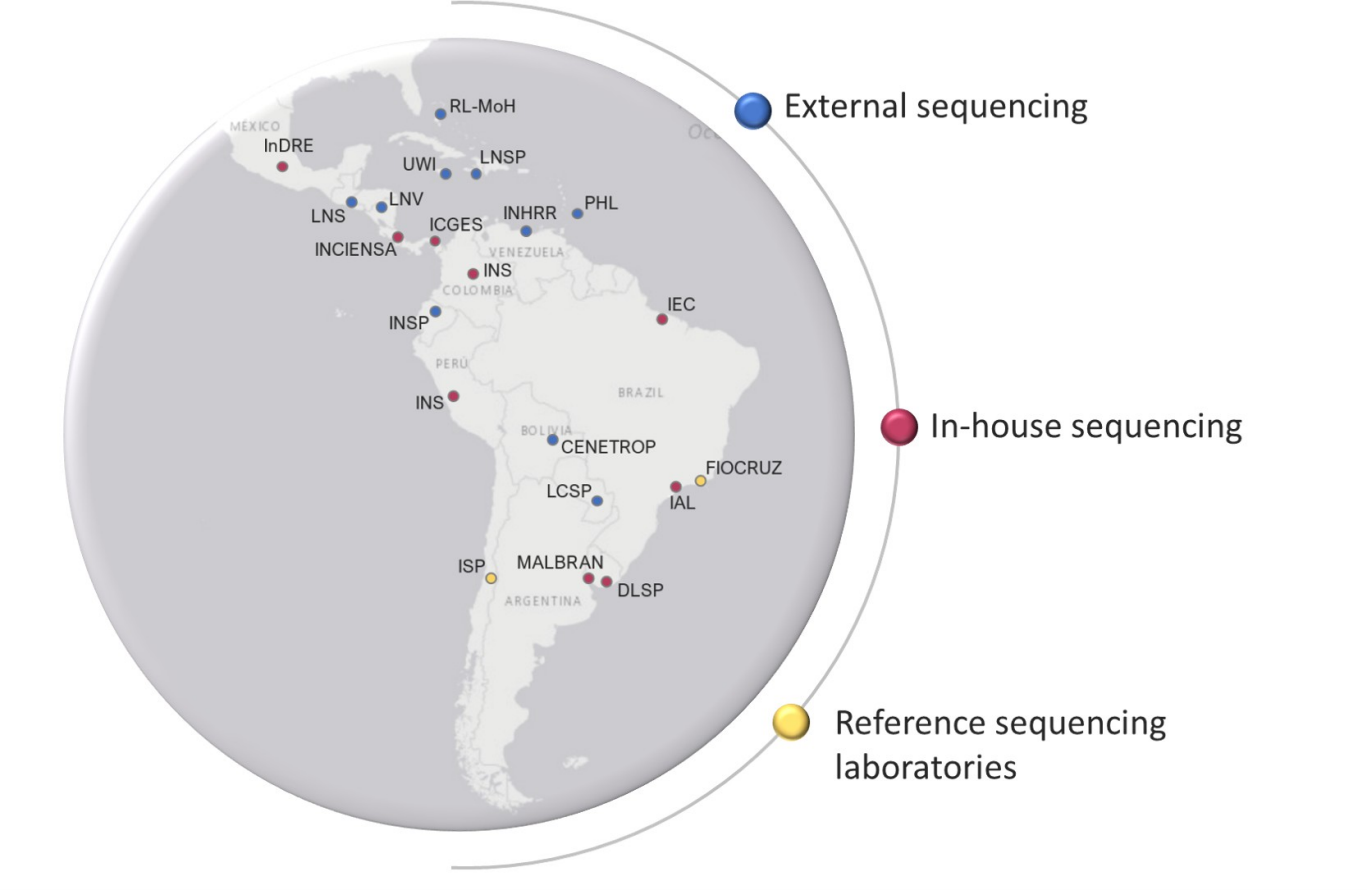
Locations of current laboratories part of the COVID-19 Genomic Surveillance Regional Network in the Americas region. CENETROP—Centro Nacional de Enfermedades Tropicales (Bolivia); DLSP–Departamento Laboratorio de Salud Pública (Uruguay); FIOCRUZ–Fundação Oswaldo Cruz (Brazil); IAL–Instituto Adolfo Lutz (Brazil); ICGES -Instituto Conmemorativo Gorgas de Estudios de la Salud (Panama); IEC–Instituto Evandro Chagas (Brazil); INCIENSA—Instituto Costarricense de Investigación y Enseñanza en Nutrición y Salud (Costa Rica); InDRE—Instituto de Diagnóstico y Referencia Epidemiológicos (Mexico); INHRR—Instituto Nacional de Higiene Rafael Rangel (Venezuela); INS–Instituto Nacional de Salud (Colombia); INS–Instituto Nacional de Salud (Peru); INSPI–Instituto Nacional de Investigación en Salud Pública (Ecuador); ISPCH–Instituto de Salud Pública (Chile); LCSP–Laboratorio Central de Salud Pública (Paraguay); LNS–Laboratorio Nacional de Salud (Guatemala); LNSP–Laboratoire National de Santé Publique (Haiti); LNV—Laboratorio Nacional de Virología (Honduras); MALBRAN—Administración Nacional de Laboratorios e Institutos de Salud “Dr. Carlos G. Malbrán” (Argentina); PHL–Best dos Santos Public Health Laboratory (Barbados); RL-MoH–Reference Laboratory of the Ministry of Health (Bahamas); UWI–University of West Indies (Jamaica). Red–In house sequencing; Blue–External sequencing; Yellow–Reference Sequencing Laboratories.

PAHO has been providing technical cooperation to countries in the Region of the Americas to strengthen their laboratory capacity to respond to the COVID-19 pandemic since the declaration of the outbreak. As part of the surveillance strengthening strategy, PAHO has been working on implementing the COVID-19 Genomic Surveillance Regional Network in the Americas, starting with 19 countries invited to participate based on most recent PAHO epidemiological data by the time of the network implementation [14]. For better representativeness of all subregions of the Americas the following countries were considered: North America: Mexico; Caribbean region: Bahamas, Barbados, Haiti and Jamaica; Central America: Costa Rica, Guatemala, Honduras and Panama; Andean region: Colombia, Ecuador, Peru and Venezuela (Boliviarian Republic of); Southern Cone: Argentina, Bolivia (Plurinational State of), Brazil, Chile, Paraguay and Uruguay (Fig 1).

In order to generate high-quality SARS-CoV-2 genomic sequence data in a faster and timely manner, next generation sequencing (NGS) was used for full genome sequencing. Some of the laboratories invited to participate in the network have NGS capacity already implemented and most of them rely on the Illumina platform; therefore, this platform was preferred for standardizing the protocols to be used among countries of the network. These in-house sequencing countries have also developed reliable protocols. The platform for NGS through in-house sequencing laboratories is heterogenic including Nanopore and Illumina technologies. NGS protocols from the reference sequencing laboratories had been previously developed independently by each laboratory, focused on short-read sequences. These protocols were made available to the network, as was provision of virtual training on SARS-CoV-2 NGS and provision of primers for implementation by the Instituto de Salud Publica de Chile (ISPCH, Chile) and Fundação Oswaldo Cruz (FIOCRUZ, Brazil) [15, 16]. The protocol developed by FIOCRUZ is suitable for different sequencing approaches from short-reads sequencing, as Illumina platform, to single-reads sequencing, such as the Nanopore platform, covering the diverse sequencing methods inside the laboratories of the network.

Two laboratories participate in the network as reference sequencing laboratories for the countries that do not have NGS capacity in place: Laboratório de Vírus Respiratório e Sarampo / Instituto Oswaldo Cruz / FIOCRUZ–Rio de Janeiro–Brazil; and Sub departamento de Genética Molecular / ISPCH–Santiago–Chile (Fig 1). Laboratories performing NGS were instructed to sequence a first-round batch and to upload the information to the open-access database GISAID for review of sequence quality and for making sequence data publicly available. Country laboratories where sequencing is not available, were instructed to send specimens of SARS-CoV-2 to one of the reference sequencing laboratories.The shipping of the samples for external sequencing in the network is supported by PAHO, as are NGS reagents for the reference sequencing laboratories.

For first-round sequencing, countries were instructed to sequence or send to one of the sequencing laboratories, at least 10 specimens of SARS-CoV-2. Sample selection was to be representative based on severity (mild, severe or fatal cases), age group, location, and period of time.

Through the network, technical support is also provided by ISPCH, FIOCRUZ and PAHO to countries performing their own in-house sequencing. All sequences are to be made available in a timely manner, uploading them to the open-access database GISAID. A metadata file is sent to PAHO for gathering regional genetic and epidemiologic information on sequenced samples, including history of travel, medical intervention and comorbidity, among others.

### SARS-CoV-2 genotype and maximum likelihood phylogenetic inference

SARS-CoV-2 complete genome sequences of the LAC region generated by the participating laboratories were retrieved from GISAID as of 19 January 2021 together with the correspondent metadata. The accession numbers were recorded (S1 Table). Sequences available in GISAD from non-participating countries in the network for the study period were not included in the alignment datasets. The pangolin nomenclature proposed by Rambaut et al. [17] was used for the genotype analysis. For phylogenetic analyses, low-quality genomes (> 5% of ambiguous positions), incomplete genome sequences, or sequences with stretches of NNNs or gaps were excluded. A final total of 5132 sequences were used for the dataset. Blastn from BLAST v. 2.5.0 was used to select the closest genomes not belonging to LAC for a broader view of the dynamics of SARS-CoV-2 evolution. A total of 1,000 of the outlier genomes were randomly sampled and included in the final dataset. The dataset generated was aligned with using MAFFT v7.467 [18] and subjected to maximum-likelihood (ML) phylogenetic analyses. The ML phylogenetic tree was inferred using IQTREE v2.0.4 [19], under the GTR+F+I+G4 nucleotide substitution model as selected by the ModelFinder application [20] and the branch support was assessed by the approximate likelihood-ratio test based on a Shimodaira–Hasegawa-like procedure (SH-aLRT) with 1,000 replicates [21]. The phylogenetic tree visualization was built using R v. 4.0.2 [22] and the following packages: tidyverse v. 1.3 [23], tidytree v. 0.3.3 [24], ggtree v. 2.2.4 [25] and RColorBrewer v. 1.1–2 [26].

## Results and discussion

### Laboratory sequencing capacity for SARS-COV-2 in Latin America and Caribbean region

Laboratories constitute one of the core pillars for response to a pandemic. In addition to the major role of laboratory response in the diagnosis, notification, and monitoring of cases, it can also generate evidence for public health actions to mitigate a pandemic. Each laboratory plays an important role in helping to improve the genomic sequence data available for supporting public health response, either by internally sequencing or by using external sequencing. A strategic response is essential for a timely laboratory preparedness and response [27]. The implementation of a genomic surveillance network throughout the Americas was key for establishing an collaborative networking among PAHO, national authorities and national public health laboratories for timely SARS-CoV-2 genomic surveillance, including efficient logistics and procurement and distribution of sequencing reagents, for in-country and sub-regional trainings in genomic sequencing and bioinformatics, and also for guidance on official notifications through international health regulations.

As shown in Table 1, most of the sequences uploaded to GISAID are from participating laboratories in this network, for a total of 842 out of 1578 sequences available. For some countries that rely on external sequencing for generating genomic sequence data (GSD), almost 100% of the data are being generated through the network (Table 1). The number of sequences available for these countries is set to increase as shipments of samples continue to arrive at the reference sequencing laboratories. Moreover, data sharing by all laboratories participating in the network becomes even more relevant as they are the national public health laboratories for respiratory virus surveillance, being capable of also sharing public health information and data for further deeper analysis.

**Table 1:** Laboratories participating in the COVID-19 Genomic Surveillance Regional Network in the Americas, July 2020.

AMERICAS SUBREGION	COUNTRY	INSTITUTION	NGS PLATFORM	EXTERNAL SEQUENCING	Total GSD^a^	Laboratory GSD^b^	Percent of GSD^c^
**North America**	Mexico	Instituto de Diagnóstico y Referencia Epidemiológicos	Y	N	599	122	20.37
**Caribbean**	Bahamas	Reference Laboratory of the Bahamas Ministry of Health	N	Y	*in process* ^*d*^	N	N
	Barbados	Best-dos Santos Public Health Laboratory	N	Y	N	N	N
	Haiti	Laboratoire National de Santé Public	N	Y	*in process* ^*d*^	N	N
	Jamaica	University of the West Indies	N	Y	13	13	100
**Central America**	Costa Rica	Instituto Costarricense de Investigación y Enseñanza en Nutrición y Salud	Y	N	181	163	92.09
	Guatemala	Laboratorio Nacional de Salud	Y	Y	32	30	93.75
	Honduras	Laboratorio Nacional de Virologia	N	Y	N	N	N
	Panama	Instituto Conmemorativo Gorgas de Estudios de la Salud	Y	N	314	314	100
**Andean Region**	Colombia	Instituto Nacional de Salud	Y	N	288	286	99.3
	Ecuador	Instituto Nacional de Investigación en Salud Pública	N	Y	196	39	19.9
	Peru	Instituto Nacional de Salud	Y	N	441	415	94.1
	Venezuela	Instituto Nacional de Higiene Rafael Rangel	N	Y	12	0	0
**Southern Cone**	Argentina	Instituto Nacional de Enfermedades Infecciosas, ANLIS C.G. Malbrán	Y	N	483	4	0.83
	Bolivia	Centro Nacional de Enfermedades Tropicales	N	Y	*2*7	27	100
	Brazil	Fundação Oswaldo Cruz	Y	N	2165	462	21.34
		Instituto Adolfo Lutz	Y	N	2165	397	18.34
		Instituto Evandro Chagas	Y	N	2165	33	1.52
	Chile	Instituto de Salud Pública de Chile	Y	N	963	492	51.09
	Colombia	Instituto Nacional de Salud	Y	N	288	286	99.3
	Paraguay	Laboratorio Central de Salud Pública	N	Y	*in process* ^*d*^	N	N
	Uruguay	Departamento de Laboratorio de Salud Publica	Y	N	129	1	0.78
**REGION OF THE AMERICAS**				**TOTAL**	**5843**	**3083**	**52.76**

GSD, Genomic Sequence Data; NGS, Next generation sequencing

^a^–GSD available in GISAID from the country

^b^–GSD available in GISAID from the participating laboratory

^c^–Percentage of GSD available from the participating laboratory in relation to total of GSD available for the country in GISIAD

^d^–in process: samples are being shipped or under sequencing.

Building an international collaboration network was important for advancing with key components to enhance the capacity for genomic sequencing in the Region of the Americas as a whole, through linking diverse types of expertise, capacitation for higher sequence quality and fostering a collaborative spirit to generate data to support COVID-19 mitigation actions. A major initial challenge, that persists due to flight restrictions, is sample transport from the originator laboratories to the reference sequencing laboratories. Different couriers have been used to bridge this gap. Considering ethical implications, through official agreement, all samples remain with the originator country as owner of the intellectual property of the material and any data generated. It is important to notice that these samples are collected under Public Health Surveillance protocols and systems, and are intended to detect only the pathogens under investigation and not to identify any human marker. The external sequencing metadata generated are only shared on GISIAD after authorization from the country concerned. In some particular cases, countries may request a material transfer agreement where additional conditions and limitations might be established. PAHO has been working closely with the countries to provide all support and articulation among the network to expedite sequencing, including the provision of reagents to the reference sequencing laboratories, funding of shipping, and any other additional support necessary for the sustainability of the network.

### SARS-CoV-2 genotypes circulating in the Latin America and Caribbean region

Since the initial genomic characterization of the COVID-19 virus, the virus has diverged in different subclades [3]. Although mutation is naturally expected in the virus evolution process and some specific mutations define the viral subclades circulating, if these mutations result in an altered virus tropism, infectivity or antigenicity it could lead to implications for vaccine and antiviral development, as well as impact measures to control the pandemic.

**Fig 2:**
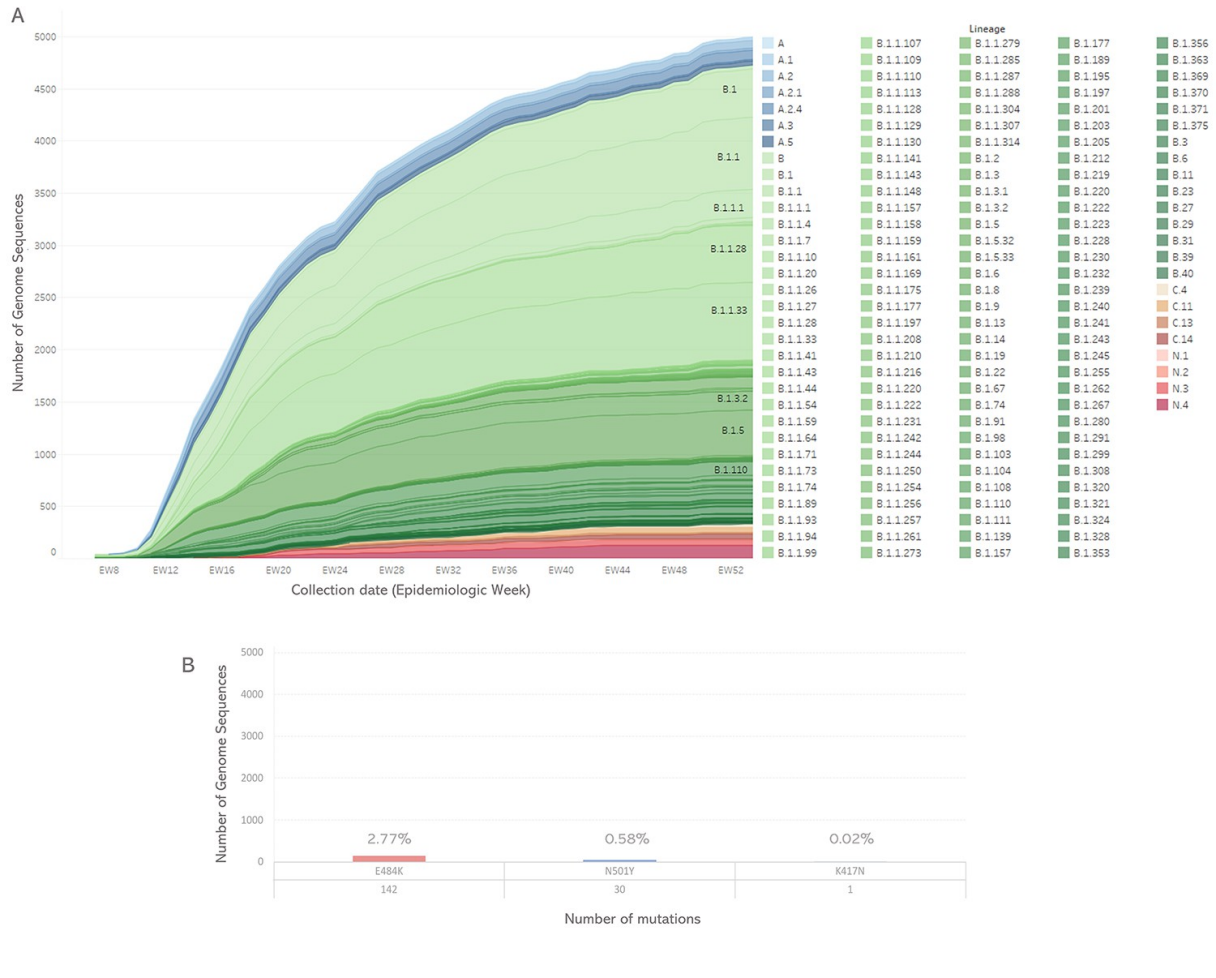
(A) Genetic groups and (B) E484K, N501Y and K417N spike mutations identified among SARS-CoV-2 circulating in Latin America and the Caribbean regions, epidemiologic weeks 1/2020-3/2021.

Due to this high priority for characterizing the SARS-CoV-2 virus, the genotypes of the SARS-CoV-2 virus circulating in the LAC Region were assessed. A heterogenicity of genetic group circulation was observed among the LAC Region sequences available (Fig 2A). Up to January 2021, most of the sequences generated for circulating SARS-CoV-2 in the region belonged to the B.1 clade, more specifically to the subclade B.1.1.33, indicating that this genetic group was the most prevalent circulating the LAC Region. Multiple subclades were identified across the inferred SARS-CoV-2 phylogeny with genomic sequences scattered across the network reflecting genetic diversity of SARS-CoV-2 circulating in the countries of the region (Fig 2A). The mutation D614G on the Spike (S) protein of the virus is one of the substitutions that defines this clade B.1, and it has been assessed for increased infectivity or virulence as this protein plays a key role in the host cell receptor recognition [28–30]. Among all the analyzed genomic sequences generated for the LAC countries in 2020, most of them (93%) shared the D614G mutation. At this moment, there is no sufficient evidence to strongly support that some circulating SARS-CoV-2 viruses have in fact increased virulence [30]. However, other mutations, such as E484K, N501Y, and K417N, have also been detected on the S protein, being found in emerging SARS-CoV-2 variants in the LAC region [31]. Although not being linked to augmented virulence, it has been suggested to increase viral transmissibility due to conformational changes induced by these mutations on the receptor binding domain of the S protein [32, 33]. So far, among the LAC sequences analyzed, 3% shared the E484K mutation and 0.5% had the N501Y mutation (Fig 2B), while the K417N spike mutation was detected in only one sequence.

Antigenic drift is seen among the common cold coronaviruses OC43 and 229E and in SARS-CoV-1 [28]. Therefore, at this point, concurrence of genetic surveillance, antigenic and neutralization assessment become important for understanding the implication of the diverse emerged genetic groups and the implications for immune recognition, antigenicity and adaptation to human host. The number of sequences available for LAC have raised exponentially since the first sequence release in GISAID, due to laboratories efforts to generate SARS-CoV-2 genomic sequence data in a timely manner (Fig 2). This information is relevant also for detecting early-signals of virus evolution, such as genetic diversity, for assessment the mutations for phenotypic implications such as antigenic characteristics, virulence and host adaptation.

### Genomic sequence alignment and phylogenetic analyses

Genome sequence analysis requires the removal of low-quality sequences from the datasets that may contain deletions, or insertions or ambiguous bases because of the potential to downstream inaccurate genetic characterization and phylogenetic analysis.

Sequences obtained using the protocols shared though the network from the reference sequencing laboratories showed high quality of sequences, with little need for exclusion. Most of the 880 sequences excluded from the analysis due to low-quality were generated using protocols from laboratories outside of the network. In the light the COVID-19 Genomic Surveillance Regional Network for the LAC region, this finding shows a strengthening of the network, with potential for generating high-quality SARS-CoV-2 genomic sequences.

**Fig 3:**
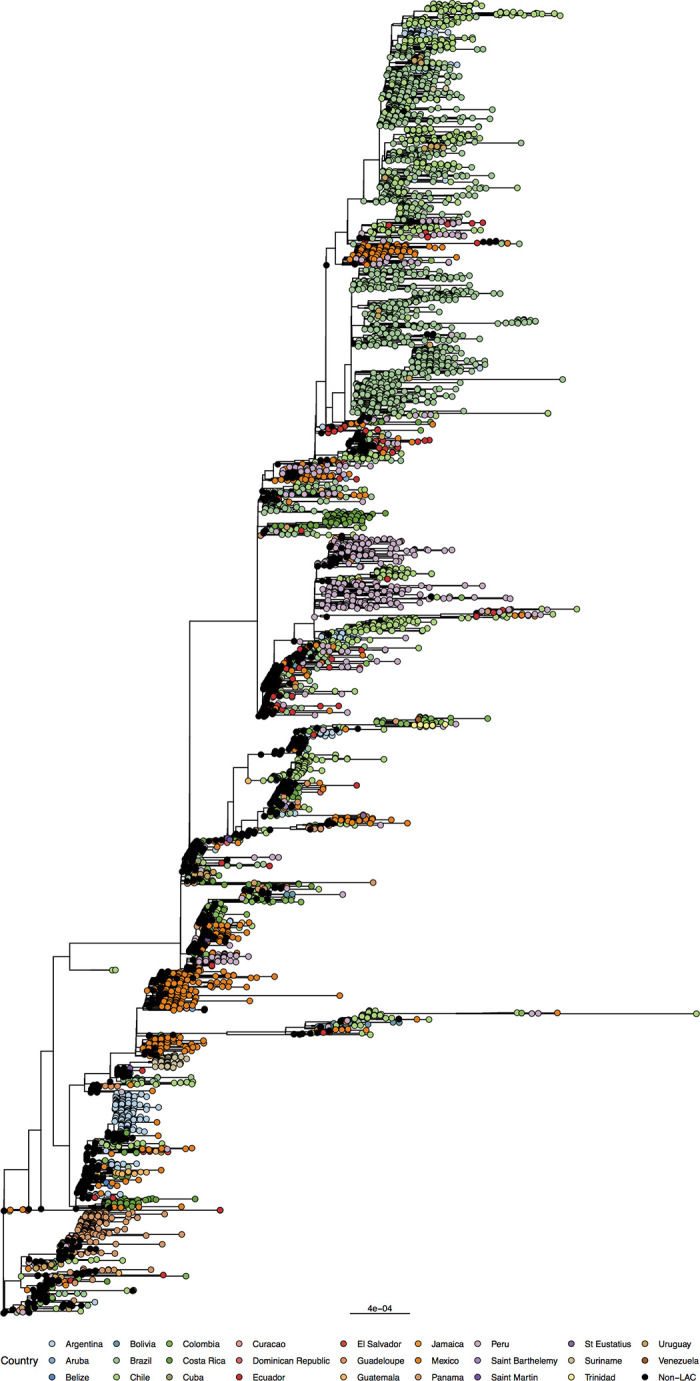
Maximum-likelihood phylogeny of the SARS-COV-2 circulating in Latin America and the Caribbean regions. Countries sequences marked by colored circles.

These sequences were used for phylogenetic reconstruction. The phylogenetic inference showed genetic group circulation heterogenicity inside the countries with no specific pattern among the LAC region sequences available (Fig 3). However, multiple introduction events in the LAC countries can be identified. These events could have been responsible for accelerating the dissemination of SARS-CoV-2 throughout the region in the initial outbreak stages. To better understand the SARS-CoV-2 virus circulation in the Region of the Americas and for reconstructing viral circulation patterns across time, a uniform number of sequences per country is needed. An increase in the generation of sequences with more geographical representativeness for the region is expected though the collaboration and networking of the laboratories of the COVID-19 Genomic Surveillance Regional Network, especially for the countries that rely on external sequencing.

## Conclusions

The COVID-19 Genomic Surveillance Regional Network is the first large-scale genomic sequencing network implemented in the LAC Region to generate data as part of the actions and public health response to a pandemic. This network formalizes the work already being carried out by participating laboratories performing in house sequencing of SARS-CoV-2, providing a space for discussion and information sharing on genomic sequencing. The detection of multiple sub-clades, including the presence of mutations that could be related to increased infectivity or virulence underscores the importance of strengthening genomic sequencing throughout the region and increasing the number of genomic sequences in order to closely monitor and survey emerging genetic variations. Additionally, this network provides the opportunity to sequence circulating strains of SARS-CoV-2 for countries with no NGS installed capacity. PAHO is working with regional partners to ensure the sustainability of this network. All the efforts from the participating laboratories to timely sequence and make available the genomic data have resulted in a higher quality and quantity of sequences from countries in the LAC Region, being a key element for the genomic surveillance of SARS-CoV-2. These data are critical not only for developing and improve viral diagnostic protocols, vaccines and antiviral drugs, but also for molecular epidemiologic studies, contact tracing, and enhanced surveillance. Working together as a network considerably increase the contribution from Latin America and the Caribbean Region to the SARS-CoV-2 genomic sequences data for global sharing. This genomic surveillance network also lay the foundation for genomic analysis to be used as a tool for public health response to future viral pandemics at regional level in the Region of the Americas.

## Supporting information

S1 Table Sequences from participating laboratories available in GISAID included in the study. (PDF)
