# Supplementary material for: Implementation of a COVID-19 Genomic Surveillance Regional Network for Latin America and Caribbean region
Source: PLoS One. 2022 Mar 3;17(3):e0252526. doi: 10.1371/journal.pone.0252526 (PMC8893691; doi:10.1371/journal.pone.0252526)
Supplement: S1 Table — (PDF) [file pone.0252526.s001.pdf]

We gratefully acknowledge the following Authors from the Originating laboratories responsible for obtaining the specimens, as well as the Submitting laboratories where the genome data were generated and shared via GISAID, on which this research is based.

All Submitters of data may be contacted directly via www.gisaid.org

Authors are sorted alphabetically.

Accession ID	Originating Laboratory	Submitting Laboratory	Authors
EPI_ISL_699655	1-Laboratory of Microbiology, National Reference Lab, Charles Nicolle Hospital; 2-University of Tunis ElManar, Faculty of Medicine of Tunis, LR99ES09, Tunis, Tunisia	1-Clinical and Experimental Pharmacology Lab, LR16SP02, National Center of Pharmacovigilance, University of Tunis El Manar, Tunis, Tunisia. 2-Neurodegenerative diseases and psychiatric troubles, LR18SP03, Razi Hospital, University of Tunis El Manar, Tunis, Tunisia. 3- Ministry of Health, National Observatory of New and Emerging Diseases, 1006, Tunis, Tunisia	Alia Ben Kahla; Asma Ferjani; Gaies Emna; Guedi Berrabeh; Hanen ElJebari; Ilhem Boutiba-Ben Boubaker; Jalila Ben Khelil; Maher Kharrat; Mouna Ben Sassi; Mouna Safer; Nissaf Ben Alaya; Riadh Daghfous; Riadh Gouider.; Salma Abid; Salwa Mrabet; Sameh Trabelsi; Sana Ferjani
EPI_ISL_451972	1. ViroGenetics - BSL3 Laboratory of Virology, Maopolska Centre of Biotechnology, Jagiellonian University; 2. II Department of Internal Medicine, Faculty of Medicine, Jagiellonian University Medical College; 3. DIAGNOSTYKA Ltd.	1. ViroGenetics - BSL3 Laboratory of Virology, Maopolska Centre of Biotechnology, Jagiellonian University; 2. II Department of Internal Medicine, Faculty of Medicine, Jagiellonian University Medical College.	Jakub Swadba; Krzysztof Pyr; Marcin Surmiak; Marek Sanak; Marta Rogalska-Kupiec; Monika Gsecka-Czapla; Pawe P abaj; Wojciech Branicki
EPI_ISL_729547	A. Krumbholz, Labor Dr. Krause und Kollegen MVZ GmbH, Kiel	Charité Universitätsmedizin Berlin, Institut für Virologie	Barbara Mühlemann; Christian Drosten; Julia Schneider; Jörn Beheim-Schwarzbach; Talitha Veith; Terry Jones; Victor M Corman
EPI_ISL_471546	AMA DR Jose Soares Hungria	Instituto Adolfo Lutz, Interdiciplinary Procedures Center, Strategic Laboratory	Claudia Regina Gonçalves; Claudio Tavares Sacchi; Erica Valessa Ramos Gomes
EPI_ISL_523989	AMA Jardim Joamar	Instituto Adolfo Lutz, Interdiciplinary Procedures Center, Strategic Laboratory	Claudia Regina Gonçalves; Claudio Tavares Sacchi; Erica Valessa Ramos Gomes
EPI_ISL_523990	AMA Jardim Peri	Instituto Adolfo Lutz, Interdiciplinary Procedures Center, Strategic Laboratory	Claudia Regina Gonçalves; Claudio Tavares Sacchi; Erica Valessa Ramos Gomes
EPI_ISL_682235, EPI_ISL_682258, EPI_ISL_682259	AREA DE SALUD ALAJUELA NORTE - CLINICA DR. MARCIAL RODRIGUEZ	Inciensa, Instituto Costarricense de Investigación y Enseñanza en Nutrición y Salud	Adriana Godinez; Adriana Godinez & Melany Calderon; Claudio Soto-Garita; Estela Cordero; Francisco Duarte; Hebleen Porras; Melany Calderon & Mariel López
EPI_ISL_682260	AREA DE SALUD CATEDRAL NORESTE	Inciensa, Instituto Costarricense de Investigación y Enseñanza en Nutrición y Salud	Adriana Godinez; Claudio Soto-Garita; Estela Cordero; Francisco Duarte; Hebleen Porras; Melany Calderon & Mariel López
EPI_ISL_682247, EPI_ISL_682248	AREA DE SALUD CIUDAD QUESADA	Inciensa, Instituto Costarricense de Investigación y Enseñanza en Nutrición y Salud	Adriana Godinez; Adriana Godinez & Melany Calderon; Claudio Soto-Garita; Estela Cordero; Francisco Duarte; Hebleen Porras; Melany Calderon & Mariel López
EPI_ISL_682237, EPI_ISL_682256	AREA DE SALUD CORREDORES	Inciensa, Instituto Costarricense de Investigación y Enseñanza en Nutrición y Salud	Adriana Godinez; Adriana Godinez & Melany Calderon; Claudio Soto-Garita; Estela Cordero; Francisco Duarte; Hebleen Porras; Melany Calderon & Mariel López
EPI_ISL_682250, EPI_ISL_682251, EPI_ISL_682252	AREA DE SALUD ESCAZU (COOPESANA)	Inciensa, Instituto Costarricense de Investigación y Enseñanza en Nutrición y Salud	Adriana Godinez & Melany Calderon; Claudio Soto-Garita; Estela Cordero; Francisco Duarte; Hebleen Porras
EPI_ISL_682240	AREA DE SALUD GOICOCHEA 1	Inciensa, Instituto Costarricense de Investigación y Enseñanza en Nutrición y Salud	Adriana Godinez & Melany Calderon; Claudio Soto-Garita; Estela Cordero; Francisco Duarte; Hebleen Porras
EPI_ISL_682238, EPI_ISL_682244	AREA DE SALUD LA CRUZ	Inciensa, Instituto Costarricense de Investigación y Enseñanza en Nutrición y Salud	Adriana Godinez; Adriana Godinez & Melany Calderon; Claudio Soto-Garita; Estela Cordero; Francisco Duarte; Hebleen Porras; Melany Calderon & Mariel López
EPI_ISL_682249	AREA DE SALUD LA UNION	Inciensa, Instituto Costarricense de Investigación y Enseñanza en Nutrición y Salud	Adriana Godinez; Claudio Soto-Garita; Estela Cordero; Francisco Duarte; Hebleen Porras; Melany Calderon & Mariel López
EPI_ISL_682236	AREA DE SALUD LOS CHILES	Inciensa, Instituto Costarricense de Investigación y Enseñanza en Nutrición y Salud	Adriana Godinez; Claudio Soto-Garita; Estela Cordero; Francisco Duarte; Hebleen Porras; Melany Calderon & Mariel López
EPI_ISL_445326, EPI_ISL_445363, EPI_ISL_445367	ASISTENCIA PUBLICA DR.ALEJANDRO DEL RIO	Instituto de Salud Publica de Chile	Alejandra Acevedo; Andrés E Castillo; Bárbara Parra; Carolina Tambley; Gabriel Leal; Jaime Lagos; Jorge Fernandez; Loredana Arata; Patricia Bustos; Paz Tapia; Rodrigo Fasce; Winston Andrade
EPI_ISL_426537, EPI_ISL_426558, EPI_ISL_427271, EPI_ISL_694079	AZ SPHL, Arizona Department of Health Services	TGen North	Ashlyn Pfeiffer; Chris French; Darrin Lemmer; Dave Engelthaler; Hayley Yaglom; Jolene Bowers; Megan Folkerts; The Arizona COVID Genomics Union (ACGU)
EPI_ISL_517613, EPI_ISL_517615, EPI_ISL_517616, EPI_ISL_517617, EPI_ISL_517618, EPI_ISL_517620, EPI_ISL_517621, EPI_ISL_517622, EPI_ISL_517623, EPI_ISL_517624, EPI_ISL_517625, EPI_ISL_517626, EPI_ISL_517627, EPI_ISL_517628, EPI_ISL_517629, EPI_ISL_517630, EPI_ISL_517631, EPI_ISL_517632, EPI_ISL_517633, EPI_ISL_517636, EPI_ISL_517637, EPI_ISL_517638, EPI_ISL_517639, EPI_ISL_517640, EPI_ISL_517641, EPI_ISL_517643, EPI_ISL_517644, EPI_ISL_517645, EPI_ISL_517646, EPI_ISL_517647, EPI_ISL_517648, EPI_ISL_517650, EPI_ISL_517651, EPI_ISL_517652, EPI_ISL_517653, EPI_ISL_517654, EPI_ISL_517657, EPI_ISL_517658, EPI_ISL_517659, EPI_ISL_517660, EPI_ISL_517661, EPI_ISL_517662, EPI_ISL_518799, EPI_ISL_518801, EPI_ISL_518802, EPI_ISL_518803, EPI_ISL_518804, EPI_ISL_518805, EPI_ISL_518806, EPI_ISL_518807, EPI_ISL_518808, EPI_ISL_518809, EPI_ISL_518810, EPI_ISL_518812, EPI_ISL_518814, EPI_ISL_518815, EPI_ISL_518816, EPI_ISL_518817, EPI_ISL_518818			
see above	Academic Hospital Paramaribo	Erasmus Medical Center	Bas Oude Munnink; Dion Gajadin; Ed Ijzerman; Emmanuelle Munger; Gary Gummels; Ingrid Krishnadath; Lycke Woittiez; Marion Koopmans; Mireille Van de Veer; Princes Wongsowidjojo; Radjesh Ori; Rohma Banwari; Stephen Vreden
EPI_ISL_427621	Alaska State Virology Laboratory	Alaska State Virology Laboratory	Chen, J.
EPI_ISL_805407, EPI_ISL_805581, EPI_ISL_805949, EPI_ISL_806092, EPI_ISL_806300, EPI_ISL_806319, EPI_ISL_806389, EPI_ISL_806410			
see above	Alberta Precision Labs (APL)	Alberta Precision Labs (APL)	Berenger B; Bernier F; Chui L; Croxen M; Gordon P; Kellner J; Lam LG; Li V; Ma R; Melin A; Pabbaraju K; Tipples G; Wong A; Zelyas N
EPI_ISL_515544, EPI_ISL_523984, EPI_ISL_523986	Ama Dr Jose Soares Hungria	Instituto Adolfo Lutz, Interdiciplinary Procedures Center, Strategic Laboratory	Claudia Regina Gonçalves; Claudio Tavares Sacchi; Erica Valessa Ramos Gomes
EPI_ISL_569875, EPI_ISL_569885	Amedeo di savoia	Crosetto lab, Karolinska Institutet, SciLifeLab	Anna Sapino; Luuk Harbers; Maria Grazia Milia; Michele Simonetti; Nicola Crosetto; Ning Zhang; Valeria Ghisetti
EPI_ISL_512663, EPI_ISL_512664, EPI_ISL_512669, EPI_ISL_527740, EPI_ISL_527749	Area De Salud Alajuela Norte - Clinica Dr. Marcial Rodriguez	Inciensa, Instituto Costarricense de Investigación y Enseñanza en Nutrición y Salud	Adriana Godinez & Melany Calderon; Claudio Soto-Garita; Estela Cordero; Francisco Duarte; Hebleen Porras
EPI_ISL_527756	Area De Salud Aserri	Inciensa, Instituto Costarricense de Investigación y Enseñanza en Nutrición y Salud	Adriana Godinez & Melany Calderon; Claudio Soto-Garita; Estela Cordero; Francisco Duarte; Hebleen Porras
EPI_ISL_770030	Area De Salud Buenos Aires	Inciensa, Instituto Costarricense de Investigación y Enseñanza en Nutrición y Salud	Adriana Godínez; Claudio Soto-Garita; Estela Cordero; Francisco Duarte; Hebleen Porras; Melany Calderón & Mariel López
EPI_ISL_769986, EPI_ISL_769994,	Area De Salud Catedral Noreste	Inciensa, Instituto Costarricense de Investigación y	Adriana Godínez; Claudio Soto-Garita; Estela Cordero; Francisco Duarte; Hebleen Porras; Melany Calderón & Mariel López

EPI_ISL_769997, EPI_ISL_769998, EPI_ISL_770021, EPI_ISL_770026		Enseñanza en Nutrición y Salud	
EPI_ISL_770011, EPI_ISL_770012	Area De Salud Coronado	Inciensa, Instituto Costarricense de Investigación y Enseñanza en Nutrición y Salud	Adriana Godínez; Claudio Soto-Garita; Estela Cordero; Francisco Duarte; Hebleen Porras; Melany Calderón & Mariel López
EPI_ISL_512668, EPI_ISL_527745, EPI_ISL_527748, EPI_ISL_527751, EPI_ISL_770008, EPI_ISL_770029	Area De Salud Corredores	Inciensa, Instituto Costarricense de Investigación y Enseñanza en Nutrición y Salud	Adriana Godinez & Melany Calderon; Adriana Godínez; Claudio Soto-Garita; Estela Cordero; Francisco Duarte; Hebleen Porras; Melany Calderón & Mariel López
EPI_ISL_770014	Area De Salud Curridabat 2	Inciensa, Instituto Costarricense de Investigación y Enseñanza en Nutrición y Salud	Adriana Godínez; Claudio Soto-Garita; Estela Cordero; Francisco Duarte; Hebleen Porras; Melany Calderón & Mariel López
EPI_ISL_512653	Area De Salud Desamparados 1 - Clinica Dr. Marcial Fallas [Grifo Alto/Desampara	Inciensa, Instituto Costarricense de Investigación y Enseñanza en Nutrición y Salud	Adriana Godinez & Melany Calderon; Claudio Soto-Garita; Estela Cordero; Francisco Duarte; Hebleen Porras
EPI_ISL_769993	Area De Salud El Guarco	Inciensa, Instituto Costarricense de Investigación y Enseñanza en Nutrición y Salud	Adriana Godínez; Claudio Soto-Garita; Estela Cordero; Francisco Duarte; Hebleen Porras; Melany Calderón & Mariel López
EPI_ISL_770009, EPI_ISL_770010	Area De Salud Escazu (Coopesana)	Inciensa, Instituto Costarricense de Investigación y Enseñanza en Nutrición y Salud	Adriana Godínez; Adriana Godínez & Melany Calderón; Claudio Soto-Garita; Estela Cordero; Francisco Duarte; Hebleen Porras; Melany Calderón & Mariel López
EPI_ISL_512659	Area De Salud Fortuna	Inciensa, Instituto Costarricense de Investigación y Enseñanza en Nutrición y Salud	Adriana Godinez & Melany Calderon; Claudio Soto-Garita; Estela Cordero; Francisco Duarte; Hebleen Porras
EPI_ISL_527757	Area De Salud Goicoechea 1	Inciensa, Instituto Costarricense de Investigación y Enseñanza en Nutrición y Salud	Adriana Godinez & Melany Calderon; Claudio Soto-Garita; Estela Cordero; Francisco Duarte; Hebleen Porras
EPI_ISL_770016, EPI_ISL_770020	Area De Salud Goicoechea 2 - Clinica Dr. Jimenez Nuñez	Inciensa, Instituto Costarricense de Investigación y Enseñanza en Nutrición y Salud	Adriana Godínez; Claudio Soto-Garita; Estela Cordero; Francisco Duarte; Hebleen Porras; Melany Calderón & Mariel López
EPI_ISL_512662, EPI_ISL_512666, EPI_ISL_512667, EPI_ISL_512671, EPI_ISL_527746, EPI_ISL_527747, EPI_ISL_527760, EPI_ISL_770000, EPI_ISL_770001, EPI_ISL_770002, EPI_ISL_770003, EPI_ISL_770027			
see above	Area De Salud La Cruz	Inciensa, Instituto Costarricense de Investigación y Enseñanza en Nutrición y Salud	Adriana Godinez & Melany Calderon; Adriana Godínez; Claudio Soto-Garita; Estela Cordero; Francisco Duarte; Hebleen Porras; Melany Calderón & Mariel López
EPI_ISL_512660, EPI_ISL_512661	Area De Salud Los Chiles	Inciensa, Instituto Costarricense de Investigación y Enseñanza en Nutrición y Salud	Adriana Godinez & Melany Calderon; Claudio Soto-Garita; Estela Cordero; Francisco Duarte; Hebleen Porras
EPI_ISL_770005	Area De Salud Moravia	Inciensa, Instituto Costarricense de Investigación y Enseñanza en Nutrición y Salud	Adriana Godínez; Claudio Soto-Garita; Estela Cordero; Francisco Duarte; Hebleen Porras; Melany Calderón & Mariel López
EPI_ISL_512658	Area De Salud Orotina-San Mateo [Orotina/Alajuela]	Inciensa, Instituto Costarricense de Investigación y Enseñanza en Nutrición y Salud	Adriana Godinez & Melany Calderon; Claudio Soto-Garita; Estela Cordero; Francisco Duarte; Hebleen Porras
EPI_ISL_769999	Area De Salud Paraiso-Cervantes	Inciensa, Instituto Costarricense de Investigación y Enseñanza en Nutrición y Salud	Adriana Godínez; Claudio Soto-Garita; Estela Cordero; Francisco Duarte; Hebleen Porras; Melany Calderón & Mariel López
EPI_ISL_512656	Area De Salud Pavas (Coopesalud) [Pavas/San Jose]	Inciensa, Instituto Costarricense de Investigación y Enseñanza en Nutrición y Salud	Adriana Godinez & Melany Calderon; Claudio Soto-Garita; Estela Cordero; Francisco Duarte; Hebleen Porras
EPI_ISL_770017, EPI_ISL_770022	Area De Salud Perez Zeledon	Inciensa, Instituto Costarricense de Investigación y Enseñanza en Nutrición y Salud	Adriana Godínez; Claudio Soto-Garita; Estela Cordero; Francisco Duarte; Hebleen Porras; Melany Calderón & Mariel López
EPI_ISL_769988, EPI_ISL_769989, EPI_ISL_770015, EPI_ISL_770023	Area De Salud San Francisco-San Antonio (Coopesana)	Inciensa, Instituto Costarricense de Investigación y Enseñanza en Nutrición y Salud	Adriana Godínez; Claudio Soto-Garita; Estela Cordero; Francisco Duarte; Hebleen Porras; Melany Calderón & Mariel López
EPI_ISL_769996, EPI_ISL_770025	Area De Salud San Juan-San Diego-Concepcion 2	Inciensa, Instituto Costarricense de Investigación y Enseñanza en Nutrición y Salud	Adriana Godínez; Claudio Soto-Garita; Estela Cordero; Francisco Duarte; Hebleen Porras; Melany Calderón & Mariel López
EPI_ISL_770007	Area De Salud San Rafael	Inciensa, Instituto Costarricense de Investigación y Enseñanza en Nutrición y Salud	Adriana Godínez; Claudio Soto-Garita; Estela Cordero; Francisco Duarte; Hebleen Porras; Melany Calderón & Mariel López
EPI_ISL_512657	Area De Salud Tibas-Uruca-Merced - Clinica Dr. Clorito Picado [Tibas/San Jose]	Inciensa, Instituto Costarricense de Investigación y Enseñanza en Nutrición y Salud	Adriana Godinez & Melany Calderon; Claudio Soto-Garita; Estela Cordero; Francisco Duarte; Hebleen Porras
EPI_ISL_491446	Area de Salud Alajuela Central	Inciensa, Instituto Costarricense de Investigación y Enseñanza en Nutrición y Salud	Adriana Godinez & Melany Calderon; Claudio Soto-Garita; Estela Cordero; Francisco Duarte; Hebleen Brenes
EPI_ISL_434533, EPI_ISL_434535, EPI_ISL_491449	Area de Salud Alajuela Sur	Inciensa, Instituto Costarricense de Investigación y Enseñanza en Nutrición y Salud	Adriana Godinez & Melany Calderon; Claudio Soto-Garita; Estela Cordero; Francisco Duarte; Hebleen Brenes; Hebleen Porras
EPI_ISL_491437, EPI_ISL_491444	Area de Salud Escazu (Coopesana)	Inciensa, Instituto Costarricense de Investigación y Enseñanza en Nutrición y Salud	Adriana Godinez & Melany Calderon; Claudio Soto-Garita; Estela Cordero; Francisco Duarte; Hebleen Brenes
EPI_ISL_491457	Area de Salud Los Santos	Inciensa, Instituto Costarricense de Investigación y Enseñanza en Nutrición y Salud	Adriana Godinez & Melany Calderon; Claudio Soto-Garita; Estela Cordero; Francisco Duarte; Hebleen Brenes
EPI_ISL_491445	Area de Salud Mata Redonda	Inciensa, Instituto Costarricense de Investigación y Enseñanza en Nutrición y Salud	Adriana Godinez & Melany Calderon; Claudio Soto-Garita; Estela Cordero; Francisco Duarte; Hebleen Brenes
EPI_ISL_434539	Area de Salud Orotina	Inciensa, Instituto Costarricense de Investigación y Enseñanza en Nutrición y Salud	Adriana Godinez & Melany Calderon; Claudio Soto-Garita; Estela Cordero; Francisco Duarte; Hebleen Porras
EPI_ISL_500649, EPI_ISL_500662, EPI_ISL_500698	Area of Virology, Serology and Virology Division (SAViD), New South Wales Health Pathology Randwick	Area of Virology, Serology and Virology Division (SAViD), New South Wales Health Pathology Randwick	Rawlinson, W.
EPI_ISL_707892	Area of Virology, Serology and Virology Division (SAViD), New South Wales Health Pathology Randwick	Virology Research Laboratory; Area of Virology, Serology and Virology Division (SAViD), New South Wales Health Pathology Randwick	Au, J.; Bull, R.; Deveson, I.; Foster, C.; Rawlinson, W.; Ruiz Silva, M.; Van Hal, S.
EPI_ISL_475862	Austrian Agency for Health and Food Safety (AGES)	Bergthaler laboratory, CeMM Research Center for Molecular Medicine of the Austrian Academy of Sciences	Alexander Lercher; Alexandra Popa; Andreas Bergthaler; Benedikt Agerer; Christoph Bock; Daniela Schmid; Dorothee von Laer; Elisabeth Puchhammer-Stoeckl; Franz Allerberger; Gregor Hörmann; Guenter Weiss; Henrique Colaco; Jakob-Wendelin Genger; Jan Laine; Judith Aberle; Kinga Rigler-Hohenwarter; Lukas Endler; Manfred Nairz; Mark Smyth; Martin Senekowitsch; Michael Schuster; Peter Hufnagl; Rainer Gattringer; Stephan Aberle; Thomas Penz; Wegene Borena
EPI_ISL_458087	B.J. Medical College and Civil hospital	Gujarat Biotechnology Research Centre	A M Kadri; Afzal Ansari; Amit Kanani; Ankit Hinsu; Apurvasinh Puvar; Bhavesh Modi; Chaitanya Joshi; Dhaval Vaghela; Dinesh Kumar; Gaurishankar Shrimali; Janvi Raval; Kamlesh J Upadhyay; Komal Patel; Labdhi Pandya; Madhvi Joshi; Maharshi Pandya; Monika Gandhi; Nidhi Patel; Nitin Savaliya; Pinal Trivedi; Pranay Shah; Pritesh Sabara; R D Dixit; Raghawendra Kumar; Ramesh Pandit; Ramesh Patel; Snehal Bagatharia; Tejas Shah; Umang Mishra; Zarna Patel; Zuber Saiyed
EPI_ISL_445362	BUPA SERVICIOS CLINICOS S.A	Instituto de Salud Publica de Chile	Alejandra Acevedo; Andrés E Castillo; Bárbara Parra; Carolina Tambley; Gabriel Leal; Jaime Lagos; Jorge Fernandez; Loredana Arata; Patricia Bustos; Paz Tapia; Rodrigo Fasce; Winston Andrade

EPI_ISL_445081	Baylor College of Medicine	Baylor College of Medicine: HGSC	David Henke; Donna Muzny; Erin Nicholson; George Weissenberger; Ginger Metcalf; Harsha Doddapaneni; Hsu Chao; Hua Shen; Joseph F. Petrosino; Kavya Kottapalli; Kristi L. Hoffman; Matthew C. Ross; Matthew Wong; Pedro Piedra; Qingchang Meng; Richard Sucgang; Sara J.J. Cregeen; Sejal Salvi; Tulin Ayvaz; Vasanthi Avadhanula; Vipin Menon; Yimiti Meiheerguli; Zeineen Momin
EPI_ISL_509711, EPI_ISL_509712, EPI_ISL_509713, EPI_ISL_509714	Belize Ministry of Health	Pathogen Discovery, Respiratory Viruses Branch, Division of Viral Diseases, Centers for Disease Control and Prevention	Anna Uehara; Clinton Paden; Haibin Wang; Jing Zhang; Krista Queen; Suxiang Tong; Yan Li; Ying Tao
EPI_ISL_816743, EPI_ISL_816749, EPI_ISL_816761, EPI_ISL_816814	Bioinformatics and Biostatistics Lab, Advanced Sequencing Facility	COVID-19 Genomics UK (COG-UK) Consortium	Aengus Stewart; Chelsea Sawyer; Harshil Patel; Jerome Nicod; Laura Cubitt; Margaret Crawford
EPI_ISL_732546	Bundeswehr Institute of Microbiology	Bundeswehr Institute of Microbiology	Alexandra Rehn; Enrico Georgi; Malena Bestehorn-Willmann; Markus Antwerpen; Mathias Walter; Roman Wölfel; Sabine Zange
EPI_ISL_445320	C.C.SALUD FAMILIAR PADRE FELIX DONOSO G.	Instituto de Salud Publica de Chile	Alejandra Acevedo; Andrés E Castillo; Bárbara Parra; Carolina Tambley; Gabriel Leal; Jaime Lagos; Jorge Fernandez; Loredana Arata; Patricia Bustos; Paz Tapia; Rodrigo Fasce; Winston Andrade
EPI_ISL_445318	C.DE SALUD FAMILIAR PABLO NERUDA	Instituto de Salud Publica de Chile	Alejandra Acevedo; Andrés E Castillo; Bárbara Parra; Carolina Tambley; Gabriel Leal; Jaime Lagos; Jorge Fernandez; Loredana Arata; Patricia Bustos; Paz Tapia; Rodrigo Fasce; Winston Andrade
EPI_ISL_445266, EPI_ISL_445267	CENTRO ONCOLOGICO DEL NORTE	Instituto de Salud Publica de Chile	Alejandra Acevedo; Andrés E Castillo; Bárbara Parra; Carolina Tambley; Gabriel Leal; Jaime Lagos; Jorge Fernandez; Loredana Arata; Patricia Bustos; Paz Tapia; Rodrigo Fasce; Winston Andrade
EPI_ISL_750175	CENUR Este-Sede Rocha-UdelaR	Institut Pasteur de Montevideo	Ana Carolina Mendonça; Andres Lizasoain; Camila Simoes; Cecilia Alonso; Cecilia Salazar; Daiana Mir; Fernando Lopez-Tort; Fernando Motta; Gonzalo Bello; Ighor Arantes; Ignacio Ferrés; Jose Sotelo; Leticia Maya; Letícia Garay Martins; Luciana Appolinario; Lucia Spangenberg; Mailen Arleo; Mariana Brandes; Marilda Mendonça Siqueira; Marilda Tereza Mar da Rosa; Maria Jose Benitez-Galeano; Martín Graña; Matias Castells; Matias Victoria; Matías Salvo; Natalia Rego; Natalia Reyes; Pablo Smircich; Paola Cristina Resende; Rodney Colina; Tamara Fernandez-Calero; Tania Possi; Tatiana Schäffer Gregianini; Veronica Noya; Yasser Vega
EPI_ISL_751185, EPI_ISL_751187, EPI_ISL_751188	CENUR Litoral Norte - UdelaR, Salto, Uruguay	Institut Pasteur de Montevideo	Ana Carolina Mendonça; Andres Lizasoain; Camila Simoes; Cecilia Alonso; Cecilia Salazar; Daiana Mir; Fernando Lopez-Tort; Fernando Motta; Gonzalo Bello; Ighor Arantes; Ignacio Ferrés; Jose Sotelo; Leticia Maya; Letícia Garay Martins; Luciana Appolinario; Lucia Spangenberg; Mailen Arleo; Mariana Brandes; Marilda Mendonça Siqueira; Marilda Tereza Mar da Rosa; Maria Jose Benitez-Galeano; Martín Graña; Matias Castells; Matias Victoria; Matías Salvo; Natalia Rego; Natalia Reyes; Pablo Smircich; Paola Cristina Resende; Rodney Colina; Tamara Fernandez-Calero; Tania Possi; Tatiana Schäffer Gregianini; Veronica Noya; Yasser Vega
EPI_ISL_445316	CESFAM BALMACEDA DE RENCA	Instituto de Salud Publica de Chile	Alejandra Acevedo; Andrés E Castillo; Bárbara Parra; Carolina Tambley; Gabriel Leal; Jaime Lagos; Jorge Fernandez; Loredana Arata; Patricia Bustos; Paz Tapia; Rodrigo Fasce; Winston Andrade
EPI_ISL_420044	CH Jean de Navarre Laboratoire de Biologie	National Reference Center for Viruses of Respiratory Infections, Institut Pasteur, Paris	Angela Brisebarre; Etienne Simon-Lorière; Flora Donati; Marion Barbet; Maud Vanpeene; Mélanie Albert; Méline Bizard; Sylvie Behillil; Sylvie van der Werf; Vincent Enouf
EPI_ISL_649964	CHU Bordeaux	CNR Virus des Infections Respiratoires - France SUD	Antonin Bal; Bruno Lina; Camille Ciccone; Gregory Destras; Gwendolyne Burfin; Hadrien Règue; Isabelle Garrigue; Laurence Josset; Marie-Edith Lafon; Martine Valette; Pantxika Bellecave; Pascale Trimoulet; Quentin Semanas
EPI_ISL_660690	CHU Montpellier	CNR Virus des Infections Respiratoires - France SUD	Antonin Bal; Bruno Lina; Gregory Destras; Gwendolyne Burfin; Hadrien Règue; Laurence Josset; Martine Valette; Michel Segondy; Quentin Semanas; Vincent Foulongne
EPI_ISL_663263	CHU Nantes	CNR Virus des Infections Respiratoires - France SUD	Antonin Bal; Bruno Lina; Celine Bressollette; Gregory Destras; Gwendolyne Burfin; Hadrien Règue; Laurence Josset; Louise Castain; Martine Valette; Quentin Semanas; Virginie Ferré
EPI_ISL_660373	CHU Toulouse	CNR Virus des Infections Respiratoires - France SUD	Antonin Bal; Bruno Lina; Gregory Destras; Gwendolyne Burfin; Hadrien Règue; Jean Michel Mansuy; Laurence Josset; Martine Valette; Quentin Semanas
EPI_ISL_641531	CHU de Nice - Hôpital Archet 13	CNR Virus des Infections Respiratoires - France SUD	Antonin Bal; Bruno Lina; Gregory Destras; Gwendolyne Burfin; Géraldine Gonfrier; Hadrien Règue; Laurence Josset; Martine Valette; Quentin Semanas; Valérie Giordanengo
EPI_ISL_641535, EPI_ISL_641536, EPI_ISL_660329, EPI_ISL_660331, EPI_ISL_660341, EPI_ISL_660345, EPI_ISL_660347			
see above	CHU de Saint-Étienne Hôpital Nord	CNR Virus des Infections Respiratoires - France SUD	Antonin Bal; Bruno Lina; Bruno Pozzetto; Gregory Destras; Gwendolyne Burfin; Hadrien Règue; Issam Bechri; Laurence Josset; Manon Vogrig; Marine Delorme; Martine Valette; Quentin Semanas; Sylvie Gonzalo; Sylvie Pillet; Thomas Bourlet
EPI_ISL_445245, EPI_ISL_445248, EPI_ISL_445250, EPI_ISL_445253, EPI_ISL_445254, EPI_ISL_445255, EPI_ISL_445258, EPI_ISL_445260			
see above	CLINICA ALEMANA DE SANTIAGO S.A.	Instituto de Salud Publica de Chile	Alejandra Acevedo; Andrés E Castillo; Bárbara Parra; Carolina Tambley; Gabriel Leal; Jaime Lagos; Jorge Fernandez; Loredana Arata; Patricia Bustos; Paz Tapia; Rodrigo Fasce; Winston Andrade
EPI_ISL_445272, EPI_ISL_445285, EPI_ISL_445287	CLINICA CIUDAD DEL MAR	Instituto de Salud Publica de Chile	Alejandra Acevedo; Andrés E Castillo; Bárbara Parra; Carolina Tambley; Gabriel Leal; Jaime Lagos; Jorge Fernandez; Loredana Arata; Patricia Bustos; Paz Tapia; Rodrigo Fasce; Winston Andrade
EPI_ISL_445297	CLINICA INTEGRAL S.A.	Instituto de Salud Publica de Chile	Alejandra Acevedo; Andrés E Castillo; Bárbara Parra; Carolina Tambley; Gabriel Leal; Jaime Lagos; Jorge Fernandez; Loredana Arata; Patricia Bustos; Paz Tapia; Rodrigo Fasce; Winston Andrade
EPI_ISL_445256, EPI_ISL_445259	CLINICA LAS CONDES S.A.	Instituto de Salud Publica de Chile	Alejandra Acevedo; Andrés E Castillo; Bárbara Parra; Carolina Tambley; Gabriel Leal; Jaime Lagos; Jorge Fernandez; Loredana Arata; Patricia Bustos; Paz Tapia; Rodrigo Fasce; Winston Andrade
EPI_ISL_445282, EPI_ISL_445283, EPI_ISL_445290, EPI_ISL_445291, EPI_ISL_445292, EPI_ISL_445299	CLINICA MAGALLANES S.A.	Instituto de Salud Publica de Chile	Alejandra Acevedo; Andrés E Castillo; Bárbara Parra; Carolina Tambley; Gabriel Leal; Jaime Lagos; Jorge Fernandez; Loredana Arata; Patricia Bustos; Paz Tapia; Rodrigo Fasce; Winston Andrade
EPI_ISL_445264	CLINICA REDSALUD VITACURA.	Instituto de Salud Publica de Chile	Alejandra Acevedo; Andrés E Castillo; Bárbara Parra; Carolina Tambley; Gabriel Leal; Jaime Lagos; Jorge Fernandez; Loredana Arata; Patricia Bustos; Paz Tapia; Rodrigo Fasce; Winston Andrade
EPI_ISL_445249	CLINICA SANTA MARIA S.A.	Instituto de Salud Publica de Chile	Alejandra Acevedo; Andrés E Castillo; Bárbara Parra; Carolina Tambley; Gabriel Leal; Jaime Lagos; Jorge Fernandez; Loredana Arata; Patricia Bustos; Paz Tapia; Rodrigo Fasce; Winston Andrade
EPI_ISL_445257, EPI_ISL_445262	CLINICA TABANCURA	Instituto de Salud Publica de Chile	Alejandra Acevedo; Andrés E Castillo; Bárbara Parra; Carolina Tambley; Gabriel Leal; Jaime Lagos; Jorge Fernandez; Loredana Arata; Patricia Bustos; Paz Tapia; Rodrigo Fasce; Winston Andrade
EPI_ISL_445306, EPI_ISL_445312, EPI_ISL_445315, EPI_ISL_445361	CLINICA UC SAN CARLOS DE APOQUINDO	Instituto de Salud Publica de Chile	Alejandra Acevedo; Andrés E Castillo; Bárbara Parra; Carolina Tambley; Gabriel Leal; Jaime Lagos; Jorge Fernandez; Loredana Arata; Patricia Bustos; Paz Tapia; Rodrigo Fasce; Winston Andrade
EPI_ISL_445334	CLINICA UNIVERSITARIA DE PUERTO MONTT S.A.	Instituto de Salud Publica de Chile	Alejandra Acevedo; Andrés E Castillo; Bárbara Parra; Carolina Tambley; Gabriel Leal; Jaime Lagos; Jorge Fernandez; Loredana Arata; Patricia Bustos; Paz Tapia; Rodrigo Fasce; Winston Andrade
EPI_ISL_445330	CLINICA VESPUCIO S. A.	Instituto de Salud Publica de Chile	Alejandra Acevedo; Andrés E Castillo; Bárbara Parra; Carolina Tambley; Gabriel Leal; Jaime Lagos; Jorge Fernandez; Loredana Arata; Patricia Bustos; Paz Tapia; Rodrigo Fasce; Winston Andrade
EPI_ISL_508997, EPI_ISL_676546, EPI_ISL_676561	CNR Virus des Infections Respiratoires - France SUD	CNR Virus des Infections Respiratoires - France SUD	Alexandre Gaymard; Antonin Bal; Bruno Lina; Carine Moustaud; Florence Morfin-Sherpa; Gregory Destras; Gwendolyne Burfin; Laurence Josset; Martine Valette; Maude Bouscambert-Duchamp; Raphaelle Lamy; Solenne Brun
EPI_ISL_434538	COOPESAIN	Inciensa, Instituto Costarricense de Investigación y Enseñanza en Nutrición y Salud	Adriana Godinez & Melany Calderon; Claudio Soto-Garita; Estela Cordero; Francisco Duarte; Hebleen Porras
EPI_ISL_735408	COVID 19 Centro de Combate ao Coronavirus CCC Jandira	Instituto Adolfo Lutz, Interdiciplinary Procedures Center, Strategic Laboratory	Claudia Regina Gonçalves; Claudio Tavares Sacchi; Erica Valessa Ramos Gomes; Karoline Rodrigues Campos
EPI_ISL_574593, EPI_ISL_574596,	CS II Dr. Antonio Vicoso Moreira de Rezende Sumare	Instituto Adolfo Lutz, Interdiciplinary Procedures Center,	Claudia Regina Gonçalves; Claudio Tavares Sacchi; Erica Valessa Ramos Gomes; Karoline Rodrigues Campos

EPI_ISL_583494		Strategic Laboratory	
EPI_ISL_450327, EPI_ISL_450331	CSIR-Centre for Cellular and Molecular Biology	CSIR-Centre for Cellular and Molecular Biology	Archana Bharadwaj Siva; Deepak Kumar; Devi Prasad Vijayashankara; Dhiviya Vedagiri; Disha Nanda; Divya Das; Divya Gupta; Divya Tej Sowpati; G. Aditya Kumar; Gangumala Srinivas Reddy; Jotin Gogoi; Karthik Bharadwaj Tallapaka; Koushick Sivakumar; Krishnan Harinivas Harshan; Lamuk Zaveri; Manish Bhattacharjee; Namami Gaur; Payel Mukherjee; Pooja Ramesh Gupta; Priya Singh; Purushotham Vodnala; Rajan Kumar Jha; Rakesh K Mishra; Ravi Prasad Mukku; Renu Sudhakar; Sakshi Shambhavi; Santosh Kumar Kuncha; Shagufta Khan; Shraddha Vijay Lahoti; Sofia Banu; Somesh Gorde; Sujoy Deb; Swati Bayyana; Tulasi Nagabandi; Vishal Sah; Zeba Rizvi
EPI_ISL_468314, EPI_ISL_583503	CTA Centro de Testagem e Aconselhamento	Instituto Adolfo Lutz, Interdiciplinary Procedures Center, Strategic Laboratory	Claudia Regina Gonçalves; Claudio Tavares Sacchi; Erica Valessa Ramos Gomes; Karoline Rodrigues Campos
EPI_ISL_445303	CTRO.DE SALUD FAMILIAR DR. RAUL YAZIGI	Instituto de Salud Publica de Chile	Alejandra Acevedo; Andrés E Castillo; Bárbara Parra; Carolina Tambley; Gabriel Leal; Jaime Lagos; Jorge Fernandez; Loredana Arata; Patricia Bustos; Paz Tapia; Rodrigo Fasce; Winston Andrade
EPI_ISL_582363, EPI_ISL_582394, EPI_ISL_582485	Cadham Provincial Laboratory	National Microbiology Laboratory (NML)	Anna Majer; Anneliese Landgraff; CanCOGeN's metadata curation team; Darian Hole; David Alexander; Elsie Grudeski; Gary Van Domselaar; Grace Seo; Jared Bullard; Jennifer Tanner; Kerry Dust; Madison Chapel; Morag Graham; Natalie Knox; Nathalie Bastien; Paul Van Caeseele; Philip Mabon; Public Health Agency of Canada CanCOGeN team; Rhiannon Huzarewich; Russell Mandes; Shari Tyson; Timothy Booth; Yan Li
EPI_ISL_515898	California Department of Public Health	California Department of Public Health	CDPH IDLB COVIDNet
EPI_ISL_583504, EPI_ISL_583505	Casa de Saude Stella Maris	Instituto Adolfo Lutz, Interdiciplinary Procedures Center, Strategic Laboratory	Claudia Regina Gonçalves; Claudio Tavares Sacchi; Erica Valessa Ramos Gomes; Karoline Rodrigues Campos
EPI_ISL_693235	Casmi Centro Atendimento Saude da Mulher e Infancia	Instituto Adolfo Lutz, Interdiciplinary Procedures Center, Strategic Laboratory	Claudia Regina Gonçalves; Claudio Tavares Sacchi; Erica Valessa Ramos Gomes; Karoline Rodrigues Campos
EPI_ISL_475577, EPI_ISL_475602, EPI_ISL_475635, EPI_ISL_475674, EPI_ISL_475680, EPI_ISL_475685	Cedars-Sinai Medical Center, Department of Pathology & Laboratory Medicine, Molecular Pathology Laboratory	Cedars-Sinai Medical Center, Molecular Pathology Laboratory of Department of Pathology & Laboratory Medicine and Genomic Core	Brian Davis; Eric Vail; Jasmine T Plummer; Jean Lopategui; Jianbo Song; John Paul Govindavari; Jong Taek Kim; Stephanie Chen; Wenjuan Zhang
EPI_ISL_756293, EPI_ISL_756294	Center for Biotechnology and Cell Therapy, São Rafael Hospital, Salvador, Brazil	Center for Biotechnology and Cell Therapy, São Rafael Hospital, Salvador, Brazil	Ana Verena Almeida Mendes; Bruno Solano de Freitas Souza; Carolina Kymie Vasques Nonaka; Marta Giovanetti; Marília Miranda Franco; Renato Santana de Aguiar; Tiago Gräf
EPI_ISL_459856, EPI_ISL_459857, EPI_ISL_459858, EPI_ISL_459859, EPI_ISL_459860, EPI_ISL_459861, EPI_ISL_459862, EPI_ISL_459863, EPI_ISL_459864, EPI_ISL_468752, EPI_ISL_468760			
see above	Center for Genome Regulation (CRG)	Center for Mathematical Modeling and Center for Genome Regulation. Santiago, Chile	Allende ML; Gaete A; González M.; Maass A; Palma R; Travisany D; Urra C; Varas M
EPI_ISL_438079, EPI_ISL_438102, EPI_ISL_475787, EPI_ISL_583724	Center for Virology, Medical University of Vienna	Bergthaler laboratory, CeMM Research Center for Molecular Medicine of the Austrian Academy of Sciences	Adi Steinrigl; Alexander Lercher; Alexandra Popa; Andreas Bergthaler; Benedikt Agerer; Christian Paar; Christoph Bock; Daniela Schmid; Dorothee von Laer; Elisabeth Puchhammer-Stoeckl; Franz Allerberger; Gernot Walder; Gregor Hörmann; Guenter Weiss; Gunther Vogl; Henrique Colaco; Jakob-Wendelin Genger; Jan Laine; Judith Aberle; Kinga Rigler-Hohenwarter; Lukas Endler; Manfred Nairz; Mark Smyth; Martin Senekowitsch; Michael Schuster; Peter Hufnagl; Peter Obrist; Rainer Gattringer; Sabine Sussitz-Rack; Stephan Aberle; Thomas Penz; Wegene Borena
EPI_ISL_815398, EPI_ISL_815399	Centogene	Centogene	Krishna Kumar Kandaswamy; Peter Bauer; Vivi Hue-Trang Lieu
EPI_ISL_429667, EPI_ISL_429669, EPI_ISL_429671, EPI_ISL_429674, EPI_ISL_429676, EPI_ISL_429679, EPI_ISL_429681, EPI_ISL_429684, EPI_ISL_429687, EPI_ISL_429688, EPI_ISL_429689, EPI_ISL_429695, EPI_ISL_429702			
see above	Central Public Health Laboratory/Octávio Magalhães Institute (IOM) from the Ezequiel Dias Foundation (FUNED)	Instituto Octávio Magalhães / Fundação Ezequiel Dias (IOM/Funed)	Joilson Xavier; Luiz Carlos Junior Alcantara; Marcos Vinícius Silva; Marluce Aparecida Assunção Oliveira; Marta Giovanetti; Talita Adelino; Vagner Fonseca
EPI_ISL_693197	Central de Rede de Frio Municipal	Instituto Adolfo Lutz, Interdiciplinary Procedures Center, Strategic Laboratory	Claudia Regina Gonçalves; Claudio Tavares Sacchi; Erica Valessa Ramos Gomes; Karoline Rodrigues Campos
EPI_ISL_536224	Centre hospitalier Anna-Laberge	Laboratoire de santé publique du Québec	Guillaume Bourque; Ioannis Ragoussis; Jesse Shapiro; Mark Lathrop and Michel Roger; Sandrine Moreira
EPI_ISL_700379, EPI_ISL_700387, EPI_ISL_700405	Centre hospitalier Métropole Savoie	CNR Virus des Infections Respiratoires - France SUD	Antonin Bal; Bruno Lina; Carine Dumollard; Gregory Destras; Gwendolyne Burfin; Hadrien Règue; Jérôme Grosjean; Laurence Josset; Martine Valette; Quentin Semanas
EPI_ISL_536115	Centre hospitalier régional du Grand Portage	Laboratoire de santé publique du Québec	Guillaume Bourque; Ioannis Ragoussis; Jesse Shapiro; Mark Lathrop and Michel Roger; Sandrine Moreira
EPI_ISL_515547	Centro Medico da Policia Militar do Estado de Sao Paulo	Instituto Adolfo Lutz, Interdiciplinary Procedures Center, Strategic Laboratory	Claudia Regina Gonçalves; Claudio Tavares Sacchi; Erica Valessa Ramos Gomes
EPI_ISL_693248	Centro Municipal de Epidemiologia e Imunizações	Instituto Adolfo Lutz, Interdiciplinary Procedures Center, Strategic Laboratory	Claudia Regina Gonçalves; Claudio Tavares Sacchi; Erica Valessa Ramos Gomes; Karoline Rodrigues Campos
EPI_ISL_693244	Centro Médico da Polícia Militar do Estado de Sao Paulo	Instituto Adolfo Lutz, Interdiciplinary Procedures Center, Strategic Laboratory	Claudia Regina Gonçalves; Claudio Tavares Sacchi; Erica Valessa Ramos Gomes; Karoline Rodrigues Campos
EPI_ISL_512670, EPI_ISL_527742, EPI_ISL_527755	Centro Nacional De Rehabilitacion Humberto Araya Rojas (Cenare)	Inciensa, Instituto Costarricense de Investigación y Enseñanza en Nutrición y Salud	Adriana Godinez & Melany Calderon; Claudio Soto-Garita; Estela Cordero; Francisco Duarte; Hebleen Porras
EPI_ISL_837550, EPI_ISL_837553, EPI_ISL_837554, EPI_ISL_837555, EPI_ISL_837556, EPI_ISL_837557, EPI_ISL_837558, EPI_ISL_837559, EPI_ISL_837560, EPI_ISL_837561, EPI_ISL_837562, EPI_ISL_837563, EPI_ISL_837564, EPI_ISL_837565, EPI_ISL_837566, EPI_ISL_837567, EPI_ISL_837568, EPI_ISL_837569, EPI_ISL_837570, EPI_ISL_837571, EPI_ISL_837572, EPI_ISL_837573, EPI_ISL_837574, EPI_ISL_837575, EPI_ISL_837576, EPI_ISL_837577, EPI_ISL_837578			
see above	Centro Nacional de Enfermedades Tropicales (CENETROP)	Laboratory of Respiratory Viruses and Measles, Oswaldo Cruz Institute, FIOCRUZ	Ana Carolina Mendonca; Anna Carolina Paixao; Cinthia Avila; Fernando Motta; Luciana Appolinario; Marilda Siqueira; Paola Resende; Roxana Loayza
EPI_ISL_629011, EPI_ISL_629012, EPI_ISL_629013, EPI_ISL_629014, EPI_ISL_629015, EPI_ISL_629016, EPI_ISL_629017, EPI_ISL_629018, EPI_ISL_629019, EPI_ISL_629020, EPI_ISL_629021, EPI_ISL_629022, EPI_ISL_629023, EPI_ISL_629024, EPI_ISL_629025, EPI_ISL_629026, EPI_ISL_629027, EPI_ISL_629028			
see above	Centro de Biotecnología Vegetal, Universidad Andrés Bello, Center for Genome Regulation	Center for Mathematical Modeling and Center for Genome Regulation. Santiago, Chile	Allende ML; Arriagada G; Bastias M; Bustos F; Castro E; González M; M; Maass A; Meneses C.; Montecino; Orellana A; Sanhueza D; Travisany D
EPI_ISL_483065, EPI_ISL_509430, EPI_ISL_509431, EPI_ISL_509432, EPI_ISL_509433, EPI_ISL_509434, EPI_ISL_509435, EPI_ISL_510536, EPI_ISL_529139			
see above	Centro de Desenvolvimento Tecnologico em Saude, Fundacao Oswaldo Cruz	Centro de Desenvolvimento Tecnologico em Saude, Fundacao Oswaldo Cruz	A.D.; Barroso; C.Q.; C.Q. and Medeiros; De Paula; F.B.; Ferreira; Fintelman-Rodrigues, N.; Gregorio; J.S.; M.A.; M.L.; Medeiros; Oliveira; S.P.; Sacramento; Saraiva; Souza; T.M.; Tschoeke, D.
EPI_ISL_510541, EPI_ISL_529140	Centro de Desenvolvimento Tecnologico em Saude, Fundacao Oswaldo Cruz	Centro de Desenvolvimento Tecnologico em Saude, Fundacao Oswaldo Cruz	A.D.; C.Q.; C.Q. and Medeiros; De Paula; F.B.; Ferreira; Fintelman-Rodrigues, N.; M.A.; Medeiros; Sacramento; Saraiva; Souza; T.M.
EPI_ISL_635479, EPI_ISL_635480, EPI_ISL_635481, EPI_ISL_635482, EPI_ISL_635483, EPI_ISL_635484, EPI_ISL_635485, EPI_ISL_635486, EPI_ISL_635487, EPI_ISL_635488, EPI_ISL_635489, EPI_ISL_635490, EPI_ISL_635491, EPI_ISL_635492, EPI_ISL_635493, EPI_ISL_635494, EPI_ISL_635495, EPI_ISL_635496, EPI_ISL_635497, EPI_ISL_635498, EPI_ISL_635499, EPI_ISL_635500, EPI_ISL_635501, EPI_ISL_635502, EPI_ISL_635503, EPI_ISL_635504, EPI_ISL_635505, EPI_ISL_635506, EPI_ISL_635507, EPI_ISL_635508, EPI_ISL_635509, EPI_ISL_635510, EPI_ISL_635511, EPI_ISL_635512, EPI_ISL_635513, EPI_ISL_635514, EPI_ISL_635515, EPI_ISL_635516, EPI_ISL_635517, EPI_ISL_635518, EPI_ISL_635519, EPI_ISL_635520, EPI_ISL_635521, EPI_ISL_635522, EPI_ISL_635523, EPI_ISL_635524, EPI_ISL_635525, EPI_ISL_635526, EPI_ISL_635527, EPI_ISL_635528, EPI_ISL_635529, EPI_ISL_635530, EPI_ISL_635531, EPI_ISL_635532, EPI_ISL_635533, EPI_ISL_635534, EPI_ISL_635535, EPI_ISL_635536, EPI_ISL_635537, EPI_ISL_635538, EPI_ISL_635539, EPI_ISL_635540, EPI_ISL_635541, EPI_ISL_635542, EPI_ISL_635543, EPI_ISL_635544, EPI_ISL_635545, EPI_ISL_635546, EPI_ISL_635547, EPI_ISL_635548, EPI_ISL_635549, EPI_ISL_635550, EPI_ISL_635551, EPI_ISL_635552, EPI_ISL_635553, EPI_ISL_635554, EPI_ISL_635555, EPI_ISL_635556, EPI_ISL_635557, EPI_ISL_635558, EPI_ISL_635559, EPI_ISL_635560, EPI_ISL_635561, EPI_ISL_635562, EPI_ISL_635563, EPI_ISL_635564, EPI_ISL_635565, EPI_ISL_635566, EPI_ISL_635567, EPI_ISL_635568, EPI_ISL_635569, EPI_ISL_635570, EPI_ISL_635571, EPI_ISL_635572, EPI_ISL_635573, EPI_ISL_635574, EPI_ISL_635575, EPI_ISL_635576			
see above	Centro de Diagnstico COVID-19 UABC Tijuana	Andersen lab at Scripps Research	Germán Ibarra; Jonathan Vincent Baena; Jorge Luis Jiménez Niebla; Manuel Sánchez Alavez; Oscar Efrén Zazueta Fierro; SEARCH Alliance San Diego with Idanya Rubí Serafín Higuera
EPI_ISL_491933, EPI_ISL_491934, EPI_ISL_491935, EPI_ISL_491941, EPI_ISL_527818, EPI_ISL_527819, EPI_ISL_594118, EPI_ISL_697797			
see above	Centro de Investigaciones, Universidad de Especialidades Espíritu Santo	Institute of Microbiology, Universidad San Francisco de Quito	Belén Prado-Vivar; Bernardo Gutiérrez; Derly Andrade; Edith Lopez; Fernando Espinoza; Gabriel Morey; Gabriel Trueba; Jose Pedro Barberan; Juan Carlos Fernandez; Juan José Guadalupe; Michelle Grunauer; Monica Becerra-Wong; Patricio Rojas-Silva; Paúl Cárdenas; Ruben Armas; Sully Márquez;

			Verónica Barragán
EPI_ISL_500398, EPI_ISL_500429	Centro de Investigación Biomédica de La Rioja - Hospital San Pedro Logroño	SeqCOVID-SPAIN consortium/IBV(CSIC)	José Manuel Azcona Gutiérrez; María Pilar Bea Escudero; María de Toro; Miriam Blasco Alberdi and SeqCOVID-SPAIN consortium
EPI_ISL_583491	Centro de Saude Esf IV Zona Rual Domingos de SJ Rio Pardo	Instituto Adolfo Lutz, Interdiciplinary Procedures Center, Strategic Laboratory	Claudia Regina Gonçalves; Claudio Tavares Sacchi; Erica Valessa Ramos Gomes; Karoline Rodrigues Campos
EPI_ISL_471543, EPI_ISL_583500	Centro de Saude I Tacito Leite de Carvalho e Silva	Instituto Adolfo Lutz, Interdiciplinary Procedures Center, Strategic Laboratory	Claudia Regina Gonçalves; Claudio Tavares Sacchi; Erica Valessa Ramos Gomes; Karoline Rodrigues Campos
EPI_ISL_735416	Centro de Saude II Dr Jose Paione Mococa	Instituto Adolfo Lutz, Interdiciplinary Procedures Center, Strategic Laboratory	Claudia Regina Gonçalves; Claudio Tavares Sacchi; Erica Valessa Ramos Gomes; Karoline Rodrigues Campos
EPI_ISL_468305, EPI_ISL_468307, EPI_ISL_735411, EPI_ISL_735423, EPI_ISL_735424, EPI_ISL_735426	Centro de Vigilancia a Saude de Diadema	Instituto Adolfo Lutz, Interdiciplinary Procedures Center, Strategic Laboratory	Claudia Regina Gonçalves; Claudio Tavares Sacchi; Erica Valessa Ramos Gomes; Karoline Rodrigues Campos
EPI_ISL_693240, EPI_ISL_693242	Centro de Vigilância a Saude de Diadema	Instituto Adolfo Lutz, Interdiciplinary Procedures Center, Strategic Laboratory	Claudia Regina Gonçalves; Claudio Tavares Sacchi; Erica Valessa Ramos Gomes; Karoline Rodrigues Campos
EPI_ISL_753917, EPI_ISL_753919, EPI_ISL_753923, EPI_ISL_753945, EPI_ISL_754036, EPI_ISL_754041	Charité Universitätsmedizin Berlin, Institut für Virologie/Labor Berlin	Charité Universitätsmedizin Berlin, Institut für Virologie	Barbara Mühlemann; Christian Drosten; Julia Schneider; Jörn Beheim-Schwarzbach; Talitha Veith; Terry Jones; Victor M Corman
EPI_ISL_468070	Child Health Research Foundation	Child Health Research Foundation	Hafizur Rahman; Maksuda Islam; Md Saiful Islam Sajib; Roly Malaker; Samir K Saha; Senjuti Saha
EPI_ISL_414579	Clinica Alemana de Santiago, Chile	Instituto de Salud Publica de Chile	Alejandra Acevedo; Andrés E. Castillo; Bárbara Parra; Carolina Tambley; Gabriel Leal; Gisselle Barra; Jaime Lagos; Javier Tognarelli; Jorge Fernández.; Loredana Arata; Patricia Bustos; Paz Tapia; Rodrigo Fasce; Soledad Ulloa; Winston Andrade
EPI_ISL_527759, EPI_ISL_770004	Clinica Biblica	Inciensa, Instituto Costarricense de Investigación y Enseñanza en Nutrición y Salud	Adriana Godinez & Melany Calderon; Adriana Godínez; Claudio Soto-Garita; Estela Cordero; Francisco Duarte; Hebleen Porras; Melany Calderón & Karla Gutiérrez-González
EPI_ISL_414580	Clinica Santa Maria, Santiago, Chile	Instituto de Salud Publica de Chile	Alejandra Acevedo; Andrés E. Castillo; Bárbara Parra; Carolina Tambley; Gabriel Leal; Gisselle Barra; Jaime Lagos; Javier Tognarelli; Jorge Fernández.; Loredana Arata; Patricia Bustos; Paz Tapia; Rodrigo Fasce; Soledad Ulloa; Winston Andrade
EPI_ISL_462454, EPI_ISL_462465, EPI_ISL_462474	Clinical Center, University of Sarajevo	Charite Universitatsmedizin Berlin, Institute of Virology	Almedina Hadzihasanovic-Moro; Amela Dedeic-Ljubovic; Barbara Muehlemann; Christian Drosten; Irma Salimovic-Besic; Jorn Beheim-Schwarzbach; Julia Schneider; Selma Mutevelic; Suzana Arapcic; Talitha Veith; Terry Jones; Victor M Corman
EPI_ISL_421279	Clinical Diagnostics Laboratory, Diagnostic & Experimental Pathology, Lilly Research Laboratories	Clinical Diagnostics Laboratory, Diagnostic & Experimental Pathology, Lilly Research Laboratories	Andrew Schade; Angie Fulford; Erin Wray; Jeff Fill; Joe Oakley; John Calley; John McElwee; Leslie O'Neill Reising; Mayuri Vaidya; Pat Finnegan; Phil Ebert; Rachael Redmond; Sam McNeely; Tim Holzer
EPI_ISL_450506	Clinical Laboratory, Hospital Israelita Albert Einstein	Clinical Laboratory, Hospital Israelita Albert Einstein	Amgarten, D.; Araujo; C.L.P.; D.B.; D.B.L.; Durigon; E.L. and Pinho; J.R.R.; Machado; Malta, F.; Mangueira; R.A.F.; R.R.G.; Santana; de Oliveira
EPI_ISL_605802, EPI_ISL_605808	Clinical Virology Laboratory, Institute of Liver and Biliary Sciences	ILBS - IGIB	Abhishek Padhi; Ekta Gupta; Jaswinder Singh Maras; Reshu Agarwal; Sheetalnath Rooge; Shridhar Sivasubbu; Shvetank Sharma; Vinod Scaria
EPI_ISL_447350	Clinical Virology Unit, Hadassah Hebrew University Medical Center	Stern Lab	Stern Lab
EPI_ISL_527403, EPI_ISL_527408	Colorado State University - Ebel Lab	Colorado State University - Ebel Lab	Greg Ebel et al.
EPI_ISL_583497	Complexo Hospitalar Ouro Verde de Campinas	Instituto Adolfo Lutz, Interdiciplinary Procedures Center, Strategic Laboratory	Claudia Regina Gonçalves; Claudio Tavares Sacchi; Erica Valessa Ramos Gomes; Karoline Rodrigues Campos
EPI_ISL_523970	Conjunto Hospitalar do Mandaqui	Instituto Adolfo Lutz, Interdiciplinary Procedures Center, Strategic Laboratory	Claudia Regina Gonçalves; Claudio Tavares Sacchi; Erica Valessa Ramos Gomes
EPI_ISL_468410	County of San Luis Obispo Public Health Laboratory	Chan-Zuckerberg Biohub	CZB Cliahub Consortium
EPI_ISL_693207	Cs II Doutor Antonio Vicoso Moreira de Rezende	Instituto Adolfo Lutz, Interdiciplinary Procedures Center, Strategic Laboratory	Claudia Regina Gonçalves; Claudio Tavares Sacchi; Erica Valessa Ramos Gomes; Karoline Rodrigues Campos
EPI_ISL_833167, EPI_ISL_833168, EPI_ISL_833169, EPI_ISL_833170, EPI_ISL_833171, EPI_ISL_833172, EPI_ISL_833173, EPI_ISL_833174, EPI_ISL_833175, EPI_ISL_833176			
see above	DB Diagnosticos do Brasil	Instituto Adolfo Lutz, Interdiciplinary Procedures Center, Strategic Laboratory	Claudia Regina Gonçalves; Claudio Tavares Sacchi; Erica Valessa Ramos Gomes; Karoline Rodrigues Campos
EPI_ISL_672666, EPI_ISL_672675, EPI_ISL_672679, EPI_ISL_672684, EPI_ISL_722134, EPI_ISL_722135, EPI_ISL_722136, EPI_ISL_722137, EPI_ISL_722138, EPI_ISL_722139, EPI_ISL_722140, EPI_ISL_804814, EPI_ISL_804815, EPI_ISL_804816, EPI_ISL_804817, EPI_ISL_804818, EPI_ISL_804819, EPI_ISL_804820, EPI_ISL_804821, EPI_ISL_804822, EPI_ISL_804823, EPI_ISL_804824, EPI_ISL_804825, EPI_ISL_804826, EPI_ISL_804827, EPI_ISL_804828, EPI_ISL_804829, EPI_ISL_804830			
see above	DB Diagnosticos do Brasil	Laboratório de Parasitologia Médica - Instituto de Medicina Tropical - Universidade de São Paulo	Andrew Rambaut; Brazil-UK Centre for Arbovirus Discovery Diagnosis Genomics and Epidemiology (CADDE) Genomic Network - Instituto de Medicina Tropical; CADDE Genomic Network.; CDL; Camila A. Maia da Silva; Cecília da Cunha Camilo; DB; Darlan Candido; Erika Regina Manuli; Ester C. Sabino; Flavia Cristina Sales; HEMOAM; Ingra Morales Claro; Lucas A. Moyses Franco; Maria do Perpétuo Socorro Sampaio Carvalho; Myuki Alfaia Esashika Crispim; Nelson Abrahim Fraiji; Nelson Gaburo; Nick Loman; Nuno Faria; Oliver G. Pybus; Pamela dos Santos Andrade; Renato A. Santana; Thais de Moura Coletti
EPI_ISL_476178, EPI_ISL_476180, EPI_ISL_476184, EPI_ISL_476188, EPI_ISL_476192, EPI_ISL_476194, EPI_ISL_476196, EPI_ISL_476200, EPI_ISL_476201, EPI_ISL_476218, EPI_ISL_476278, EPI_ISL_476281, EPI_ISL_476282, EPI_ISL_476286, EPI_ISL_476287, EPI_ISL_476288, EPI_ISL_476289, EPI_ISL_476292, EPI_ISL_476293, EPI_ISL_476296, EPI_ISL_476297, EPI_ISL_476298, EPI_ISL_476299, EPI_ISL_476303, EPI_ISL_476305, EPI_ISL_476307, EPI_ISL_476309, EPI_ISL_476311, EPI_ISL_476312, EPI_ISL_476313, EPI_ISL_476317, EPI_ISL_476318, EPI_ISL_476319, EPI_ISL_476320, EPI_ISL_476321, EPI_ISL_476322, EPI_ISL_476324, EPI_ISL_476327, EPI_ISL_476330, EPI_ISL_476331, EPI_ISL_476332, EPI_ISL_476333, EPI_ISL_476335, EPI_ISL_476336, EPI_ISL_476350, EPI_ISL_476351, EPI_ISL_476352, EPI_ISL_476359, EPI_ISL_476362, EPI_ISL_476366, EPI_ISL_476370			
see above	DB Diagnósticos do Brasil	Instituto de Medicina Tropical da Univesidade de São Paulo	Camila Alves Maia da Silva; Darlan da Silva Candido; Erika Regina Manuli; Ester Sabino; Flavia Cristina da Silva Sales; Giula Magalhaes Ferreira; Jaqueline Goes de Jesus; Julien Theze; Mariana Severo Ramundo; Nuno Faria; Samples: Nelson Gaburo Jr; Sequencing: Ingra Morales Claro; Thais de Moura Coletti
EPI_ISL_804845, EPI_ISL_804903, EPI_ISL_804906	DC Public Health Lab/ Dept. of Forensic Sciences	DC Public Health Lab/ Dept. of Forensic Sciences	Brittany Hamilton; Connie Maza; David Payne; Elizabeth Zelaya; Jocelyn Hauser; Monica Mann; Scott Nguyen
EPI_ISL_794661	DRECCION DE SANIDAD POLICIA NACIONAL	Instituto Nacional de Salud - Dirección de Investigación en Salud Pública	Carlos Franco-Muñoz; Diego A. Álvarez-Díaz; Diego Andrés Prada; Gerardo Santamaría; Jonathan Reales; Julian Naizaque; Katherine Laiton-Donato; Magdalena Wiesner; Marcela Mercado-Reyes; Maria T. Herrera; Martha Lucia Ospina Martinez; Mauricio Pacheco-Montealegre; Paola Muñoz-Laiton; Sheryl Corchuelo
EPI_ISL_561335, EPI_ISL_576162, EPI_ISL_576164, EPI_ISL_576166	Delaware Public Health Lab	Delaware Public Health Lab	Gregory Hovan
EPI_ISL_430796, EPI_ISL_430797, EPI_ISL_430798, EPI_ISL_430802, EPI_ISL_430805, EPI_ISL_430808, EPI_ISL_792301			
see above	Departamento de Biología y genética molecular, IACA Laboratorios.	Área de Secuenciación del Laboratorio de Virología del Hospital de Niños Dr. Ricardo Gutierrez on behalf of 'Proyecto Argentino Interinstitucional de genomica de SARS-CoV-2' (PAIS Consortium)	A; AS; E; Goya; LE; Lusso; MI; MS; Masciovecchio MV; Mistchenko; Nabaes Jodar; Natale; S; Streitenberger ER; Suárez; Tittarelli; Valinotto; Viegas, M.
EPI_ISL_444493	Departamento de Laboratorios de Salud Publica (DLSP, Division Epidemiologia, Ministerio de Salud Publica)	Facultad de Ciencias (Sección Genética Evolutiva, Sección Virología).	Arbiza; Calleros, L.; Chiparelli, H.; Coppola, L.; Delfraro, A.; Frabasile, S.; Fuques, E.; Goni, N.; Grecco, S.; J. and Perez, R.; Panzera, Y.; Ramos, N.; Ramos, V.; Techera, C.

EPI_ISL_603022, EPI_ISL_603034	Departamento de Vigilância à Saúde	Instituto Adolfo Lutz, Interdiciplinary Procedures Center, Strategic Laboratory	Claudia Regina Gonçalves; Claudio Tavares Sacchi; Erica Valessa Ramos Gomes; Karoline Rodrigues Campos
EPI_ISL_429284, EPI_ISL_452035	Department of Clinical Microbiology, Copenhagen University Hospital, Hvidovre, Kettegaard Alle 30, 2650 Hvidovre.	Albertsen lab, Department of Chemistry and Bioscience, Aalborg University, Denmark	Rasmus Kirkegaard
EPI_ISL_636991	Department of Infectious Diseases and Immunology, National Hospital Organization Nagoya Medical Center	Clinical Research Center, National Hospital Organization Nagoya Medical Center	Hirotaka Ode; Kazuhiro Matsuoka; Mai Kubota; Masakazu Matsuda; Mayumi Imahashi; Miho Nakasuji; Mikiko Mori; Yasumasa Iwatani; Yoshihiro Nakata; Yoshiyuki Yokomaku
EPI_ISL_457721	Department of Infectious Diseases, Istituto Superiore di Sanità, Roma , Italy	Army Medical and Veterinary Research Center	Alessandra Lo Presti; Anna Anselmo; Antonella Fortunato; Antonella Marchi; Concetta Fabiani Silvia Fillo; Eleonora Benedetti; Florigio Lista; Francesco Giordani; Giovanni Faggioni; Nino D'Amore; Paola Stefanelli; Riccardo De Sanctis; Stefano Fiore; Vanessa Vera Fain
EPI_ISL_516805	Department of Laboratory Medicine, Tan Tock Seng Hospital	Department of Laboratory Medicine, Tan Tock Seng Hospital	Barkham TMS; Chen YYC; Li C; Maurer-Stroh S; Nagarajan N; Sessions OM; Tang WY; Zair X
EPI_ISL_422422	Department of Laboratory Medicine, National Taiwan University Hospital	Microbial Genomics Core Lab, National Taiwan University Centers of Genomic and Precision Medicine	Chiao-Ling Li; Pei-Jer Chen; Shan-Chwen Chang; Shiou-Hwei Yeh; Sui-Yuan Chang; Ya-Yun Lai; You-Yu Lin
EPI_ISL_507003, EPI_ISL_648064	Department of Laboratory Medicine, Tan Tock Seng Hospital	Department of Laboratory Medicine, Tan Tock Seng Hospital	Barkham TMS; Chen YYC; Li C; Lim JX; Maurer-Stroh S; Nagarajan N; Sessions OM; Tang WY; Zair X
EPI_ISL_803883	Department of Medical Biotechnologies, University of Siena	Laboratory of Infectious Diseases, Department of Biomedical and Clinical Sciences L. Sacco, University of Milan	Alessia Lai; Annalisa Bergna; Carla Della Ventura; Claudia Balotta; Filippo Dragoni; Gianguglielmo Zehender on behalf of SARS-CoV-2 ITALIAN RESEARCH ENTERPRISE-(SCIRE) Collaborative Group; Ilaria Vicenti; Maria Grazia Cusi; Massimo Galli; Maurizio Zazzi
EPI_ISL_454416	Department of Medical Microbiology, Leiden University Medical Center	Department of Medical Microbiology, Leiden University Medical Center	Dalebout; E.J.; J.C.; J.J. and Sidorov, I.; N.S.; Ogando; Snijder; T.J.; Zevenhoven; de Vries
EPI_ISL_483861	Department of Microbiology, Government Medical College, Surat	Gujarat Biotechnology Research Centre	A M Kadri; Afzal Ansari; Amit gamit; Apurvasinh Puvar; Chaitanya Joshi; Dinesh Kumar; Harsh Bakshi; Janvi Raval; Komal Patel; Labdhi Pandya; Madhvi Joshi; Maharshi Pandya; Monika Gandhi; Naresh Chauhan; Nidhi Patel; Nikha Trivedi; Nitin Savaliya; Pinal Trivedi; R D Dixit; Raghawendra Kumar; Summaiya Mullan; Zarna Patel; Zuber Saiyed
EPI_ISL_497820	Department of Microbiology, The University of Hong Kong	Department of Microbiology, The University of Hong Kong	Kelvin K.W. To; Kwok-Yung Yuen
EPI_ISL_433746	Department of Pathology, University of Cambridge	COVID-19 Genomics UK (COG-UK) Consortium	Aminu S. Jahun; Anna Yakovleva; Charlotte J. Houldcroft; Fahad A Khokhar; Grant Hall; Ian Goodfellow; Laura G Caller; Luke W Meredith; M. Estee Torok; Martin D. Curran; Myra Hosmillo; Sarah L. Caddy; Theresa Feltwell; William L. Hamilton
EPI_ISL_441312	Department of Pathology, University of Cambridge	Wellcome Sanger Institute for the COVID-19 Genomics UK (COG-UK) consortium	Alex Alderton; Aminu S. Jahun; Anna Yakovleva; Charlotte J. Houldcroft; Cordelia Langford; David K. Jackson; Dominic Kwiatkowski; Ewan Harrison; Fahad A Khokhar; Grant Hall; Ian Goodfellow; Ian Johnston; John Sillitoe on behalf of the Wellcome Sanger Institute COVID-19 Surveillance Team (http://www.sanger.ac.uk/covid-team); Laura G Caller; Luke W Meredith; M. Estée Török; Martin D. Curran; Myra Hosmillo; Roberto Amato; Sarah L. Caddy; Sonia Goncalves; Theresa Feltwell; William L. Hamilton
EPI_ISL_614551, EPI_ISL_614618, EPI_ISL_614631, EPI_ISL_617949, EPI_ISL_618313, EPI_ISL_618639, EPI_ISL_622242, EPI_ISL_622579, EPI_ISL_622615			
see above	Department of Virus and Microbiological Special Diagnostics, Statens Serum Institut, Denmark	Albertsen lab, Department of Chemistry and Bioscience, Aalborg University, Denmark	Danish Covid-19 Genome Consortia
EPI_ISL_774874	Designated Reference Institute for Chemical Measurements (DRICM)	DNA SOLUTION LTD.	Abdul Khaleque; Abu Sufian; Hasan Ul Haider; Jannatun Naima; Kazi Nadim Hasan; MSM Chowdhury; Mala Khan; Mamudul Hasan Razu; Md. Imran Khan; Mizanur Rahman; Mohammad Fazle Alam Rabbi
EPI_ISL_763074, EPI_ISL_763075	Diagnosticos da America - DASA	Instituto Adolfo Lutz, Interdiciplinary Procedures Center, Strategic Laboratory	Claudia Regina Gonçalves; Claudio Tavares Sacchi; Erica Valessa Ramos Gomes; Karoline Rodrigues Campos
EPI_ISL_845623	Dirección de Sanidad Ejército	Instituto Nacional de Salud - Dirección de Investigación en Salud Pública	Carlos Franco-Muñoz; Diego A. Álvarez-Díaz; Diego Andrés Prada; Gerardo Santamaría; Jonathan Reales; Julian Naizaque; Katherine Laiton-Donato; Magdalena Wiesner; Marcela Mercado-Reyes; Maria T. Herrera-Sepúlveda; Martha Lucia Ospina Martinez; Mauricio Pacheco-Montealegre; Paola Muñoz-Laiton; Sheryll Corchuelo
EPI_ISL_534312	Distrito Sanitario Sul	Instituto Adolfo Lutz, Interdiciplinary Procedures Center, Strategic Laboratory	Claudia Regina Gonçalves; Claudio Tavares Sacchi; Erica Valessa Ramos Gomes
EPI_ISL_583499	Distrito Sanitario Sul Campinas	Instituto Adolfo Lutz, Interdiciplinary Procedures Center, Strategic Laboratory	Claudia Regina Gonçalves; Claudio Tavares Sacchi; Erica Valessa Ramos Gomes; Karoline Rodrigues Campos
EPI_ISL_498042, EPI_ISL_498046, EPI_ISL_506966, EPI_ISL_506967, EPI_ISL_506979	Division of Viral Diseases, Center for Laboratory Control of Infectious Diseases, Korea Centers for Diseases Control and Prevention	Division of Viral Diseases, Center for Laboratory Control of Infectious Diseases, Korea Centers for Diseases Control and Prevention	Daesang Lee; Dong Hyun Song; Heui Man Kim; Hye-Jun Jo; Jeong-Min Kim; Jun-Sub Kim; Myung Guk Han; Namjoo Lee; Sang Hee Woo; Seong Tae Jeong; Yoon-Seok Chung
EPI_ISL_481742, EPI_ISL_481748, EPI_ISL_481750	Dr. Georges-L.-Dumont University Hospital Centre	National Microbiology Laboratory	Anna Majer; Elsie Grudeski; Gary Van Domselaar; Grace Seo; Guillaume Desnoyers; Jennifer Tanner; Kristyn Burak; Morag Graham; Natalie Knox; Nathalie Bastien; Philip Mabon; Rhiannon Huzarewich; Richard Garceau; Russell Mandes; Shari Tyson; Timothy Booth; Yan Li
EPI_ISL_422616, EPI_ISL_455132, EPI_ISL_455134, EPI_ISL_460786, EPI_ISL_523504	Dutch COVID-19 response team	Erasmus Medical Center	Anne van der Linden; Annemiek van der Eijk; Aura Timen; Bas Oude Munnink; Claudia Schapendonk; Corien Swaan; Corine GeurtsvanKessel; David Nieuwenhuijse; Irina Chestakova; Jeroen van Kampen; Jolanda Voermans; Madelief Mollers; Manon Haverkate; Marion Koopmans; Mark Pronk; Mart Stein; Pascal Lexmond; Reina Sikkema; Richard Molenkamp; Sandra Kengne Kamga Mobou; Stefan van Nieuwkoop; Theo Bestebroer; on behalf of the Dutch national COVID-19 response team.
EPI_ISL_454758, EPI_ISL_454769, EPI_ISL_547445, EPI_ISL_547446, EPI_ISL_547447, EPI_ISL_547448, EPI_ISL_547449, EPI_ISL_547450, EPI_ISL_547451, EPI_ISL_547452, EPI_ISL_547453, EPI_ISL_636492, EPI_ISL_636493, EPI_ISL_636513, EPI_ISL_636514, EPI_ISL_636515, EPI_ISL_636516, EPI_ISL_636517, EPI_ISL_636518, EPI_ISL_636519, EPI_ISL_636520, EPI_ISL_636521, EPI_ISL_636600, EPI_ISL_636601, EPI_ISL_636602, EPI_ISL_636603, EPI_ISL_804385, EPI_ISL_804411, EPI_ISL_804451			
see above	Dutch COVID-19 response team	National Institute for Public Health and the Environment (RIVM)	Adam Meijer; AnneMarie van den Brandt; Bas van der Veer; Chantal Reusken; Dennis Schmitz; Dirk Eggink; Florian Zwagemaker; Harry Vennema; Jeroen Cremer; Matthijs Welkers; Pieter Overduin; Sharon van den Brink; on behalf of the national COVID-19 response team
EPI_ISL_426287	E. Gulbja Laboratorija	Latvian Biomedical Research and Study Centre	Dmitrijs Perminovs; Ivars Silamielis; Jnis Kloviš; Kaspars Megnis; Mikus Gavars; Monta Ustinova; Uga Dumpis; Vita Rovte; ikita Zrelovs
EPI_ISL_434540	EBAIS Concepción Norte	Inciensa, Instituto Costarricense de Investigación y Enseñanza en Nutrición y Salud	Adriana Godinez & Melany Calderon; Claudio Soto-Garita; Estela Cordero; Francisco Duarte; Hebleen Porras
EPI_ISL_593507	Eastern Ontario Regional Laboratory Association	McMaster University	Ahmed Draia; Andrew G. McArthur; Emily Panousis; Hooman Derakhshani; Jalees Nasir; Leanne Mortimer; Robert Slinger
EPI_ISL_754757, EPI_ISL_754765	Emory Molecular Diagnostics Laboratory, Emory Healthcare	Piantadosi Lab, Emory Department of Pathology	Ahmed Babiker; Anne Piantadosi
EPI_ISL_450873, EPI_ISL_450874	Evandro Chagas Institute	Evandro Chagas Institute	A.M.; Barbagelata; E.C.; E.M.A.; Ferreira; G.M.R; J.A.; Junior; L.C.; L.S.; M.C.; Martins; P.S.; Santos; Silva; Sousa; Sousa Junior; Viana; W.D.C.; da Silva
EPI_ISL_445277	FUNDACION DE SALUD EL TENIENTE	Instituto de Salud Publica de Chile	Alejandra Acevedo; Andrés E Castillo; Bárbara Parra; Carolina Tambley; Gabriel Leal; Jaime Lagos; Jorge Fernandez; Loredana Arata; Patricia Bustos; Paz Tapia; Rodrigo Fasce; Winston Andrade
EPI_ISL_468747, EPI_ISL_468748, EPI_ISL_468749, EPI_ISL_468750, EPI_ISL_468751	Facultad de Medicina UC	Center for Mathematical Modeling and Center for Genome Regulation. Santiago, Chile	Allende ML; Ferres M.; Gaete A; González M; Maass A; Palma R; Travisany D; Urra C; Varas M
EPI_ISL_804016, EPI_ISL_804017	Facultad de Medicina, Universidad de Atacama	Facultad de Ciencias de la Vida, UNAB	Claudio Meneses; César Echeverría; Dayán Sanhueza; Eduardo Castro; Jorge Olivares; Macarena Bastías; Sebastián Wolter; Waldo Díaz
EPI_ISL_480858, EPI_ISL_480888, EPI_ISL_495390, EPI_ISL_508771, EPI_ISL_509725, EPI_ISL_509730, EPI_ISL_509740, EPI_ISL_549247, EPI_ISL_568618, EPI_ISL_653101			
see above	Florida Bureau of Public Health Laboratories	Florida Bureau of Public Health Laboratories	Jason Blanton; Sarah Schmedes
EPI_ISL_632222, EPI_ISL_632225	Flushing Hospital Medical Center	New York City Public Health Laboratory	Jade Wang; et al.
EPI_ISL_475541	Follinge Halsocentral	The Public Health Agency of Sweden	Anna Risberg; Anna-Malin Linde; Karin Tegmark-Wisell; Maria Lind Karlberg; Mattias Haukland; Mia Brytting; Olov Svartstrom; Oskar Karlsson Lindsjo; Reza Advani; Sandra Broddesson

EPI_ISL_794649, EPI_ISL_794650	Fundación Cardio Infantil	Instituto Nacional de Salud - Dirección de Investigación en Salud Pública	Carlos Franco-Muñoz; Diego A. Álvarez-Díaz; Diego Andrés Prada; Gerardo Santamaría; Jonathan Reales; Julian Naizaque; Katherine Laiton-Donato; Magdalena Wiesner; Marcela Mercado-Reyes; Maria T. Herrera; Martha Lucia Ospina Martinez; Mauricio Pacheco-Montealegre; Paola Muñoz-Laiton; Sheryl Corchuelo
EPI_ISL_794654	Fundación Valle del Lili	Instituto Nacional de Salud - Dirección de Investigación en Salud Pública	Carlos Franco-Muñoz; Diego A. Álvarez-Díaz; Diego Andrés Prada; Gerardo Santamaría; Jonathan Reales; Julian Naizaque; Katherine Laiton-Donato; Magdalena Wiesner; Marcela Mercado-Reyes; Maria T. Herrera; Martha Lucia Ospina Martinez; Mauricio Pacheco-Montealegre; Paola Muñoz-Laiton; Sheryl Corchuelo
EPI_ISL_447554	GMERS Medical College and Hospital, Gandhinagar	Gujarat Biotechnology Research Centre	Akanksha Verma; Amit Kanani; Ankit Hinsu; Apurvasinh Puvar; Bhavesh Modi; Binita Aring; Chaitanya Joshi; Dinesh Kumar; Dipa Kinariwala; Disha Patel; Gaurishankar Shrimali; Geeta Vaghela; Janvi Raval; Kairavi Joshi; Kamlesh J Upadhyay; Madhvi Joshi; Maharshi Pandya; Monika Gandhi; Neeta Khandelwal; Nidhi Sood; Nitin Savaliya; Pinal Trivedi; Pranay Shah; Pritesh Sabara; R D Dixit; Raghawendra Kumar; Ramesh Pandit; Sharmistha Majumdar; Snehal Bagatharia; Sonia Barve; Tejas Shah; Zuber Saiyed
EPI_ISL_754391, EPI_ISL_754392, EPI_ISL_754393	Genetica Molecular and Subdepartamento de Virologia ISP Chile	Insituto de Salud Publica de Chile	Andres Castillo; Barbara Parra; Gisselle Barra; Jaime Lagos; Javier Tognarelli; Jorge Fernandez; Loredana Arata; Patricia Bustos; Rodrigo Fasce
EPI_ISL_746478, EPI_ISL_746479, EPI_ISL_746480, EPI_ISL_746481, EPI_ISL_746482, EPI_ISL_746483, EPI_ISL_746484, EPI_ISL_746485, EPI_ISL_746486, EPI_ISL_746487, EPI_ISL_746488, EPI_ISL_746489, EPI_ISL_746490, EPI_ISL_746491, EPI_ISL_746492, EPI_ISL_746493, EPI_ISL_746494, EPI_ISL_746495, EPI_ISL_746496, EPI_ISL_746497, EPI_ISL_746498, EPI_ISL_746499, EPI_ISL_746500, EPI_ISL_746501, EPI_ISL_746502, EPI_ISL_746503, EPI_ISL_746504, EPI_ISL_746505, EPI_ISL_746506, EPI_ISL_746507, EPI_ISL_746508, EPI_ISL_746509, EPI_ISL_746510, EPI_ISL_746511, EPI_ISL_746512, EPI_ISL_746513, EPI_ISL_746514, EPI_ISL_746515, EPI_ISL_746516, EPI_ISL_746517, EPI_ISL_746518, EPI_ISL_746519, EPI_ISL_746520, EPI_ISL_746521, EPI_ISL_746522, EPI_ISL_746523, EPI_ISL_746524, EPI_ISL_746525, EPI_ISL_746526, EPI_ISL_746527, EPI_ISL_746528, EPI_ISL_746529, EPI_ISL_746530, EPI_ISL_746531, EPI_ISL_746532, EPI_ISL_746533, EPI_ISL_746534, EPI_ISL_746535, EPI_ISL_746536, EPI_ISL_746537, EPI_ISL_746538, EPI_ISL_746539, EPI_ISL_746540, EPI_ISL_746541, EPI_ISL_746542, EPI_ISL_746543, EPI_ISL_746544, EPI_ISL_746545, EPI_ISL_746546, EPI_ISL_746547, EPI_ISL_746548, EPI_ISL_746549, EPI_ISL_746550, EPI_ISL_746551, EPI_ISL_746552, EPI_ISL_746553, EPI_ISL_746554, EPI_ISL_746555, EPI_ISL_746556, EPI_ISL_746557, EPI_ISL_746558, EPI_ISL_746559, EPI_ISL_746560, EPI_ISL_746561, EPI_ISL_746562, EPI_ISL_746563, EPI_ISL_746564, EPI_ISL_746565, EPI_ISL_746566, EPI_ISL_746567, EPI_ISL_746568, EPI_ISL_746569, EPI_ISL_746570, EPI_ISL_746571, EPI_ISL_746572, EPI_ISL_746573, EPI_ISL_746574, EPI_ISL_746575, EPI_ISL_746576, EPI_ISL_746577, EPI_ISL_746578, EPI_ISL_746579, EPI_ISL_746580, EPI_ISL_746581, EPI_ISL_746582, EPI_ISL_746583, EPI_ISL_746584, EPI_ISL_746585, EPI_ISL_746586, EPI_ISL_746587, EPI_ISL_746588, EPI_ISL_746589, EPI_ISL_746590, EPI_ISL_746591, EPI_ISL_746592, EPI_ISL_746593, EPI_ISL_746594, EPI_ISL_746595, EPI_ISL_746596, EPI_ISL_746597, EPI_ISL_746598, EPI_ISL_746599, EPI_ISL_746600, EPI_ISL_746601, EPI_ISL_746602, EPI_ISL_746603, EPI_ISL_746604, EPI_ISL_746605, EPI_ISL_746606, EPI_ISL_746607, EPI_ISL_746608, EPI_ISL_746609, EPI_ISL_746610, EPI_ISL_746611, EPI_ISL_746612, EPI_ISL_746613, EPI_ISL_746614, EPI_ISL_746615, EPI_ISL_746616, EPI_ISL_746617, EPI_ISL_746618, EPI_ISL_746619, EPI_ISL_746620, EPI_ISL_746621, EPI_ISL_746622, EPI_ISL_746623, EPI_ISL_746624, EPI_ISL_746625, EPI_ISL_746626, EPI_ISL_746627, EPI_ISL_746628, EPI_ISL_746629, EPI_ISL_746630, EPI_ISL_746631, EPI_ISL_746632, EPI_ISL_746633, EPI_ISL_746634, EPI_ISL_746635, EPI_ISL_746636, EPI_ISL_746637, EPI_ISL_746638, EPI_ISL_746639, EPI_ISL_746640, EPI_ISL_746641, EPI_ISL_746642, EPI_ISL_746643, EPI_ISL_746644, EPI_ISL_746645, EPI_ISL_746646, EPI_ISL_746647, EPI_ISL_746648, EPI_ISL_746649, EPI_ISL_746650, EPI_ISL_746651, EPI_ISL_746652, EPI_ISL_746653, EPI_ISL_746654, EPI_ISL_746655, EPI_ISL_746656, EPI_ISL_746657, EPI_ISL_746658, EPI_ISL_746659, EPI_ISL_746660, EPI_ISL_746661, EPI_ISL_746662, EPI_ISL_746663, EPI_ISL_746664, EPI_ISL_746665, EPI_ISL_746666, EPI_ISL_746667, EPI_ISL_746668, EPI_ISL_746669, EPI_ISL_746670, EPI_ISL_746671, EPI_ISL_746672, EPI_ISL_746673, EPI_ISL_746674, EPI_ISL_746675, EPI_ISL_746676, EPI_ISL_746677, EPI_ISL_746678, EPI_ISL_746679, EPI_ISL_746680, EPI_ISL_746681, EPI_ISL_746682, EPI_ISL_746683, EPI_ISL_746684, EPI_ISL_746685, EPI_ISL_746686, EPI_ISL_746687, EPI_ISL_746688, EPI_ISL_746689, EPI_ISL_746690, EPI_ISL_746691, EPI_ISL_746692, EPI_ISL_746693, EPI_ISL_746694, EPI_ISL_746695, EPI_ISL_746696, EPI_ISL_746697, EPI_ISL_746698, EPI_ISL_746699, EPI_ISL_746700, EPI_ISL_746701, EPI_ISL_746702, EPI_ISL_746703, EPI_ISL_746704, EPI_ISL_746705, EPI_ISL_746706, EPI_ISL_746707, EPI_ISL_746708, EPI_ISL_746709, EPI_ISL_746710, EPI_ISL_746711, EPI_ISL_746712, EPI_ISL_746713, EPI_ISL_746714, EPI_ISL_746715, EPI_ISL_746716, EPI_ISL_746717, EPI_ISL_746718, EPI_ISL_746719, EPI_ISL_746720, EPI_ISL_746721, EPI_ISL_746722, EPI_ISL_746723, EPI_ISL_746724, EPI_ISL_746725, EPI_ISL_746726, EPI_ISL_746727, EPI_ISL_746728, EPI_ISL_746729, EPI_ISL_746730, EPI_ISL_746731, EPI_ISL_746732, EPI_ISL_746733, EPI_ISL_746734, EPI_ISL_746735, EPI_ISL_746736, EPI_ISL_746737, EPI_ISL_746738, EPI_ISL_746739, EPI_ISL_746740, EPI_ISL_746741, EPI_ISL_746742, EPI_ISL_746743, EPI_ISL_746744, EPI_ISL_746745, EPI_ISL_746746, EPI_ISL_746747, EPI_ISL_746748, EPI_ISL_746749, EPI_ISL_746750, EPI_ISL_746751, EPI_ISL_746752, EPI_ISL_746753, EPI_ISL_746754, EPI_ISL_746755, EPI_ISL_746756, EPI_ISL_746757, EPI_ISL_746758, EPI_ISL_746759, EPI_ISL_746760, EPI_ISL_746761, EPI_ISL_746762, EPI_ISL_746763, EPI_ISL_746764, EPI_ISL_746765, EPI_ISL_746766, EPI_ISL_746767, EPI_ISL_746768, EPI_ISL_746769, EPI_ISL_746770, EPI_ISL_746771, EPI_ISL_746772, EPI_ISL_746773, EPI_ISL_746774, EPI_ISL_746775, EPI_ISL_746776, EPI_ISL_746777, EPI_ISL_746778, EPI_ISL_746779, EPI_ISL_746780, EPI_ISL_746781, EPI_ISL_746782, EPI_ISL_746783, EPI_ISL_746784, EPI_ISL_746785, EPI_ISL_746786, EPI_ISL_746787, EPI_ISL_746788, EPI_ISL_746789, EPI_ISL_746790, EPI_ISL_746791, EPI_ISL_746792, EPI_ISL_746793, EPI_ISL_746794, EPI_ISL_746795, EPI_ISL_746796, EPI_ISL_746797, EPI_ISL_746798, EPI_ISL_746799, EPI_ISL_746800, EPI_ISL_746801, EPI_ISL_746802, EPI_ISL_746803, EPI_ISL_746804, EPI_ISL_746805, EPI_ISL_746806, EPI_ISL_746807, EPI_ISL_746808, EPI_ISL_746809, EPI_ISL_746810, EPI_ISL_746811, EPI_ISL_746812, EPI_ISL_746813, EPI_ISL_746814, EPI_ISL_746815, EPI_ISL_746816, EPI_ISL_746817, EPI_ISL_746818, EPI_ISL_746819, EPI_ISL_746820, EPI_ISL_746821, EPI_ISL_746822, EPI_ISL_746823, EPI_ISL_746824, EPI_ISL_746825			
see above	Genetica Molecular and Subdepartamento de Virologia ISP Chile	Instituto de Salud Publica de Chile	Andres Castillo; Barbara Parra; Gisselle Barra; Jaime Lagos; Javier Tognarelli; Jorge Fernandez; Loredana Arata; Patricia Bustos; Rodrigo Fasce
EPI_ISL_730197, EPI_ISL_730198, EPI_ISL_730199, EPI_ISL_730200, EPI_ISL_730201, EPI_ISL_730202, EPI_ISL_730203, EPI_ISL_730204, EPI_ISL_730205, EPI_ISL_730206, EPI_ISL_730207, EPI_ISL_730208, EPI_ISL_730209, EPI_ISL_730210, EPI_ISL_730211, EPI_ISL_730212, EPI_ISL_730213, EPI_ISL_730214, EPI_ISL_730215, EPI_ISL_730216, EPI_ISL_730217, EPI_ISL_730218, EPI_ISL_730219, EPI_ISL_730220, EPI_ISL_730221, EPI_ISL_730222, EPI_ISL_730223, EPI_ISL_730224, EPI_ISL_730225, EPI_ISL_730226, EPI_ISL_730227, EPI_ISL_730228			
see above	Genomica Lab Molecular, M©xico	Andersen lab at Scripps Research	Jose Horacio Reyna Verdugo; Jose Roman Chavez Mendez; Luis Alberto Rangel Gonzalez; Martin Gonzalez Ibarra; SEARCH Alliance San Diego with Jonathan Gonzalez Garcia
EPI_ISL_640016	George Hospital wc GRH	NHLS/UCT	Arash Iranzadeh; Bruna Galvao; Carolyn Williamson; Deelan Doolabh; Diana Hardie; Innocent Mudau; Kruger Marais; Lynn Tyers; Marvin Hsiao; Stephen Korsman
EPI_ISL_415152	Gorgas Memorial Institute for Health Studies	Gorgas Memorial Institute for Health Studies	Alexander A. Martinez.; Ambar Moreno; Claudia Gonzalez; Danilo Franco; Elimelec Valdespino; Juan M. Pascale; Leyda Abrego; Oris Chavarria; Sandra Lopez-Verges; Yamilka Diaz
EPI_ISL_496604, EPI_ISL_496605, EPI_ISL_496606, EPI_ISL_496607, EPI_ISL_496608, EPI_ISL_496609, EPI_ISL_496610, EPI_ISL_496611, EPI_ISL_496612, EPI_ISL_496613, EPI_ISL_496614, EPI_ISL_496615, EPI_ISL_496616, EPI_ISL_496617, EPI_ISL_496618, EPI_ISL_496619, EPI_ISL_496621, EPI_ISL_496624, EPI_ISL_496625, EPI_ISL_496626, EPI_ISL_496627, EPI_ISL_496629, EPI_ISL_496630, EPI_ISL_496631, EPI_ISL_496632, EPI_ISL_496633, EPI_ISL_496634, EPI_ISL_496636, EPI_ISL_496637, EPI_ISL_496638, EPI_ISL_496640, EPI_ISL_496641, EPI_ISL_496642, EPI_ISL_496643, EPI_ISL_496644, EPI_ISL_496645, EPI_ISL_496646, EPI_ISL_496647, EPI_ISL_496648, EPI_ISL_496650, EPI_ISL_496651, EPI_ISL_496652, EPI_ISL_496653, EPI_ISL_496654, EPI_ISL_496655, EPI_ISL_496657, EPI_ISL_496658, EPI_ISL_496659, EPI_ISL_496660, EPI_ISL_496663, EPI_ISL_496664, EPI_ISL_496665, EPI_ISL_496666, EPI_ISL_496669, EPI_ISL_496670, EPI_ISL_496671, EPI_ISL_496675, EPI_ISL_496676, EPI_ISL_496678, EPI_ISL_496679, EPI_ISL_496680, EPI_ISL_496681, EPI_ISL_496682, EPI_ISL_496683, EPI_ISL_496684, EPI_ISL_496687, EPI_ISL_496688, EPI_ISL_496689, EPI_ISL_496692, EPI_ISL_496693, EPI_ISL_496694, EPI_ISL_496695, EPI_ISL_496699, EPI_ISL_496700, EPI_ISL_496701, EPI_ISL_496702, EPI_ISL_496703, EPI_ISL_496705, EPI_ISL_496706, EPI_ISL_496708, EPI_ISL_496709, EPI_ISL_496710, EPI_ISL_496711, EPI_ISL_496712, EPI_ISL_496713, EPI_ISL_496714, EPI_ISL_496715, EPI_ISL_496716, EPI_ISL_496718, EPI_ISL_496719, EPI_ISL_496721, EPI_ISL_496722, EPI_ISL_496723, EPI_ISL_496724, EPI_ISL_496725, EPI_ISL_496726, EPI_ISL_496727, EPI_ISL_496728, EPI_ISL_496730, EPI_ISL_496731, EPI_ISL_496732, EPI_ISL_496733, EPI_ISL_496734, EPI_ISL_496735, EPI_ISL_496736, EPI_ISL_496737, EPI_ISL_496740, EPI_ISL_496741, EPI_ISL_496742, EPI_ISL_496743, EPI_ISL_496744, EPI_ISL_496745, EPI_ISL_496746, EPI_ISL_496748, EPI_ISL_496749, EPI_ISL_496750, EPI_ISL_496751, EPI_ISL_496753, EPI_ISL_496756, EPI_ISL_496762, EPI_ISL_496763, EPI_ISL_496764, EPI_ISL_496766, EPI_ISL_496767, EPI_ISL_496768, EPI_ISL_496770, EPI_ISL_496771, EPI_ISL_496772, EPI_ISL_496774, EPI_ISL_496775, EPI_ISL_496776, EPI_ISL_496780, EPI_ISL_496781, EPI_ISL_496784, EPI_ISL_496785, EPI_ISL_496786, EPI_ISL_496787, EPI_ISL_496790, EPI_ISL_496792, EPI_ISL_496793, EPI_ISL_496795, EPI_ISL_496798, EPI_ISL_496799, EPI_ISL_496800, EPI_ISL_496801, EPI_ISL_496802, EPI_ISL_496803, EPI_ISL_496804, EPI_ISL_496805, EPI_ISL_496806, EPI_ISL_496809, EPI_ISL_496815, EPI_ISL_496817, EPI_ISL_496819, EPI_ISL_496822, EPI_ISL_496823, EPI_ISL_496824, EPI_ISL_496825, EPI_ISL_496826, EPI_ISL_496827, EPI_ISL_496828, EPI_ISL_496829, EPI_ISL_496830, EPI_ISL_496832, EPI_ISL_496833, EPI_ISL_496834, EPI_ISL_496835, EPI_ISL_496837, EPI_ISL_496840, EPI_ISL_496841, EPI_ISL_496842, EPI_ISL_496843, EPI_ISL_496844, EPI_ISL_496846, EPI_ISL_496847, EPI_ISL_496848, EPI_ISL_496850, EPI_ISL_496851, EPI_ISL_496852, EPI_ISL_496853, EPI_ISL_496854, EPI_ISL_496855, EPI_ISL_496856, EPI_ISL_496857, EPI_ISL_496859, EPI_ISL_496860, EPI_ISL_496861, EPI_ISL_496862, EPI_ISL_496863, EPI_ISL_496865, EPI_ISL_496867, EPI_ISL_496868, EPI_ISL_496870, EPI_ISL_496871, EPI_ISL_496873, EPI_ISL_496874, EPI_ISL_496875, EPI_ISL_496877, EPI_ISL_496878, EPI_ISL_496879, EPI_ISL_496880, EPI_ISL_496881, EPI_ISL_496884, EPI_ISL_496885, EPI_ISL_496886, EPI_ISL_496887, EPI_ISL_496888, EPI_ISL_496889, EPI_ISL_496891, EPI_ISL_496892, EPI_ISL_496893, EPI_ISL_496894, EPI_ISL_496895, EPI_ISL_496896, EPI_ISL_496897, EPI_ISL_496898, EPI_ISL_496900, EPI_ISL_496901, EPI_ISL_496904, EPI_ISL_496906, EPI_ISL_496907, EPI_ISL_496908, EPI_ISL_496909, EPI_ISL_496911, EPI_ISL_496914, EPI_ISL_496915			
see above	Gorgas Memorial Laboratory of Health Studies	Gorgas Memorial Laboratory of Health Studies	Alexander A Martinez; Claudia Gonzalez Sandra Lopez-Verges; Danilo Franco
EPI_ISL_469031	Government Medical College, Vadodara	Gujarat Biotechnology Research Centre	A M Kadri; Ankit Hinsu; Apurvasinh Puvar; Bhavya Jindal; Chaitanya Joshi; Dinesh Kumar; Harsh Bakshi; Janvi Raval; Komal Patel; Labdhi Pandya; Madhvi Joshi; Maharshi Pandya; Meenakshi Shah; Monika Gandhi; Neena Doshi; Nidhi Patel; Nitin Savaliya; Pinal Trivedi; Pritesh Sabara; R D Dixit; Raghawendra Kumar; Snehal Bagatharia; Tejas Shah; Varsha Godbole; Zarna Patel; Zuber Saiyed
EPI_ISL_698232, EPI_ISL_698314, EPI_ISL_698548	Group 42 (G42) Healthcare, Abu Dhabi, United Arab Emirates; Department of Health, The United Arab Emirates	G42 Healthcare	Ashish Koshy; Budoor Alqarni; Denghui Liu; Fang Chen; Hanif Khalak; Huanming Yang; Javier Quilez; Jian Wang; Junhua Li; Ke Liang; Long Lin; Mohammed Saifuddin Fasihuddin; Nan Qiao; Nawal Ahmed Mohamed Al Kaabi; Pauline Ogrodzki; Pei Wu; Peng Xiao; Pengjuan Liu; Rong Liu; Sally Mahmoud; Siyang Liu; Stephen S. Francis; Tao Ma; Vinay Kusuma; Walid Abbas Zaher; Weibin Liu; Wenjun He; Xavier Anton; Xin Jin; Xin Meng; Xinyu Huang; Xun Xu; Zhaorong Yuan
EPI_ISL_447734, EPI_ISL_447735, EPI_ISL_447736, EPI_ISL_447737, EPI_ISL_447738, EPI_ISL_447739, EPI_ISL_447740, EPI_ISL_447741, EPI_ISL_447742, EPI_ISL_447743, EPI_ISL_447744, EPI_ISL_447745, EPI_ISL_447746, EPI_ISL_447747, EPI_ISL_447748, EPI_ISL_447749, EPI_ISL_447750, EPI_ISL_447752, EPI_ISL_447753, EPI_ISL_447754			
see above	Grupo de Investigaciones Microbiológicas-UR (GIMUR), Departamento de Biología, Facultad de Ciencias Naturales, Universidad del Rosario, Bogotá, Colombia	Grupo de Investigaciones Microbiológicas-UR (GIMUR), Departamento de Biología, Facultad de Ciencias Naturales, Universidad del Rosario, Bogotá, Colombia Instituto Nacional de Salud, Bogotá, Colombia Icahn School of Medicine at Mount Sinai, New York, USA	Adriana Castillo; Alberto Paniz-Mondolfi; Ana S. Gonzalez-Reiche; Angelica Rico; Aníbal A. Teherán; Carolina Florez; Carolina Hernandez; David Martínez; Emilia Mia Sordillo; Esther C. Barros; Harm van Bakel; Jesús E. Jaimes; Juan David Ramírez; Laura Vega; Lisseth Pardo; Marina Muñoz; Martha L. Ospina; Matthew M. Hernandez; Nathalia Ballesteros; Sergio Castañeda; Sergio Gomez; Viviana Simon
EPI_ISL_509695, EPI_ISL_509696, EPI_ISL_509697, EPI_ISL_509698, EPI_ISL_509699, EPI_ISL_509700, EPI_ISL_509701, EPI_ISL_509702, EPI_ISL_509703, EPI_ISL_509710			
see above	Guatemala Ministry of Public Health	Pathogen Discovery, Respiratory Viruses Branch, Division of Viral Diseases, Centers for Disease Control and Prevention	Anna Uehara; Clinton Paden; Haibin Wang; Jing Zhang; Krista Queen; Suxiang Tong; Yan Li; Ying Tao
EPI_ISL_700512	Guguletu CHC wc GDH	NHLS/UCT	Arash Iranzadeh; Bruna Galvao; Carolyn Williamson; Deelan Doolabh; Diana Hardie; Innocent Mudau; Kruger Marais; Lynn Tyers; Marvin Hsiao; Stephen Korsman
EPI_ISL_445359	HOSP. SANTIAGO ORIENTE DR. LUIS TISNE B.	Instituto de Salud Publica de Chile	Alejandra Acevedo; Andrés E Castillo; Bárbara Parra; Carolina Tambley; Gabriel Leal; Jaime Lagos; Jorge Fernandez; Loredana Arata; Patricia Bustos; Paz Tapia; Rodrigo Fasce; Winston Andrade

EPI_ISL_445323	HOSP.ENFERMEDADES INFECCIOSAS	Instituto de Salud Publica de Chile	Alejandra Acevedo; Andrés E Castillo; Bárbara Parra; Carolina Tambley; Gabriel Leal; Jaime Lagos; Jorge Fernandez; Loredana Arata; Patricia Bustos; Paz Tapia; Rodrigo Fasce; Winston Andrade
EPI_ISL_682239	HOSPITAL CIUDAD NEILY	Inciensa, Instituto Costarricense de Investigación y Enseñanza en Nutrición y Salud	Adriana Godinez & Melany Calderon; Claudio Soto-Garita; Estela Cordero; Francisco Duarte; Hebleen Porras
EPI_ISL_445281	HOSPITAL CLINICO DEL SUR	Instituto de Salud Publica de Chile	Alejandra Acevedo; Andrés E Castillo; Bárbara Parra; Carolina Tambley; Gabriel Leal; Jaime Lagos; Jorge Fernandez; Loredana Arata; Patricia Bustos; Paz Tapia; Rodrigo Fasce; Winston Andrade
EPI_ISL_445275, EPI_ISL_445276	HOSPITAL CLINICO FUSAT	Instituto de Salud Publica de Chile	Alejandra Acevedo; Andrés E Castillo; Bárbara Parra; Carolina Tambley; Gabriel Leal; Jaime Lagos; Jorge Fernandez; Loredana Arata; Patricia Bustos; Paz Tapia; Rodrigo Fasce; Winston Andrade
EPI_ISL_445378	HOSPITAL DE BULNES	Instituto de Salud Publica de Chile	Alejandra Acevedo; Andrés E Castillo; Bárbara Parra; Carolina Tambley; Gabriel Leal; Jaime Lagos; Jorge Fernandez; Loredana Arata; Patricia Bustos; Paz Tapia; Rodrigo Fasce; Winston Andrade
EPI_ISL_445335	HOSPITAL DE CALBUCO	Instituto de Salud Publica de Chile	Alejandra Acevedo; Andrés E Castillo; Bárbara Parra; Carolina Tambley; Gabriel Leal; Jaime Lagos; Jorge Fernandez; Loredana Arata; Patricia Bustos; Paz Tapia; Rodrigo Fasce; Winston Andrade
EPI_ISL_445251, EPI_ISL_445305, EPI_ISL_445354, EPI_ISL_445369, EPI_ISL_445370	HOSPITAL DE CARABINEROS	Instituto de Salud Publica de Chile	Alejandra Acevedo; Andrés E Castillo; Bárbara Parra; Carolina Tambley; Gabriel Leal; Jaime Lagos; Jorge Fernandez; Loredana Arata; Patricia Bustos; Paz Tapia; Rodrigo Fasce; Winston Andrade
EPI_ISL_445300	HOSPITAL DE RANCAGUA	Instituto de Salud Publica de Chile	Alejandra Acevedo; Andrés E Castillo; Bárbara Parra; Carolina Tambley; Gabriel Leal; Jaime Lagos; Jorge Fernandez; Loredana Arata; Patricia Bustos; Paz Tapia; Rodrigo Fasce; Winston Andrade
EPI_ISL_445298	HOSPITAL DE SAN FERNANDO	Instituto de Salud Publica de Chile	Alejandra Acevedo; Andrés E Castillo; Bárbara Parra; Carolina Tambley; Gabriel Leal; Jaime Lagos; Jorge Fernandez; Loredana Arata; Patricia Bustos; Paz Tapia; Rodrigo Fasce; Winston Andrade
EPI_ISL_445352, EPI_ISL_445360, EPI_ISL_445368	HOSPITAL DEL PROFESOR	Instituto de Salud Publica de Chile	Alejandra Acevedo; Andrés E Castillo; Bárbara Parra; Carolina Tambley; Gabriel Leal; Jaime Lagos; Jorge Fernandez; Loredana Arata; Patricia Bustos; Paz Tapia; Rodrigo Fasce; Winston Andrade
EPI_ISL_445270, EPI_ISL_445338, EPI_ISL_445339, EPI_ISL_445340, EPI_ISL_445341, EPI_ISL_445342, EPI_ISL_445343, EPI_ISL_445344, EPI_ISL_445345, EPI_ISL_445346, EPI_ISL_445347, EPI_ISL_445348, EPI_ISL_447119			
see above	HOSPITAL DR.HERNAN HENRIQUEZ ARAVENA	Instituto de Salud Publica de Chile	Alejandra Acevedo; Andrés E Castillo; Bárbara Parra; Carolina Tambley; Gabriel Leal; Jaime Lagos; Jorge Fernandez; Loredana Arata; Patricia Bustos; Paz Tapia; Rodrigo Fasce; Winston Andrade
EPI_ISL_445310, EPI_ISL_445325, EPI_ISL_445329, EPI_ISL_445365, EPI_ISL_445366, EPI_ISL_445371	HOSPITAL DR.SOTERO DEL RIO	Instituto de Salud Publica de Chile	Alejandra Acevedo; Andrés E Castillo; Bárbara Parra; Carolina Tambley; Gabriel Leal; Jaime Lagos; Jorge Fernandez; Loredana Arata; Patricia Bustos; Paz Tapia; Rodrigo Fasce; Winston Andrade
EPI_ISL_445308, EPI_ISL_445313, EPI_ISL_445314, EPI_ISL_445364	HOSPITAL EL CARMEN DR.LUIS VALENTIN F.	Instituto de Salud Publica de Chile	Alejandra Acevedo; Andrés E Castillo; Bárbara Parra; Carolina Tambley; Gabriel Leal; Jaime Lagos; Jorge Fernandez; Loredana Arata; Patricia Bustos; Paz Tapia; Rodrigo Fasce; Winston Andrade
EPI_ISL_445319, EPI_ISL_445321	HOSPITAL FELIX BULNES	Instituto de Salud Publica de Chile	Alejandra Acevedo; Andrés E Castillo; Bárbara Parra; Carolina Tambley; Gabriel Leal; Jaime Lagos; Jorge Fernandez; Loredana Arata; Patricia Bustos; Paz Tapia; Rodrigo Fasce; Winston Andrade
EPI_ISL_445372	HOSPITAL FF.AA. "CIRUJANO C. GUZMAN	Instituto de Salud Publica de Chile	Alejandra Acevedo; Andrés E Castillo; Bárbara Parra; Carolina Tambley; Gabriel Leal; Jaime Lagos; Jorge Fernandez; Loredana Arata; Patricia Bustos; Paz Tapia; Rodrigo Fasce; Winston Andrade
EPI_ISL_445286, EPI_ISL_445337	HOSPITAL HANGA ROA	Instituto de Salud Publica de Chile	Alejandra Acevedo; Andrés E Castillo; Bárbara Parra; Carolina Tambley; Gabriel Leal; Jaime Lagos; Jorge Fernandez; Loredana Arata; Patricia Bustos; Paz Tapia; Rodrigo Fasce; Winston Andrade
EPI_ISL_445331, EPI_ISL_445332	HOSPITAL HERMINDA MARTIN CHILLAN	Instituto de Salud Publica de Chile	Alejandra Acevedo; Andrés E Castillo; Bárbara Parra; Carolina Tambley; Gabriel Leal; Jaime Lagos; Jorge Fernandez; Loredana Arata; Patricia Bustos; Paz Tapia; Rodrigo Fasce; Winston Andrade
EPI_ISL_445336	HOSPITAL LAS HIGUERAS DE TALCAHUANO	Instituto de Salud Publica de Chile	Alejandra Acevedo; Andrés E Castillo; Bárbara Parra; Carolina Tambley; Gabriel Leal; Jaime Lagos; Jorge Fernandez; Loredana Arata; Patricia Bustos; Paz Tapia; Rodrigo Fasce; Winston Andrade
EPI_ISL_445289, EPI_ISL_445301	HOSPITAL NAVAL PUERTO WILLIAMS	Instituto de Salud Publica de Chile	Alejandra Acevedo; Andrés E Castillo; Bárbara Parra; Carolina Tambley; Gabriel Leal; Jaime Lagos; Jorge Fernandez; Loredana Arata; Patricia Bustos; Paz Tapia; Rodrigo Fasce; Winston Andrade
EPI_ISL_445327, EPI_ISL_445353	HOSPITAL PADRE HURTADO	Instituto de Salud Publica de Chile	Alejandra Acevedo; Andrés E Castillo; Bárbara Parra; Carolina Tambley; Gabriel Leal; Jaime Lagos; Jorge Fernandez; Loredana Arata; Patricia Bustos; Paz Tapia; Rodrigo Fasce; Winston Andrade
EPI_ISL_445246	HOSPITAL PUERTO MONTT	Instituto de Salud Publica de Chile	Alejandra Acevedo; Andrés E Castillo; Bárbara Parra; Carolina Tambley; Gabriel Leal; Jaime Lagos; Jorge Fernandez; Loredana Arata; Patricia Bustos; Paz Tapia; Rodrigo Fasce; Winston Andrade
EPI_ISL_445268, EPI_ISL_445269, EPI_ISL_445280, EPI_ISL_445284, EPI_ISL_445288, EPI_ISL_445293, EPI_ISL_445294, EPI_ISL_445295			
see above	HOSPITAL REG.LAUTARO NAVARRO AVARIA	Instituto de Salud Publica de Chile	Alejandra Acevedo; Andrés E Castillo; Bárbara Parra; Carolina Tambley; Gabriel Leal; Jaime Lagos; Jorge Fernandez; Loredana Arata; Patricia Bustos; Paz Tapia; Rodrigo Fasce; Winston Andrade
EPI_ISL_445296	HOSPITAL REGIONAL DE COYHAIQUE	Instituto de Salud Publica de Chile	Alejandra Acevedo; Andrés E Castillo; Bárbara Parra; Carolina Tambley; Gabriel Leal; Jaime Lagos; Jorge Fernandez; Loredana Arata; Patricia Bustos; Paz Tapia; Rodrigo Fasce; Winston Andrade
EPI_ISL_593772, EPI_ISL_593773, EPI_ISL_593774, EPI_ISL_593775, EPI_ISL_593776	HOSPITAL REGIONAL LAMBAYEQUE	GENOMA MAYOR	Franklin R. Aguilar-Gamboa; Heber Silva-Díaz; Juan J. Bonifacio-Briceño; Luis M. López-Serquén; Luis Salcedo-Mejía; Marco E. Mechán-Llontop and Juan P. Cárdenas.; Percy O. Tullume-Vergara; Ramsés Salas-Asencios
EPI_ISL_445307	HOSPITAL SAN JOSE DE MAIPO	Instituto de Salud Publica de Chile	Alejandra Acevedo; Andrés E Castillo; Bárbara Parra; Carolina Tambley; Gabriel Leal; Jaime Lagos; Jorge Fernandez; Loredana Arata; Patricia Bustos; Paz Tapia; Rodrigo Fasce; Winston Andrade
EPI_ISL_682241, EPI_ISL_682243, EPI_ISL_682245, EPI_ISL_682246, EPI_ISL_682253, EPI_ISL_682254, EPI_ISL_682257, EPI_ISL_682262, EPI_ISL_682263, EPI_ISL_682264, EPI_ISL_682266, EPI_ISL_682267, EPI_ISL_682268, EPI_ISL_682269, EPI_ISL_682270, EPI_ISL_682271, EPI_ISL_682272, EPI_ISL_682273, EPI_ISL_682274			
see above	HOSPITAL SAN JUAN DE DIOS	Inciensa, Instituto Costarricense de Investigación y Enseñanza en Nutrición y Salud	Adriana Godinez & Melany Calderon; Claudio Soto-Garita; Estela Cordero; Francisco Duarte; Hebleen Porras
EPI_ISL_445304, EPI_ISL_445349, EPI_ISL_445350, EPI_ISL_445351, EPI_ISL_445373, EPI_ISL_445374, EPI_ISL_445375, EPI_ISL_445376, EPI_ISL_445377, EPI_ISL_449800			
see above	HOSPITAL SAN JUAN DE DIOS	Instituto de Salud Publica de Chile	Alejandra Acevedo; Andrés E Castillo; Bárbara Parra; Carolina Tambley; Gabriel Leal; Jaime Lagos; Jorge Fernandez; Loredana Arata; Patricia Bustos; Paz Tapia; Rodrigo Fasce; Winston Andrade
EPI_ISL_682242, EPI_ISL_682255	HOSPITAL SAN VICENTE DE PAUL	Inciensa, Instituto Costarricense de Investigación y Enseñanza en Nutrición y Salud	Adriana Godinez; Adriana Godinez & Melany Calderon; Claudio Soto-Garita; Estela Cordero; Francisco Duarte; Hebleen Porras; Melany Calderon & Mariel López
EPI_ISL_794659, EPI_ISL_794660	HOSPITAL UNIVERSITARIO SAN IGNACIO	Instituto Nacional de Salud - Dirección de Investigación en Salud Pública	Carlos Franco-Muñoz; Diego A. Álvarez-Díaz; Diego Andrés Prada; Gerardo Santamaría; Jonathan Reales; Julian Naizaque; Katherine Laiton-Donato; Magdalena Wiesner; Marcela Mercado-Reyes; Maria T. Herrera; Martha Lucia Ospina Martinez; Mauricio Pacheco-Montealegre; Paola Muñoz-Laiton; Sheryl Corchuelo
EPI_ISL_682265	HOSPITAL UPALA	Inciensa, Instituto Costarricense de Investigación y Enseñanza en Nutrición y Salud	Adriana Godinez & Melany Calderon; Claudio Soto-Garita; Estela Cordero; Francisco Duarte; Hebleen Porras
EPI_ISL_482581	Hangzhou Center for Diseases Control and Prevention	Hangzhou Center for Diseases Control and Prevention	Haoqiu Wang; Hua Yu; Jun Li; Junfang Chen; Lingfeng Mao; Shuchang Chen; Xin Qian; Xinfen Yu; Xuchu Wang; Zhou Sun

EPI_ISL_700480	Hanover Park CHC wc HPH	NHLS/UCT	Arash Iranzadeh; Bruna Galvao; Carolyn Williamson; Deelan Doolabh; Diana Hardie; Innocent Mudau; Kruger Marais; Lynn Tyers; Marvin Hsiao; Stephen Korsman
EPI_ISL_700499	Heideveld CDC wc HVP	NHLS/UCT	Arash Iranzadeh; Bruna Galvao; Carolyn Williamson; Deelan Doolabh; Diana Hardie; Innocent Mudau; Kruger Marais; Lynn Tyers; Marvin Hsiao; Stephen Korsman
EPI_ISL_451649	Hematology Laboratory, Section of Molecular Diagnostics, University Clinical Centre, Medical University of Gdansk	Laboratory of Recombinant Vaccines	Adam Sodol; Aneta Szulc; Boguslaw Szewczyk; Ewa Milosz; Krystyna Bienkowska-Szewczyk; Krzysztof Lewandowski; Lukasz Rabalski; Marlena Robakowska
EPI_ISL_699708, EPI_ISL_699715, EPI_ISL_699721, EPI_ISL_699908	Hematopathology Laboratory, ACTREC, TMC	Hematopathology Laboratory, ACTREC, TMC	ACTREC; Hematopathology Laboratory
EPI_ISL_470568, EPI_ISL_470569, EPI_ISL_470570, EPI_ISL_470571, EPI_ISL_470573, EPI_ISL_470574, EPI_ISL_470575, EPI_ISL_470576, EPI_ISL_470577, EPI_ISL_470578, EPI_ISL_470579, EPI_ISL_470580, EPI_ISL_470581, EPI_ISL_470582, EPI_ISL_470583, EPI_ISL_470584, EPI_ISL_470585, EPI_ISL_470586, EPI_ISL_470587, EPI_ISL_470588, EPI_ISL_470600, EPI_ISL_470601, EPI_ISL_470602, EPI_ISL_470603, EPI_ISL_470604, EPI_ISL_470605, EPI_ISL_470606, EPI_ISL_470608, EPI_ISL_470609, EPI_ISL_470610, EPI_ISL_470611, EPI_ISL_470613, EPI_ISL_470651, EPI_ISL_470652, EPI_ISL_470653, EPI_ISL_470654, EPI_ISL_470655			
see above	Hermes Pardini	Bioinformatics Laboratory / LNCC	Alexandra Gerber; Amilcar Tanuri; Ana Paula Guimarães; CADDE-group; Carolina Voloch; Ester Cerdeira Sabino; Filipe Romero; Ingra Morales Claro; Jaqueline Goes de Jesus; Laboratorio Hermes Pardini; Laboratorio Simile; Luiz Gonzaga Paula de Almeida; Mariane Talon; Nuno Rodrigues Faria; Renato Santana Aguiar e Ana Tereza Vasconcelos; Ronaldo da Silva Francisco Junior; Terezinha Marta Pereira; working group UFMG; working group UFRJ; Átila Duque Rossi
EPI_ISL_770013	Hle - Asilos De Ancianos	Inciensa, Instituto Costarricense de Investigación y Enseñanza en Nutrición y Salud	Adriana Godínez; Claudio Soto-Garita; Estela Cordero; Francisco Duarte; Hebleen Porras; Melany Calderón & Mariel López
EPI_ISL_770006	Hle - Asociacion Hogar De Ancianos De Palmar Sur De Osa	Inciensa, Instituto Costarricense de Investigación y Enseñanza en Nutrición y Salud	Adriana Godínez; Claudio Soto-Garita; Estela Cordero; Francisco Duarte; Hebleen Porras; Melany Calderón & Mariel López
EPI_ISL_770024	Hle - Asociacion Hogar De Ancianos Santiago Crespo Calvo	Inciensa, Instituto Costarricense de Investigación y Enseñanza en Nutrición y Salud	Adriana Godínez; Claudio Soto-Garita; Estela Cordero; Francisco Duarte; Hebleen Porras; Melany Calderón & Mariel López
EPI_ISL_770031, EPI_ISL_770032	Hle-Asociacion De Atencion Integral Del Anciano San Cayetano	Inciensa, Instituto Costarricense de Investigación y Enseñanza en Nutrición y Salud	Adriana Godínez; Claudio Soto-Garita; Estela Cordero; Francisco Duarte; Hebleen Porras; Melany Calderón & Mariel López
EPI_ISL_471562, EPI_ISL_471581, EPI_ISL_471582, EPI_ISL_515564, EPI_ISL_523977	Hosp. Municipal Prof. Dr. Alípio Corrêa Netto	Instituto Adolfo Lutz, Interdiciplinary Procedures Center, Strategic Laboratory	Claudia Regina Gonçalves; Claudio Tavares Sacchi; Erica Valessa Ramos Gomes
EPI_ISL_560622	Hospital	National Reference Center for Viruses of Respiratory Infections, Institut Pasteur, Paris	Etienne Simon-Lorière; Fabiana Gambaro; Maud Vanpeene; Sylvie Behillil; Sylvie van der Werf; Vincent Enouf
EPI_ISL_693229	Hospital 8 de Maio	Instituto Adolfo Lutz, Interdiciplinary Procedures Center, Strategic Laboratory	Claudia Regina Gonçalves; Claudio Tavares Sacchi; Erica Valessa Ramos Gomes; Karoline Rodrigues Campos
EPI_ISL_471554	Hospital Bosque da Saúde	Instituto Adolfo Lutz, Interdiciplinary Procedures Center, Strategic Laboratory	Claudia Regina Gonçalves; Claudio Tavares Sacchi; Erica Valessa Ramos Gomes
EPI_ISL_491439	Hospital Calderon Guardia	Inciensa, Instituto Costarricense de Investigación y Enseñanza en Nutrición y Salud	Adriana Godinez & Melany Calderon; Claudio Soto-Garita; Estela Cordero; Francisco Duarte; Hebleen Brenes
EPI_ISL_574580	Hospital Cidade Tiradentes Carmen Prudente	Instituto Adolfo Lutz, Interdiciplinary Procedures Center, Strategic Laboratory	Claudia Regina Gonçalves; Claudio Tavares Sacchi; Erica Valessa Ramos Gomes; Karoline Rodrigues Campos
EPI_ISL_527743, EPI_ISL_527744	Hospital Cima	Inciensa, Instituto Costarricense de Investigación y Enseñanza en Nutrición y Salud	Adriana Godinez & Melany Calderon; Claudio Soto-Garita; Estela Cordero; Francisco Duarte; Hebleen Porras
EPI_ISL_491441, EPI_ISL_491442, EPI_ISL_491455	Hospital Clinica Biblica	Inciensa, Instituto Costarricense de Investigación y Enseñanza en Nutrición y Salud	Adriana Godinez & Melany Calderon; Claudio Soto-Garita; Estela Cordero; Francisco Duarte; Hebleen Brenes
EPI_ISL_480315, EPI_ISL_480316, EPI_ISL_480317, EPI_ISL_480319	Hospital Clínica Bíblica	Charité Virology-University of Costa Rica	Andres Moreira-Soto; Eugenia Corrales-Aguilar; Ignacio Postigo-Hidalgo; Jan Felix Drexler; Karla Sofía Gutiérrez
EPI_ISL_512665	Hospital De Las Mujeres Dr. Adolfo Carit	Inciensa, Instituto Costarricense de Investigación y Enseñanza en Nutrición y Salud	Adriana Godinez & Melany Calderon; Claudio Soto-Garita; Estela Cordero; Francisco Duarte; Hebleen Porras
EPI_ISL_512655, EPI_ISL_512672, EPI_ISL_512673, EPI_ISL_512674, EPI_ISL_512675, EPI_ISL_527750	Hospital De Niños Dr. Carlos Saenz Herrera [San Jose/San Jose]	Inciensa, Instituto Costarricense de Investigación y Enseñanza en Nutrición y Salud	Adriana Godinez & Melany Calderon; Claudio Soto-Garita; Estela Cordero; Francisco Duarte; Hebleen Porras
EPI_ISL_574591, EPI_ISL_574592, EPI_ISL_603030, EPI_ISL_693218	Hospital Domingos Leonardo Ceravolo Presidente Prudente	Instituto Adolfo Lutz, Interdiciplinary Procedures Center, Strategic Laboratory	Claudia Regina Gonçalves; Claudio Tavares Sacchi; Erica Valessa Ramos Gomes; Karoline Rodrigues Campos
EPI_ISL_547578	Hospital Doutor Domingos Leonardo Cerávolo	Instituto Adolfo Lutz, Interdiciplinary Procedures Center, Strategic Laboratory	Claudia Regina Gonçalves; Claudio Tavares Sacchi; Erica Valessa Ramos Gomes; Karoline Rodrigues Campos
EPI_ISL_527752, EPI_ISL_769990	Hospital Dr. Rafael A. Calderon Guardia	Inciensa, Instituto Costarricense de Investigación y Enseñanza en Nutrición y Salud	Adriana Godinez & Melany Calderon; Adriana Godínez; Claudio Soto-Garita; Estela Cordero; Francisco Duarte; Hebleen Porras; Melany Calderón & Mariel López
EPI_ISL_512654	Hospital Dr. Rafael A. Calderon Guardia [San Jose/San Jose]	Inciensa, Instituto Costarricense de Investigación y Enseñanza en Nutrición y Salud	Adriana Godinez & Melany Calderon; Claudio Soto-Garita; Estela Cordero; Francisco Duarte; Hebleen Porras
EPI_ISL_770028	Hospital Dr. Raul Blanco Cervantes	Inciensa, Instituto Costarricense de Investigación y Enseñanza en Nutrición y Salud	Adriana Godínez; Claudio Soto-Garita; Estela Cordero; Francisco Duarte; Hebleen Porras; Melany Calderón & Mariel López
EPI_ISL_693213	Hospital E Maternidade Municipal Governador Mario Covas	Instituto Adolfo Lutz, Interdiciplinary Procedures Center, Strategic Laboratory	Claudia Regina Gonçalves; Claudio Tavares Sacchi; Erica Valessa Ramos Gomes; Karoline Rodrigues Campos
EPI_ISL_574594	Hospital Escola da Universidade de Taubate	Instituto Adolfo Lutz, Interdiciplinary Procedures Center, Strategic Laboratory	Claudia Regina Gonçalves; Claudio Tavares Sacchi; Erica Valessa Ramos Gomes; Karoline Rodrigues Campos
EPI_ISL_574588, EPI_ISL_583490	Hospital Estadual Sumare	Instituto Adolfo Lutz, Interdiciplinary Procedures Center, Strategic Laboratory	Claudia Regina Gonçalves; Claudio Tavares Sacchi; Erica Valessa Ramos Gomes; Karoline Rodrigues Campos
EPI_ISL_583501	Hospital Estadual de CampanhaCOVID 19 Barradas	Instituto Adolfo Lutz, Interdiciplinary Procedures Center, Strategic Laboratory	Claudia Regina Gonçalves; Claudio Tavares Sacchi; Erica Valessa Ramos Gomes; Karoline Rodrigues Campos
EPI_ISL_491443, EPI_ISL_491447	Hospital Fernando Escalante Pradilla	Inciensa, Instituto Costarricense de Investigación y Enseñanza en Nutrición y Salud	Adriana Godinez & Melany Calderon; Claudio Soto-Garita; Estela Cordero; Francisco Duarte; Hebleen Brenes
EPI_ISL_476496, EPI_ISL_476561	Hospital Garrahan	Héritas	Andrea Mangano; Cristian Rohr; Dalmacio Pereyra; Fabian Fay; Maria Florencia Fernandez; Martin Vazquez; Mauricio Grisolia; Roberta Crespo
EPI_ISL_467224	Hospital General Universitario Gregorio Marañón	SeqCOVID-SPAIN consortium/IBV(CSIC)	Darío García de Viedma and SeqCOVID-SPAIN consortium; Jon Sicilia; Julia Suárez; Laura Pérez-Lago; Marta Herranz; Patricia Muñoz; Pilar Catalán
EPI_ISL_471541, EPI_ISL_523971	Hospital Geral Santa Marcelina	Instituto Adolfo Lutz, Interdiciplinary Procedures Center, Strategic Laboratory	Claudia Regina Gonçalves; Claudio Tavares Sacchi; Erica Valessa Ramos Gomes

EPI_ISL_534317	Hospital Geral de Itapevi	Instituto Adolfo Lutz, Interdiciplinary Procedures Center, Strategic Laboratory	Claudia Regina Gonçalves; Claudio Tavares Sacchi; Erica Valessa Ramos Gomes
EPI_ISL_603037	Hospital Geral de Pedreira	Instituto Adolfo Lutz, Interdiciplinary Procedures Center, Strategic Laboratory	Claudia Regina Gonçalves; Claudio Tavares Sacchi; Erica Valessa Ramos Gomes; Karoline Rodrigues Campos
EPI_ISL_515555	Hospital Geral de Vila Nova Cachoeirinha	Instituto Adolfo Lutz, Interdiciplinary Procedures Center, Strategic Laboratory	Claudia Regina Gonçalves; Claudio Tavares Sacchi; Erica Valessa Ramos Gomes
EPI_ISL_574595	Hospital Geral de Vila Penteado Dr. Jose Pamgella	Instituto Adolfo Lutz, Interdiciplinary Procedures Center, Strategic Laboratory	Claudia Regina Gonçalves; Claudio Tavares Sacchi; Erica Valessa Ramos Gomes; Karoline Rodrigues Campos
EPI_ISL_471267, EPI_ISL_471268, EPI_ISL_481244, EPI_ISL_481245, EPI_ISL_481246, EPI_ISL_481247, EPI_ISL_481248, EPI_ISL_574431			
see above	Hospital IESS Babahoyo	Institute of Microbiology, Universidad San Francisco de Quito	Belén Prado-Vivar; Bernardo Gutiérrez; Carla Torres; Fernanda Zurita; Francisco Cordova; Gabriel Trueba; Juan José Guadalupe; Killen Briones-Claudette; Killen Briones-Zamora; Michelle Grunauer; Monica Becerra-Wong; Ninfa Henriquez; Patricio Rojas-Silva; Paúl Cárdenas; Sully Márquez; Verónica Barragán
EPI_ISL_412964	Hospital Israelita Albert Einstein	Instituto Adolfo Lutz Interdisciplinary Procedures Center Strategic Laboratory	Andrew Rambaut; Claudia Regina Gonçalves; Claudio Tavares Sacchi; Daniela Bernardes Borges da Silva; Ester Cerdeira Sabino; Flávia Cristina da Silva Sales; Ingra Morales Claro; Jaqueline Goes de Jesus; Joshua Quick; Maria do Carmo; Nicholas James Loman; Nuno Rodrigues Faria; Sampaio Tavares Timenetsky
EPI_ISL_414014, EPI_ISL_416033, EPI_ISL_416034	Hospital Israelita Albert Einstein	Instituto Adolfo Lutz, Interdisciplinary Procedures Center, Strategic Laboratory	Adriana Bugno; Adriano Abbud; Carlos Henrique Camargo; Claudia Regina Gonçalves; Claudio Tavares Sacchi; Daniela Bernardes Borges da Silva; Erica Valessa Ramos Gomes; Ester Cerdeira Sabino; Fabiana Cristina Pereira dos Santos; Katia Correia dos Santos; Maria do Carmo Sampaio Tavares Timenetsky; Maria do Carmo Sampaito Tavares Timenetsky; Simone Guadagnucci Morillo; Terezinha Maria de Paiva
EPI_ISL_413016	Hospital Israelita Albert Einstein	Instituto Adolfo Lutz, Interdisciplinary Procedures Center, Strategic Laboratory	Andrew Rambaut; Claudia Regina Gonçalves; Claudio Tavares Sacchi; Ester Cerdeira Sabino; Fabiana Cristina Pereira dos Santos; Flávia Cristina da Silva Sales; Ingra Morales Claro; Jaqueline Goes de Jesus; Joshua Quick; Maria do Carmo Sampaio Tavares Timenetsky; Nicholas James Loman; Nuno Rodrigues Faria
EPI_ISL_523957	Hospital Itamaraty	Instituto Adolfo Lutz, Interdiciplinary Procedures Center, Strategic Laboratory	Claudia Regina Gonçalves; Claudio Tavares Sacchi; Erica Valessa Ramos Gomes
EPI_ISL_527741, EPI_ISL_769992, EPI_ISL_769995	Hospital Metropolitano	Inciensa, Instituto Costarricense de Investigación y Enseñanza en Nutrición y Salud	Adriana Godinez & Melany Calderon; Adriana Godínez; Claudio Soto-Garita; Estela Cordero; Francisco Duarte; Hebleen Porras; Melany Calderón & Margarita Lee Lui
EPI_ISL_524462	Hospital Metropolitano	Instituto Adolfo Lutz, Interdiciplinary Procedures Center, Strategic Laboratory	Claudia Regina Gonçalves; Claudio Tavares Sacchi; Erica Valessa Ramos Gomes
EPI_ISL_458236, EPI_ISL_480312, EPI_ISL_480313, EPI_ISL_480314	Hospital Mexico	Charité Virology-University of Costa Rica	Andres Moreira-Soto; Eugenia Corrales-Aguilar; Ignacio Postigo-Hidalgo; Jan Felix Drexler; Teresita Somogyi
EPI_ISL_527739, EPI_ISL_527758	Hospital Mexico [San Jose/San Jose]	Inciensa, Instituto Costarricense de Investigación y Enseñanza en Nutrición y Salud	Adriana Godinez & Melany Calderon; Claudio Soto-Garita; Estela Cordero; Francisco Duarte; Hebleen Porras
EPI_ISL_515541, EPI_ISL_515561	Hospital Montemagno	Instituto Adolfo Lutz, Interdiciplinary Procedures Center, Strategic Laboratory	Claudia Regina Gonçalves; Claudio Tavares Sacchi; Erica Valessa Ramos Gomes
EPI_ISL_534324	Hospital Mun Ver Jose Storopolli	Instituto Adolfo Lutz, Interdiciplinary Procedures Center, Strategic Laboratory	Claudia Regina Gonçalves; Claudio Tavares Sacchi; Erica Valessa Ramos Gomes
EPI_ISL_534318, EPI_ISL_693208, EPI_ISL_693209	Hospital Municipal Antonio Giglio	Instituto Adolfo Lutz, Interdiciplinary Procedures Center, Strategic Laboratory	Claudia Regina Gonçalves; Claudio Tavares Sacchi; Erica Valessa Ramos Gomes; Karoline Rodrigues Campos
EPI_ISL_547571	Hospital Municipal Antônio Giglio	Instituto Adolfo Lutz, Interdiciplinary Procedures Center, Strategic Laboratory	Claudia Regina Gonçalves; Claudio Tavares Sacchi; Erica Valessa Ramos Gomes; Karoline Rodrigues Campos
EPI_ISL_471549, EPI_ISL_523991, EPI_ISL_523992, EPI_ISL_527869	Hospital Municipal Carmen Prudente	Instituto Adolfo Lutz, Interdiciplinary Procedures Center, Strategic Laboratory	Claudia Regina Gonçalves; Claudio Tavares Sacchi; Erica Valessa Ramos Gomes
EPI_ISL_515562	Hospital Municipal Doutor Alexandre Zaio	Instituto Adolfo Lutz, Interdiciplinary Procedures Center, Strategic Laboratory	Claudia Regina Gonçalves; Claudio Tavares Sacchi; Erica Valessa Ramos Gomes
EPI_ISL_693203	Hospital Municipal Doutor Arthur Ribeiro de Saboya	Instituto Adolfo Lutz, Interdiciplinary Procedures Center, Strategic Laboratory	Claudia Regina Gonçalves; Claudio Tavares Sacchi; Erica Valessa Ramos Gomes; Karoline Rodrigues Campos
EPI_ISL_468311, EPI_ISL_468312	Hospital Municipal Dr Ignacio Proenca de Gouvea	Instituto Adolfo Lutz, Interdiciplinary Procedures Center, Strategic Laboratory	Claudia Regina Gonçalves; Claudio Tavares Sacchi; Erica Valessa Ramos Gomes
EPI_ISL_515521	Hospital Municipal Dr Waldemar Tebaldi	Instituto Adolfo Lutz, Interdiciplinary Procedures Center, Strategic Laboratory	Claudia Regina Gonçalves; Claudio Tavares Sacchi; Erica Valessa Ramos Gomes
EPI_ISL_523985	Hospital Municipal Dr. Benedicto Montenegro	Instituto Adolfo Lutz, Interdiciplinary Procedures Center, Strategic Laboratory	Claudia Regina Gonçalves; Claudio Tavares Sacchi; Erica Valessa Ramos Gomes
EPI_ISL_515553, EPI_ISL_574577, EPI_ISL_574579	Hospital Municipal Dr. Ignacio Proença de Gouvea	Instituto Adolfo Lutz, Interdiciplinary Procedures Center, Strategic Laboratory	Claudia Regina Gonçalves; Claudio Tavares Sacchi; Erica Valessa Ramos Gomes; Karoline Rodrigues Campos
EPI_ISL_515548, EPI_ISL_515563, EPI_ISL_574582, EPI_ISL_574589	Hospital Municipal Dr. Jose Soares Hungria	Instituto Adolfo Lutz, Interdiciplinary Procedures Center, Strategic Laboratory	Claudia Regina Gonçalves; Claudio Tavares Sacchi; Erica Valessa Ramos Gomes; Karoline Rodrigues Campos
EPI_ISL_515557, EPI_ISL_524467	Hospital Municipal Dr. Moysés Deutsch	Instituto Adolfo Lutz, Interdiciplinary Procedures Center, Strategic Laboratory	Claudia Regina Gonçalves; Claudio Tavares Sacchi; Erica Valessa Ramos Gomes
EPI_ISL_583498	Hospital Municipal Dr. Waldemar Tebaldi	Instituto Adolfo Lutz, Interdiciplinary Procedures Center, Strategic Laboratory	Claudia Regina Gonçalves; Claudio Tavares Sacchi; Erica Valessa Ramos Gomes; Karoline Rodrigues Campos
EPI_ISL_693206	Hospital Municipal Mario Gatti	Instituto Adolfo Lutz, Interdiciplinary Procedures Center, Strategic Laboratory	Claudia Regina Gonçalves; Claudio Tavares Sacchi; Erica Valessa Ramos Gomes; Karoline Rodrigues Campos
EPI_ISL_527870, EPI_ISL_574578, EPI_ISL_603029, EPI_ISL_603039	Hospital Municipal Mário Gatti	Instituto Adolfo Lutz, Interdiciplinary Procedures Center, Strategic Laboratory	Claudia Regina Gonçalves; Claudio Tavares Sacchi; Erica Valessa Ramos Gomes; Karoline Rodrigues Campos
EPI_ISL_527856	Hospital Municipal Prof. Waldomiro de Paula	Instituto Adolfo Lutz, Interdiciplinary Procedures Center, Strategic Laboratory	Claudia Regina Gonçalves; Claudio Tavares Sacchi; Erica Valessa Ramos Gomes
EPI_ISL_603028	Hospital Municipal Santa Ana	Instituto Adolfo Lutz, Interdiciplinary Procedures Center, Strategic Laboratory	Claudia Regina Gonçalves; Claudio Tavares Sacchi; Erica Valessa Ramos Gomes; Karoline Rodrigues Campos
EPI_ISL_524468, EPI_ISL_527859	Hospital Municipal Vereador Jose Storopolli	Instituto Adolfo Lutz, Interdiciplinary Procedures Center, Strategic Laboratory	Claudia Regina Gonçalves; Claudio Tavares Sacchi; Erica Valessa Ramos Gomes
EPI_ISL_471647	Hospital Municipal de Barueri Dr. Francisco Moran	Instituto Adolfo Lutz, Interdiciplinary Procedures Center, Strategic Laboratory	Claudia Regina Gonçalves; Claudio Tavares Sacchi; Erica Valessa Ramos Gomes

EPI_ISL_527860	Hospital Municipal de Parelheiros Josanias Castanha Braga	Instituto Adolfo Lutz, Interdiciplinary Procedures Center, Strategic Laboratory	Claudia Regina Gonçalves; Claudio Tavares Sacchi; Erica Valessa Ramos Gomes
EPI_ISL_527862	Hospital Municipal de Urgência	Instituto Adolfo Lutz, Interdiciplinary Procedures Center, Strategic Laboratory	Claudia Regina Gonçalves; Claudio Tavares Sacchi; Erica Valessa Ramos Gomes
EPI_ISL_468308, EPI_ISL_468315, EPI_ISL_515520, EPI_ISL_515526, EPI_ISL_515546, EPI_ISL_515551, EPI_ISL_515552, EPI_ISL_523955, EPI_ISL_523974, EPI_ISL_523976, EPI_ISL_527863			
see above	Hospital Municipal do Tatuape Carmino Caricchio	Instituto Adolfo Lutz, Interdiciplinary Procedures Center, Strategic Laboratory	Claudia Regina Gonçalves; Claudio Tavares Sacchi; Erica Valessa Ramos Gomes
EPI_ISL_735399	Hospital Municiplal Dr Ignacio de gouvea	Instituto Adolfo Lutz, Interdiciplinary Procedures Center, Strategic Laboratory	Claudia Regina Gonçalves; Claudio Tavares Sacchi; Erica Valessa Ramos Gomes; Karoline Rodrigues Campos
EPI_ISL_836977	Hospital Municiplal Dr. Jose de Carvalho Florence	Instituto Adolfo Lutz, Interdiciplinary Procedures Center, Strategic Laboratory	Claudia Regina Gonçalves; Claudio Tavares Sacchi; Erica Valessa Ramos Gomes; Karoline Rodrigues Campos
EPI_ISL_491451, EPI_ISL_491453	Hospital México	Inciensa, Instituto Costarricense de Investigación y Enseñanza en Nutrición y Salud	Adriana Godinez & Melany Calderon; Claudio Soto-Garita; Estela Cordero; Francisco Duarte; Hebleen Brenes
EPI_ISL_480322, EPI_ISL_480323, EPI_ISL_480325, EPI_ISL_480326, EPI_ISL_480327	Hospital Nacional de Niños	Charité Virology-University of Costa Rica	Andrei Montero Bonilla; Andres Moreira-Soto; Cristian Pérez Corrales; Eugenia Corrales-Aguilar; Ignacio Postigo-Hidalgo; Jan Felix Drexler
EPI_ISL_735428, EPI_ISL_735429, EPI_ISL_735431, EPI_ISL_735432	Hospital Nipo Brasileiro	Instituto Adolfo Lutz, Interdiciplinary Procedures Center, Strategic Laboratory	Claudia Regina Gonçalves; Claudio Tavares Sacchi; Erica Valessa Ramos Gomes; Karoline Rodrigues Campos
EPI_ISL_471269, EPI_ISL_471270, EPI_ISL_471271	Hospital Oncológico Solca Núcleo de Quito	Institute of Microbiology, Universidad San Francisco de Quito	Belén Prado-Vivar; Bernardo Gutiérrez; Gabriel Trueba; Grace Salazar; Juan José Guadalupe; Marcos Di Stefano; Michelle Grunauer; Patricio Rojas-Silva; Paúl Cárdenas; Sully Márquez; Verónica Barragán
EPI_ISL_527857	Hospital Regional Vale do Ribeira	Instituto Adolfo Lutz, Interdiciplinary Procedures Center, Strategic Laboratory	Claudia Regina Gonçalves; Claudio Tavares Sacchi; Erica Valessa Ramos Gomes
EPI_ISL_523956	Hospital Regional de Assis	Instituto Adolfo Lutz, Interdiciplinary Procedures Center, Strategic Laboratory	Claudia Regina Gonçalves; Claudio Tavares Sacchi; Erica Valessa Ramos Gomes
EPI_ISL_524463	Hospital Regional de Cotia	Instituto Adolfo Lutz, Interdiciplinary Procedures Center, Strategic Laboratory	Claudia Regina Gonçalves; Claudio Tavares Sacchi; Erica Valessa Ramos Gomes
EPI_ISL_735418	Hospital Regional do Vale do Paraiba	Instituto Adolfo Lutz, Interdiciplinary Procedures Center, Strategic Laboratory	Claudia Regina Gonçalves; Claudio Tavares Sacchi; Erica Valessa Ramos Gomes; Karoline Rodrigues Campos
EPI_ISL_833165, EPI_ISL_833166	Hospital Samaritano	Instituto Adolfo Lutz, Interdiciplinary Procedures Center, Strategic Laboratory	Claudia Regina Gonçalves; Claudio Tavares Sacchi; Erica Valessa Ramos Gomes; Karoline Rodrigues Campos
EPI_ISL_491450, EPI_ISL_491454, EPI_ISL_491456	Hospital San Juan de Dios	Inciensa, Instituto Costarricense de Investigación y Enseñanza en Nutrición y Salud	Adriana Godinez & Melany Calderon; Claudio Soto-Garita; Estela Cordero; Francisco Duarte; Hebleen Brenes
EPI_ISL_491438, EPI_ISL_491440, EPI_ISL_491448, EPI_ISL_491452	Hospital San Rafael de Alajuela	Inciensa, Instituto Costarricense de Investigación y Enseñanza en Nutrición y Salud	Adriana Godinez & Melany Calderon; Claudio Soto-Garita; Estela Cordero; Francisco Duarte; Hebleen Brenes
EPI_ISL_527753, EPI_ISL_527754	Hospital San Vicente De Paul	Inciensa, Instituto Costarricense de Investigación y Enseñanza en Nutrición y Salud	Adriana Godinez & Melany Calderon; Claudio Soto-Garita; Estela Cordero; Francisco Duarte; Hebleen Porras
EPI_ISL_434536	Hospital San Vicente de Paul	Inciensa, Instituto Costarricense de Investigación y Enseñanza en Nutrición y Salud	Adriana Godinez & Melany Calderon; Claudio Soto-Garita; Estela Cordero; Francisco Duarte; Hebleen Porras
EPI_ISL_471552, EPI_ISL_523967	Hospital Sancta Maggiore	Instituto Adolfo Lutz, Interdiciplinary Procedures Center, Strategic Laboratory	Claudia Regina Gonçalves; Claudio Tavares Sacchi; Erica Valessa Ramos Gomes
EPI_ISL_603036	Hospital Santa Ana	Instituto Adolfo Lutz, Interdiciplinary Procedures Center, Strategic Laboratory	Claudia Regina Gonçalves; Claudio Tavares Sacchi; Erica Valessa Ramos Gomes; Karoline Rodrigues Campos
EPI_ISL_515527, EPI_ISL_693196	Hospital Santa Clara	Instituto Adolfo Lutz, Interdiciplinary Procedures Center, Strategic Laboratory	Claudia Regina Gonçalves; Claudio Tavares Sacchi; Erica Valessa Ramos Gomes; Karoline Rodrigues Campos
EPI_ISL_693233	Hospital Santa Cruz	Instituto Adolfo Lutz, Interdiciplinary Procedures Center, Strategic Laboratory	Claudia Regina Gonçalves; Claudio Tavares Sacchi; Erica Valessa Ramos Gomes; Karoline Rodrigues Campos
EPI_ISL_693236	Hospital Santa Marcelina Sao Paulo	Instituto Adolfo Lutz, Interdiciplinary Procedures Center, Strategic Laboratory	Claudia Regina Gonçalves; Claudio Tavares Sacchi; Erica Valessa Ramos Gomes; Karoline Rodrigues Campos
EPI_ISL_468310	Hospital Sao Paulo de Ensino da UNIFESP	Instituto Adolfo Lutz, Interdiciplinary Procedures Center, Strategic Laboratory	Claudia Regina Gonçalves; Claudio Tavares Sacchi; Erica Valessa Ramos Gomes
EPI_ISL_471545, EPI_ISL_471551, EPI_ISL_515528, EPI_ISL_515545, EPI_ISL_515559, EPI_ISL_515560, EPI_ISL_523969, EPI_ISL_523981, EPI_ISL_523988, EPI_ISL_693201			
see above	Hospital Sao Paulo de Ensino da Unifesp	Instituto Adolfo Lutz, Interdiciplinary Procedures Center, Strategic Laboratory	Claudia Regina Gonçalves; Claudio Tavares Sacchi; Erica Valessa Ramos Gomes; Karoline Rodrigues Campos
EPI_ISL_414015, EPI_ISL_414016, EPI_ISL_414017	Hospital São Joaquim Beneficencia Portuguesa	Instituto Adolfo Lutz, Interdiciplinary Procedures Center, Strategic Laboratory	Audrey Cilli; Carlos Henrique Camargo; Claudia Regina Gonçalves; Claudio Tavares Sacchi; Daniela Bernardes Borges da Silva; Ester Cerdeira Sabino; Fabiana Cristina Pereira dos Santos; Fabiana Cristina Pereira dos Santos Terezinha Maria de Paiva; Maria do Carmo Sampaito Tavares Timenetsky; SimoneGuadagnucci Morillo; Terezinha Maria de Paiva
EPI_ISL_819335	Hospital Universitari Vall d'Hebron - Vall d'Hebron Institut de Rercerca	Hospital Universitari Vall d'Hebron	Andrés Antón; Ariadna Rando; Carla Castillo; Cristina Andrés; Damir Garcia-Cehic; Josep F Abril; Josep Quer; Juliana Esperalba; Maria Carmen Martin; Maria Gema Codina; Maria Piñana; Tomàs Pumarola
EPI_ISL_421176, EPI_ISL_428686, EPI_ISL_529977, EPI_ISL_529980	Hospital Universitario 12 de Octubre	Hospital Universitario 12 de Octubre	Elias Dahdouh; Esther Viedma; Fernando Lázaro; Jesús Mingorance; Juan Carlos Galán; Julio García; Mª Dolores Folgueira; Natalia Stella; Rafael Cantón; Rafael Delgado; Raúl Recio; Sara González
EPI_ISL_417963	Hospital Universitario 12 de Octubre	Hospital Universitario La Paz	Elias Dahdouh; Esther Viedma; Fernando Lázaro; Jesús Mingorance; Juan Carlos Galán; Julio García; Mª Dolores Folgueira; Natalia Stella; Rafael Cantón; Rafael Delgado; Sara González
EPI_ISL_467096, EPI_ISL_467124, EPI_ISL_467130, EPI_ISL_467138, EPI_ISL_467144, EPI_ISL_467150, EPI_ISL_467177, EPI_ISL_467182			
see above	Hospital Universitario Araba. Vitoria-Gasteiz	SeqCOVID-SPAIN consortium/IBV(CSIC)	Amaia Aguirre Quiñonero; Andrés Canut Blasco. and SeqCOVID-SPAIN consortium; Carmen Gómez González; Marina Fernández Torres; Mª Concepción Lecaroz Agara; Mª Rosario Almela Ferrer; Silvia Hernáez Crespo
EPI_ISL_452722, EPI_ISL_452735, EPI_ISL_452747	Hospital Universitario Araba. Vitoria-Gasteiz,	SeqCOVID-SPAIN consortium/IBV(CSIC)	Amaia Aguirre Quiñonero; Andrés Canut Blasco and SeqCOVID-SPAIN consortium; Carmen Gómez González; Maria Concepción Lecaroz Agara; Maria Rosario Almela Ferrer; Marina Fernández Torres; Silvia Hernáez Crespo
EPI_ISL_831057, EPI_ISL_831072, EPI_ISL_831082	Hospital Universitario La Paz (Madrid)	SeqCOVID-SPAIN consortium/IBV(CSIC)	Elias Dahdouh; Fernando Lázaro-Perona; Jesús Mingorance and SeqCOVID-SPAIN consortium; María Rodríguez-Tejedor
EPI_ISL_537809	Hospital Universitario Marqués de Valdecilla (Santander),	SeqCOVID-SPAIN consortium/IBV(CSIC)	Daniel Pablo Marcos; Jesús Rodríguez Lozano; Jose Manuel Méndez Legaza; María Eliecer Cano García; María Siller Ruiz and SeqCOVID-SPAIN

	Servicio de Microbiología		consortium; Mónica Gozalo Margüello
EPI_ISL_467062	Hospital Universitario Virgen de las Nieves de Granada-SAS	SeqCOVID-SPAIN consortium/IBV(CSIC)	Irene Pedrosa Corral; José M. Navarro-Marí and SeqCOVID-SPAIN consortium; Mercedes Pérez Ruiz; Sara Sanbonmatsu Gámez
EPI_ISL_468318, EPI_ISL_468321, EPI_ISL_547574	Hospital Universitario da USP	Instituto Adolfo Lutz, Interdiciplinary Procedures Center, Strategic Laboratory	Claudia Regina Gonçalves; Claudio Tavares Sacchi; Erica Valessa Ramos Gomes; Karoline Rodrigues Campos
EPI_ISL_471539	Hospital Universitario da USP Sao Paulo	Instituto Adolfo Lutz, Interdiciplinary Procedures Center, Strategic Laboratory	Claudia Regina Gonçalves; Claudio Tavares Sacchi; Erica Valessa Ramos Gomes
EPI_ISL_534314	Hospital Universitario da USP de SP	Instituto Adolfo Lutz, Interdiciplinary Procedures Center, Strategic Laboratory	Claudia Regina Gonçalves; Claudio Tavares Sacchi; Erica Valessa Ramos Gomes
EPI_ISL_539254	Hospital Universitario de La Ribera (Alzira, València)	SeqCOVID-SPAIN consortium/IBV(CSIC)	Julia González and SeqCOVID-SPAIN consortium; Olalla Martínez Macias
EPI_ISL_476204, EPI_ISL_476243, EPI_ISL_476244, EPI_ISL_476245, EPI_ISL_476246, EPI_ISL_476250, EPI_ISL_476254, EPI_ISL_476256, EPI_ISL_476259, EPI_ISL_476260, EPI_ISL_476261, EPI_ISL_476262, EPI_ISL_476265, EPI_ISL_476266, EPI_ISL_476267, EPI_ISL_476268, EPI_ISL_476274, EPI_ISL_476373, EPI_ISL_476374, EPI_ISL_476375, EPI_ISL_476376, EPI_ISL_476379, EPI_ISL_476380, EPI_ISL_476383, EPI_ISL_476384, EPI_ISL_476386, EPI_ISL_476431, EPI_ISL_476432, EPI_ISL_476435, EPI_ISL_476437, EPI_ISL_476439, EPI_ISL_476441, EPI_ISL_476443, EPI_ISL_476445, EPI_ISL_476446, EPI_ISL_476447, EPI_ISL_476448, EPI_ISL_476450, EPI_ISL_476452, EPI_ISL_476456, EPI_ISL_476461, EPI_ISL_476462, EPI_ISL_476464, EPI_ISL_476467, EPI_ISL_476469, EPI_ISL_476471, EPI_ISL_476472, EPI_ISL_476484, EPI_ISL_476486, EPI_ISL_476487, EPI_ISL_476488, EPI_ISL_476489, EPI_ISL_476490			
see above	Hospital da Clínicas da Faculdade de Medicina da Universidade de São Paulo	Instituto de Medicina Tropical da Univesidade de São Paulo	Camila Alves Maia da Silva; Carolina S. Lazar; Cecilia Salete Alencar; Darlan da Silva Candido; Erika Regina Manuli; Ester Sabino; Flavia Cristina da Silva Sales; Giula Magalhaes Ferreira; Jaqueline Goes de Jesus; Julien Theze; Mariana Severo Ramundo; Nuno Faria; Samples: Ingra Morales Claro; Sequencing: Ingra Morales Claro; Silvia F. Costa; Thais de Moura Coletti
EPI_ISL_534313	Hospital da Sta Casa de Sto Amaro	Instituto Adolfo Lutz, Interdiciplinary Procedures Center, Strategic Laboratory	Claudia Regina Gonçalves; Claudio Tavares Sacchi; Erica Valessa Ramos Gomes
EPI_ISL_721987, EPI_ISL_721988, EPI_ISL_721989, EPI_ISL_721990, EPI_ISL_721991, EPI_ISL_721992, EPI_ISL_721993, EPI_ISL_721994, EPI_ISL_721995, EPI_ISL_721996, EPI_ISL_721997, EPI_ISL_721998, EPI_ISL_721999, EPI_ISL_722000, EPI_ISL_722001, EPI_ISL_722002, EPI_ISL_722003, EPI_ISL_722004, EPI_ISL_722005, EPI_ISL_722006, EPI_ISL_722007, EPI_ISL_722008, EPI_ISL_722009, EPI_ISL_722010, EPI_ISL_722011, EPI_ISL_722012, EPI_ISL_722013, EPI_ISL_722014, EPI_ISL_722015, EPI_ISL_722016, EPI_ISL_722017, EPI_ISL_722018, EPI_ISL_722019, EPI_ISL_722020, EPI_ISL_722021, EPI_ISL_722022, EPI_ISL_722023, EPI_ISL_722024, EPI_ISL_722025, EPI_ISL_722026, EPI_ISL_722027, EPI_ISL_722028, EPI_ISL_722029, EPI_ISL_722030, EPI_ISL_722031, EPI_ISL_722032, EPI_ISL_722033, EPI_ISL_722034, EPI_ISL_722035, EPI_ISL_722036, EPI_ISL_722037, EPI_ISL_722038, EPI_ISL_722039, EPI_ISL_722040, EPI_ISL_722041, EPI_ISL_722042, EPI_ISL_722043, EPI_ISL_722044, EPI_ISL_722045, EPI_ISL_722046, EPI_ISL_722047, EPI_ISL_722048, EPI_ISL_722049, EPI_ISL_722050, EPI_ISL_722051, EPI_ISL_722129			
see above	Hospital das Clínicas Universidade de São Paulo Medical School	Laboratório de Parasitologia Médica - Instituto de Medicina Tropical - Universidade de São Paulo	Brazil-UK Centre for Arbovirus Discovery Diagnosis Genomics and Epidemiology (CADDE) Genomic Network - Instituto de Medicina Tropical
EPI_ISL_672687, EPI_ISL_672688, EPI_ISL_672693, EPI_ISL_672695, EPI_ISL_672696, EPI_ISL_672699, EPI_ISL_672700, EPI_ISL_672722, EPI_ISL_672725, EPI_ISL_672728, EPI_ISL_672731, EPI_ISL_672734, EPI_ISL_672735, EPI_ISL_672736, EPI_ISL_672739, EPI_ISL_672741			
see above	Hospital das Clínicas da Faculdade de Medicina da Universidade de São Paulo (HC-FMUSP)	Laboratório de Parasitologia Médica - Instituto de Medicina Tropical - Universidade de São Paulo	Brazil-UK Centre for Arbovirus Discovery Diagnosis Genomics and Epidemiology (CADDE) Genomic Network - Instituto de Medicina Tropical
EPI_ISL_836143	Hospital de Campanha COVID-19 de Mairipora	Instituto Adolfo Lutz, Interdiciplinary Procedures Center, Strategic Laboratory	Claudia Regina Gonçalves; Claudio Tavares Sacchi; Erica Valessa Ramos Gomes; Karoline Rodrigues Campos
EPI_ISL_693205	Hospital de Campanha Covid-19 Assis	Instituto Adolfo Lutz, Interdiciplinary Procedures Center, Strategic Laboratory	Claudia Regina Gonçalves; Claudio Tavares Sacchi; Erica Valessa Ramos Gomes; Karoline Rodrigues Campos
EPI_ISL_735396	Hospital de Camplanha COVID 19 SER	Instituto Adolfo Lutz, Interdiciplinary Procedures Center, Strategic Laboratory	Claudia Regina Gonçalves; Claudio Tavares Sacchi; Erica Valessa Ramos Gomes; Karoline Rodrigues Campos
EPI_ISL_476563, EPI_ISL_476565, EPI_ISL_476567, EPI_ISL_476568, EPI_ISL_476571, EPI_ISL_476573, EPI_ISL_615121			
see above	Hospital de Pediatría "Prof. Dr. Juan P Garrahan"	Héritas	Andrea Mangano; Bianca Brun; Cristian Rohr; Dalmacio Pereyra; Fabian Fay; Maria Florencia Fernandez; Martin Vazquez; Mauricio Grisolia; Priscila Aldabe; Roberta Crespo
EPI_ISL_414577, EPI_ISL_414578	Hospital de Talca, Chile	Instituto de Salud Publica de Chile	Alejandra Acevedo; Andrés E. Castillo; Bárbara Parra; Carolina Tambley; Gabriel Leal; Gisselle Barra; Jaime Lagos; Javier Tognarelli; Jorge Fernández.; Loredana Arata; Patricia Bustos; Paz Tapia; Rodrigo Fasce; Soledad Ulloa; Winston Andrade
EPI_ISL_534319, EPI_ISL_534320	Hospital do Serv Pub ESTAFCO Morato de Oliveira	Instituto Adolfo Lutz, Interdiciplinary Procedures Center, Strategic Laboratory	Claudia Regina Gonçalves; Claudio Tavares Sacchi; Erica Valessa Ramos Gomes
EPI_ISL_693199	Hospital do Servidor Publico Estadual Francisco Morato de Oliveira	Instituto Adolfo Lutz, Interdiciplinary Procedures Center, Strategic Laboratory	Claudia Regina Gonçalves; Claudio Tavares Sacchi; Erica Valessa Ramos Gomes; Karoline Rodrigues Campos
EPI_ISL_471548, EPI_ISL_515565, EPI_ISL_523965, EPI_ISL_523972, EPI_ISL_523978, EPI_ISL_523982, EPI_ISL_524470			
see above	Hospital do Servidor Público Estadual Francisco Morato de Oliveira	Instituto Adolfo Lutz, Interdiciplinary Procedures Center, Strategic Laboratory	Claudia Regina Gonçalves; Claudio Tavares Sacchi; Erica Valessa Ramos Gomes
EPI_ISL_527861	Hospital e Maternidade Celso Pierro	Instituto Adolfo Lutz, Interdiciplinary Procedures Center, Strategic Laboratory	01246-1301; 355 - Brazil; Av. Dr. Arnaldo; Cerqueira Cesar; São Paulo - SP
EPI_ISL_693200	Hospital e Maternidade Mairipora	Instituto Adolfo Lutz, Interdiciplinary Procedures Center, Strategic Laboratory	Claudia Regina Gonçalves; Claudio Tavares Sacchi; Erica Valessa Ramos Gomes; Karoline Rodrigues Campos
EPI_ISL_547577	Hospital e Maternidade Nossa Senhora das Graças	Instituto Adolfo Lutz, Interdiciplinary Procedures Center, Strategic Laboratory	Claudia Regina Gonçalves; Claudio Tavares Sacchi; Erica Valessa Ramos Gomes; Karoline Rodrigues Campos
EPI_ISL_693241, EPI_ISL_735425	Hospital e Maternidade Sao Lucas	Instituto Adolfo Lutz, Interdiciplinary Procedures Center, Strategic Laboratory	Claudia Regina Gonçalves; Claudio Tavares Sacchi; Erica Valessa Ramos Gomes; Karoline Rodrigues Campos
EPI_ISL_527865	Hospital e Maternidade São Cristóvão	Instituto Adolfo Lutz, Interdiciplinary Procedures Center, Strategic Laboratory	Claudia Regina Gonçalves; Claudio Tavares Sacchi; Erica Valessa Ramos Gomes
EPI_ISL_527868	Hospital e Maternidade do Braz	Instituto Adolfo Lutz, Interdiciplinary Procedures Center, Strategic Laboratory	Claudia Regina Gonçalves; Claudio Tavares Sacchi; Erica Valessa Ramos Gomes
EPI_ISL_534323	Hospital e Pronto Socorro Comunitario Vila Yolanda	Instituto Adolfo Lutz, Interdiciplinary Procedures Center, Strategic Laboratory	Claudia Regina Gonçalves; Claudio Tavares Sacchi; Erica Valessa Ramos Gomes
EPI_ISL_527864	Hospital e Pronto Socorro Comunitário Vila Iolanda	Instituto Adolfo Lutz, Interdiciplinary Procedures Center, Strategic Laboratory	Claudia Regina Gonçalves; Claudio Tavares Sacchi; Erica Valessa Ramos Gomes
EPI_ISL_693195, EPI_ISL_693230, EPI_ISL_693232, EPI_ISL_735412	Hospital e Pronto Socorro Portinari	Instituto Adolfo Lutz, Interdiciplinary Procedures Center, Strategic Laboratory	Claudia Regina Gonçalves; Claudio Tavares Sacchi; Erica Valessa Ramos Gomes; Karoline Rodrigues Campos
EPI_ISL_434916, EPI_ISL_542573, EPI_ISL_542598, EPI_ISL_544102, EPI_ISL_545867, EPI_ISL_545901, EPI_ISL_546352, EPI_ISL_546565, EPI_ISL_546893, EPI_ISL_546913, EPI_ISL_780156, EPI_ISL_780687, EPI_ISL_781174, EPI_ISL_785420, EPI_ISL_789651			
see above	Houston Methodist Hospital	Houston Methodist Hospital	Chia-Wei Chou; Concepcion C. Cantu; Daniel Boutz; David W. Bernard; Ghazaleh Eskandari; Hakon Jonsson; Heather Hendrickson; Hoang A. T. Nguyen; Hung-Che Kuo; Ilya J. Finkelstein; J. Hunter Long; James J. Davis; Jason S. McLellan; Jimmy Gollihar; Jule Goike; Kamyab Javanmardi; Kari Stefansson; Layne Pruitt; Marcus Nguyen; Matthew Ojeda Saavedra; Maulik Shukla; Muthiah Kumaraswami; Paul A. Christensen; Prasanti Yerramilli; Randall J. Olsen; S. Wesley Long; Sishir Subedi; and James M. Musser
EPI_ISL_536046	Hôpital d'Alma	Laboratoire de santé publique du Québec	Guillaume Bourque; Ioannis Ragoussis; Jesse Shapiro; Mark Lathrop and Michel Roger; Sandrine Moreira
EPI_ISL_535889	Hôpital de Saint-Eustache	Laboratoire de santé publique du Québec	Guillaume Bourque; Ioannis Ragoussis; Jesse Shapiro; Mark Lathrop and Michel Roger; Sandrine Moreira
EPI_ISL_536201	Hôpital du Suroît	Laboratoire de santé publique du Québec	Guillaume Bourque; Ioannis Ragoussis; Jesse Shapiro; Mark Lathrop and Michel Roger; Sandrine Moreira

EPI_ISL_647990, EPI_ISL_647999	IA State Hygienic Laboratory	Pathogen Discovery, Respiratory Viruses Branch, Division of Viral Diseases, Centers for Disease Control and Prevention	Anna Uehara; Brian Lynch; Clinton R. Paden; Haibin Wang; Jing Zhang; Krista Queen; Suxiang Tong; Yan Li; Ying Tao
EPI_ISL_455647	ICMR-National Institute of Cholera and Enteric Diseases	National Institute of Biomedical Genomics	Ananya Chatterjee; Arindam Maitra; Hasina Banu; Mamta Chawla Sarkar; Saumitra Das; Shanta Dutta; Sreedhar Chinnaswamy
EPI_ISL_445379	IMALAB- HOSPITAL FACH	Instituto de Salud Publica de Chile	Alejandra Acevedo; Andrés E Castillo; Bárbara Parra; Carolina Tambley; Gabriel Leal; Jaime Lagos; Jorge Fernandez; Loredana Arata; Patricia Bustos; Paz Tapia; Rodrigo Fasce; Winston Andrade
EPI_ISL_648209, EPI_ISL_648210, EPI_ISL_648211, EPI_ISL_648212, EPI_ISL_648213, EPI_ISL_648214, EPI_ISL_648215, EPI_ISL_648216, EPI_ISL_648218, EPI_ISL_648677			
see above	INBIRS-UBA	Laboratorio Mixto de Biotecnología Acuática (LMBA)	Adriana Giri; Agustina Cerri; Ana Cavatorta; Ana Paletta; Diego Chouhy; Elisa Bolatti; Elizabeth Tapia; Federico Remes Lenicov; Flavio Spetale; Gastón Viarengo; Ignacio García Labari; Javier Murillo; Joaquín Ezpeleta; Julian Acosta; Laura Angelone; Leandro Ciappina; María Re; Pablo Casal; Pilar Bulacio; Silvana Spinelli; Silvia Arranz; Sofía Lavista Llanos; Vanina Villanova; Victoria Posner
EPI_ISL_603173	INMI Lazzaro Spallanzani IRCCS	INMI Lazzaro Spallanzani IRCCS	Antonino Di Caro; Barbara Bartolini; Cesare E.M. Gruber; Emanuela Giombini; Francesco Messina; Giuseppina Cappiello; Maria R. Capobianchi; Martina Rueca; Simone Lanini
EPI_ISL_806544	INSPI Instituto Nacional de Investigación en Salud Pública	Av. Julián Coronel 905 entre Esmeraldas y José Mascote Av. Juan Tanca Marengo No. 100 y Av. de las Américas	Alberto Orlando.; Alfredo Bruno; Andrés Carrazco; Doménica de Mora; Leandro Patiño; Manuel González; Maritza Olmedo; Mary Regato; Melissa Zambrano; Orson Mestanza
EPI_ISL_826798, EPI_ISL_826800, EPI_ISL_826802, EPI_ISL_826804, EPI_ISL_826805, EPI_ISL_826807, EPI_ISL_826809, EPI_ISL_826811, EPI_ISL_826812, EPI_ISL_826814, EPI_ISL_826816, EPI_ISL_826818, EPI_ISL_826820, EPI_ISL_826822, EPI_ISL_826823, EPI_ISL_826825, EPI_ISL_826827, EPI_ISL_826829, EPI_ISL_826830, EPI_ISL_826832, EPI_ISL_826834, EPI_ISL_826836, EPI_ISL_831020			
see above	INSPI-CRN DE INFLUENZA Y OTROS VIRUS RESPIRATORIOS	Instituto de Salud Publica de Chile	Alfredo Bruno; Andres Castillo; Barbara Parra; Domenica de Mora; Gisselle Barra; Jaime Lagos; Javier Tognarelli; Jimmy Garcez; Jorge Fernandez; Loredana Arata; Manuel Gonzalez; Martiza Olmedo; Michelle Paez; Patricia Bustos; Rodrigo Fasce; Solon Narvaez
EPI_ISL_445302	INSTITUTO MEDICO LEGAL	Instituto de Salud Publica de Chile	Alejandra Acevedo; Andrés E Castillo; Bárbara Parra; Carolina Tambley; Gabriel Leal; Jaime Lagos; Jorge Fernandez; Loredana Arata; Patricia Bustos; Paz Tapia; Rodrigo Fasce; Winston Andrade
EPI_ISL_445357	INTEGRAMEDICA CENTROS MEDICOS S.A.	Instituto de Salud Publica de Chile	Alejandra Acevedo; Andrés E Castillo; Bárbara Parra; Carolina Tambley; Gabriel Leal; Jaime Lagos; Jorge Fernandez; Loredana Arata; Patricia Bustos; Paz Tapia; Rodrigo Fasce; Winston Andrade
EPI_ISL_445261	INTEGRAMEDICA LAB. CLINICO LTDA.	Instituto de Salud Publica de Chile	Alejandra Acevedo; Andrés E Castillo; Bárbara Parra; Carolina Tambley; Gabriel Leal; Jaime Lagos; Jorge Fernandez; Loredana Arata; Patricia Bustos; Paz Tapia; Rodrigo Fasce; Winston Andrade
EPI_ISL_445324	INTEGRAMEDICA S.A	Instituto de Salud Publica de Chile	Alejandra Acevedo; Andrés E Castillo; Bárbara Parra; Carolina Tambley; Gabriel Leal; Jaime Lagos; Jorge Fernandez; Loredana Arata; Patricia Bustos; Paz Tapia; Rodrigo Fasce; Winston Andrade
EPI_ISL_751358, EPI_ISL_751440, EPI_ISL_751447	IRCCS Sacro Cuore Don Calabria Hospital, Department of Infectious, Tropical Diseases & Microbiology	University of Verona, Department of Biotechnology	Antonio Mori; Chiara Degli Esposti; Chiara Piubelli; Cristina Beltrami; Elena Pomari; Emanuela Cosentino; Giulia Lopatriello; Luca Marcolungo; Massimo Delledonne; Michela Deiana
EPI_ISL_476702, EPI_ISL_476704	Incubadora Venezolana de Ciencia, Venezuela	Incubadora Venezolana de Ciencia, Venezuela / Instituto Nacional de Salud, Bogotá, Colombia / Grupo de Investigaciones Microbiológicas-UR (GIMUR), Departamento de Biología, Facultad de Ciencias Naturales, Universidad del Rosario, Bogotá, Colombia / Icahn School of Medicine at Mount Sinai, New York, USA	Alberto Paniz-Mondolfi; Ana S. Gonzalez-Reiche; Angelica Rico; Aníbal A. Teherán; Carolina Florez; Carolina Hernández; Emilia Mia Sordillo; Esther C. Barros; Harm van Bakel; Jesús E. Jaimes; Juan David Ramírez; Lisseth Pardo; Lourdes Delgado; Luis Perez-Garcia; Marina Muñoz; Matthew M. Hernandez; Sergio Gomez; Viviana Simon
EPI_ISL_496339, EPI_ISL_496340, EPI_ISL_496341, EPI_ISL_496342, EPI_ISL_496343, EPI_ISL_496344, EPI_ISL_496345, EPI_ISL_496346, EPI_ISL_496347, EPI_ISL_496348, EPI_ISL_496349, EPI_ISL_496350, EPI_ISL_496351, EPI_ISL_496352, EPI_ISL_496353, EPI_ISL_496354, EPI_ISL_496355, EPI_ISL_496356, EPI_ISL_496357, EPI_ISL_496358, EPI_ISL_496359, EPI_ISL_496360, EPI_ISL_496361, EPI_ISL_496362, EPI_ISL_496363, EPI_ISL_496364, EPI_ISL_496365, EPI_ISL_496366, EPI_ISL_496367, EPI_ISL_496368, EPI_ISL_496369, EPI_ISL_496370, EPI_ISL_496371, EPI_ISL_496372, EPI_ISL_496373, EPI_ISL_496374, EPI_ISL_496375, EPI_ISL_496376			
see above	Infectolab	Andersen lab at Scripps Research	Carlos A. Cota Haros; Octavio Renteria Pacheco; SEARCH Alliance San Diego with Samuel Navarro Alvarez
EPI_ISL_480556	Institut Pasteur Dakar	Institut Pasteur de Dakar	Amadou Alpha Sall.; Mamadou Diop; Mamadou Malado Jallow; Marie Henriette Dior Ndione; Moussa Moise Diagne; Ndongo Dia; Ousmane Faye; Safietou Sanke
EPI_ISL_613419, EPI_ISL_613420, EPI_ISL_613421, EPI_ISL_613422, EPI_ISL_613423, EPI_ISL_613424, EPI_ISL_613425, EPI_ISL_613426, EPI_ISL_613427, EPI_ISL_613428, EPI_ISL_613429, EPI_ISL_613430, EPI_ISL_613431, EPI_ISL_613432, EPI_ISL_613433, EPI_ISL_613434, EPI_ISL_613435, EPI_ISL_613436, EPI_ISL_613437, EPI_ISL_613438, EPI_ISL_613439, EPI_ISL_613440, EPI_ISL_613441, EPI_ISL_613442, EPI_ISL_613443, EPI_ISL_613444, EPI_ISL_613445, EPI_ISL_613446, EPI_ISL_613447, EPI_ISL_613448, EPI_ISL_613449, EPI_ISL_613450, EPI_ISL_613451, EPI_ISL_613452, EPI_ISL_613453, EPI_ISL_613454, EPI_ISL_613455, EPI_ISL_613456			
see above	Institut Pasteur de la Guadeloupe	Institut Pasteur de la Guadeloupe	Angela Brisebarre; Antoine Talarmin; Camille Capel; Cherina Fleming; Etienne Simon-Lorière; Marion Barbet; Maud Vanpeene; Méline Bizard; Radjin Steingrover; Stéphanie Guyomard; Sylvie Behillil; Sylvie van der Werf; Sébastien Breurec; Vincent Enouf
EPI_ISL_508884	Institut des Agents Infectieux (IAI), Hospices Civils de Lyon	CNR Virus des Infections Respiratoires - France SUD	Alexandre Gaymard; Antonin Bal; Bruno Lina; Carine Moustaud; Florence Morfin-Sherpa; Gregory Destras; Gwendolyne Burfin; Laurence Josset; Martine Valette; Maude Bouscambert-Duchamp; Raphaelle Lamy; Solenne Brun
EPI_ISL_602465, EPI_ISL_602500	Institute for Virology, University Hospital Essen	Center of Medical Microbiology, Virology, and Hospital Hygiene, University of Duesseldorf	Alexander Dilthey; Andreas Walker; Daniel Strelow; Jessica Nicolai; Jörg Timm; Klaus Pfeffer; Lisanna Hülse; Malte Kohns Vasconcelos; Maximilian Damagnez; Nadine Lübke; Olympia E. Anastasiou; Tobias Wienemann; Torsten Houwaart; Ulf Dittmer
EPI_ISL_723101	Institute of Medical Genetics and Applied Genomics	Institute of Medical Genetics and Applied Genomics	Angel Angelov; Caspar Gross; Daniela Bezdan; Michael Bitzer; Michael Sonnabend; Michaela Pogoda; Nicolas Casadei; Siri Göpel; Stephan Ossowski; Thomas Iftner; Tina Ganzenmüller
EPI_ISL_590835	Institute of Medical Virology, University of Zurich	Institute of Medical Virology, University of Zurich	Alexandra Trkola; Benjamin G. Hale; Idoia Busnadiego; Irene Abela; Marie O. Pohl; Maryam Zaheri; Michael Huber; Silke Stertz; Stefan Schmutz; Verena Kufner
EPI_ISL_422563, EPI_ISL_422564, EPI_ISL_422565	Institute of Microbiology Universidad San Francisco de Quito	Institute of Microbiology Universidad San Francisco de Quito	Alejandra Ramones; Belen Prado-Vivar; Bernardo Gutierrez; Edison Ligña; Francisco Mora; Franklin Espinoza; Gabriel Trueba; Jorge Reyes; Juan Gaviria; Juan Jose Guadalupe; Michelle Grunauer; Patricio Rojas-Silva; Paul Cardenas; Sully Marquez; Veronica Barragan
EPI_ISL_635251, EPI_ISL_635260	Institute of Microbiology and Immunology, Faculty of Medicine, University of Ljubljana	Institute of Microbiology and Immunology, Faculty of Medicine, University of Ljubljana	Mario Poljak; Miša Korva; Samo Zakotnik; Tatjana Avši - Županc; Tomaž Mark Zorec
EPI_ISL_548944	Institute of Microbiology, University of Veterinary and Animal sciences	Institute of Microbiology, University of Veterinary and Animal sciences	Ali; Altaf, I.; Anwar, M.; Ashraf; Asif, A.; Attique; Awan; Aziz; Bhatti; Cheema; Fazal, S.; Hassan, S.; Khan; Khan, N.; M.A.; M.B.; M.M.; M.S.; M.T.; M.U.; M.W.; M.Z.; Mehmood, A.; Mukhtar, N.; N.A.; Nawaz, M.; Rafique, S.; Rana; Raza, S.; S.Q.; S.Z.; Sardar, N.; Sarwar, H.; Shabbir; Shah; Tahir, Z.; Yaqub, T.; Younis
EPI_ISL_417482, EPI_ISL_477014, EPI_ISL_477015, EPI_ISL_477016, EPI_ISL_486842, EPI_ISL_486843, EPI_ISL_486844, EPI_ISL_486845, EPI_ISL_486846, EPI_ISL_486847, EPI_ISL_486848, EPI_ISL_486849, EPI_ISL_486850, EPI_ISL_486851, EPI_ISL_491932, EPI_ISL_491936, EPI_ISL_491937, EPI_ISL_491938, EPI_ISL_491939, EPI_ISL_491940, EPI_ISL_516648, EPI_ISL_516649, EPI_ISL_516650, EPI_ISL_516651, EPI_ISL_516652, EPI_ISL_525430, EPI_ISL_525431, EPI_ISL_525432, EPI_ISL_525433, EPI_ISL_525434, EPI_ISL_525435, EPI_ISL_525436, EPI_ISL_525437, EPI_ISL_525438, EPI_ISL_527809, EPI_ISL_527810, EPI_ISL_527811, EPI_ISL_527812, EPI_ISL_527813, EPI_ISL_527814, EPI_ISL_527815, EPI_ISL_527816, EPI_ISL_527817, EPI_ISL_539785, EPI_ISL_539786, EPI_ISL_539787, EPI_ISL_539788, EPI_ISL_539789, EPI_ISL_539790, EPI_ISL_539791, EPI_ISL_539792, EPI_ISL_539793, EPI_ISL_660529, EPI_ISL_660530, EPI_ISL_660531, EPI_ISL_660532, EPI_ISL_660533, EPI_ISL_660534, EPI_ISL_660535, EPI_ISL_660536, EPI_ISL_660537, EPI_ISL_660538, EPI_ISL_660539, EPI_ISL_697783, EPI_ISL_697784, EPI_ISL_697785, EPI_ISL_697786, EPI_ISL_697787, EPI_ISL_697788, EPI_ISL_697789, EPI_ISL_697790, EPI_ISL_697791, EPI_ISL_697792, EPI_ISL_697793, EPI_ISL_697794, EPI_ISL_697795, EPI_ISL_697798, EPI_ISL_697799, EPI_ISL_697800, EPI_ISL_728202, EPI_ISL_728203, EPI_ISL_728204, EPI_ISL_728205, EPI_ISL_803098, EPI_ISL_824284, EPI_ISL_824285, EPI_ISL_824286, EPI_ISL_824287, EPI_ISL_824288, EPI_ISL_824289, EPI_ISL_824290, EPI_ISL_824291, EPI_ISL_824292, EPI_ISL_824293, EPI_ISL_824294			
see above	Institute of Microbiology, Universidad San Francisco de Quito	Institute of Microbiology, Universidad San Francisco de Quito	Alejandra Ramones; Alexandra Tino; Andrea Cunguan; Andrea Macias; Anita Garcia; Belen Prado-Vivar; Belén Prado-Vivar; Bernardo Gutierrez; Bernardo Gutiérrez; Carla Torres; Carlos Guerrero; Carlos Mena; Carlos Tobar; Carolina Pacheco; Damaris Zandoya; DamarisSandoya; Dayron Brossard; Diana Zambrano; Diego Egas; Eddy Chavez; Edison Chavez; Edison Ligña; Edy Quizhpe; Eulalia Pazmiño; Fausto Maldonado; Francisco Mora; Francisco Rodriguez; Franklin Espinoza; Freddy Iza; Fredy Loor; Gabriel Trueba; Geovanny Carzola; Hermelinda Paguay; Jonathan Araujo; Jorge Luis Velez; Jorge Montaño; Jorge Reyes; Juan Gaviria; Juan Jose Guadalupe; Juan José Guadalupe; Juan Zuñiga; Karina Barragan; Katalina Pacheco; Khurram Mahbbob; Ligia Briceño; Manuel Jaramillo; Manuel Jibaja; Maureen Mosquera; Michelle Grunauer; Milton Tobar; Monica Becerra-Wong; Nabih Dahik; Patricio Reyes; Patricio Rojas-Silva; Paul Cardenas; Paúl Cárdenas; Prado-Vivar; Rene Bracho; Rosario Erazo; Stalin Castillo; Stephanie Arregui; Sully Marquez; Sully Márquez; Tania Guayasamin; Veronica Barragan; Verónica Barragán; Yomara Napa

EPI_ISL_508407	Institute of Post Graduate Medical Education & Research	National Institute of Biomedical Genomics	Arindam Maitra; Aritra Biswas; Jayeeta Haldar; Monimoy Banerjee; Raja Ray; Saumitra Das
EPI_ISL_672702, EPI_ISL_672703, EPI_ISL_672704, EPI_ISL_672705, EPI_ISL_672706, EPI_ISL_672710, EPI_ISL_672711, EPI_ISL_672713, EPI_ISL_672717, EPI_ISL_672718, EPI_ISL_672719, EPI_ISL_672720, EPI_ISL_672721, EPI_ISL_672748, EPI_ISL_672750, EPI_ISL_672751			
see above	Institute of Tropical Medicine at the University of São Paulo (IMT-USP)	Laboratório de Parasitologia Médica - Instituto de Medicina Tropical - Universidade de São Paulo	Brazil-UK Centre for Arbovirus Discovery Diagnosis Genomics and Epidemiology (CADDE) Genomic Network - Instituto de Medicina Tropical
EPI_ISL_755640, EPI_ISL_755642, EPI_ISL_755643, EPI_ISL_755651, EPI_ISL_755653, EPI_ISL_755654, EPI_ISL_776750, EPI_ISL_776751, EPI_ISL_776752, EPI_ISL_776753, EPI_ISL_776754, EPI_ISL_776755, EPI_ISL_776756, EPI_ISL_776759, EPI_ISL_776760, EPI_ISL_776761, EPI_ISL_776762, EPI_ISL_776763, EPI_ISL_792101, EPI_ISL_792102, EPI_ISL_792104, EPI_ISL_792105, EPI_ISL_792106, EPI_ISL_792107, EPI_ISL_792108, EPI_ISL_792109, EPI_ISL_792110, EPI_ISL_792111, EPI_ISL_792112, EPI_ISL_792113, EPI_ISL_792114, EPI_ISL_833152, EPI_ISL_833153, EPI_ISL_833154, EPI_ISL_833155, EPI_ISL_833159, EPI_ISL_833161			
see above	Instituto Adolfo Lutz - Central	Instituto Adolfo Lutz, Interdiciplinary Procedures Center, Strategic Laboratory	Claudia Regina Gonçalves; Claudio Tavares Sacchi; Erica Valessa Ramos Gomes; Karoline Rodrigues Campos
EPI_ISL_776768	Instituto Adolfo Lutz - Regional de Aracatuba	Instituto Adolfo Lutz, Interdiciplinary Procedures Center, Strategic Laboratory	Claudia Regina Gonçalves; Claudio Tavares Sacchi; Erica Valessa Ramos Gomes; Karoline Rodrigues Campos
EPI_ISL_755655	Instituto Adolfo Lutz - Regional de Campinas	Instituto Adolfo Lutz, Interdiciplinary Procedures Center, Strategic Laboratory	Claudia Regina Gonçalves; Claudio Tavares Sacchi; Erica Valessa Ramos Gomes; Karoline Rodrigues Campos
EPI_ISL_776757, EPI_ISL_776758, EPI_ISL_776767, EPI_ISL_833163	Instituto Adolfo Lutz - Regional de Marilia	Instituto Adolfo Lutz, Interdiciplinary Procedures Center, Strategic Laboratory	Claudia Regina Gonçalves; Claudio Tavares Sacchi; Erica Valessa Ramos Gomes; Karoline Rodrigues Campos
EPI_ISL_735401, EPI_ISL_735402, EPI_ISL_735403, EPI_ISL_735404, EPI_ISL_735410	Instituto Adolfo Lutz - Regional de Rio Claro	Instituto Adolfo Lutz, Interdiciplinary Procedures Center, Strategic Laboratory	Claudia Regina Gonçalves; Claudio Tavares Sacchi; Erica Valessa Ramos Gomes; Karoline Rodrigues Campos
EPI_ISL_755641, EPI_ISL_755646, EPI_ISL_755647, EPI_ISL_755649, EPI_ISL_776764, EPI_ISL_776765, EPI_ISL_776766, EPI_ISL_776769, EPI_ISL_792103, EPI_ISL_833157, EPI_ISL_833158, EPI_ISL_833160			
see above	Instituto Adolfo Lutz - Regional de Santo Andre	Instituto Adolfo Lutz, Interdiciplinary Procedures Center, Strategic Laboratory	Claudia Regina Gonçalves; Claudio Tavares Sacchi; Erica Valessa Ramos Gomes; Karoline Rodrigues Campos
EPI_ISL_735400, EPI_ISL_735427, EPI_ISL_735430	Instituto Adolfo Lutz - Regional de Santos	Instituto Adolfo Lutz, Interdiciplinary Procedures Center, Strategic Laboratory	Claudia Regina Gonçalves; Claudio Tavares Sacchi; Erica Valessa Ramos Gomes; Karoline Rodrigues Campos
EPI_ISL_833156	Instituto Adolfo Lutz - Regional de Sorocaba	Instituto Adolfo Lutz, Interdiciplinary Procedures Center, Strategic Laboratory	Claudia Regina Gonçalves; Claudio Tavares Sacchi; Erica Valessa Ramos Gomes; Karoline Rodrigues Campos
EPI_ISL_755648, EPI_ISL_755650, EPI_ISL_792115, EPI_ISL_792116	Instituto Adolfo Lutz - Regional de Taubate	Instituto Adolfo Lutz, Interdiciplinary Procedures Center, Strategic Laboratory	Claudia Regina Gonçalves; Claudio Tavares Sacchi; Erica Valessa Ramos Gomes; Karoline Rodrigues Campos
EPI_ISL_426364	Instituto Nacional de Ciencias Medicas y Nutricion Salvador Zubiran	Instituto Nacional de Ciencias Medicas y Nutricion	Adnan Araiza Rodríguez; Alejandro Sánchez; Alfredo Ponce de León Garduño; Blanca Taboada; Carlos F. Arias; Carolina González Torres; Celia Boukadida; Cesar Raúl González Bonilla; Concepción Grajales Muñiz; Edgar Mendieta Condado; Eduardo Becerril Vargas; Fabiola Garcés Ayala; Fernando Ledesma Barrientos; Francisco Javier Gaytán Cervantes; Francisco Pulido; Gisela Barrera Badillo; Gloria Vázquez; Guillermo M. Ruiz-Palacios; Irma López Martínez; Joel Armando Vázquez Pérez; José Arturo Martínez Orozco; José Ernesto Ramírez González; José Esteban Muñoz Medina; Lucia Hernández Rivas; Luis Alberto García Andrade; Mario Mújica Sánchez; Pavel Isa; Pilar Ramos Cervantes; Ricardo Grande; Santiago Avila Ríos; Victor Hugo Borja Aburto; Violeta Ibarra Gonzalez
EPI_ISL_426361, EPI_ISL_426362, EPI_ISL_426363, EPI_ISL_426365	Instituto Nacional de Ciencias Medicas y Nutricion Salvador Zubiran	Instituto Nacional de Ciencias Medicas y Nutricion Salvador Zubiran	Adnan Araiza Rodríguez; Alejandro Sánchez; Alfredo Ponce de León Garduño; Blanca Taboada; Carlos F. Arias; Carolina González Torres; Celia Boukadida; Cesar Raúl González Bonilla; Concepción Grajales Muñiz; Edgar Mendieta Condado; Eduardo Becerril Vargas; Fabiola Garcés Ayala; Fernando Ledesma Barrientos; Francisco Javier Gaytán Cervantes; Francisco Pulido; Gisela Barrera Badillo; Gloria Vázquez; Guillermo M. Ruiz-Palacios; Irma López Martínez; Joel Armando Vázquez Pérez; José Arturo Martínez Orozco; José Ernesto Ramírez González; José Esteban Muñoz Medina; Lucia Hernández Rivas; Luis Alberto García Andrade; Mario Mújica Sánchez; Pavel Isa; Pilar Ramos Cervantes; Ricardo Grande; Santiago Avila Ríos; Victor Hugo Borja Aburto; Violeta Ibarra Gonzalez
EPI_ISL_424345, EPI_ISL_424348, EPI_ISL_424626, EPI_ISL_424627	Instituto Nacional de Enfermedades Respiratorias	Instituto Nacional de Enfermedades Respiratorias	Adnan Araiza Rodríguez; Alejandro Sánchez; Alfredo Ponce de León Garduño; Blanca Taboada; Carlos F. Arias.; Carolina González Torres; Celia Boukadida; Cesar Raúl González Bonilla; Concepción Grajales Muñiz; Edgar Mendieta Condado; Eduardo Becerril Vargas; Fabiola Garcés Ayala; Fernando Ledesma Barrientos; Francisco Javier Gaytán Cervantes; Francisco Pulido; Gisela Barrera Badillo; Gloria Vázquez; Guillermo M. Ruiz-Palacios; Irma López Martínez; Joel Armando Vázquez Pérez; Jorge Salas Hernández; José Arturo Martínez Orozco; José Ernesto Ramírez González; José Esteban Muñoz Medina; Lucia Hernández Rivas; Luis Alberto García Andrade; Mario Mújica Sánchez; Pavel Isa; Pilar Ramos Cervantes; Ricardo Grande; Santiago Avila Ríos; Victor Hugo Borja Aburto; Violeta Ibarra Gonzalez
EPI_ISL_412972	Instituto Nacional de Enfermedades Respiratorias	Instituto de Diagnostico y Referencia Epidemiologicos (INDRE)	Araiza-Rodriguez Adnan; Arias Carlos; Barrera-Badillo Gisela; Boukadida Celia; Garces-Ayala Fabiola; Hernandez-Rivas Lucia; Isa Pavel; Lopez Susana; Lopez-Martinez Irma; Martinez Arturo; Mendieta-Condado Edgar; Munoz-Medina Esteban; Ramirez-Gonzalez Ernesto; Rodriguez-Maldonado Abril; Sanchez Alejandro; Taboada Blanca; Vazquez-Perez Joel; Wong-Arambula Claudia
EPI_ISL_837600, EPI_ISL_837601, EPI_ISL_837602, EPI_ISL_837603, EPI_ISL_837604, EPI_ISL_837605, EPI_ISL_837606, EPI_ISL_837607, EPI_ISL_837608, EPI_ISL_837609, EPI_ISL_837610, EPI_ISL_837611, EPI_ISL_837612, EPI_ISL_837613, EPI_ISL_837614, EPI_ISL_837615, EPI_ISL_837616, EPI_ISL_837617, EPI_ISL_837618, EPI_ISL_837619, EPI_ISL_837620, EPI_ISL_837621, EPI_ISL_837622, EPI_ISL_837623, EPI_ISL_837624, EPI_ISL_837625, EPI_ISL_837626, EPI_ISL_837627, EPI_ISL_837628, EPI_ISL_837629, EPI_ISL_837630, EPI_ISL_837631, EPI_ISL_837632, EPI_ISL_837633, EPI_ISL_837634, EPI_ISL_837635, EPI_ISL_837636, EPI_ISL_837637, EPI_ISL_837638, EPI_ISL_837639, EPI_ISL_837640, EPI_ISL_837641, EPI_ISL_837642, EPI_ISL_837643, EPI_ISL_837644, EPI_ISL_837645, EPI_ISL_837646, EPI_ISL_837647, EPI_ISL_837648, EPI_ISL_837649, EPI_ISL_837650, EPI_ISL_837651, EPI_ISL_837652, EPI_ISL_837653, EPI_ISL_837654, EPI_ISL_837655, EPI_ISL_837656, EPI_ISL_837657, EPI_ISL_837658, EPI_ISL_837659, EPI_ISL_837660, EPI_ISL_837661, EPI_ISL_837662, EPI_ISL_837663, EPI_ISL_837664, EPI_ISL_837665, EPI_ISL_837666, EPI_ISL_837667, EPI_ISL_837668, EPI_ISL_837669, EPI_ISL_837670, EPI_ISL_837671, EPI_ISL_837672, EPI_ISL_837673, EPI_ISL_837674, EPI_ISL_837675, EPI_ISL_837676, EPI_ISL_837677, EPI_ISL_837678, EPI_ISL_837679, EPI_ISL_837680, EPI_ISL_837681, EPI_ISL_837682, EPI_ISL_837683, EPI_ISL_837684, EPI_ISL_837685, EPI_ISL_837686, EPI_ISL_837687, EPI_ISL_837688, EPI_ISL_837689, EPI_ISL_837690, EPI_ISL_837691, EPI_ISL_837692, EPI_ISL_837693, EPI_ISL_837694, EPI_ISL_837695, EPI_ISL_837696, EPI_ISL_837697, EPI_ISL_837698, EPI_ISL_837699, EPI_ISL_837700, EPI_ISL_837701, EPI_ISL_837702, EPI_ISL_837703, EPI_ISL_837704, EPI_ISL_837705, EPI_ISL_837706, EPI_ISL_837707, EPI_ISL_837708, EPI_ISL_837709, EPI_ISL_837710, EPI_ISL_837711, EPI_ISL_837712, EPI_ISL_837713, EPI_ISL_837714, EPI_ISL_837715, EPI_ISL_837716, EPI_ISL_837717, EPI_ISL_837718, EPI_ISL_837719, EPI_ISL_837720, EPI_ISL_837721, EPI_ISL_837722, EPI_ISL_837723, EPI_ISL_837724, EPI_ISL_837725, EPI_ISL_837726, EPI_ISL_837727, EPI_ISL_837728, EPI_ISL_837729, EPI_ISL_837730, EPI_ISL_837731, EPI_ISL_837732, EPI_ISL_837733, EPI_ISL_837734, EPI_ISL_837735, EPI_ISL_837736, EPI_ISL_837737, EPI_ISL_837738, EPI_ISL_837739, EPI_ISL_837740, EPI_ISL_837741, EPI_ISL_837742, EPI_ISL_837743, EPI_ISL_837744, EPI_ISL_837745, EPI_ISL_837746, EPI_ISL_837747, EPI_ISL_837748, EPI_ISL_837749, EPI_ISL_837750, EPI_ISL_837751, EPI_ISL_837752, EPI_ISL_837753, EPI_ISL_837754, EPI_ISL_837755, EPI_ISL_837756, EPI_ISL_837757, EPI_ISL_837758, EPI_ISL_837759, EPI_ISL_837760, EPI_ISL_837761, EPI_ISL_837762, EPI_ISL_837763, EPI_ISL_837764, EPI_ISL_837765, EPI_ISL_837766, EPI_ISL_837767, EPI_ISL_837768, EPI_ISL_837769, EPI_ISL_837770, EPI_ISL_837771, EPI_ISL_837772, EPI_ISL_837773, EPI_ISL_837774, EPI_ISL_837775, EPI_ISL_837776, EPI_ISL_837777, EPI_ISL_837778, EPI_ISL_837779, EPI_ISL_837780, EPI_ISL_837781, EPI_ISL_837782, EPI_ISL_837783, EPI_ISL_837784, EPI_ISL_837785, EPI_ISL_837786, EPI_ISL_837787, EPI_ISL_837788, EPI_ISL_837789, EPI_ISL_837790, EPI_ISL_837791, EPI_ISL_837792, EPI_ISL_837793, EPI_ISL_837794, EPI_ISL_837795, EPI_ISL_837796, EPI_ISL_837797, EPI_ISL_837798, EPI_ISL_837799, EPI_ISL_837800, EPI_ISL_837801, EPI_ISL_837802, EPI_ISL_837803, EPI_ISL_837804, EPI_ISL_837805, EPI_ISL_837806, EPI_ISL_837807, EPI_ISL_837808, EPI_ISL_837809, EPI_ISL_837810, EPI_ISL_837811, EPI_ISL_837812, EPI_ISL_837813, EPI_ISL_837814, EPI_ISL_843191, EPI_ISL_843192			
see above	Instituto Nacional de Enfermedades Respiratorias (INER)	Instituto Nacional de Enfermedades Respiratorias (INER)	Alejandra Hernández-Terán; Alma Rincón-Rubio; Celia Boukadida; Edgar Sevilla-Reyes; Eduardo Becerril-Vargas; Fidencio Mejía-Nepomuceno; Hector Esteban Paz-Juárez; Joel Armando Vázquez-Pérez; Jorge Salas-Hernández; José Arturo Martínez-Orozco; Margarita Matías-Florentino; Mario Mújica-Sánchez; Olivia Briceño; Santiago Ávila-Ríos
EPI_ISL_792351, EPI_ISL_792352, EPI_ISL_792353	Instituto Nacional de Epidemiología Dr. Jara	Área de Secuenciación del Laboratorio de Virología del Hospital de Niños Dr. Ricardo Gutierrez on behalf of 'Proyecto Argentino Interinstitucional de genomica de SARS-CoV-2' (PAIS Consortium)	CJ; Cimmino; Goya; I; LE; Lusso; MI; MS; Nabaes Jodar; Natale; O; Pagano; S; Uez; Valinotto; Viegas, M.
EPI_ISL_574294	Instituto Nacional de Investigacion en Salud Pública	Instituto Nacional de Investigación en Salud Pública	Alberto Orlando Narvaez; Alfredo Bruno; Andrés Carrasco; Denisse Portugal; Doménica de Mora Coloma; Leandro Patino Patino; Manuel Gonzalez; Orson Mestanza
EPI_ISL_574292	Instituto Nacional de Investigación en Salud Pública	Instituto Nacional de Investigación en Salud Pública	Alberto Orlando Narvaez.; Alfredo Bruno Caicedo; Andrés Carrasco; Denisse Portugal; Domenica de Mora Coloma; Leandro Patino Patino; Manuel Gonzalez; Orson Mestanza
EPI_ISL_574293	Instituto Nacional de Investigación en Salud Pública	Instituto Nacional de Investigación en Salud Publica	Alberto Orlando Narvaez.; Alfredo Bruno Caicedo; Andrés Carrasco; Denisse Portugal; Doménica de Mora Coloma; Leandro Patino Patino; Manuel Gonzalez; Orson Mestanza
EPI_ISL_491944, EPI_ISL_491945, EPI_ISL_491946, EPI_ISL_491947, EPI_ISL_491948, EPI_ISL_491949, EPI_ISL_491950, EPI_ISL_491951, EPI_ISL_491952, EPI_ISL_491953, EPI_ISL_491954			

see above	Instituto Nacional de Investigación en Salud Pública - INSPI	INSPI - Charité	Alberto Orlando; Alexandra Usiña; Alfredo Bruno Caicedo; Andres Moreira-Soto; Anna-Lena Sander; Denisses Portugal; Domenica de Mora Coloma; Jan Felix Drexler; Juan Carlos Zeballos; Manuel Gonzalez; Maritza Olmedo; Nina Krause; Silvia Salgado
EPI_ISL_522873, EPI_ISL_522874, EPI_ISL_522875, EPI_ISL_522876, EPI_ISL_522877, EPI_ISL_522878	Instituto Nacional de Medicina Genomica	Instituto Nacional de Medicina Genomica	Cedro-Tanda A; Cisneros-Villanueva M; Herrera-Montalvo LA; Hidalgo-Miranda A; Hurtado-Cordova E; Mendoza-Vargas A; Peñaloza-Figueroa F; Reyes-Grajeda JP
EPI_ISL_522872, EPI_ISL_522879, EPI_ISL_522880, EPI_ISL_522940, EPI_ISL_522941, EPI_ISL_522942, EPI_ISL_522978, EPI_ISL_522979, EPI_ISL_522980, EPI_ISL_522981, EPI_ISL_522982, EPI_ISL_522983, EPI_ISL_522984, EPI_ISL_522985, EPI_ISL_522986, EPI_ISL_523500			
see above	Instituto Nacional de Medicina Genómica	Instituto Nacional de Medicina Genómica	Cedro-Tanda A; Cisneros-Villanueva M; Herrera-Montalvo LA; Hidalgo-Miranda A; Hurtado-Cordova E; Mendoza-Vargas A; Peñaloza-Figueroa F; Reyes-Grajeda JP
EPI_ISL_418262	Instituto Nacional de Salud	Instituto Nacional de Salud Universidad Cooperativa de Colombia Instituto Alexander von Humboldt Imperial College-London London School of Hygiene & Tropical Medicine	Astrid C. Flórez; Carlos Franco-Muñoz; Christian Julian VillabonaArenas; Diana Marcela Walteros-Acero; Diego A. Álvarez-Díaz; Erika Ospitia; Gloria Puerto; Jose A. Usme-Ciro; Juliana Barbosa; Katherine Laiton-Donato; Liz Villabona-Arenas; Luz Dary Rodriguez; Mailyn A.Gonzalez; Marcela Mercado-Reyes; Martha Lucia Ospina Martinez; Nicolas D. Franco-Sierra; Nuno Rodrigues Faria; Sergio Gomez Rangel; Sussy Echeverria; Zulma M. Cucunubá
EPI_ISL_536477, EPI_ISL_536478, EPI_ISL_536479, EPI_ISL_536480, EPI_ISL_536481, EPI_ISL_536482, EPI_ISL_536483, EPI_ISL_536484, EPI_ISL_536485, EPI_ISL_536486, EPI_ISL_536487, EPI_ISL_536488, EPI_ISL_536489, EPI_ISL_536490, EPI_ISL_536491, EPI_ISL_536492, EPI_ISL_536493, EPI_ISL_536494, EPI_ISL_536495, EPI_ISL_536496, EPI_ISL_536497, EPI_ISL_536498, EPI_ISL_536499, EPI_ISL_536500, EPI_ISL_536501, EPI_ISL_536502, EPI_ISL_536503, EPI_ISL_536504, EPI_ISL_536505, EPI_ISL_536506, EPI_ISL_536507, EPI_ISL_536508, EPI_ISL_536509, EPI_ISL_536510, EPI_ISL_536511, EPI_ISL_536512, EPI_ISL_536513, EPI_ISL_536514, EPI_ISL_536515, EPI_ISL_536516, EPI_ISL_536517, EPI_ISL_536518, EPI_ISL_536519, EPI_ISL_536520, EPI_ISL_536521, EPI_ISL_536522, EPI_ISL_536523, EPI_ISL_536524, EPI_ISL_536525, EPI_ISL_536526, EPI_ISL_536527, EPI_ISL_536528, EPI_ISL_536529, EPI_ISL_536530, EPI_ISL_536531, EPI_ISL_536532, EPI_ISL_536533, EPI_ISL_536534, EPI_ISL_536535, EPI_ISL_536536, EPI_ISL_536537, EPI_ISL_536538, EPI_ISL_536539, EPI_ISL_536540, EPI_ISL_536541, EPI_ISL_536542, EPI_ISL_536543, EPI_ISL_536544, EPI_ISL_536545, EPI_ISL_536546, EPI_ISL_536547, EPI_ISL_536548, EPI_ISL_536549, EPI_ISL_536550, EPI_ISL_536551, EPI_ISL_536552, EPI_ISL_536553, EPI_ISL_536554, EPI_ISL_536555, EPI_ISL_536556, EPI_ISL_536557, EPI_ISL_536558, EPI_ISL_536559, EPI_ISL_536560, EPI_ISL_536561, EPI_ISL_536562, EPI_ISL_536563, EPI_ISL_536564, EPI_ISL_536565			
see above	Instituto Nacional de Salud	Laboratorio de Infecciones Respiratorias Agudas	David Tarazona; Dennis Carhuaricra; Eduardo Juscamayta Lopez; Faviola Valdivia Guerrero; Lenin Maturrano Hernandez; Nancy Rojas Serrano; Ronnie Gavilan Chavez
EPI_ISL_791087, EPI_ISL_791088, EPI_ISL_791093, EPI_ISL_791096	Instituto Nacional de Salud - Unidad de Secuenciación y Análisis Genómico	Instituto Nacional de Salud - Dirección de Investigación en Salud Pública	Carlos Franco-Muñoz; Diego A. Álvarez-Díaz; Diego Andrés Prada; Gerardo Santamaría; Jonathan Reales; Julian Naizaque; Katherine Laiton-Donato; Magdalena Wiesner; Marcela Mercado-Reyes; Maria T. Herrera; Martha Lucia Ospina Martinez; Mauricio Pacheco-Montealegre; Paola Muñoz-Laiton; Sheryl Corchuelo
EPI_ISL_456116, EPI_ISL_456117, EPI_ISL_456118, EPI_ISL_456119, EPI_ISL_456120, EPI_ISL_456122, EPI_ISL_456123, EPI_ISL_456124, EPI_ISL_456125, EPI_ISL_456126, EPI_ISL_456127, EPI_ISL_456128, EPI_ISL_456131, EPI_ISL_456134, EPI_ISL_456135, EPI_ISL_456136, EPI_ISL_456138, EPI_ISL_456139, EPI_ISL_456143, EPI_ISL_456144, EPI_ISL_456145, EPI_ISL_456146, EPI_ISL_456147, EPI_ISL_456148, EPI_ISL_456149, EPI_ISL_456150, EPI_ISL_456151, EPI_ISL_456152, EPI_ISL_456153, EPI_ISL_456154, EPI_ISL_456155, EPI_ISL_456156			
see above	Instituto Nacional de Salud - Unidad de Secuenciación y Análisis Genómico	Instituto Nacional de Salud, Universidad Cooperativa de Colombia, Instituto Alexander von Humboldt, Imperial College-London, London School of Hygiene & Tropical Medicine	Astrid C. Flórez; Carlos Franco-Muñoz; Christian Julian VillabonaArenas; Diana Marcela Walteros-Acero; Diego A. Álvarez-Díaz; Erika Ospitia; Gloria Puerto; Jose A. Usme-Ciro; Juliana Barbosa; Katherine Laiton-Donato; Liz Villabona-Arenas; Luz Dary Rodriguez; Mailyn A.Gonzalez; Marcela Mercado-Reyes.; Martha Lucia Ospina Martinez; Nicolas D. Franco-Sierra; Sergio Gomez-Rangel; Sussy Echeverria; Zulma M. Cucunubá
EPI_ISL_447755, EPI_ISL_447756, EPI_ISL_447757, EPI_ISL_447758, EPI_ISL_447759, EPI_ISL_447760, EPI_ISL_447761, EPI_ISL_447762, EPI_ISL_447763, EPI_ISL_447765, EPI_ISL_447766, EPI_ISL_447767, EPI_ISL_447768, EPI_ISL_447769, EPI_ISL_447771, EPI_ISL_447774, EPI_ISL_447775, EPI_ISL_447777, EPI_ISL_447778, EPI_ISL_447779, EPI_ISL_447780, EPI_ISL_447781, EPI_ISL_447782, EPI_ISL_447784, EPI_ISL_447785, EPI_ISL_447786, EPI_ISL_447789, EPI_ISL_447790, EPI_ISL_447791, EPI_ISL_447792, EPI_ISL_447793, EPI_ISL_447794, EPI_ISL_447795, EPI_ISL_447796, EPI_ISL_447797, EPI_ISL_447798, EPI_ISL_447800, EPI_ISL_447802, EPI_ISL_447803, EPI_ISL_447804, EPI_ISL_447805, EPI_ISL_447806, EPI_ISL_447807, EPI_ISL_447808, EPI_ISL_447809, EPI_ISL_447810, EPI_ISL_447811, EPI_ISL_447812, EPI_ISL_447813, EPI_ISL_447814, EPI_ISL_447816, EPI_ISL_447817			
see above	Instituto Nacional de Salud, Bogotá, Colombia	Grupo de Investigaciones Microbiológicas-UR (GIMUR), Departamento de Biología, Facultad de Ciencias Naturales, Universidad del Rosario, Bogotá, Colombia Instituto Nacional de Salud, Bogotá, Colombia Icahn School of Medicine at Mount Sinai, New York, USA	Adriana Castillo; Alberto Paniz-Mondolfi; Ana S. Gonzalez-Reiche; Angelica Rico; Aníbal A. Teherán; Carolina Florez; Carolina Hernandez; David Martínez; Emilia Mia Sordillo; Esther C. Barros; Harm van Bakel; Jesús E. Jaimes; Juan David Ramírez; Laura Vega; Lisseth Pardo; Marina Muñoz; Martha L. Ospina; Matthew M. Hernandez; Nathalia Ballesteros; Sergio Castañeda; Sergio Gomez; Viviana Simon
EPI_ISL_497736, EPI_ISL_497738, EPI_ISL_497744, EPI_ISL_497745, EPI_ISL_498152, EPI_ISL_498153, EPI_ISL_498154, EPI_ISL_498155, EPI_ISL_498156, EPI_ISL_498157, EPI_ISL_498158, EPI_ISL_498159, EPI_ISL_498160, EPI_ISL_498161, EPI_ISL_498162, EPI_ISL_498163, EPI_ISL_498164, EPI_ISL_498165, EPI_ISL_498166, EPI_ISL_498167, EPI_ISL_498168, EPI_ISL_498169, EPI_ISL_498170, EPI_ISL_526932, EPI_ISL_526933, EPI_ISL_526934, EPI_ISL_526949, EPI_ISL_526950, EPI_ISL_526951, EPI_ISL_526953, EPI_ISL_526955, EPI_ISL_526956, EPI_ISL_526958, EPI_ISL_526959, EPI_ISL_526963, EPI_ISL_526964, EPI_ISL_526965, EPI_ISL_526967, EPI_ISL_526969, EPI_ISL_526971, EPI_ISL_653745, EPI_ISL_653746, EPI_ISL_653747, EPI_ISL_653749, EPI_ISL_653750, EPI_ISL_653751, EPI_ISL_653752, EPI_ISL_653754, EPI_ISL_653755, EPI_ISL_653756, EPI_ISL_653757, EPI_ISL_653758, EPI_ISL_653759, EPI_ISL_653761, EPI_ISL_653762, EPI_ISL_739663, EPI_ISL_739671, EPI_ISL_739672, EPI_ISL_739673, EPI_ISL_739675, EPI_ISL_739680			
see above	Instituto Nacional de Salud, Bogotá, Colombia	Instituto Nacional de Salud, Bogotá, Colombia	Astrid C. Flórez; Carlos Andrés Durán; Carlos Franco-Muñoz; Carolina Ferro; Christian Julian VillabonaArenas; Diana Marcela Walteros-Acero; Diego A. Álvarez-Díaz; Diego Andrés Prada; Edmilson F. de Oliveira-Filho; Felix Betzler; Franklin Prieto; Jeadran Malagón-Rojas; Jonathan Reales; Jose A. Usme-Ciro; Katherine Laiton-Donato; Liz Villabona-Arenas; Magdalena Weisner; Marcela Mercado-Reyes; Martha Lucia Ospina Martinez; Mauricio Pacheco-Montealegre; Nicolas D. Franco-Sierra; Sheryl Corchuelo; Sussy Echeverria; Wendy K. Jo; Zulma M. Cucunubá
EPI_ISL_510956, EPI_ISL_511052, EPI_ISL_511074, EPI_ISL_511085, EPI_ISL_511094, EPI_ISL_511551, EPI_ISL_511729, EPI_ISL_693549			
see above	Instituto Nacional de Saude (INSA)	Instituto Nacional de Saude (INSA)	Borges et al
EPI_ISL_511211	Instituto Nacional de Saude (INSA) and Instituto Gulbenkian de Ciencia (IGC)	Instituto Nacional de Saude (INSA) and Instituto Gulbenkian de Ciencia (IGC)	Borges et al
EPI_ISL_427294, EPI_ISL_427295, EPI_ISL_427296, EPI_ISL_427297, EPI_ISL_427298, EPI_ISL_427299, EPI_ISL_427300, EPI_ISL_427301, EPI_ISL_427302, EPI_ISL_427303, EPI_ISL_427304			
see above	Instituto Oswaldo Cruz FIOCRUZ - Laboratory of Respiratory Viruses and Measles (LVRS)	Instituto Oswaldo Cruz FIOCRUZ - Laboratory of Respiratory Viruses and Measles (LVRS)	Aline Mattos; Braulia Caetano; Cristiana Garcia; Fernando Motta; Jonathan Lopes; Luciana Appolinario; Maria Ogrzewalska; Marilda Siqueira; Milene Miranda; Paola Resende; Priscila Born; Sunando Roy
EPI_ISL_426580	Instituto Sabin	Laboratory of Virology	Bergmann M Ribeiro; Fernando L Melo; Gustavo Barra; Ikaro A Andrade; Pedro G Mesquita; Tatsuya Nagata; Ticiane H Santa-Rita
EPI_ISL_492032, EPI_ISL_492033, EPI_ISL_492034, EPI_ISL_492035, EPI_ISL_492036, EPI_ISL_492037, EPI_ISL_492038, EPI_ISL_492039, EPI_ISL_492040, EPI_ISL_492041, EPI_ISL_492042, EPI_ISL_492043, EPI_ISL_492044, EPI_ISL_492045, EPI_ISL_492046, EPI_ISL_492047, EPI_ISL_492048			
see above	Instituto de Biologia do Exército	Laboratório Metabolismo Macromolecular FirminoTorres de Castro, Instituto de Biofísica Carlos Chagas Filho, Universidade Federal do Rio de Janeiro	Aline Rosa Vianna de Souza; Bianca Catarina Azevedo Cabral; Caleb GM Santos; Clarissa Damaso; Elizabeth Valentin; Marcio da Costa Cipitelli; Marcos Dornelas-Ribeiro; Nádia Vaez Gonçalves da Cruz; Rodrigo Soares de Moura Neto; Rosane Silva; Tatiana LS Nogueira; Virginia Sara Grancieri do Amaral
EPI_ISL_424673	Instituto de Diagnostico y Referencia Epidemiologicos	Instituto de Diagnostico y Referencia Epidemiologicos	Adnan Araiza Rodríguez; Alejandro Sánchez; Alfredo Ponce de León Garduño; Blanca Taboada; Carlos F. Arias; Carolina González Torres; Celia Boukadida; Cesar Raúl González Bonilla; Concepción Grajales Muñiz; Edgar Mendieta Condado; Eduardo Becerril Vargas; Fabiola Garcés Ayala; Fernando Ledesma Barrientos; Francisco Javier Gaytán Cervantes; Francisco Pulido; Gisela Barrera Badillo; Gloria Vázquez; Guillermo M. Ruiz-Palacios; Irma López Martínez; Joel Armando Vázquez Pérez; José Arturo Martínez Orozco; José Ernesto Ramírez González; José Esteban Muñoz Medina; Lucia Hernández Rivas; Luis Alberto García Andrade; Mario Mújica Sánchez; Pavel Isa; Pilar Ramos Cervantes; Ricardo Grande; Santiago Avila Ríos; Victor Hugo Borja Aburto; Violeta Ibarra Gonzalez
EPI_ISL_455432, EPI_ISL_455434, EPI_ISL_455435, EPI_ISL_455436, EPI_ISL_455437, EPI_ISL_455438, EPI_ISL_455439, EPI_ISL_455454, EPI_ISL_455455, EPI_ISL_455456, EPI_ISL_493334, EPI_ISL_493335, EPI_ISL_493336, EPI_ISL_493338, EPI_ISL_493339, EPI_ISL_493340, EPI_ISL_493341, EPI_ISL_493342, EPI_ISL_493343, EPI_ISL_493344, EPI_ISL_493346, EPI_ISL_493347, EPI_ISL_493348, EPI_ISL_493349, EPI_ISL_516608, EPI_ISL_516609, EPI_ISL_516610, EPI_ISL_516611, EPI_ISL_516613, EPI_ISL_516614, EPI_ISL_516615, EPI_ISL_516616, EPI_ISL_516617, EPI_ISL_516618, EPI_ISL_516619, EPI_ISL_516620, EPI_ISL_516622, EPI_ISL_516623, EPI_ISL_516624, EPI_ISL_516625, EPI_ISL_576257, EPI_ISL_576258, EPI_ISL_576259, EPI_ISL_576260, EPI_ISL_576261, EPI_ISL_576262, EPI_ISL_576263, EPI_ISL_576264, EPI_ISL_576265, EPI_ISL_576266, EPI_ISL_576267, EPI_ISL_576268, EPI_ISL_576269, EPI_ISL_576270, EPI_ISL_576271, EPI_ISL_576272, EPI_ISL_576273, EPI_ISL_576274, EPI_ISL_576275, EPI_ISL_576276, EPI_ISL_576277, EPI_ISL_576278, EPI_ISL_576279, EPI_ISL_658863, EPI_ISL_658865, EPI_ISL_658868, EPI_ISL_658870, EPI_ISL_658873, EPI_ISL_658875, EPI_ISL_658878, EPI_ISL_658880, EPI_ISL_658883, EPI_ISL_658886, EPI_ISL_658888, EPI_ISL_658891, EPI_ISL_658893, EPI_ISL_658896, EPI_ISL_658899, EPI_ISL_658901, EPI_ISL_658904			
see above	Instituto de Diagnostico y Referencia Epidemiologicos (INDRE)	Instituto de Diagnostico y Referencia Epidemiologicos (INDRE)	; Abril Rodriguez-Maldonado; Adnan Araiza-Rodriguez; Araiza-Rodriguez Adnan; Barrera-Badillo Gisela; Claudia Wong-Arambula; Dayanira Arellano-Suarez; Edgar Mendieta-Condado; Ernesto Ramirez-Gonzalez; Ernesto Ramirez-Gonzalez.; Fabiola Garces-Ayala; Garces-Ayala Fabiola; Garces-Ayala Fabiola. Ramirez-Gonzalez Ernesto; Garces-Ayala Fabiola. Taboada Ramírez Blanca. Ramirez-Gonzalez Ernesto; Garces-Ayala Fabiola. Taboada Ramirez Blanca. Ramirez-Gonzalez Ernesto; Garces-Ayala Fabiola. Taboada Ramírez Blanca. Ramirez-Gonzalez Ernesto; Gaudalupe Herrera-Ramirez; Gisela Barrera-Badillo; Gisela Barrera-Badillo.; Hernandez-Rivas Lucia; Irma Lopez-Martinez; Jazmin Galicia-Hernandez.; Linda Andarade-Sanchez; Lopez-Martinez Irma; Lopez-Martinez Irma.; Lucia Hernandez-Rivas; Marisol Galindo-Galindo; Mendieta-Condado Edgar; Natividad

			Cruz-Ortiz; Octavio Ruiz-Muñiz; Pamela Ramirez-Medina; Ramirez-Gonzalez Ernesto; Ramirez-Gonzalez Ernesto.; Rodriguez-Maldonado Abril; Roman Canul-Aguilar; Taboada Ramirez Blanca; Taboada Ramírez Blanca; Taboada Ramírez Blanca.; Taboada Ramírez Blanca. Ramirez-Gonzalez Ernesto; Tatiana Nunez-Garcia; Wong-Arambula Claudia; Wong-Arambula Claudia.
EPI_ISL_452139, EPI_ISL_452141	Instituto de Diagnostico y Referencia Epidemiologicos (INDRE)	Instituto de diagnóstico y Referencia Epidemiologicos (INDRE)	Araiza-Rodriguez Adnan; Barrera-Badillo Gisela; Garces-Ayala Fabiola; Hernandez-Rivas Lucia; Lopez-Martinez Irma; Lopez-Martinez Irma.; Mendieta-Condado Edgar; Ramirez-Gonzalez Ernesto; Rodriguez-Maldonado Abril; Wong-Arambula Claudia
EPI_ISL_660068	Instituto de Diagnostico y Referencia Epidemiologicos (INDRE)	Instituto de diagnóstico y Referencia Epidemiologicos (INDRE) Departamento de Virología	Abril Rodriguez-Maldonado; Claudia Wong-Arambula; Dayanira Arellano-Suarez; Ernesto Ramirez-Gonzalez.; Fabiola Garces-Ayala; Gisela Barrera-Badillo; Irma Lopez-Martinez; Lucia Hernandez-Rivas; Natividad Cruz-Ortiz; Tatiana Nunez-Garcia
EPI_ISL_747615, EPI_ISL_748138, EPI_ISL_748139, EPI_ISL_748140, EPI_ISL_748141, EPI_ISL_748142, EPI_ISL_748143, EPI_ISL_748144, EPI_ISL_748145			
see above	Instituto de Investigaciones Biologicas Clemente Estable	Institut Pasteur de Montevideo	Ana Carolina Mendonça; Andres Lizasoain; Camila Simoes; Cecilia Alonso; Cecilia Salazar; Daiana Mir; Fernando Lopez-Tort; Fernando Motta; Gonzalo Bello; Ighor Arantes; Ignacio Ferrés; Jose Sotelo; Leticia Maya; Letícia Garay Martins; Luciana Appolinario; Lucia Spangenberg; Mailen Arleo; Mariana Brandes; Marilda Mendonça Siqueira; Marilda Tereza Mar da Rosa; Maria Jose Benitez-Galeano; Martín Graña; Matias Castells; Matias Victoria; Matías Salvo; Natalia Rego; Natalia Reyes; Pablo Smircich; Paola Cristina Resende; Rodney Colina; Tamara Fernandez-Calero; Tania Possi; Tatiana Schäffer Gregianini; Veronica Noya; Yasser Vega
EPI_ISL_792320, EPI_ISL_792321, EPI_ISL_792322, EPI_ISL_792323, EPI_ISL_792324, EPI_ISL_792325, EPI_ISL_792326, EPI_ISL_792327, EPI_ISL_792328, EPI_ISL_792329, EPI_ISL_792330, EPI_ISL_792331, EPI_ISL_792332			
see above	Instituto de Investigaciones Biomédicas en Retrovirus y SIDA	Área de Secuenciación del Laboratorio de Virología del Hospital de Niños Dr. Ricardo Gutierrez on behalf of 'Proyecto Argentino Interinstitucional de genomica de SARS-CoV-2' (PAIS Consortium)	F; Goya; H; LE; Lusso; MI; MS; Nabaes Jodar; Natale; Remes Lenicov; S; Salomón; Seery; V; Valinotto; Viegas, M.
EPI_ISL_722130, EPI_ISL_722131	Instituto de Medicina Tropical Universidade de São Paulo	Laboratório de Parasitologia Médica - Instituto de Medicina Tropical - Universidade de São Paulo	Brazil-UK Centre for Arbovirus Discovery Diagnosis Genomics and Epidemiology (CADDE) Genomic Network - Instituto de Medicina Tropical
EPI_ISL_729869, EPI_ISL_729870, EPI_ISL_729871, EPI_ISL_729902, EPI_ISL_729903, EPI_ISL_729904, EPI_ISL_729905, EPI_ISL_729906, EPI_ISL_729907, EPI_ISL_729908, EPI_ISL_729909, EPI_ISL_729910, EPI_ISL_729911, EPI_ISL_729912, EPI_ISL_729913, EPI_ISL_729914, EPI_ISL_729915, EPI_ISL_729916, EPI_ISL_729917, EPI_ISL_729918, EPI_ISL_729919			
see above	Instituto de Medicina Tropical, Universidad Nacional Toribio Rodríguez de Mendoza de Amazonas	Laboratorio de Genómica Microbiana, Universidad Peruana Cayetano Heredia	Alejandra Dávila-Barclay; Brenda Ayzanoa; Carla Montenegro; Cecilia Pajuelo; Janet Huancachoque; Luis González; Pablo Tsukayama; Pedro E. Romero; Pool Marcos; Rafael Tapia; Stella Chenet
EPI_ISL_499083	Instituto de Virologia "Dr. J. M. Vanella", Facultad de Ciencias Medicas, Universidad Nacional de Cordoba. Laboratorio Central de la Provincia de Cordoba, Argentina. Ministerio de Salud de la provincia de Cordoba, Argentina.	Laboratorio de Virología, Hospital de Niños Ricardo Gutiérrez, CABA, Argentina.	Adrian Diaz; Brenda Konigheim; Gabriela Barbas; Gonzalo Castro; Javier Aguilar; Lorena Spinsanti; Mariana Viegas.; Mercedes Nabaes; Monica Natale; Sandra Gallego; Sebastian Blanco; Silvina Lusso; Stephanie Goya
EPI_ISL_836978	Irmandade da Santa Casa de Misericordia de Lorena	Instituto Adolfo Lutz, Interdiciplinary Procedures Center, Strategic Laboratory	Claudia Regina Gonçalves; Claudio Tavares Sacchi; Erica Valessa Ramos Gomes; Karoline Rodrigues Campos
EPI_ISL_475000, EPI_ISL_475004, EPI_ISL_475008, EPI_ISL_475023, EPI_ISL_649107	Israel Central Virology laboratory	Israel Central Virology laboratory	Efrat Dahan Bucris; Ella Mendelson; Michal Mandelboim; Neta Zuckerman; Oran Erster
EPI_ISL_469020, EPI_ISL_525565	Istituto Zooprofilattico Sperimentale Puglia e Basilicata; Dipartimento di Bioscienze, Biotecnologie e Biofarmaceutica dell'Università degli Studi di Bari "A.Moro"; Istituto di Biomembrane. Bioenergetica e Biotecnologie Molecolari del Consiglio Nazionale delle Ricerche di Bari	Beaconlab (Bioinformatics, Evolution and Comparative Genomics lab), Dept of Biosciences, University on Milan	Chiara M; Chiara M.; Manzari C.; Parisi A.; Pesole G.
EPI_ISL_778649, EPI_ISL_778727	Istituto Zooprofilattico Sperimentale del Mezzogiorno	TIGEM	Andrea Ballabio; Anna Manfredi; Antonio Grimaldi; Antonio Limone; Biancamaria Pierri; Chiara Colantuono; Davide Cacchiarelli (CorrespAuthor); Francesco Panariello; Gabriella Loconte; Lucia Vassallo; Lucio Di Filippo; Marcello Salvi; Patrizia Annunziata; Pellegrino Cerino; Valentina Bouche
EPI_ISL_631867, EPI_ISL_632106, EPI_ISL_632184	Jamaica Hospital Medical Center	New York City Public Health Laboratory	Jade Wang; et al.
EPI_ISL_450792, EPI_ISL_450793, EPI_ISL_450794, EPI_ISL_450795, EPI_ISL_450796, EPI_ISL_450797, EPI_ISL_450798, EPI_ISL_450799			
see above	Jamaica Ministry of Health and Wellness	Pathogen Discovery, Respiratory Viruses Branch, Division of Viral Diseases, Centers for Disease Control and Prevention	Anna Montmayeur; Anna Uehara; Bettina Bankamp; Clinton R. Paden; Haibin Wang; Jasmine Padilla; Jing Zhang; Justin Lee; Krista Queen; Rachel Marine; Suxiang Tong; Yan Li; Ying Tao; Zachary Weiner
EPI_ISL_420342, EPI_ISL_420402, EPI_ISL_420438	KU Leuven, Clinical and Epidemiological Virology	KU Leuven, Clinical and Epidemiological Virology	Bert Vanmechelen; Joan Marti-Carreras; Piet Maes; Tony Wawina
EPI_ISL_462160, EPI_ISL_462205, EPI_ISL_462213, EPI_ISL_476996	KU Leuven, Rega Institute, Clinical and Epidemiological Virology	KU Leuven, Rega Institute, Clinical and Epidemiological Virology	Bert Vanmechelen; Joan Marti-Carerras; Piet Maes; Tony Wawina-Bokalanga
EPI_ISL_454464, EPI_ISL_454483, EPI_ISL_454484, EPI_ISL_454874, EPI_ISL_454878, EPI_ISL_454879, EPI_ISL_454885, EPI_ISL_454893, EPI_ISL_454897, EPI_ISL_455847, EPI_ISL_455859, EPI_ISL_455876, EPI_ISL_455897, EPI_ISL_510861, EPI_ISL_615086, EPI_ISL_615088			
see above	Karolinska Universitetslaboratoriet	The Public Health Agency of Sweden	Anna Risberg; Anna-Malin Linde; Karin Tegmark-Wisell; Maria Lind Karlberg; Mattias Haukland; Mia Brytting; Olov Svartstrom; Oskar Karlsson Lindsjo; Petra Edquist; Reza Advani; Sandra Broddesson; Shamam Muradrasoli
EPI_ISL_512812	Kenema Government Hospital, Ministry of Health and Sanitation	Kenema Government Hospital, Ministry of Health and Sanitation	Andersen, K.; Garry, R.; Goba, A.; Grant, D.; Happi, C.; Jalloh, S.; Mehta, S.; Momoh, M.; Olawoye, I.; Oluniyi, P.; Park, D.; Sandi, J.; Siddle, K.; Tomkins-Tinch, C.
EPI_ISL_459874, EPI_ISL_459880	Kingston Health Sciences Center	Queen's Genomics Lab at Ongwanada (Q-GLO)	Ayub M; Colautti R; Evans GA; Guan H; Huang D; Hudson ML; Perez-Patrigeon S; Rustom N; Sheth P; Sjaarda CP; Soares CN; Wong H
EPI_ISL_538329, EPI_ISL_538338, EPI_ISL_538365	Kingston Health Sciences Centre / Queen's University	Ontario Institute for Cancer Research	Bernard Lam; Calvin Sjaarda; Ilinca Lungu; Jared T. Simpson; Katya Douchant; Lawrence E Heisler; Michael Laszloffy; Paul Krzyzanowski; Prameet M. Sheth; Richard de Borja; Robert Colautti
EPI_ISL_717742	Kingston Health Sciences Centre and Queen's University	Ontario Institute for Cancer Research	Bernard Lam; Calvin Sjaarda; Ilinca Lungu; Jared T. Simpson; Katya Douchant; Lawrence E. Heisler; Michael Laszloffy; Paul Krzyzanowski; Prameet M. Sheth; Richard de Borja; Robert Colautti
EPI_ISL_660417	Klinisk mikrobiologi	The Public Health Agency of Sweden	Anna Risberg; Anna-Malin Linde; Karin Tegmark-Wisell; Maria Lind Karlberg; Mattias Haukland; Mia Brytting; Olov Svartstrom; Oskar Karlsson Lindsjo; Petra Edquist; Reza Advani; Sandra Broddesson
EPI_ISL_429160	Klinisk mikrobiologi Orebro	The Public Health Agency of Sweden	Anna Risberg; Anna-Malin Linde; Karin Tegmark-Wisell; Maria Lind Karlberg; Martin Sundqvist; Olov Svartstrom; Oskar Karlsson Lindsjo; Shaman Muradrasoli
EPI_ISL_429122	Klinisk mikrobiologi och vardhygien Halmstad	The Public Health Agency of Sweden	Anna Risberg; Anna-Malin Linde; Arne Kotz; Karin Tegmark-Wisell; Maria Lind Karlberg; Olov Svartstrom; Oskar Karlsson Lindsjo; Shaman Muradrasoli
EPI_ISL_455904	Klinisk mikrobiologi, UAS	The Public Health Agency of Sweden	Anna Risberg; Anna-Malin Linde; Karin Tegmark-Wisell; Maria Lind Karlberg; Mattias Haukland; Olov Svartstrom; Oskar Karlsson Lindsjo; Petra Edquist; Reza Advani; Shamam Muradrasoli
EPI_ISL_660408, EPI_ISL_661279	Klinsisk mikrobiologi Linkoping	The Public Health Agency of Sweden	Anna Risberg; Anna-Malin Linde; Department of Microbiology; Karin Tegmark-Wisell; Maria Lind Karlberg; Mattias Haukland; Mia Brytting; Olov Svartstrom; Oskar Karlsson Lindsjo; Petra Edquist; Reza Advani; Sandra Broddesson; The Public Health Agency of Sweden
EPI_ISL_445271	LABORATORIO CLINICA CHILLAN	Instituto de Salud Publica de Chile	Alejandra Acevedo; Andrés E Castillo; Bárbara Parra; Carolina Tambley; Gabriel Leal; Jaime Lagos; Jorge Fernandez; Loredana Arata; Patricia Bustos; Paz Tapia; Rodrigo Fasce; Winston Andrade
EPI_ISL_445333	LABORATORIO CLINICA UNIVERSITARIA DE CONCEPCION	Instituto de Salud Publica de Chile	Alejandra Acevedo; Andrés E Castillo; Bárbara Parra; Carolina Tambley; Gabriel Leal; Jaime Lagos; Jorge Fernandez; Loredana Arata; Patricia Bustos; Paz Tapia; Rodrigo Fasce; Winston Andrade

EPI_ISL_792403, EPI_ISL_792405, EPI_ISL_792407, EPI_ISL_792408, EPI_ISL_792409, EPI_ISL_792410, EPI_ISL_792411, EPI_ISL_792412, EPI_ISL_792413, EPI_ISL_792414, EPI_ISL_792415, EPI_ISL_792417, EPI_ISL_792418, EPI_ISL_792421, EPI_ISL_792422, EPI_ISL_792423			
see above	LABORATORIO DE CAMPAÑA COVID 19 INTA	Instituto de Biotecnología, IABIMO (CONICET), Instituto de Virología, IVIT(CONICET), Instituto de Patobiología, IPVET(CONICET), CICVyA, INTA on behalf of 'Proyecto Argentino Interinstitucional de genomica de SARS-CoV-2' (PAIS Consortium)	A; AF; AJ; AV; Asurmendi; B; Bengoa Luoni; Cacciabué; Chimeno; Craig; D; Del Médico; Delgado; Diagiacomo; Distéfano; Dus Santos; Díaz Carrasco; E; F; Farber; Fass; Ferández PC; Franco; Fusco; GA; Garbaccio; Gioffré; Gómez; Huertas; I; J; K; Klepp; König; L; LC; Lozano Calderón; Lucero; M; MD; MG; MI; MJ; MPD; MS; Muñoz Hidalgo; NB; Olivera; P; PA; Paniego; Pedroarias; Peralta; Perea; Pereda; Puebla; Rimondi; Rivarola; Rodríguez; Rosende; S; Schammas; Sioya; Soria; Trono; V; VC; Vera; Viegas, M.; Vissani; W; Zavallo; Zumárraga; Álvarez
EPI_ISL_445279	LABORATORIO INMUNOLAB SPA	Instituto de Salud Publica de Chile	Alejandra Acevedo; Andrés E Castillo; Bárbara Parra; Carolina Tambley; Gabriel Leal; Jaime Lagos; Jorge Fernandez; Loredana Arata; Patricia Bustos; Paz Tapia; Rodrigo Fasce; Winston Andrade
EPI_ISL_445273, EPI_ISL_445274, EPI_ISL_445278	LABORATORIO TORRE MEDICA LTDA.	Instituto de Salud Publica de Chile	Alejandra Acevedo; Andrés E Castillo; Bárbara Parra; Carolina Tambley; Gabriel Leal; Jaime Lagos; Jorge Fernandez; Loredana Arata; Patricia Bustos; Paz Tapia; Rodrigo Fasce; Winston Andrade
EPI_ISL_682275, EPI_ISL_682276, EPI_ISL_682277	LABORATORIOS LABIN	Inciensa, Instituto Costarricense de Investigación y Enseñanza en Nutrición y Salud	Adriana Godinez; Claudio Soto-Garita; Estela Cordero; Francisco Duarte; Hebleen Porras; Melany Calderon & Pei Ling Chan
EPI_ISL_717809, EPI_ISL_717832, EPI_ISL_717833, EPI_ISL_717834, EPI_ISL_717835, EPI_ISL_717836, EPI_ISL_717841, EPI_ISL_717910, EPI_ISL_717911, EPI_ISL_717912, EPI_ISL_717913, EPI_ISL_717914, EPI_ISL_717915, EPI_ISL_717916, EPI_ISL_717917, EPI_ISL_717918, EPI_ISL_717919, EPI_ISL_717958, EPI_ISL_717963, EPI_ISL_717964			
see above	LACEN Dr. Francisco Rimolo Neto	Bioinformatics Laboratory / LNCC	Alexandra L Gerber; Amilcar Tanuri; Ana Paula de C Guimarães; Ana Tereza R de Vasconcelos; Andréa Cony Cavalcanti; Carolina M Voloch; Claudia dos Santos Rodrigues; Cynthia C Cardoso; Diana Mariani; Luiz G P de Almeida; Otavio Bustrolini; Ronaldo da Silva F Jr; Terezinha M P P Castiñeira
EPI_ISL_414045	LACEN RJ - Laboratório Central de Saúde Pública Noel Nutels	Instituto Oswaldo Cruz FIOCRUZ - Laboratory of Respiratory Viruses and Measles (LVRS)	Aline Mattos; Alisson Fabri; Braulia Caetano; Cristiana Garcia; Fernando Motta; Joilson Xavier; Jonathan Lopes; Luciana Appolinario; Maria Nóbrega; Maria Ogrzewalska; Marilda Siqueira; Milene Miranda; Paola Resende; Sunando Roy
EPI_ISL_456076, EPI_ISL_456077, EPI_ISL_456082, EPI_ISL_456083, EPI_ISL_456088	LACEN RJ - Laboratório Central de Saúde Pública Noel Nutels	Laboratory of Respiratory Viruses and Measles, Oswaldo Cruz Institute, FIOCRUZ	Aline Mattos; Braulia Caetano; Cristiana Garcia; Fernando Motta; Jonathan Lopes; Luciana Appolinario; Maria Ogrzewalska; Marilda Siqueira; Milene Miranda; Paola Resende
EPI_ISL_717785, EPI_ISL_717786, EPI_ISL_717787, EPI_ISL_717788, EPI_ISL_717789, EPI_ISL_717790, EPI_ISL_717792, EPI_ISL_717794, EPI_ISL_717899, EPI_ISL_717900, EPI_ISL_717901, EPI_ISL_717902, EPI_ISL_717903, EPI_ISL_717904, EPI_ISL_717905, EPI_ISL_717906, EPI_ISL_717907, EPI_ISL_717908, EPI_ISL_717909, EPI_ISL_717962			
see above	LACEN RJ - Noel Nutels	Bioinformatics Laboratory / LNCC	Alexandra L Gerber; Amilcar Tanuri; Ana Paula de C Guimarães; Ana Tereza R de Vasconcelos; Andréa Cony Cavalcanti; Carolina M Voloch; Claudia dos Santos Rodrigues; Cynthia C Cardoso; Diana Mariani; Luiz G P de Almeida; Otavio Bustrolini; Ronaldo da Silva F Jr; Terezinha M P P Castiñeira
EPI_ISL_792639, EPI_ISL_792640, EPI_ISL_792641, EPI_ISL_792642, EPI_ISL_792643, EPI_ISL_792644	LACEN-AL	Laboratory of Respiratory Viruses and Measles, Oswaldo Cruz Institute, FIOCRUZ	Ana Carolina Mendonca; Anderson Brandao Leite; Anna Carolina Paixao; Fernando Motta; Luciana Appolinario; Marilda Siqueira; Paola Resende
EPI_ISL_427292	LACEN-AL - Laboratorio Central de Alagoas	Instituto Oswaldo Cruz FIOCRUZ - Laboratory of Respiratory Viruses and Measles (LVRS)	Aline Mattos; Braulia Caetano; Cristiana Garcia; Fernando Motta; Jonathan Lopes; Luciana Appolinario; Maria Ogrzewalska; Marilda Siqueira; Milene Miranda; Paola Resende; Priscila Born; Sunando Roy
EPI_ISL_427293	LACEN-BA - Laboratório Central de Saúde Pública Professor Gonçalo Moniz	Instituto Oswaldo Cruz FIOCRUZ - Laboratory of Respiratory Viruses and Measles (LVRS)	Aline Mattos; Braulia Caetano; Cristiana Garcia; Fernando Motta; Jonathan Lopes; Luciana Appolinario; Maria Ogrzewalska; Marilda Siqueira; Milene Miranda; Paola Resende; Priscila Born; Sunando Roy
EPI_ISL_792561, EPI_ISL_792562, EPI_ISL_792563, EPI_ISL_792564, EPI_ISL_792565, EPI_ISL_792566, EPI_ISL_792567, EPI_ISL_792568, EPI_ISL_792569, EPI_ISL_792570, EPI_ISL_792571, EPI_ISL_792572, EPI_ISL_792573, EPI_ISL_792574, EPI_ISL_792575, EPI_ISL_792576, EPI_ISL_792577, EPI_ISL_792578, EPI_ISL_792579, EPI_ISL_792580, EPI_ISL_792581, EPI_ISL_792582, EPI_ISL_792583, EPI_ISL_792584, EPI_ISL_792585, EPI_ISL_792586, EPI_ISL_792587, EPI_ISL_792588, EPI_ISL_792589, EPI_ISL_792590, EPI_ISL_792591, EPI_ISL_792592, EPI_ISL_792593, EPI_ISL_792594, EPI_ISL_792595, EPI_ISL_792596, EPI_ISL_792597, EPI_ISL_792598, EPI_ISL_792599, EPI_ISL_792600, EPI_ISL_792601, EPI_ISL_792602, EPI_ISL_792603, EPI_ISL_792604, EPI_ISL_792605, EPI_ISL_792606, EPI_ISL_792607, EPI_ISL_792608, EPI_ISL_792609, EPI_ISL_792610, EPI_ISL_792611, EPI_ISL_792612, EPI_ISL_792613, EPI_ISL_792614, EPI_ISL_792615, EPI_ISL_792616, EPI_ISL_792617, EPI_ISL_792618, EPI_ISL_792619, EPI_ISL_792620, EPI_ISL_792621, EPI_ISL_792622, EPI_ISL_792623, EPI_ISL_792624, EPI_ISL_792625, EPI_ISL_792626, EPI_ISL_792627, EPI_ISL_792628, EPI_ISL_792629, EPI_ISL_792630, EPI_ISL_792631, EPI_ISL_792632, EPI_ISL_792633, EPI_ISL_792634, EPI_ISL_792635, EPI_ISL_792636, EPI_ISL_792637, EPI_ISL_792638			
see above	LACEN-PB	Laboratory of Respiratory Viruses and Measles, Oswaldo Cruz Institute, FIOCRUZ	Ana Carolina Mendonca; Anna Carolina Paixao; Dalane Loudal Florentino Teixeira; Fernando Motta; João Felipe Bezerra; Luciana Appolinario; Marilda Siqueira; Paola Resende; Romero Henrique Teixeira de Vasconcelos; Thiago Franco de Oliveira Carneiro
EPI_ISL_792645, EPI_ISL_792646, EPI_ISL_792647, EPI_ISL_792648, EPI_ISL_792649, EPI_ISL_792650, EPI_ISL_792651, EPI_ISL_792652, EPI_ISL_792653, EPI_ISL_792654			
see above	LACEN-PR	Laboratory of Respiratory Viruses and Measles, Oswaldo Cruz Institute, FIOCRUZ	Ana Carolina Mendonca; Anna Carolina Paixao; Fernando Motta; Irina Nastassja Riediger; Luciana Appolinario; Maria do Carmo Debur; Marilda Siqueira; Paola Resende
EPI_ISL_427305, EPI_ISL_427306	LACEN-SC - Laboratorio Central de Santa Catarina	Instituto Oswaldo Cruz FIOCRUZ - Laboratory of Respiratory Viruses and Measles (LVRS)	Aline Mattos; Braulia Caetano; Cristiana Garcia; Fernando Motta; Jonathan Lopes; Luciana Appolinario; Maria Ogrzewalska; Marilda Siqueira; Milene Miranda; Paola Resende; Priscila Born; Sunando Roy
EPI_ISL_415128	LACEN/ES - Laboratório Central de Saúde Pública do Espírito Santo	Instituto Oswaldo Cruz FIOCRUZ - Laboratory of Respiratory Viruses and Measles (LVRS)	Aline Mattos; Allison Fabri; Braulia Caetano; Cristiana Garcia; Fernando Motta; Joilson Xavier; Jonathan Lopes; Luciana Appolinario; Maria Nóbrega; Maria Ogrzewalska; Marilda Siqueira; Milene Miranda; Paola Resende; Sunando Roy
EPI_ISL_502779, EPI_ISL_502875	LACEN/PE	LABBE, Federal University of Pernambuco	ANTONIO CARLOS DE FREITAS; BRUNO SAMPAIO; HEIDI LACERDA ALVES DA CRUZ; MAIRA GALDINO DA ROCHA PITTA; MARCOS ANTONIO DE MORAIS JUNIOR; MARCOS DA SILVEIRA REGUEIRA NETO; MICHELLY CRISTINY PEREIRA; REGINALDO GONCALVES DE LIMA NETO; SERGIO DE SA LEITAO PAIVA JUNIOR; VALDIR DE QUEIROZ BALBINO.; WILSON JOSE DA SILVA JUNIOR; ZILDENE DE SOUSA SILVEIRA
EPI_ISL_500460, EPI_ISL_500461, EPI_ISL_500462, EPI_ISL_500463, EPI_ISL_500464, EPI_ISL_500465, EPI_ISL_500466, EPI_ISL_500467, EPI_ISL_500468, EPI_ISL_500469, EPI_ISL_500470, EPI_ISL_500471, EPI_ISL_500472, EPI_ISL_500473, EPI_ISL_500474, EPI_ISL_500475, EPI_ISL_500476, EPI_ISL_500477, EPI_ISL_500478, EPI_ISL_500480, EPI_ISL_500481, EPI_ISL_500482, EPI_ISL_500483, EPI_ISL_500484, EPI_ISL_500485, EPI_ISL_500486, EPI_ISL_500865, EPI_ISL_500866, EPI_ISL_500867, EPI_ISL_500868, EPI_ISL_500869, EPI_ISL_500870, EPI_ISL_500871, EPI_ISL_500872, EPI_ISL_500874, EPI_ISL_500875, EPI_ISL_572334, EPI_ISL_572335, EPI_ISL_572336, EPI_ISL_572338, EPI_ISL_572342, EPI_ISL_572351, EPI_ISL_572353, EPI_ISL_572355, EPI_ISL_572358, EPI_ISL_572359, EPI_ISL_572360, EPI_ISL_572361, EPI_ISL_572363, EPI_ISL_572366, EPI_ISL_572367, EPI_ISL_572371, EPI_ISL_572372, EPI_ISL_572375, EPI_ISL_572379, EPI_ISL_572384, EPI_ISL_572385, EPI_ISL_572386, EPI_ISL_572388, EPI_ISL_572394, EPI_ISL_572396			
see above	LACEN/PE	WallauLab, Aggeu Magalhaes Institute	; Alexandre Freitas da Silva; Antonio Mauro Rezende; Armando de Menezes Neto; Bruna Santos Lima Figueiredo de Sá; Caroline Targino Alves da Silva; Claudio Eduardo Cavalcanti; Constância Flávia Junqueira Ayres; Cássia Docena; Derciliano Lopes da Cruz; Duschinka Ribeiro Duarte Guedes; Elisama Helvecio; Filipe Zimmer Dezordi; Gabriel Luz Wallau; Gonzalo Bello; Gonzalo Bello0; Kamila Gaudêncio da Silva Sales; Larissa Krokovsky; Laís Ceschini Machado; Luciane Caroline Albuquerque Bezerra; Luydson Richardson Silva Vasconcelos; Marcelo Henrique Santos Paiva; Maria Almerice Lopes da Silva; Matheus Filgueira Bezerra; Michelle da Silva Barros; Paola Cristina Resende; Renata Pessôa Germano Mendes; Rodrigo Moraes Loyo Arcoverde; Severino Jefferson Ribeiro da Silva; Sinval Pinto Brandão Filho; Tiago Gräf; Wheverton Ricardo Correia do Nascimento
EPI_ISL_541340, EPI_ISL_541341, EPI_ISL_541342, EPI_ISL_541343, EPI_ISL_541344, EPI_ISL_541345, EPI_ISL_541346			
see above	LACEN/PR	Laboratory of Respiratory Viruses and Measles, Oswaldo Cruz Institute, FIOCRUZ	Ana Carolina Mendonça; Anna Carolina Paixão; Fernando Motta; Irina Riediger; Jonathan Lopes; Luciana Appolinario; Maria do Carmo Debur; Marilda Siqueira; Paola Resende
EPI_ISL_541370, EPI_ISL_541371	LACEN/SC	Laboratory of Respiratory Viruses and Measles, Oswaldo Cruz Institute, FIOCRUZ	Ana Carolina Mendonça; Anna Carolina Paixão; Fernando Motta; Jonathan Lopes; Luciana Appolinario; Marilda Siqueira; Paola Resende; Sandra Bianchini
EPI_ISL_541372, EPI_ISL_541373, EPI_ISL_541374, EPI_ISL_541375, EPI_ISL_541376, EPI_ISL_541377, EPI_ISL_541378, EPI_ISL_541379, EPI_ISL_541380, EPI_ISL_541381, EPI_ISL_541382, EPI_ISL_541383, EPI_ISL_541384, EPI_ISL_541385, EPI_ISL_541386, EPI_ISL_541387, EPI_ISL_541388, EPI_ISL_541389, EPI_ISL_541390, EPI_ISL_541391, EPI_ISL_541392, EPI_ISL_541393, EPI_ISL_541394, EPI_ISL_541395, EPI_ISL_541396			
see above	LACEN/SE	Laboratory of Respiratory Viruses and Measles, Oswaldo Cruz Institute, FIOCRUZ	Ana Carolina Mendonça; Anna Carolina Paixão; Clioma Santos; Fernando Motta; Jonathan Lopes; Luciana Appolinario; Marilda Siqueira; Paola Resende
EPI_ISL_469049, EPI_ISL_469052, EPI_ISL_469054	LNR National Reference Laboratory, Mohammed VI University of Health Sciences	Medical Biotechnology Laboratory, Rabat Medical and Pharmacy School, Mohammed The Vth University in Rabat	Chakib NEJJARI; Houda BENRAHMA; Idrissa Diawara; Imane SMYEJ; Jalil El Atar; Jalila RAHOUI; Lahcen BELYAMANI and Azeddine IBRAHIMI; Laila SBABOU; Loubna ALLAM; M.W. CHEMAO-ELFIHRI; Meriem LAAMARTI; Mouna OUADGHIRI; Rachid EL JAOUDI; Rachid MENTAG; Rokaia LAAMRTI; Saaid AMZAZI; Souad KARTTI
EPI_ISL_794653	LSP DEL TOLIMA	Instituto Nacional de Salud - Dirección de Investigación en Salud Pública	Carlos Franco-Muñoz; Diego A. Álvarez-Díaz; Diego Andrés Prada; Gerardo Santamaría; Jonathan Reales; Julian Naizaque; Katherine Laiton-Donato; Magdalena Wiesner; Marcela Mercado-Reyes; Maria T. Herrera; Martha Lucia Ospina Martinez; Mauricio Pacheco-Montealegre; Paola Muñoz-Laiton;

			Sheryl Corchuelo
EPI_ISL_528637, EPI_ISL_528638	LVM/UFRJ	Bioinformatics Laboratory / LNCC	Amilcar Tanuri; Ana Teresa R. Vasconcelos; Bruno B. Bezerra; Diana Marianni; Elena Cobos; Fabio Limonte; Gustavo D. P. Silva; Isadora A. Correa; Luciana B. Arruda; Luciana J. Costa; Lucio A. Caldas; Luiz Almeida; Luiza Higga; M. Romário M. de Souza; Marcelo Bozza; Orlando Ferreira; Sharton V. A. Coelho; Terezinha M. Castineiras; Wanderley de Souza
EPI_ISL_528539	LVM/UFRJ	LNCC	Amilcar Tanuri; Ana Teresa R. Vasconcelos; Bruno B. Bezerra; Diana Marianni; Elena Cobos; Fabio Limonte; Gustavo M. Romário M. de Souza; Isadora A. Correa; Luciana B. Arruda; Luciana J. Costa.; Lucio A. Caldas; Luiz Almeida; Luiza Higga; Marcelo Bozza; Orlando Ferreira; Sharton V. A. Coelho; Terezinha M. Castineiras; Wanderley de Souza
EPI_ISL_755644, EPI_ISL_755645, EPI_ISL_755652, EPI_ISL_833162	Lab LOC - Itapecerica da Serra	Instituto Adolfo Lutz, Interdiciplinary Procedures Center, Strategic Laboratory	Claudia Regina Gonçalves; Claudio Tavares Sacchi; Erica Valessa Ramos Gomes; Karoline Rodrigues Campos
EPI_ISL_425064, EPI_ISL_451175	Lab voor klinische biologie	Onderzoeksgroep Virologie	Bruno Verhasselt; Hans Nauwynck; Jozefien De Clercq; Laurens Lambrechts; Linos Vandekerckhove; Marthe Pauwels; Nick Vereecke; Sebastiaan Theuns
EPI_ISL_548040, EPI_ISL_548069	LabPLUS	Institute of Environmental Science and Research (ESR)	Anja Werno; Antje van der Linden; Arlo Upton; Chris Mansell; David Hammer; Dragana Drinkovic; Erasmus Smit; Gary McAuliffe; Hana Sofia Andersson; Hermes Perez; James Ussher; Jill Sherwood; Jing Wang; Joep de Ligt; Josh Freeman; Julia Howard; Juliet Elvy; Lauren Jelly; Mary DeAlmeida; Matt Blakiston; Matt Storey; Matthew Rogers; Max Bloomfield; Michael Addidle; Michelle Balm; Muhammad Faisal; Nikki Freed; Olin Silander; Sally Roberts; Sarah Jefferies; Sharmini Muttaiyah; Susan Morpeth; Susan Taylor; Timothy Blackmore; Vani Sathyendran; Veronica Playle; Virginia Hope; Xiaoyun Ren
EPI_ISL_420063	Labo BM - Site de Juvisy - Hopital Général	National Reference Center for Viruses of Respiratory Infections, Institut Pasteur, Paris	Angela Brisebarre; Etienne Simon-Lorière; Flora Donati; Marion Barbet; Maud Vanpeene; Mélanie Albert; Méline Bizard; Sylvie Behillil; Sylvie van der Werf; Vincent Enouf
EPI_ISL_416029	Laboiratório Fleury	Instituto Adolfo Lutz, Interdiciplinary Procedures Center, Strategic Laboratory	Adriana Bugno; Adriano Abbud; Carlos Henrique Camargo; Claudia Regina Gonçalves; Claudio Tavares Sacchi; Daniela Bernardes Borges da Silva; Fabiana Cristina Pereira dos Santos; Maria do Carmo Sampaio Tavares Timenetsky; Simone Guadagnucci Morillo; Terezinha Maria de Paiva
EPI_ISL_421741, EPI_ISL_428956	Laboratoire National de Sante, Microbiology, Virology	Laboratoire National de Sante, Microbiology, Epidemiology and Microbial Genomics	Anke Wienecke-Baldacchino; Ardashel Latsuzbaia; Catherine Ragimbeau; Guillaume Fournier; Jessica Tapp; Joel Mossong; Tamir Abdelrahman; Trung Nguyen Nguyen
EPI_ISL_419584	Laboratoire National de Santé, Microbiology, Virology	Laboratoire National de Santé, Microbiology, Epidemiology and Microbial Genomics	Anke Wienecke-Baldacchino; Ardashel Latsuzbaia; Catherine Ragimbeau; Guillaume Fournier; Jessica Tapp; Joel Mossong; Tamir Abdelrahman; Trung Nguyen Nguyen
EPI_ISL_825868, EPI_ISL_826032	Laboratoire de santé publique du Québec	Laboratoire de santé publique du Québec	Guillaume Bourque; Ioannis Ragoussis; Jesse Shapiro; Mark Lathrop and Michel Roger on behalf of the CoVSeQ research group (http://covseq.ca/researchgroup); Sandrine Moreira
EPI_ISL_629097, EPI_ISL_629105, EPI_ISL_666669	Laboratoire du Centre Hospitalier Annecy Genevois	CNR Virus des Infections Respiratoires - France SUD	Antonin Bal; Bruno Chanzy; Bruno Lina; Gregory Destras; Gwendolyne Burfin; Hadrien Règue; Hélène Petitprez; Laurence Josset; Martine Valette; Quentin Semanas
EPI_ISL_430793	Laboratorio Análisis Clínicos, Unidad de Servicios Diagnósticos, Swiss Medical Group	Área de Secuenciación del Laboratorio de Virología del Hospital de Niños Dr. Ricardo Gutierrez on behalf of 'Proyecto Argentino Interinstitucional de genomica de SARS-CoV-2' (PAIS Consortium)	AS; D; Goya; Guevara; LE; Lusso; MI; MS; Mistchenko; Nabaes Jodar; Natale; O; S; SM; Sanchez; Valinotto; Vicario; Viegas, M.
EPI_ISL_770018, EPI_ISL_770019	Laboratorio Cenahce	Inciensa, Instituto Costarricense de Investigación y Enseñanza en Nutrición y Salud	Adriana Godínez; Claudio Soto-Garita; Estela Cordero; Francisco Duarte; Hebleen Porras; Melany Calderón & Andrés Feoli-Grant
EPI_ISL_792478, EPI_ISL_792481, EPI_ISL_792482, EPI_ISL_792483, EPI_ISL_792484, EPI_ISL_792489, EPI_ISL_792491, EPI_ISL_792492, EPI_ISL_792494, EPI_ISL_792495, EPI_ISL_792496, EPI_ISL_792497, EPI_ISL_792499			
see above	Laboratorio Central Mg. Luis Alfredo Pianciola	Hospital Regional Ushuaia - Centro Austral De Investigaciones Científicas - Universidad Nacional De Tierra Del Fuego on behalf of 'Proyecto Argentino Interinstitucional de genomica de SARS-CoV-2' (PAIS Consortium)	C; CF; Ceballos; F; Gallego; Gramundi; ID; M; MC; Mazzeo; Nardi; Pianciola, L.; Pinto; SG; Ziehm
EPI_ISL_729794, EPI_ISL_729795, EPI_ISL_729796, EPI_ISL_729797, EPI_ISL_729798, EPI_ISL_729799, EPI_ISL_729800, EPI_ISL_729801, EPI_ISL_729802, EPI_ISL_729803, EPI_ISL_729804, EPI_ISL_729805, EPI_ISL_729806, EPI_ISL_729807, EPI_ISL_729808, EPI_ISL_729809, EPI_ISL_729810, EPI_ISL_729811, EPI_ISL_729812, EPI_ISL_729813, EPI_ISL_729814, EPI_ISL_729815, EPI_ISL_729816, EPI_ISL_729817, EPI_ISL_729818, EPI_ISL_729819, EPI_ISL_729820, EPI_ISL_729821, EPI_ISL_729822, EPI_ISL_729823, EPI_ISL_729824, EPI_ISL_729825, EPI_ISL_729826, EPI_ISL_729827, EPI_ISL_729828, EPI_ISL_729829, EPI_ISL_729830, EPI_ISL_729831, EPI_ISL_729832, EPI_ISL_729833, EPI_ISL_729834, EPI_ISL_729835, EPI_ISL_729836, EPI_ISL_729837, EPI_ISL_729838, EPI_ISL_729839, EPI_ISL_729840, EPI_ISL_729841, EPI_ISL_729842, EPI_ISL_729843, EPI_ISL_729844, EPI_ISL_729845, EPI_ISL_729846, EPI_ISL_729847, EPI_ISL_729848, EPI_ISL_729849, EPI_ISL_729850, EPI_ISL_729851, EPI_ISL_729852, EPI_ISL_729853, EPI_ISL_729854, EPI_ISL_729855, EPI_ISL_729856, EPI_ISL_729857, EPI_ISL_729858, EPI_ISL_729859, EPI_ISL_729860, EPI_ISL_729861			
see above	Laboratorio Central de Saude Publica do Estado do Rio Grande do Sul (LACEN-RS)	Laboratory of Respiratory Viruses and Measles, Oswaldo Cruz Institute, FIOCRUZ	Ana Carolina Mendonça; Anna Carolina Paixão; Fernando Motta; Luciana Appolinario; Marilda Siqueira; Marilda Tereza Mar da Rosa; Paola Resende; Tatiana Schaffer Gregianini
EPI_ISL_792512, EPI_ISL_792513, EPI_ISL_792514, EPI_ISL_792515, EPI_ISL_792516, EPI_ISL_792517, EPI_ISL_792518, EPI_ISL_792519, EPI_ISL_792520, EPI_ISL_792521			
see above	Laboratorio Central de la Ciudad de Santa Fe	Grupo de Genómica y Bioinformática del Instituto de Investigación de la Cadena Láctea CONICET-INTA on behalf of 'Proyecto Argentino Interinstitucional de genomica de SARS-CoV-2' (PAIS Consortium)	AF; Amadio; C; Eberhardt; G; Irazoqui; JM; MF; Mugna; Ojeda; Pastor; Rompato; V
EPI_ISL_792526, EPI_ISL_792527, EPI_ISL_792528, EPI_ISL_792529, EPI_ISL_792530, EPI_ISL_792531, EPI_ISL_792532, EPI_ISL_792533, EPI_ISL_792534, EPI_ISL_792535, EPI_ISL_792536, EPI_ISL_792537, EPI_ISL_792538, EPI_ISL_792541, EPI_ISL_792542, EPI_ISL_792543, EPI_ISL_792544, EPI_ISL_792545, EPI_ISL_792546			
see above	Laboratorio Central, Ministerio de Salud Córdoba	Instituto de Patologia Vegetal (CIAP-INTA) on behalf of 'Proyecto Argentino Interinstitucional de genomica de SARS-CoV-2' (PAIS Consortium)	Barbas, G.; Castro, G.; Debat, HJ.; FD; Fernández; MB; Pisano; Re; V
EPI_ISL_769987, EPI_ISL_769991	Laboratorio Clinico Labin	Inciensa, Instituto Costarricense de Investigación y Enseñanza en Nutrición y Salud	Adriana Godinez; Adriana Godínez; Claudio Soto-Garita; Estela Cordero; Francisco Duarte; Hebleen Porras; Melany Calderon & Pei Chan Ma; Melany Calderón & Mariel López
EPI_ISL_751201	Laboratorio DILAVE/MGAP-INIA-UdelaR -Tacuarembó	Institut Pasteur de Montevideo	Ana Carolina Mendonça; Andres Lizasoain; Camila Simoes; Cecilia Alonso; Cecilia Salazar; Daiana Mir; Fernando Lopez-Tort; Fernando Motta; Gonzalo Bello; Ighor Arantes; Ignacio Ferrés; Jose Sotelo; Leticia Maya; Letícia Garay Martins; Luciana Appolinario; Lucia Spangenberg; Mailen Arleo; Mariana Brandes; Marilda Mendonça Siqueira; Marilda Tereza Mar da Rosa; Maria Jose Benitez-Galeano; Martín Graña; Matias Castells; Matias Victoria; Matías Salvo; Natalia Rego; Natalia Reyes; Pablo Smircich; Paola Cristina Resende; Rodney Colina; Tamara Fernandez-Calero; Tania Possi; Tatiana Schäffer Gregianini; Veronica Noya; Yasser Vega
EPI_ISL_424667	Laboratorio Estatal de Salud Publica del Estado de México	Instituto de Diagnóstico y Referencia Epidemiológicos	Adnan Araiza Rodríguez; Alejandro Sánchez; Alfredo Ponce de León Garduño; Blanca Taboada; Carlos F. Arias.; Carolina González Torres; Celia Boukadida; Cesar Raúl González Bonilla; Concepción Grajales Muñiz; Edgar Mendieta Condado; Eduardo Becerril Vargas; Fabiola Garcés Ayala; Fernando Ledesma Barrientos; Francisco Javier Gaytán Cervantes; Francisco Pulido; Gisela Barrera Badillo; Gloria Vázquez; Guillermo M. Ruiz-Palacios; Irma López Martínez; Joel Armando Vázquez Pérez; José Arturo Martínez Orozco; José Ernesto Ramírez González; José Esteban Muñoz Medina; Lucia Hernández Rivas; Luis Alberto García Andrade; Mario Mújica Sánchez; Pavel Isa; Pilar Ramos Cervantes; Ricardo Grande; Santiago Avila Ríos; Victor Hugo Borja Aburto; Violeta Ibarra Gonzalez
EPI_ISL_424672	Laboratorio Estatal de Salud Publica del Estado de Puebla	Instituto de Diagnostico y Referencia Epidemiologicos	Adnan Araiza Rodríguez; Alejandro Sánchez; Alfredo Ponce de León Garduño; Blanca Taboada; Carlos F. Arias; Carolina González Torres; Celia Boukadida; Cesar Raúl González Bonilla; Concepción Grajales Muñiz; Edgar Mendieta Condado; Eduardo Becerril Vargas; Fabiola Garcés Ayala; Fernando Ledesma Barrientos; Francisco Javier Gaytán Cervantes; Francisco Pulido; Gisela Barrera Badillo; Gloria Vázquez; Guillermo M. Ruiz-Palacios; Irma López Martínez; Joel Armando Vázquez Pérez; José Arturo Martínez Orozco; José Ernesto Ramírez González; José Esteban Muñoz Medina; Lucia Hernández Rivas; Luis Alberto García Andrade; Mario Mújica Sánchez; Pavel Isa; Pilar Ramos Cervantes; Ricardo Grande; Santiago Avila Ríos; Victor Hugo Borja Aburto; Violeta Ibarra Gonzalez
EPI_ISL_424670	Laboratorio Estatal de Salud Publica del Estado de Queretaro	Instituto de Diagnóstico y Referencia Epidemiologicos	Adnan Araiza Rodríguez; Alejandro Sánchez; Alfredo Ponce de León Garduño; Blanca Taboada; Carlos F. Arias; Carolina González Torres; Celia Boukadida; Cesar Raúl González Bonilla; Concepción Grajales Muñiz; Edgar Mendieta Condado; Eduardo Becerril Vargas; Fabiola Garcés Ayala;

			Fernando Ledesma Barrientos; Francisco Javier Gaytán Cervantes; Francisco Pulido; Gisela Barrera Badillo; Gloria Vázquez; Guillermo M. Ruiz-Palacios; Irma López Martínez; Joel Armando Vázquez Pérez; José Arturo Martínez Orozco; José Ernesto Ramírez González; José Esteban Muñoz Medina; Lucia Hernández Rivas; Luis Alberto García Andrade; Mario Mújica Sánchez; Pavel Isa; Pilar Ramos Cervantes; Ricardo Grande; Santiago Avila Ríos; Victor Hugo Borja Aburto; Violeta Ibarra Gonzalez
EPI_ISL_779185, EPI_ISL_779186, EPI_ISL_779187, EPI_ISL_779188, EPI_ISL_779189, EPI_ISL_779190, EPI_ISL_779191, EPI_ISL_779192, EPI_ISL_779193, EPI_ISL_779194, EPI_ISL_779195, EPI_ISL_779196, EPI_ISL_779197, EPI_ISL_779198			
see above	Laboratorio Estatal de Salud Pública de Nuevo León	Laboratorio de Infectología Molecular, Departamento de Bioquímica y Medicina Molecular,Facultad de Medicina - Universidad Autónoma de Nuevo León	Ana M. Rivas-Estilla; Consuelo Treviño-Garza; Daniel Arellanos-Soto; Else del Carmen García-García; Gloria A. Jasso-de-la-Peña; Kame A. Galán-Huerta; Manuel E. de-la-O-Cavazos; María F. Herrera-Saldivar; Natalia Martínez-Acuña; Roberto Montes-de-Oca; Samuel Buentello-Wong; Sonia A. Lozano-Sepúlveda
EPI_ISL_794592	Laboratorio Estatal de Salud Pública de Tamaulipas	Instituto de diagnóstico y Referencia Epidemiologicos (INDRE)	Abril Rodriguez-Maldonado; Ana Maria Cortez-Calderon; Bernardita Reyes-Berrones; Celia Alpuche-Aranda; Claudia Wong-Arambula; Ernesto Ramirez-Gonzalez.; Fabiola Garces-Ayala; Gisela Barrera-Badillo; Gloria Molina-Gamboa; Hilda del Carmen-Selvera; Hugo Lopez Gatell-Ramirez; Irma Lopez-Martinez; Jose Luis Alomia-Zegarra; Lucia Hernandez-Rivas
EPI_ISL_735398	Laboratorio Fleury	Instituto Adolfo Lutz, Interdiciplinary Procedures Center, Strategic Laboratory	Claudia Regina Gonçalves; Claudio Tavares Sacchi; Erica Valessa Ramos Gomes; Karoline Rodrigues Campos
EPI_ISL_480328	Laboratorio LABIN	Charité Virology-University of Costa Rica	Andres Moreira-Soto; Eugenia Corrales-Aguilar; Ignacio Postigo-Hidalgo; Ignacio Soto Pacheco; Jan Felix Drexler
EPI_ISL_693246	Laboratorio Municipal de Rio Grande da Serra	Instituto Adolfo Lutz, Interdiciplinary Procedures Center, Strategic Laboratory	Claudia Regina Gonçalves; Claudio Tavares Sacchi; Erica Valessa Ramos Gomes; Karoline Rodrigues Campos
EPI_ISL_837551, EPI_ISL_837552, EPI_ISL_837579, EPI_ISL_837580, EPI_ISL_837581, EPI_ISL_837582, EPI_ISL_837583, EPI_ISL_837584, EPI_ISL_837585, EPI_ISL_837586, EPI_ISL_837587, EPI_ISL_837588, EPI_ISL_837589, EPI_ISL_837590, EPI_ISL_837591, EPI_ISL_837592, EPI_ISL_837593, EPI_ISL_837594, EPI_ISL_837595, EPI_ISL_837596			
see above	Laboratorio Nacional de Salud	Laboratory of Respiratory Viruses and Measles, Oswaldo Cruz Institute, FIOCRUZ	Ana Carolina Mendonca; Anna Carolina Paixao; Cesar Roberto Conde Pereira; Claudia Estrada; Fernando Motta; Luciana Appolinario; Marilda Siqueira; Paola Resende
EPI_ISL_755302, EPI_ISL_755303	Laboratorio de Bioingeniería, Instituto de Ciencias de la Ingeniería, Universidad de O´Higgins	Center for Mathematical Modeling and Center for Genome Regulation. Santiago, Chile	Allende ML; Arriagada G; Bastias M; Bustos F; Castro E; G. Galvez; González M; J. Ortega; M; M. Latorre; Maass A; Meneses C.; Montecino; Orellana A; Sanhueza D; Travisany D
EPI_ISL_468753, EPI_ISL_468754, EPI_ISL_468755, EPI_ISL_468757, EPI_ISL_468758, EPI_ISL_468759	Laboratorio de Biologia Molecular, Facultad de Medicina, Universidad de Atacama	Center for Mathematical Modeling and Center for Genome Regulation. Santiago, Chile	Allende ML; C Echeverria; Gaete A; González M; Maass A; Palma R; Travisany D; Urra C; Varas M
EPI_ISL_457940, EPI_ISL_457942, EPI_ISL_457946, EPI_ISL_457948, EPI_ISL_457952, EPI_ISL_457953, EPI_ISL_457956, EPI_ISL_457957, EPI_ISL_457958, EPI_ISL_457959, EPI_ISL_457960, EPI_ISL_457961, EPI_ISL_457962, EPI_ISL_457963, EPI_ISL_457965, EPI_ISL_457968, EPI_ISL_457969, EPI_ISL_457972, EPI_ISL_457973, EPI_ISL_480432, EPI_ISL_480435, EPI_ISL_480437			
see above	Laboratorio de Biología Molecular Asociación Española Primera en Salud	Departments of Pathology and Medicine, New York University School of Medicine	Adriana Heguy; Christian Marier; Gael Westby; Gonzalo Manrique; Maria Noel Zubillaga; Maria Victoria Elizondo; Matthew T Maurano; Paul Zappile
EPI_ISL_842652	Laboratorio de Biología Molecular Hospital Pedro de Elizalde	Grupo de Genómica y Bioinformática del Instituto de Investigación de la Cadena Láctea CONICET-INTA on behalf of 'Proyecto Argentino Interinstitucional de genomica de SARS-CoV-2' (PAIS Consortium)	A; AF; Alegre; Alexay; Amadio; Aulicino; B; Bressan; C; Chamorro; Claps; D; Diaz; E; Eberhardt; F; FJ; G; Gondolessi; Goya; Gómez; Indart; Irazoqui; J; König; L; Lorenzo; Lusso; M; ME; MF; ML; MS; Marchetti; Martín; Montoto Piazza; Morandi; N; Nabaes Jodar; Natale; Osaba; P; Paez; Rocovich; Rosales; S; Sanchez; Sueiro; Torres; Valinotto; Viegas, M.; Wenk; Zamora
EPI_ISL_626549, EPI_ISL_626550, EPI_ISL_626551, EPI_ISL_626552, EPI_ISL_626553, EPI_ISL_626554, EPI_ISL_626555, EPI_ISL_626556, EPI_ISL_626557, EPI_ISL_626558, EPI_ISL_626559, EPI_ISL_626560, EPI_ISL_626561, EPI_ISL_626562, EPI_ISL_626563, EPI_ISL_626564, EPI_ISL_626565			
see above	Laboratorio de Biología Molecular, Facultad de Medicina, Universidad de Atacama, Copiapo, Chile/ FONDAP CRG, Universidad Andrés Bello, Santiago, Chile	Center for Mathematical Modeling and Center for Genome Regulation. Santiago, Chile	Allende ML; Bastias M; Castro E; Echeverría C; González M; M; Maass A; Manríquez R; Meneses C.; Montecino; Orellana A; Sanhueza D; Travisany D
EPI_ISL_792354, EPI_ISL_792355, EPI_ISL_792356, EPI_ISL_792357	Laboratorio de Biología Molecular. Hospital Dr. Héctor Cura	Área de Secuenciación del Laboratorio de Virología del Hospital de Niños Dr. Ricardo Gutierrez on behalf of 'Proyecto Argentino Interinstitucional de genomica de SARS-CoV-2' (PAIS Consortium)	Ghiano; Goya; J; LE; Lusso; MB; MI; MS; N; Nabaes Jodar; Natale; R; S; Spina; Turrina; Valinotto; Viegas, M.; Zaffanella
EPI_ISL_417034, EPI_ISL_792560, EPI_ISL_801386, EPI_ISL_801387, EPI_ISL_801388, EPI_ISL_801389, EPI_ISL_801390, EPI_ISL_801391, EPI_ISL_801392, EPI_ISL_801393, EPI_ISL_801394, EPI_ISL_801395, EPI_ISL_801396, EPI_ISL_811148, EPI_ISL_811149, EPI_ISL_833131, EPI_ISL_833132, EPI_ISL_833133, EPI_ISL_833134, EPI_ISL_833135, EPI_ISL_833136, EPI_ISL_833137, EPI_ISL_833138, EPI_ISL_833139, EPI_ISL_833140			
see above	Laboratorio de Ecologia de Doencas Transmissiveis na Amazonia, Instituto Leonidas e Maria Deane - Fiocruz Amazonia	Laboratorio de Ecologia de Doencas Transmissiveis na Amazonia, Instituto Leonidas e Maria Deane - Fiocruz Amazonia	André Corado; Debora Duarte; Felipe Naveca; Fernanda Nascimento; George Silva; Karina Pessoa; Luciana Gonçalves; Maria Júlia Brandão; Matilde Mejía; Michele Jesus; Sérgio Luz; Valdinete Nascimento; Victor Souza; Ágatha Costa
EPI_ISL_545955	Laboratorio de Infecciones Respiratorias Agudas. Centro Nacional de Salud Publica,Instituto Nacional de Salud	Laboratorio de Infecciones Respiratorias Agudas. Centro Nacional de Salud Publica,Instituto Nacional de Salud	Juscamayta, E.
EPI_ISL_591531, EPI_ISL_591532, EPI_ISL_591533, EPI_ISL_591534	Laboratorio de Infectologia y virologia molecular	Center for Mathematical Modeling and Center for Genome Regulation. Santiago, Chile	Allende ML; Ferres M.; Gaete A; González M; Maass A; Palma R; Travisany D; Urra C; Valiente F; Varas M
EPI_ISL_648595, EPI_ISL_648602, EPI_ISL_648603	Laboratorio de Infectología Servicio de Infectología Hospital Universitario Dr. José Eleuterio González - Universidad Autónoma de Nuevo León	Laboratorio de Infectología Molecular Departamento de Bioquímica y Medicina Molecular Facultad de Medicina - Universidad Autónoma de Nuevo León	Adrian Camacho-Ortiz; Ana M. Rivas-Estilla; Consuelo Treviño-Garza; Daniel Arellanos-Soto; Eduardo Perez-Alba; Elvira Garza-González; Kame A. Galán-Huerta; Laura Nuzzolo-Shihadeh; Manuel E. de-la-O-Cavazos; María F. Herrera-Saldivar; Natalia Martínez-Acuña; Paola Bocanegra-Ibarias; Roberto Montes-de-Oca; Samantha M. Flores-Treviño; Sonia A. Lozano-Sepúlveda
EPI_ISL_648607, EPI_ISL_779169, EPI_ISL_779170, EPI_ISL_779171, EPI_ISL_779172, EPI_ISL_779173, EPI_ISL_779174, EPI_ISL_779175, EPI_ISL_779176, EPI_ISL_779177, EPI_ISL_779178, EPI_ISL_779179, EPI_ISL_779180, EPI_ISL_779181, EPI_ISL_779182, EPI_ISL_779183, EPI_ISL_779184			
see above	Laboratorio de Infectología, Servicio de Infectología, Hospital Universitario Dr. José Eleuterio González - Universidad Autónoma de Nuevo León	Laboratorio de Infectología Molecular, Departamento de Bioquímica y Medicina Molecular,Facultad de Medicina - Universidad Autónoma de Nuevo León	Adrian Camacho-Ortiz; Ana M. Rivas-Estilla; Consuelo Treviño-Garza; Daniel Arellanos-Soto; Eduardo Perez-Alba; Elvira Garza-González; Kame A. Galán-Huerta; Laura Nuzzolo-Shihadeh; Manuel E. de-la-O-Cavazos; María F. Herrera-Saldivar; Natalia Martínez-Acuña; Paola Bocanegra-Ibarias; Roberto Montes-de-Oca; Samantha M. Flores-Treviño; Sonia A. Lozano-Sepúlveda
EPI_ISL_792500, EPI_ISL_792501, EPI_ISL_792502, EPI_ISL_792503, EPI_ISL_792504, EPI_ISL_792505, EPI_ISL_792506, EPI_ISL_792508, EPI_ISL_792510, EPI_ISL_792511			
see above	Laboratorio de Inmunología del Hospital Perrando e Instituto de Medicina Regional de la UNNE	Grupo de Genómica y Bioinformática del Instituto de Investigación de la Cadena Láctea CONICET-INTA on behalf of 'Proyecto Argentino Interinstitucional de genomica de SARS-CoV-2' (PAIS Consortium)	A; AF; Amadio; Ayala; Cayré; Deluca; Eberhardt; Foussal; G; Giusiano; Gómez; H; Irazoqui; JM; L; Lescano; Lucero; M; MD; MF; MV; Marin; NA
EPI_ISL_792425, EPI_ISL_792426, EPI_ISL_792427, EPI_ISL_792430, EPI_ISL_792431, EPI_ISL_792432, EPI_ISL_792433, EPI_ISL_792434, EPI_ISL_792435, EPI_ISL_792436, EPI_ISL_792437, EPI_ISL_792439, EPI_ISL_792440, EPI_ISL_792441, EPI_ISL_792442			
see above	Laboratorio de Inmunología del Hospital Perrando e Instituto de Medicina Regional de la UNNE	Instituto de Biotecnología, IABIMO (CONICET), Instituto de Virología, IVIT(CONICET), Instituto de Patobiología, IPVET(CONICET), CICVyA, INTA on behalf of 'Proyecto Argentino Interinstitucional de genomica de SARS-CoV-2' (PAIS Consortium)	A; AF; AJ; AV; Asurmendi; Ayala; Bengoa Luoni; Cacciabué; Cayré; D; Deluca; Distéfano; Farber; Fass; Foussal; G; GA; Giusiano; Gómez; H; König; L; LC; Lescano; Lozano Calderón; Lucero; M; MD; MG; MPD; MV; Marin; Muñoz Hidalgo; NA; NB; PA; Paniego; Pedroarias; Peralta; Puebla; Rivarola; S; VC; Vera; Viegas, M.; Zavallo
EPI_ISL_517770, EPI_ISL_517959	Laboratorio de Referencia Nacional de Virus Respiratorio. Centro Nacional de Salud Publica. Instituto Nacional de Salud Peru	Laboratorio de Referencia Nacional de Biotecnología y Biología Molecular. Centro Nacional de Salud Publica. Instituto Nacional de Salud Peru.	Carlos Padilla Rojas; Henri Bailon Calderon; Johanna Balbuena Torres; Karolyn Vega Chozo; Marco Galarza Perez; Maribel Huaringa Nuñez; Nancy Rojas Serrano.; Omar Caceres Rey; Priscila Lope Pari
EPI_ISL_516626, EPI_ISL_516628, EPI_ISL_516646, EPI_ISL_516647, EPI_ISL_516653, EPI_ISL_516654, EPI_ISL_516685, EPI_ISL_516686, EPI_ISL_516721, EPI_ISL_516722, EPI_ISL_516987, EPI_ISL_517531, EPI_ISL_517686, EPI_ISL_517687, EPI_ISL_517713, EPI_ISL_517958, EPI_ISL_523810, EPI_ISL_523954, EPI_ISL_523979, EPI_ISL_523994, EPI_ISL_524471, EPI_ISL_524473, EPI_ISL_525206, EPI_ISL_527787, EPI_ISL_527789			

see above	Laboratorio de Referencia Nacional de Virus Respiratorio. Centro Nacional de Salud Publica. Instituto Nacional de Salud Peru.	Laboratorio de Referencia Nacional de Biotecnología y Biología Molecular. Centro Nacional de Salud Publica. Instituto Nacional de Salud Peru.	Carlos Padilla Rojas; Henri Bailon Calderon; Johanna Balbuena Torres; Karolyn Vega Chozo; Marco Galarza Perez; Maribel Huaringa Nuñez; Nancy Rojas Serrano.; Omar Caceres Rey; Priscila Lope Pari
EPI_ISL_489836, EPI_ISL_489837, EPI_ISL_489838, EPI_ISL_489839, EPI_ISL_489987, EPI_ISL_489988, EPI_ISL_489989, EPI_ISL_489990, EPI_ISL_490209, EPI_ISL_490315, EPI_ISL_490316, EPI_ISL_490975, EPI_ISL_490976, EPI_ISL_491427, EPI_ISL_491428, EPI_ISL_491429, EPI_ISL_491430, EPI_ISL_491433, EPI_ISL_491434, EPI_ISL_491435, EPI_ISL_491436, EPI_ISL_491458, EPI_ISL_491459, EPI_ISL_491460, EPI_ISL_491461, EPI_ISL_491462, EPI_ISL_491463, EPI_ISL_491464, EPI_ISL_514226, EPI_ISL_514317, EPI_ISL_514338, EPI_ISL_514339, EPI_ISL_514340, EPI_ISL_514341, EPI_ISL_514342, EPI_ISL_833038			
see above	Laboratorio de Referencia Nacional de Virus Respiratorio. Instituto Nacional de Salud Perú	Laboratorio de Referencia Nacional de Biotecnología y Biología Molecular. Instituto Nacional de Salud Perú	Carlos Padilla Rojas; Henri Bailon Calderon; Johanna Balbuena Torres; Johanna Balbuena Torrez; Karolyn Vega Chozo; Karolyn Chozo Vega; Karolyn Vega Chozo; Luis Barcena; Marco Galarza Perez; Maribel Huaringa Nuñez; Nancy Rojas Serrano; Nancy Rojas Serrano.; Omar Caceres Rey; Priscila Lope Pari
EPI_ISL_491172	Laboratorio de Referencia Nacional de Virus Respiratorio. Instituto Nacional de Salud Perú	Laboratorio de Referencia Nacional de Biotecnología y Biología Molecular. Instituto Nacional de Salud Perú.	Carlos Padilla Rojas; Henri Bailon Calderon; Johanna Balbuena Torres; Karolyn Chozo Vega; Marco Galarza Perez; Maribel Huaringa Nuñez; Nancy Rojas Serrano; Omar Caceres Rey; Priscila Lope Pari
EPI_ISL_487269	Laboratorio de Referencia Nacional de Virus Respiratorio. Instituto Nacional de Salud.	Laboratorio de Referencia Nacional de Biotecnología y Biología Molecular. Instituto Nacional de Salud.	Carlos Padilla Rojas; Henri Bailon Calderon; Johanna Balbuena Torrez; Karolyn Chozo Vega; Marco Galarza Perez; Maribel Huaringa Nuñez; Nancy Rojas Serrano; Omar Caceres Rey; Priscila Lope Pari
EPI_ISL_415787, EPI_ISL_514227	Laboratorio de Referencia Nacional de Virus Respiratorio. Instituto Nacional de Salud. Peru	Laboratorio de Referencia Nacional de Biotecnologia y Biologia Molecular.Instituto Nacional de Salud.Peru	Carlos Padilla Rojas; Hemri Bailon Calderon; Henri Bailon Calderon; Johanna Balbuena Torres; Johanna Balbuena Torrez; Karolyn Vega Chozo; Marco Galarza Perez; Maribel Huaringa Nuñez; Nancy Rojas Serrano; Omar Caceres Rey; Priscila Lope Pari
EPI_ISL_514264, EPI_ISL_514265	Laboratorio de Referencia Nacional de Virus Respiratorio. Instituto Nacional de Salud. Perú	Laboratorio de Referencia Nacional de Biotecnología y Biologia Molecular.Instituto Nacional de Salud.Perú	Carlos Padilla Rojas; Henri Bailon Calderon; Johanna Balbuena Torres; Karolyn Vega Chozo; Marco Galarza Perez; Maribel Huaringa Nuñez; Nancy Rojas Serrano; Omar Caceres Rey; Priscila Lope Pari
EPI_ISL_491431	Laboratorio de Referencia Nacional de Virus Respiratorio. Instituto Nacional de Salud. Perú	Laboratorio de Referencia Nacional de Biotecnología y Biología Molecular. Instituto Nacional de Salud.Perú	Carlos Padilla Rojas; Henri Bailon Calderon; Johanna Balbuena Torres; Karolyn Vega Chozo; Marco Galarza Perez; Maribel Huaringa Nuñez; Nancy Rojas Serrano.; Omar Caceres Rey; Priscila Lope Pari
EPI_ISL_491432	Laboratorio de Referencia Nacional de Virus Respiratorio. Instituto Nacional de Salud.Perú	Laboratorio de Referencia Nacional de Biotecnología y Biología Molecular. Instituto Nacional de Salud.Perú	Carlos Padilla Rojas; Henri Bailon Calderon; Johanna Balbuena Torres; Karolyn Vega Chozo; Marco Galarza Perez; Maribel Huaringa Nuñez; Nancy Rojas Serrano.; Omar Caceres Rey; Priscila Lope Pari
EPI_ISL_529065, EPI_ISL_529066, EPI_ISL_529067, EPI_ISL_529068, EPI_ISL_529069, EPI_ISL_529070, EPI_ISL_529071, EPI_ISL_529072, EPI_ISL_529073, EPI_ISL_529074, EPI_ISL_529075, EPI_ISL_529076, EPI_ISL_529077, EPI_ISL_529078, EPI_ISL_529079, EPI_ISL_540923, EPI_ISL_540924, EPI_ISL_540925, EPI_ISL_540926, EPI_ISL_540927, EPI_ISL_540928, EPI_ISL_540929, EPI_ISL_540930, EPI_ISL_540931, EPI_ISL_540932, EPI_ISL_540933, EPI_ISL_540934, EPI_ISL_540935, EPI_ISL_540936, EPI_ISL_540937, EPI_ISL_540938, EPI_ISL_540939, EPI_ISL_540940, EPI_ISL_540941, EPI_ISL_540942, EPI_ISL_540943, EPI_ISL_540944, EPI_ISL_540945, EPI_ISL_540946, EPI_ISL_540947, EPI_ISL_540948, EPI_ISL_540949, EPI_ISL_540950, EPI_ISL_540951, EPI_ISL_540952, EPI_ISL_540953, EPI_ISL_540954, EPI_ISL_540955, EPI_ISL_540956, EPI_ISL_540957, EPI_ISL_540958, EPI_ISL_540959, EPI_ISL_540960, EPI_ISL_540961, EPI_ISL_540962, EPI_ISL_540963, EPI_ISL_540964, EPI_ISL_540965, EPI_ISL_540966, EPI_ISL_540967, EPI_ISL_540968, EPI_ISL_540969, EPI_ISL_540970, EPI_ISL_540971, EPI_ISL_540972, EPI_ISL_540973, EPI_ISL_540974, EPI_ISL_540975, EPI_ISL_540976, EPI_ISL_540977, EPI_ISL_540978, EPI_ISL_540979, EPI_ISL_540980, EPI_ISL_540981, EPI_ISL_540982, EPI_ISL_540983, EPI_ISL_540984, EPI_ISL_540985, EPI_ISL_540986, EPI_ISL_540987, EPI_ISL_540988, EPI_ISL_540989, EPI_ISL_540990, EPI_ISL_540991, EPI_ISL_540992, EPI_ISL_568510, EPI_ISL_568511, EPI_ISL_568512, EPI_ISL_568513, EPI_ISL_568514, EPI_ISL_568515, EPI_ISL_568516, EPI_ISL_568517, EPI_ISL_568518, EPI_ISL_568519, EPI_ISL_568520, EPI_ISL_568521, EPI_ISL_568522, EPI_ISL_568523, EPI_ISL_568524, EPI_ISL_568525, EPI_ISL_568526, EPI_ISL_568527, EPI_ISL_568528, EPI_ISL_568529, EPI_ISL_568530, EPI_ISL_568531, EPI_ISL_568532, EPI_ISL_568533, EPI_ISL_568534, EPI_ISL_568535, EPI_ISL_568536, EPI_ISL_568537, EPI_ISL_568538, EPI_ISL_568539, EPI_ISL_568540, EPI_ISL_568541, EPI_ISL_568542, EPI_ISL_568543, EPI_ISL_568544, EPI_ISL_568545, EPI_ISL_568546, EPI_ISL_568547, EPI_ISL_568548, EPI_ISL_568549, EPI_ISL_568550, EPI_ISL_568551, EPI_ISL_568552, EPI_ISL_568553, EPI_ISL_568554, EPI_ISL_568555, EPI_ISL_729862, EPI_ISL_729863, EPI_ISL_729864, EPI_ISL_729865, EPI_ISL_729866, EPI_ISL_729867, EPI_ISL_729868, EPI_ISL_729872, EPI_ISL_729873, EPI_ISL_729874, EPI_ISL_729875, EPI_ISL_729876, EPI_ISL_729877, EPI_ISL_729878, EPI_ISL_729879, EPI_ISL_729880, EPI_ISL_729881, EPI_ISL_729882, EPI_ISL_729883, EPI_ISL_729884, EPI_ISL_729885, EPI_ISL_729886, EPI_ISL_729887, EPI_ISL_729888, EPI_ISL_729889, EPI_ISL_729890, EPI_ISL_729891, EPI_ISL_729892, EPI_ISL_729893, EPI_ISL_729894, EPI_ISL_729895, EPI_ISL_729896, EPI_ISL_729897, EPI_ISL_729898, EPI_ISL_729899, EPI_ISL_729900, EPI_ISL_729901, EPI_ISL_812443, EPI_ISL_812444, EPI_ISL_812445, EPI_ISL_812446, EPI_ISL_812447, EPI_ISL_812448, EPI_ISL_812449, EPI_ISL_812450, EPI_ISL_812451, EPI_ISL_812452, EPI_ISL_812453, EPI_ISL_812454, EPI_ISL_812455, EPI_ISL_812456, EPI_ISL_812457, EPI_ISL_812458, EPI_ISL_812459, EPI_ISL_812460, EPI_ISL_812461, EPI_ISL_812462, EPI_ISL_812463, EPI_ISL_812464, EPI_ISL_812465, EPI_ISL_812466, EPI_ISL_812467, EPI_ISL_812468, EPI_ISL_812469, EPI_ISL_812470, EPI_ISL_812471, EPI_ISL_812472, EPI_ISL_812473, EPI_ISL_812474, EPI_ISL_812475, EPI_ISL_812476, EPI_ISL_812477, EPI_ISL_812478, EPI_ISL_812479, EPI_ISL_812480, EPI_ISL_812481, EPI_ISL_812482, EPI_ISL_812483, EPI_ISL_812484, EPI_ISL_812485, EPI_ISL_812486, EPI_ISL_812487, EPI_ISL_812488, EPI_ISL_812489, EPI_ISL_812490, EPI_ISL_812491, EPI_ISL_812492, EPI_ISL_812493, EPI_ISL_812494, EPI_ISL_812495, EPI_ISL_812496, EPI_ISL_812497, EPI_ISL_812498, EPI_ISL_812499, EPI_ISL_812500, EPI_ISL_812501, EPI_ISL_812502, EPI_ISL_812503, EPI_ISL_812504, EPI_ISL_812505, EPI_ISL_812506, EPI_ISL_812507, EPI_ISL_812508, EPI_ISL_812509, EPI_ISL_812510, EPI_ISL_812511, EPI_ISL_812512, EPI_ISL_812513, EPI_ISL_812514, EPI_ISL_812515, EPI_ISL_812516, EPI_ISL_812517, EPI_ISL_812712, EPI_ISL_812713, EPI_ISL_812714, EPI_ISL_812715, EPI_ISL_812716, EPI_ISL_812717, EPI_ISL_812718, EPI_ISL_812719, EPI_ISL_812720, EPI_ISL_812721			
see above	Laboratorio de Referencia Nacional de Virus Respiratorios, Instituto Nacional de Salud Peru	Laboratorio de Genómica Microbiana, Universidad Peruana Cayetano Heredia	Alejandra Dávila-Barclay; Brenda Ayzanoa; Camila Castillo-Vilcahuaman; Camila Castillo-Vilcahuamán; Guillermo Salvatierra; Janet Huancachoque; Luis González; Marco Galarza; Maribel Huaringa; Nancy Rojas; Oscar Escalante; Pablo Tsukayama; Pedro E. Romero; Pool Marcos; Priscila Lope
EPI_ISL_482468	Laboratorio de Referencia Nacional de Virus Respiratorios. Instituto Nacional de Salud Peru	Laboratorio de Referencia Nacional de Biotecnologia y Biologia Molecular. Instituto Nacional de Salud Peru	Carlos Padilla Rojas; Henri Bailon Calderon; Johanna Balbuena Torres; Karolyn Vega Chozo; Maribel Huaringa Nuñez; Nancy Rojas Serrano; Omar Caceres Rey; Priscila Lope Pari
EPI_ISL_792358, EPI_ISL_792359, EPI_ISL_792360, EPI_ISL_792361, EPI_ISL_792362, EPI_ISL_792363, EPI_ISL_792364, EPI_ISL_792365, EPI_ISL_792366, EPI_ISL_792367, EPI_ISL_792368, EPI_ISL_792369, EPI_ISL_792370			
see above	Laboratorio de Virologia - HIEAyC San Juan de Dios	Área de Secuenciación del Laboratorio de Virología del Hospital de Niños Dr. Ricardo Gutierrez on behalf of 'Proyecto Argentino Interinstitucional de genomica de SARS-CoV-2' (PAIS Consortium)	A; Colmeiro; Ercole; Ferioli; Gatelli; Goya; LE; Lusso; M; MI; MS; Nabaes Jodar; Natale; R; S; Valinotto; Viegas, M.
EPI_ISL_470615, EPI_ISL_470616, EPI_ISL_470617, EPI_ISL_470618, EPI_ISL_470619, EPI_ISL_470620, EPI_ISL_470622, EPI_ISL_470623, EPI_ISL_470624, EPI_ISL_470625, EPI_ISL_470626, EPI_ISL_470627, EPI_ISL_470628, EPI_ISL_470629, EPI_ISL_470630, EPI_ISL_470631, EPI_ISL_470632, EPI_ISL_470633, EPI_ISL_470634, EPI_ISL_470635, EPI_ISL_470636, EPI_ISL_470637, EPI_ISL_470638, EPI_ISL_470639, EPI_ISL_470640, EPI_ISL_470641, EPI_ISL_470642, EPI_ISL_470643, EPI_ISL_470644, EPI_ISL_470645, EPI_ISL_470646, EPI_ISL_470647, EPI_ISL_470648, EPI_ISL_470649, EPI_ISL_470650, EPI_ISL_623106, EPI_ISL_623107, EPI_ISL_623108, EPI_ISL_623110, EPI_ISL_623112, EPI_ISL_623113, EPI_ISL_623114, EPI_ISL_623116, EPI_ISL_623117, EPI_ISL_623118, EPI_ISL_623119, EPI_ISL_623120, EPI_ISL_623121, EPI_ISL_623122, EPI_ISL_623124, EPI_ISL_623125, EPI_ISL_623126, EPI_ISL_623127, EPI_ISL_623128, EPI_ISL_623129, EPI_ISL_623130, EPI_ISL_623131, EPI_ISL_623132, EPI_ISL_623133, EPI_ISL_623134, EPI_ISL_623136, EPI_ISL_623137, EPI_ISL_623138, EPI_ISL_623139, EPI_ISL_623140, EPI_ISL_623141, EPI_ISL_623142, EPI_ISL_623143, EPI_ISL_623144, EPI_ISL_623145, EPI_ISL_623146, EPI_ISL_623147, EPI_ISL_623148, EPI_ISL_623149, EPI_ISL_623150, EPI_ISL_623152, EPI_ISL_623153, EPI_ISL_623154, EPI_ISL_623156, EPI_ISL_623157, EPI_ISL_623158, EPI_ISL_623159, EPI_ISL_623160, EPI_ISL_623161, EPI_ISL_623162, EPI_ISL_623163, EPI_ISL_623164, EPI_ISL_623165, EPI_ISL_623166, EPI_ISL_623167, EPI_ISL_623168, EPI_ISL_623169, EPI_ISL_717791, EPI_ISL_717793, EPI_ISL_717806, EPI_ISL_717807, EPI_ISL_717808, EPI_ISL_717810, EPI_ISL_717811, EPI_ISL_717812, EPI_ISL_717813, EPI_ISL_717814, EPI_ISL_717815, EPI_ISL_717816, EPI_ISL_717817, EPI_ISL_717818, EPI_ISL_717819, EPI_ISL_717820, EPI_ISL_717821, EPI_ISL_717822, EPI_ISL_717823, EPI_ISL_717824, EPI_ISL_717825, EPI_ISL_717826, EPI_ISL_717827, EPI_ISL_717828, EPI_ISL_717829, EPI_ISL_717830, EPI_ISL_717831, EPI_ISL_717837, EPI_ISL_717838, EPI_ISL_717839, EPI_ISL_717840, EPI_ISL_717842, EPI_ISL_717843, EPI_ISL_717844, EPI_ISL_717845, EPI_ISL_717846, EPI_ISL_717847, EPI_ISL_717848, EPI_ISL_717849, EPI_ISL_717850, EPI_ISL_717851, EPI_ISL_717852, EPI_ISL_717853, EPI_ISL_717854, EPI_ISL_717855, EPI_ISL_717856, EPI_ISL_717857, EPI_ISL_717858, EPI_ISL_717859, EPI_ISL_717860, EPI_ISL_717861, EPI_ISL_717862, EPI_ISL_717863, EPI_ISL_717864, EPI_ISL_717865, EPI_ISL_717866, EPI_ISL_717867, EPI_ISL_717868, EPI_ISL_717869, EPI_ISL_717870, EPI_ISL_717871, EPI_ISL_717872, EPI_ISL_717873, EPI_ISL_717874, EPI_ISL_717875, EPI_ISL_717876, EPI_ISL_717877, EPI_ISL_717878, EPI_ISL_717879, EPI_ISL_717880, EPI_ISL_717881, EPI_ISL_717882, EPI_ISL_717883, EPI_ISL_717884, EPI_ISL_717885, EPI_ISL_717886, EPI_ISL_717887, EPI_ISL_717888, EPI_ISL_717889, EPI_ISL_717890, EPI_ISL_717891, EPI_ISL_717892, EPI_ISL_717893, EPI_ISL_717894, EPI_ISL_717895, EPI_ISL_717896, EPI_ISL_717897, EPI_ISL_717898, EPI_ISL_717920, EPI_ISL_717921, EPI_ISL_717922, EPI_ISL_717923, EPI_ISL_717924, EPI_ISL_717925, EPI_ISL_717926, EPI_ISL_717927, EPI_ISL_717928, EPI_ISL_717929, EPI_ISL_717930, EPI_ISL_717931, EPI_ISL_717932, EPI_ISL_717933, EPI_ISL_717934, EPI_ISL_717935, EPI_ISL_717936, EPI_ISL_717937, EPI_ISL_717938, EPI_ISL_717939, EPI_ISL_717940, EPI_ISL_717941, EPI_ISL_717942, EPI_ISL_717943, EPI_ISL_717944, EPI_ISL_717945, EPI_ISL_717946, EPI_ISL_717947, EPI_ISL_717948, EPI_ISL_717949, EPI_ISL_717950, EPI_ISL_717951, EPI_ISL_717952, EPI_ISL_717953, EPI_ISL_717954, EPI_ISL_717955, EPI_ISL_717956, EPI_ISL_717957, EPI_ISL_717959, EPI_ISL_717960, EPI_ISL_717961			
see above	Laboratorio de Virologia Molecular / UFRJ	Bioinformatics Laboratory / LNCC	Alexandra Gerber; Alexandra L Gerber; Amilcar Tanuri; Ana Paula Guimarães; Ana Paula de C Guimarães; Ana Tereza R de Vasconcelos; Andréa Cony Cavalcanti; CADDE-group; Carolina M Voloch; Carolina Voloch; Claudia dos Santos Rodrigues; Covid19-UFRJ Workgroup; Cynthia C Cardoso; Diana Mariani; Ester Cerdeira Sabino; Filipe Romero; Ingra Morales Claro; Jaqueline Goes de Jesus; Laboratorio Hermes Pardini; Laboratorio Simile; Luiz G P de Almeida; Luiz Gonzaga Paula de Almeida; Luís Cristóvão Pôrto; Mariane Talon; Nuno Rodrigues Faria; Orlando C. Ferreira; Otavio Bustrolini; Otavio J. Brustolini; Renato S Aguiar; Renato Santana Aguiar e Ana Tereza Vasconcelos; Ronaldo S Francisco Jr; Ronaldo da Silva F Jr; Ronaldo da Silva Francisco Junior; Terezinha M P P Castiñeira; Terezinha M P P Castiñeiras; Terezinha Marta Pereira; working group UFMG; working group UFRJ; Átila Duque Rossi
EPI_ISL_792397, EPI_ISL_792399, EPI_ISL_792400, EPI_ISL_792401	Laboratorio de Virología del Hospital de Niños Dr. Ricardo Gutierrez	Biocódices SA. on behalf of 'Proyecto Argentino Interinstitucional de genomica de SARS-CoV-2' (PAIS Consortium)	Acevedo; Alexay; Alvarez Lopez; Berros JM; C; Dopazo H; E; Gravis; Jacques; ME; Mistchenko; O; S; Zubrzycki J
EPI_ISL_792522, EPI_ISL_792523, EPI_ISL_792524	Laboratorio de Virología del Hospital de Niños Dr. Ricardo Gutierrez	Grupo de Genómica y Bioinformática del Instituto de Investigación de la Cadena Láctea CONICET-INTA on behalf of 'Proyecto Argentino Interinstitucional de genomica de SARS-CoV-2' (PAIS Consortium)	AF; AS; Acevedo; Alexay; Alvarez Lopez; Amadio; Aulicino; C; Eberhardt; G; Goya; Irazoqui; Jacques; König; M; ME; MF; MS; Mistchenko; Nabaes Jodar; O; P; S; Torres; Viegas, M.
EPI_ISL_430795, EPI_ISL_430799, EPI_ISL_430800, EPI_ISL_430801, EPI_ISL_430803, EPI_ISL_430804, EPI_ISL_430807, EPI_ISL_430809, EPI_ISL_430810, EPI_ISL_430811, EPI_ISL_430812, EPI_ISL_430813, EPI_ISL_430814, EPI_ISL_430815, EPI_ISL_430816, EPI_ISL_430817, EPI_ISL_430818, EPI_ISL_792117, EPI_ISL_792118, EPI_ISL_792119, EPI_ISL_792120, EPI_ISL_792121, EPI_ISL_792122, EPI_ISL_792123, EPI_ISL_792124, EPI_ISL_792125, EPI_ISL_792126, EPI_ISL_792127, EPI_ISL_792128, EPI_ISL_792129, EPI_ISL_792130, EPI_ISL_792131, EPI_ISL_792132, EPI_ISL_792133, EPI_ISL_792134, EPI_ISL_792135, EPI_ISL_792136, EPI_ISL_792137, EPI_ISL_792138, EPI_ISL_792139, EPI_ISL_792140, EPI_ISL_792141, EPI_ISL_792142, EPI_ISL_792143, EPI_ISL_792144, EPI_ISL_792145, EPI_ISL_792146, EPI_ISL_792147, EPI_ISL_792148, EPI_ISL_792149, EPI_ISL_792150, EPI_ISL_792151, EPI_ISL_792152, EPI_ISL_792153, EPI_ISL_792154, EPI_ISL_792155, EPI_ISL_792156, EPI_ISL_792157, EPI_ISL_792158, EPI_ISL_792159, EPI_ISL_792160, EPI_ISL_792161, EPI_ISL_792162, EPI_ISL_792163, EPI_ISL_792164, EPI_ISL_792165, EPI_ISL_792166, EPI_ISL_792167, EPI_ISL_792168, EPI_ISL_792169, EPI_ISL_792170, EPI_ISL_792171, EPI_ISL_792172, EPI_ISL_792173, EPI_ISL_792174, EPI_ISL_792175, EPI_ISL_792176, EPI_ISL_792177, EPI_ISL_792178, EPI_ISL_792179, EPI_ISL_792180, EPI_ISL_792181, EPI_ISL_792182, EPI_ISL_792183, EPI_ISL_792184, EPI_ISL_792185, EPI_ISL_792186, EPI_ISL_792187, EPI_ISL_792188, EPI_ISL_792189, EPI_ISL_792190, EPI_ISL_792191, EPI_ISL_792192, EPI_ISL_792193, EPI_ISL_792194, EPI_ISL_792196, EPI_ISL_792197, EPI_ISL_792198, EPI_ISL_792199, EPI_ISL_792200, EPI_ISL_792201, EPI_ISL_792202, EPI_ISL_792203, EPI_ISL_792204, EPI_ISL_792205, EPI_ISL_792206, EPI_ISL_792207, EPI_ISL_792208, EPI_ISL_792209, EPI_ISL_792210, EPI_ISL_792211, EPI_ISL_792212, EPI_ISL_792213, EPI_ISL_792214, EPI_ISL_792215, EPI_ISL_792216, EPI_ISL_792217, EPI_ISL_792218, EPI_ISL_792219, EPI_ISL_792220, EPI_ISL_792221, EPI_ISL_792222, EPI_ISL_792223, EPI_ISL_792224, EPI_ISL_792226, EPI_ISL_792227,			

EPI_ISL_792228, EPI_ISL_792229, EPI_ISL_792230, EPI_ISL_792231, EPI_ISL_792232, EPI_ISL_792233, EPI_ISL_792234, EPI_ISL_792235, EPI_ISL_792236, EPI_ISL_792237, EPI_ISL_792238, EPI_ISL_792239, EPI_ISL_792240, EPI_ISL_792241, EPI_ISL_792242, EPI_ISL_792243, EPI_ISL_792244, EPI_ISL_792245, EPI_ISL_792246, EPI_ISL_792247, EPI_ISL_792248, EPI_ISL_792249, EPI_ISL_792250, EPI_ISL_792251, EPI_ISL_792252, EPI_ISL_792253, EPI_ISL_792254, EPI_ISL_792255, EPI_ISL_792256, EPI_ISL_792257, EPI_ISL_792258, EPI_ISL_792259, EPI_ISL_792260, EPI_ISL_792261, EPI_ISL_792262, EPI_ISL_792263, EPI_ISL_792264, EPI_ISL_792265, EPI_ISL_792266, EPI_ISL_792267, EPI_ISL_792268, EPI_ISL_792269, EPI_ISL_792270, EPI_ISL_792271, EPI_ISL_792272, EPI_ISL_792273, EPI_ISL_792274, EPI_ISL_792275, EPI_ISL_792276, EPI_ISL_792277, EPI_ISL_792278, EPI_ISL_792279, EPI_ISL_792280, EPI_ISL_792281, EPI_ISL_792282, EPI_ISL_792283, EPI_ISL_792284, EPI_ISL_792285, EPI_ISL_792286, EPI_ISL_792287, EPI_ISL_792288, EPI_ISL_792289, EPI_ISL_792290			
see above	Laboratorio de Virología del Hospital de Niños Dr. Ricardo Gutierrez	Área de Secuenciación del Laboratorio de Virología del Hospital de Niños Dr. Ricardo Gutierrez on behalf of 'Proyecto Argentino Interinstitucional de genomica de SARS-CoV-2' (PAIS Consortium)	AS; Acevedo; Alexay; Alvarez Lopez; C; E; Gallino; Goya; Gravis; I; Jacques; LE; Lusso; ME; MI; MS; Mistchenko; Nabaes Jodar; Natale; O; Primost; S; Valinotto; Viegas, M.
EPI_ISL_671978	Laboratorio de Virología y Microbiología Molecular, Depto. de Microbiología, Facultad de Medicina, Universidad de El Salvador/INS-laboratorio de Ref. Ministerio de Salud	Laboratorio de Virología y Microbiología Molecular, Depto. de Microbiología, Facultad de Medicina, Universidad de El Salvador/INS-laboratorio de Ref. Ministerio de Salud	Rivera NR et al
EPI_ISL_672573	Laboratorio de Virología y Microbiología Molecular, Depto. de Microbiología, Facultad de Medicina, Universidad de El Salvador/INS-laboratorio de Ref. Ministerio de Salud. 1*: Dr. Noé Rigoberto Rivera, profesor del Departamento de Bioquímica e Investigador adjunto de la Sección de Virología y Microbiología Molecular;director de la Unidad de Investigaciones Científicas (UNICA), Facultad de Medicina Universidad de El Salvador,El Salvador C.A 1**: Dr. Carlos Alexander Ortega Pérez, Profesor del Departamento de Microbiología;investigador y Jefe de la Sección de Virología, Director Metodológico de la Unidad de Investigaciones Científicas(UNICA), Facultad de Medicina, Universidad de El Salvador, El Salvador C.A 2*: Dra. Xochitl Sandoval López:Directora e investigadora del Instituto Nacional de Salud (INS) El Salvador C.A 2** Dr. Hernandez Avila CarlosE Director de Gobernanza e investigador del Instituto Nacional de Salud (INS) El Salvador C.A	Laboratorio de Virología y Microbiología Molecular, Depto. de Microbiología, Facultad de Medicina, Universidad de El Salvador/INS-laboratorio de Ref. Ministerio de Salud	Rivera NR et al
EPI_ISL_672572	Laboratorio de Virología y Microbiología Molecular, Depto. de Microbiología, Facultad de Medicina, Universidad de El Salvador/INS-laboratorio de Ref. Ministerio de Salud. 1*: Dr. Noé Rigoberto Rivera, profesor del Departamento de Bioquímica e Investigador adjunto de la Sección de Virología y Microbiología Molecular; director de la Unidad de Investigaciones Científicas (UNICA), Facultad de Medicina Universidad de El Salvador, El Salvador C.A 1**: Dr. Carlos Alexander Ortega Pérez, Profesor del Departamento de Microbiología; investigador y Jefe de la Sección de Virología, Director Metodológico de la Unidad de Investigaciones Científicas (UNICA), Facultad de Medicina, Universidad de El Salvador, El Salvador C.A 2*: Dra. Xochitl Sandoval López: Directora e investigadora del Instituto Nacional de Salud (INS) El Salvador C.A 2** Dr. Hernandez Avila Carlos E Director de Gobernanza e investigador del Instituto Nacional de Salud (INS) El Salvador C.A	Laboratorio de Virología y Microbiología Molecular, Depto. de Microbiología, Facultad de Medicina, Universidad de El Salvador/INS-laboratorio de Ref. Ministerio de Salud.	Rivera NR et al
EPI_ISL_671974, EPI_ISL_672570	Laboratorio de Virología y Microbiología Molecular, Depto. de Microbiología, Facultad de Medicina, Universidad de El Salvador/INS-laboratorio de Ref. Ministerio de Salud	Laboratorio de Virología y Microbiología Molecular, Depto. de Microbiología, Facultad de Medicina, Universidad de El Salvador/INS-laboratorio de Ref. Ministerio de Salud	Carlos Hernández Ávila; Ortega Pérez CA et al; Ortega-Pérez C A; Rivera NR; Xochitl Sandoval López
EPI_ISL_672012	Laboratorio de Virología y Microbiología Molecular, Depto. de Microbiología, Facultad de Medicina, Universidad de El Salvador/INS-laboratorio de Ref. Ministerio de Salud. 1*: Dr. Noé Rigoberto Rivera, profesor del Departamento de Bioquímica e Investigador adjunto de la Sección de Virología y Microbiología Molecular; director de la Unidad de Investigaciones Científicas (UNICA), Facultad de Medicina Universidad de El Salvador, El Salvador C.A 1**: Dr. Carlos Alexander Ortega Pérez, Profesor del Departamento de Microbiología; investigador y Jefe de la Sección de Virología, Director Metodológico de la Unidad de Investigaciones Científicas (UNICA), Facultad de Medicina, Universidad de El Salvador, El Salvador C.A 2*: Dra. Xochitl Sandoval López: Directora e investigadora del Instituto Nacional de Salud (INS) El Salvador C.A 2** Dr. Hernandez Avila Carlos E Director de Gobernanza e investigador del Instituto Nacional de Salud (INS) El Salvador C.A	Laboratorio de Virología y Microbiología Molecular, Depto. de Microbiología, Facultad de Medicina, Universidad de El Salvador/INS-laboratorio de Ref. Ministerio de Salud.	Ortega Pérez CA et al
EPI_ISL_755304	Laboratorio de Virología, Centro de Biotecnología Acuícola, Universidad de Santiato de Chile	Center for Mathematical Modeling and Center for Genome Regulation. Santiago, Chile	A. Sandino; Allende ML; Arriagada G; Bastias M; Bustos F; Castro E; González M; M; Maass A; Meneses C.; Montecino; Orellana A; Reyes-López F.; Sanhueza D; Travisany D
EPI_ISL_792291, EPI_ISL_792292, EPI_ISL_792293, EPI_ISL_792294, EPI_ISL_792295, EPI_ISL_792296, EPI_ISL_792297, EPI_ISL_792298			
see above	Laboratorio de genética y biología molecular del Hospital de trauma y emergencia Dr Federico Abete	Área de Secuenciación del Laboratorio de Virología del Hospital de Niños Dr. Ricardo Gutierrez on behalf of 'Proyecto Argentino Interinstitucional de genomica de SARS-CoV-2' (PAIS Consortium)	Gallino; Goya; I; LE; Lusso; MI; MS; Nabaes Jodar; Natale; Primost; S; Valinotto; Viegas, M.
EPI_ISL_792375, EPI_ISL_792376, EPI_ISL_792377, EPI_ISL_792378, EPI_ISL_792379, EPI_ISL_792380, EPI_ISL_792381, EPI_ISL_792382, EPI_ISL_792383, EPI_ISL_792384, EPI_ISL_792385			
see above	Laboratorio de salud pública, Facultad de Ciencias Exactas, Universidad Nacional de La Plata	Área de Secuenciación del Laboratorio de Virología del Hospital de Niños Dr. Ricardo Gutierrez on behalf of 'Proyecto Argentino Interinstitucional de genomica de SARS-CoV-2' (PAIS Consortium)	A; Angeletti; Cordero; Goya; LE; Lusso; MI; MS; Nabaes Jodar; Nadalich; Natale; R; S; Toro; V; Valinotto; Viegas, M.
EPI_ISL_792299, EPI_ISL_792300	Laboratorio del Hospital Alemán	Área de Secuenciación del Laboratorio de Virología del Hospital de Niños Dr. Ricardo Gutierrez on behalf of 'Proyecto Argentino Interinstitucional de genomica de SARS-CoV-2' (PAIS Consortium)	Della Latta; E; García Allende; Goya; Ibañez; LE; Lusso; MI; MP; MS; N; Nabaes Jodar; Natale; S; Valinotto; Viegas, M.

EPI_ISL_792306, EPI_ISL_792307, EPI_ISL_792308, EPI_ISL_792309, EPI_ISL_792310, EPI_ISL_792311, EPI_ISL_792312, EPI_ISL_792313, EPI_ISL_792314, EPI_ISL_792315, EPI_ISL_792316, EPI_ISL_792317, EPI_ISL_792318, EPI_ISL_792319			
see above	Laboratorio del Hospital El Cruce Dr. Néstor C. Kirchner	Área de Secuenciación del Laboratorio de Virología del Hospital de Niños Dr. Ricardo Gutierrez on behalf of 'Proyecto Argentino Interinstitucional de genomica de SARS-CoV-2' (PAIS Consortium)	Goya; LE; Lusso; M; MI; MS; Nabaes Jodar; Natale; Rahhal; S; Valinotto; Viegas, M.; Zubieta
EPI_ISL_792371, EPI_ISL_792372, EPI_ISL_792373, EPI_ISL_792374	Laboratorio del Hospital Interzonal General de Agudos Eva Perón	Área de Secuenciación del Laboratorio de Virología del Hospital de Niños Dr. Ricardo Gutierrez on behalf of 'Proyecto Argentino Interinstitucional de genomica de SARS-CoV-2' (PAIS Consortium)	C; Carulla; Goya; Kairiyama; LE; Lusso; M; MI; MS; Nabaes Jodar; Natale; Pengue; Piñeyro C; S; Valinotto; Viegas, M.
EPI_ISL_792525	Laboratorio del Hospital Interzonal General de Agudos Evita	Grupo de Genómica y Bioinformática del Instituto de Investigación de la Cadena Láctea CONICET-INTA on behalf of 'Proyecto Argentino Interinstitucional de genomica de SARS-CoV-2' (PAIS Consortium)	AF; Alexay; Amadio; Aulicino; C; Desimone; E; Eberhardt; G; Goya; Grossi; I; Irazoqui; König; L; Luczac; M; MF; MS; Musto; Nabaes Jodar; O; P; S; Serrano; Torres; Viegas, M.
EPI_ISL_792333, EPI_ISL_792334, EPI_ISL_792335, EPI_ISL_792336, EPI_ISL_792337, EPI_ISL_792338, EPI_ISL_792339, EPI_ISL_792340, EPI_ISL_792341, EPI_ISL_792342, EPI_ISL_792343, EPI_ISL_792344, EPI_ISL_792345, EPI_ISL_792346, EPI_ISL_792347, EPI_ISL_792348, EPI_ISL_792349, EPI_ISL_792350			
see above	Laboratorio del Hospital Interzonal General de Agudos Evita	Área de Secuenciación del Laboratorio de Virología del Hospital de Niños Dr. Ricardo Gutierrez on behalf of 'Proyecto Argentino Interinstitucional de genomica de SARS-CoV-2' (PAIS Consortium)	A; Desimone; E; Goya; Grossi; I; L; LE; Luczac; Lusso; MI; MS; Musto; Nabaes Jodar; Natale; O; S; Serrano; Valinotto; Viegas, M.
EPI_ISL_792444, EPI_ISL_792447, EPI_ISL_792450, EPI_ISL_792451, EPI_ISL_792452, EPI_ISL_792454, EPI_ISL_792455, EPI_ISL_792459, EPI_ISL_792461, EPI_ISL_792462, EPI_ISL_792463, EPI_ISL_792465, EPI_ISL_792467, EPI_ISL_792469, EPI_ISL_792470, EPI_ISL_792471, EPI_ISL_792473			
see above	Laboratorio del Hospital Regional Ushuaia Gdor. Ernesto Campos	Hospital Regional Ushuaia - Centro Austral De Investigaciones Científicas - Universidad Nacional De Tierra Del Fuego on behalf of 'Proyecto Argentino Interinstitucional de genomica de SARS-CoV-2' (PAIS Consortium)	Boutureira, MF.; CA; CB; CF; Castro; Ceballos; Cáceres; De Roccis; F; G; Gallego; Gramundi; ID; Nardi; SB; SG; Yulan
EPI_ISL_476220, EPI_ISL_476221	Laboratory Fleury	Instituto de Medicina Tropical da Univesidade de São Paulo	Camila Alves Maia da Silva; Darlan da Silva Candido; Erika Regina Manuli; Ester Sabino; Flavia Cristina da Silva Sales; Giula Magalhaes Ferreira; Jaqueline Goes de Jesus; Julien Theze; Mariana Severo Ramundo; Nuno Faria; Samples: Celso Granato; Sequencing: Ingra Morales Claro; Thais de Moura Coletti
EPI_ISL_417525	Laboratory Medicine	Department of Laboratory Medicine, Lin-Kou Chang Gung Memorial Hospital, Taoyuan, Taiwan	Cheng-Hsun Chiu; Cheng-Ta Yang; Chung-Guei Huang; Guang-Wu Chen; Kuo-Chien Tsao; Kuo-Ming Lee; Mei-Jen Hsiao; Peng-Nien Huang; Po-Wei Huang; Shin-Ru Shih; Shu-Li Yang; Yi-Chun Liu; Yu-Nong Gong
EPI_ISL_455474, EPI_ISL_455477	Laboratory for Respiratory Viruses, Cantacuzino National Military-Medical Institute for Research and Development	Cantacuzino Institute	A.Cretu; L.Ustea; M.Lazar; T.Durfee; Tim Durfee
EPI_ISL_536399	Laboratory of Immunovirology. Universidad de Antioquia	Instituto Nacional de Salud - Unidad de Secuenciación y Genómica	Carlos Franco-Muñoz; Diego Álvarez-Diaz and Marcela Mercado-Reyes; Francisco J. Díaz; Katherine Laiton-Donato; Lizdany Flórez; Wbeimar Aguilar-Jimenez
EPI_ISL_452329	Laboratory of Infectious Diseases Center of Beijing Ditan Hospital	Laboratory of Infectious Diseases Center of Beijing Ditan Hospital	Chengjie Jie; Fengting Yu; Linghang Wang; Liting Yan; Siyuan Yang; Yunxia Tang
EPI_ISL_434475, EPI_ISL_437910	Laboratory of Microbiology, Medical School, National and Kapodistrian University of Athens	Laboratory of Biology, Department of Medicine, Democritus University of Thrace	Bampali, M.; Dovrolis, N.; Froukala, E.; Gatzidou, E.; Kassela K.; N. and Karakasiliotis, I.; Spanakis; Stavropoulou, A.; Tsakris, A.; Veletza, S.
EPI_ISL_613563, EPI_ISL_613564, EPI_ISL_613707, EPI_ISL_613708	Laboratory of Molecular Biology, Blood Center of Ribeirão Preto	Laboratory of Molecular Biology, Blood Center of Ribeirão Preto, Faculty of Medicine of Ribeirão Preto, University of São Paulo	Aparecida Y Yamamoto; Diego Villa Clé; Dimas T Covas; Elaine V Santos; Evandra S Rodrigues; Glauco de Carvalho Pereira; Joilson Xavier; Luiz CJ Alcantara; Marta Giovanetti; Rodrigo T Calado; Simone Kashima; Svetoslav N Slavov; Talita Adelino; Vagner Fonseca
EPI_ISL_613709, EPI_ISL_613951, EPI_ISL_613964	Laboratory of Molecular Biology, Blood Center of Ribeirão Preto, Faculty of Medicine of Ribeirão Preto, University of São Paulo	Laboratory of Molecular Biology, Blood Center of Ribeirão Preto, Faculty of Medicine of Ribeirão Preto, University of São Paulo	Aparecida Y Yamamoto; Diego Villa Clé; Dimas T Covas; Elaine V Santos; Evandra S Rodrigues; Glauco de Carvalho Pereira; Joilson Xavier; Luiz CJ Alcantara; Marta Giovanetti; Rodrigo T Calado; Simone Kashima; Svetoslav N Slavov; Talita Adelino; Vagner Fonseca
EPI_ISL_451644	Laboratory of Molecular Biology, Diagnostyka sp. z o.o.	Laboratory of Recombinant Vaccines	Anna Piotrowska-Mietelska; Boguslaw Szewczyk; Krystyna Bienkowska-Szewczyk; Lukasz Rabalski; Maciej Kosinski
EPI_ISL_625673, EPI_ISL_625674, EPI_ISL_625675, EPI_ISL_625676, EPI_ISL_625677, EPI_ISL_625678, EPI_ISL_625679, EPI_ISL_625680, EPI_ISL_625681, EPI_ISL_625682			
see above	Laboratory of Molecular Medicine, University of Magallanes	Centro Asistencial Docente y de Investigacion, Universidad de Magallanes	Diego Alvarez; Jacqueline Aldridge; Jorge Gonzalez; Marcelo Navarrete
EPI_ISL_415658, EPI_ISL_415660, EPI_ISL_415661, EPI_ISL_801539, EPI_ISL_801540, EPI_ISL_801541, EPI_ISL_801542, EPI_ISL_801543, EPI_ISL_801544, EPI_ISL_801545, EPI_ISL_801546, EPI_ISL_801548, EPI_ISL_801549, EPI_ISL_801550, EPI_ISL_801551, EPI_ISL_801552, EPI_ISL_801553, EPI_ISL_801554, EPI_ISL_801555, EPI_ISL_801556, EPI_ISL_801557, EPI_ISL_801558, EPI_ISL_801559, EPI_ISL_801560, EPI_ISL_801561, EPI_ISL_801562, EPI_ISL_801563, EPI_ISL_801564, EPI_ISL_801565, EPI_ISL_801566, EPI_ISL_801567, EPI_ISL_801568, EPI_ISL_801569, EPI_ISL_801570, EPI_ISL_801571, EPI_ISL_801572, EPI_ISL_801573, EPI_ISL_801574, EPI_ISL_801575, EPI_ISL_801576, EPI_ISL_801577, EPI_ISL_801578, EPI_ISL_801579, EPI_ISL_801580, EPI_ISL_801581, EPI_ISL_801582, EPI_ISL_801583, EPI_ISL_801584, EPI_ISL_801585, EPI_ISL_801586, EPI_ISL_801587, EPI_ISL_801588, EPI_ISL_801589, EPI_ISL_801590, EPI_ISL_801591, EPI_ISL_801592, EPI_ISL_801593, EPI_ISL_801594, EPI_ISL_801595, EPI_ISL_801596, EPI_ISL_801597, EPI_ISL_801598, EPI_ISL_801599, EPI_ISL_801600, EPI_ISL_801601, EPI_ISL_801602, EPI_ISL_801603, EPI_ISL_801604, EPI_ISL_801605, EPI_ISL_801606, EPI_ISL_801607, EPI_ISL_801608, EPI_ISL_801609, EPI_ISL_801610, EPI_ISL_801611, EPI_ISL_801612, EPI_ISL_801613, EPI_ISL_801614, EPI_ISL_801615, EPI_ISL_801616, EPI_ISL_801617, EPI_ISL_801618, EPI_ISL_801619, EPI_ISL_801620, EPI_ISL_801621, EPI_ISL_801622, EPI_ISL_801623, EPI_ISL_801624, EPI_ISL_801625, EPI_ISL_801626, EPI_ISL_801627, EPI_ISL_801628, EPI_ISL_801629, EPI_ISL_801630, EPI_ISL_801631, EPI_ISL_801632, EPI_ISL_801633, EPI_ISL_801634, EPI_ISL_801635, EPI_ISL_801636, EPI_ISL_801637, EPI_ISL_801638, EPI_ISL_801639, EPI_ISL_801640, EPI_ISL_801641, EPI_ISL_801642, EPI_ISL_801643, EPI_ISL_801644, EPI_ISL_801645, EPI_ISL_801646, EPI_ISL_801647, EPI_ISL_801648, EPI_ISL_801649, EPI_ISL_801650, EPI_ISL_801651, EPI_ISL_801652, EPI_ISL_801653, EPI_ISL_801654, EPI_ISL_801655, EPI_ISL_801656, EPI_ISL_801657, EPI_ISL_801658, EPI_ISL_801659, EPI_ISL_801660, EPI_ISL_801661, EPI_ISL_801662, EPI_ISL_801663, EPI_ISL_801664, EPI_ISL_801665, EPI_ISL_801666, EPI_ISL_801667, EPI_ISL_801668, EPI_ISL_801669, EPI_ISL_801670, EPI_ISL_801671, EPI_ISL_801672, EPI_ISL_801673, EPI_ISL_801674, EPI_ISL_801675, EPI_ISL_801676, EPI_ISL_801677, EPI_ISL_801678, EPI_ISL_801679, EPI_ISL_801680, EPI_ISL_801681, EPI_ISL_801682, EPI_ISL_801683, EPI_ISL_801684, EPI_ISL_801685, EPI_ISL_801686, EPI_ISL_801687, EPI_ISL_801688, EPI_ISL_801689, EPI_ISL_801690, EPI_ISL_801691, EPI_ISL_801692, EPI_ISL_801693, EPI_ISL_801694, EPI_ISL_801695, EPI_ISL_801696, EPI_ISL_801697, EPI_ISL_801698, EPI_ISL_801699, EPI_ISL_801700, EPI_ISL_801701, EPI_ISL_801702, EPI_ISL_801703, EPI_ISL_801704, EPI_ISL_801705, EPI_ISL_801706, EPI_ISL_801707, EPI_ISL_801708, EPI_ISL_801709, EPI_ISL_801710, EPI_ISL_801711, EPI_ISL_801712, EPI_ISL_801713, EPI_ISL_801714, EPI_ISL_801715, EPI_ISL_801716, EPI_ISL_801717, EPI_ISL_801718, EPI_ISL_801719, EPI_ISL_801720, EPI_ISL_801721, EPI_ISL_801722, EPI_ISL_801723, EPI_ISL_801724, EPI_ISL_801725, EPI_ISL_801726, EPI_ISL_801727, EPI_ISL_801728, EPI_ISL_801729, EPI_ISL_801730, EPI_ISL_801731, EPI_ISL_801732, EPI_ISL_801733, EPI_ISL_801734, EPI_ISL_801735, EPI_ISL_801736, EPI_ISL_801737, EPI_ISL_801738, EPI_ISL_801739, EPI_ISL_801740, EPI_ISL_801741, EPI_ISL_801742, EPI_ISL_801743, EPI_ISL_801744, EPI_ISL_801745, EPI_ISL_801746, EPI_ISL_801747, EPI_ISL_801748, EPI_ISL_801749, EPI_ISL_801750, EPI_ISL_801751, EPI_ISL_801752, EPI_ISL_801753, EPI_ISL_801754, EPI_ISL_801755, EPI_ISL_801756, EPI_ISL_801757, EPI_ISL_801758, EPI_ISL_801759, EPI_ISL_801760, EPI_ISL_801761, EPI_ISL_801762, EPI_ISL_801763, EPI_ISL_801764, EPI_ISL_801765, EPI_ISL_801766, EPI_ISL_801767, EPI_ISL_801768, EPI_ISL_801769, EPI_ISL_801770, EPI_ISL_801771, EPI_ISL_801772, EPI_ISL_801773, EPI_ISL_801774, EPI_ISL_801775, EPI_ISL_801776, EPI_ISL_801777, EPI_ISL_801778, EPI_ISL_801779, EPI_ISL_801780, EPI_ISL_801781, EPI_ISL_801782, EPI_ISL_801783, EPI_ISL_801784, EPI_ISL_801785, EPI_ISL_801786, EPI_ISL_801787, EPI_ISL_801788, EPI_ISL_801789, EPI_ISL_801790, EPI_ISL_801791, EPI_ISL_801792, EPI_ISL_801793, EPI_ISL_801794, EPI_ISL_801795, EPI_ISL_801796, EPI_ISL_801797, EPI_ISL_801798, EPI_ISL_801799, EPI_ISL_801800, EPI_ISL_801801, EPI_ISL_801802, EPI_ISL_801803, EPI_ISL_801804, EPI_ISL_801805, EPI_ISL_801806, EPI_ISL_801807, EPI_ISL_801808, EPI_ISL_801809, EPI_ISL_801810, EPI_ISL_801811, EPI_ISL_801812, EPI_ISL_801813, EPI_ISL_801814, EPI_ISL_801815, EPI_ISL_801816, EPI_ISL_801817, EPI_ISL_801818, EPI_ISL_801819, EPI_ISL_801820, EPI_ISL_801821, EPI_ISL_801822, EPI_ISL_801823, EPI_ISL_801824, EPI_ISL_801825, EPI_ISL_801826, EPI_ISL_801827, EPI_ISL_801828, EPI_ISL_801829, EPI_ISL_801830, EPI_ISL_801831, EPI_ISL_801832, EPI_ISL_801833, EPI_ISL_801834, EPI_ISL_801835, EPI_ISL_801836, EPI_ISL_801837, EPI_ISL_801838, EPI_ISL_801839, EPI_ISL_801840, EPI_ISL_801841, EPI_ISL_801842, EPI_ISL_801843, EPI_ISL_801844, EPI_ISL_801845, EPI_ISL_801846, EPI_ISL_801847, EPI_ISL_801848, EPI_ISL_801849, EPI_ISL_801850, EPI_ISL_801851, EPI_ISL_801852, EPI_ISL_801853, EPI_ISL_801854, EPI_ISL_801855, EPI_ISL_801856, EPI_ISL_801857, EPI_ISL_801858, EPI_ISL_801859, EPI_ISL_801860			
see above	Laboratory of Molecular Virology, Pontificia Universidad Católica de Chile	MSHS Pathogen Surveillance Program	Adolfo Garcia-Sastre; Adriana van De Guchte; Ajay Obla; Aldo Gaggero; Ana Maria Contreras; Ana S. Gonzalez-Reiche; Ana Silvia Gonzalez-Reiche; Bremy Alburquerque; Carlos Palma; Constanza Maldonado; Edward C. Holmes; Eileen Serrano; Erick Salinas; Fernando Valiente; Hala Alshammary; Harm van Bakel; Jayeeta Dutta; Jorge Levican; Juan Soto; Leonardo I. Almonacid; M. Belen Leyton; Manuel Ampuero; Marcela Ferres; Matthew Hernandez; Matthew M. Hernandez; Melissa Smith; Mitchell Sullivan; Pablo Vial; Rafael A. Medina; Rafael A. Medina.; Robert Sebra; Shwetha Hara Sridhar; Shwetha Sridhar Hara; Tamara Garcia; Tamara García-Salum; Viviana Simon; Ying-Chih Wang; Zenab Khan
EPI_ISL_456071, EPI_ISL_456072, EPI_ISL_456073, EPI_ISL_456074, EPI_ISL_456075, EPI_ISL_456079, EPI_ISL_456080, EPI_ISL_456081, EPI_ISL_456084, EPI_ISL_456085, EPI_ISL_456086, EPI_ISL_456087, EPI_ISL_456089, EPI_ISL_456090, EPI_ISL_456091, EPI_ISL_456092, EPI_ISL_456093, EPI_ISL_456094, EPI_ISL_456095, EPI_ISL_456096, EPI_ISL_456097, EPI_ISL_456098, EPI_ISL_456099, EPI_ISL_456100, EPI_ISL_456101, EPI_ISL_456102, EPI_ISL_456103, EPI_ISL_456104, EPI_ISL_456105, EPI_ISL_456106, EPI_ISL_467344, EPI_ISL_467345, EPI_ISL_467346, EPI_ISL_467347, EPI_ISL_467348, EPI_ISL_467349, EPI_ISL_467350, EPI_ISL_467351, EPI_ISL_467352, EPI_ISL_467353, EPI_ISL_467354, EPI_ISL_467355, EPI_ISL_467356, EPI_ISL_467357, EPI_ISL_467358, EPI_ISL_467359, EPI_ISL_467360, EPI_ISL_467361, EPI_ISL_467362, EPI_ISL_467363, EPI_ISL_467364, EPI_ISL_467365, EPI_ISL_467366, EPI_ISL_467367, EPI_ISL_467368, EPI_ISL_467369, EPI_ISL_467370, EPI_ISL_467371, EPI_ISL_541347, EPI_ISL_541348, EPI_ISL_541349, EPI_ISL_541350, EPI_ISL_541351, EPI_ISL_541352, EPI_ISL_541353, EPI_ISL_541354, EPI_ISL_541355, EPI_ISL_541356, EPI_ISL_541357, EPI_ISL_541358, EPI_ISL_541359, EPI_ISL_541360, EPI_ISL_541361, EPI_ISL_541362, EPI_ISL_541363, EPI_ISL_541364, EPI_ISL_541365, EPI_ISL_541366, EPI_ISL_541367, EPI_ISL_541368, EPI_ISL_541369			
see above	Laboratory of Respiratory Viruses and Measles, Oswaldo Cruz Institute, FIOCRUZ	Laboratory of Respiratory Viruses and Measles, Oswaldo Cruz Institute, FIOCRUZ	Aline Mattos; Ana Carolina Mendonça; Anna Carolina Paixão; Braulia Caetano; Cristiana Garcia; Fernando Motta; Jonathan Lopes; Luciana Appolinario; Maria Ogrzewalska; Marilda Siqueira; Milene Miranda; Paola Resende

EPI_ISL_454582	Laboratory of virology, National Center of Expertise	Laboratory of molecular-genetic research, National Center of Expertise, Kazakhstan National Center for Biotechnology, Kazakhstan	; Abdaliyev Askar; Akhmetollayev Ilyas; Amirgazin Asylulan; Aushakhmetova Zabira; Kalendar Ruslan; Lutsay Viktoriya; Rakhmetova Akbota; Ramankulov Yerlan; Shevtsov Alexandr
EPI_ISL_415105	Laboratório Central de Saúde Pública Professor Gonçalo Moniz - LACEN/BA	Instituto Oswaldo Cruz FIOCRUZ - Laboratory of Respiratory Viruses and Measles (LVRS)	Aline Mattos; Allison Fabri; Braulia Caetano; Cristiana Garcia; Fernando Motta; Joilson Xavier; Jonathan Lopes; Luciana Appolinario; Maria Nóbrega; Maria Ogrzewalska; Marilda Siqueira; Milene Miranda; Paola Resende; Sunando Roy
EPI_ISL_801397, EPI_ISL_801398, EPI_ISL_801399, EPI_ISL_801400, EPI_ISL_801401, EPI_ISL_801402, EPI_ISL_801403			
see above	Laboratório Central de Saúde Pública do Amazonas - LACEN-AM	Laboratorio de Ecologia de Doencas Transmissiveis na Amazonia, Instituto Leonidas e Maria Deane - Fiocruz Amazonia	André Corado; Debora Duarte; Felipe Naveca; Fernanda Nascimento; George Silva; Luciana Gonçalves; Maria Júlia Brandão; Michele Jesus; Valdinete Nascimento; Victor Souza; Ágatha Costa
EPI_ISL_693220, EPI_ISL_693223, EPI_ISL_693224, EPI_ISL_693243	Laboratório Municipal de Piracicaba	Instituto Adolfo Lutz, Interdiciplinary Procedures Center, Strategic Laboratory	Claudia Regina Gonçalves; Claudio Tavares Sacchi; Erica Valessa Ramos Gomes; Karoline Rodrigues Campos
EPI_ISL_636737, EPI_ISL_636834, EPI_ISL_636835, EPI_ISL_636836, EPI_ISL_636837, EPI_ISL_636838	Laboratório de Imunofarmacologia - Instituto Oswaldo Cruz	Laboratório de Imunofarmacologia - Instituto Oswaldo Cruz	A.D.; C.Q.; De Paula; F.B.; Ferreira; Fintelman-Rodrigues, N.; M.A. and Sacramento; Saraiva; Souza; T.M.
EPI_ISL_770551, EPI_ISL_770552, EPI_ISL_770553, EPI_ISL_770554, EPI_ISL_770555, EPI_ISL_770556, EPI_ISL_770557, EPI_ISL_770558, EPI_ISL_770559, EPI_ISL_770560, EPI_ISL_770561, EPI_ISL_770562, EPI_ISL_770563, EPI_ISL_770564, EPI_ISL_770565, EPI_ISL_770566, EPI_ISL_770567, EPI_ISL_770568, EPI_ISL_770569, EPI_ISL_770570, EPI_ISL_770571, EPI_ISL_770572, EPI_ISL_770573, EPI_ISL_770574, EPI_ISL_770575, EPI_ISL_770576, EPI_ISL_770577, EPI_ISL_770578, EPI_ISL_770579, EPI_ISL_770580, EPI_ISL_770581, EPI_ISL_770582, EPI_ISL_770583, EPI_ISL_770584, EPI_ISL_770585, EPI_ISL_770586, EPI_ISL_770587, EPI_ISL_770588, EPI_ISL_770589, EPI_ISL_770590, EPI_ISL_770591, EPI_ISL_770592, EPI_ISL_770593, EPI_ISL_770594, EPI_ISL_770595, EPI_ISL_770596, EPI_ISL_770597, EPI_ISL_770598, EPI_ISL_770599, EPI_ISL_770600, EPI_ISL_770601, EPI_ISL_770602, EPI_ISL_770603, EPI_ISL_770604, EPI_ISL_770605, EPI_ISL_770606, EPI_ISL_770607, EPI_ISL_770608, EPI_ISL_770609, EPI_ISL_770610, EPI_ISL_770611, EPI_ISL_770612, EPI_ISL_770613, EPI_ISL_770614, EPI_ISL_770615, EPI_ISL_770616, EPI_ISL_770617, EPI_ISL_770618, EPI_ISL_770619, EPI_ISL_770620, EPI_ISL_770621, EPI_ISL_770622, EPI_ISL_770623, EPI_ISL_770624, EPI_ISL_770625, EPI_ISL_770626, EPI_ISL_770627, EPI_ISL_770628, EPI_ISL_770629, EPI_ISL_770630, EPI_ISL_779155, EPI_ISL_779156, EPI_ISL_779157, EPI_ISL_779158, EPI_ISL_779159, EPI_ISL_779160, EPI_ISL_779161, EPI_ISL_779162, EPI_ISL_779163, EPI_ISL_779164, EPI_ISL_779165, EPI_ISL_779166, EPI_ISL_779167, EPI_ISL_779168			
see above	Laboratório de Microbiologia Molecular - Universidade FEEVALE	Bioinformatics Laboratory / LNCC	Alana Witt Hansen; Alessandra Pavan Lamarca da Silva; Alexandra L Gerber; Ana Karolina Eisen Antunes; Ana Luiza Ziulkoski; Ana Paula de C Guimarães; Ana Tereza R de Vasconcelos; Bruna Hermann; Fagner Henrique Heldt; Felipe Benites; Fernando Rosado Spilki; Juliana Schons; Juliane Deise Fleck; Karoline Schallenberger; Larissa Mallmann; Luiz G P de Almeida; Matheus Nunes Weber; Meriane Demoliner; Paula Rodrigues de Almeida; Ronaldo da Silva F Jr; Vyctoria Goes
EPI_ISL_831474, EPI_ISL_831645, EPI_ISL_831646, EPI_ISL_831660, EPI_ISL_831678, EPI_ISL_831681, EPI_ISL_831683, EPI_ISL_831685, EPI_ISL_831688, EPI_ISL_831689, EPI_ISL_831892, EPI_ISL_831898, EPI_ISL_831913, EPI_ISL_831938, EPI_ISL_831939, EPI_ISL_831940, EPI_ISL_832009, EPI_ISL_832010, EPI_ISL_832011, EPI_ISL_832012, EPI_ISL_832013			
see above	Laboratório de Microbiologia Molecular - Universidade FEEVALE	Universidade Federal de Ciências da Saúde de Porto Alegre	Amanda de Menezes Mayer; Carla Andretta Moreira Neves; Claudia Elizabeth Thompson; Fernando Rosado Spilki; Gabriel Dickin Caldana; Gabriela Bettella Cybis; Lívia Kmetzsch; Patrícia Aline Gröhs Ferrareze; Ricardo Ariel Zimerman; Vinícius Bonetti Franceschi
EPI_ISL_476152, EPI_ISL_476155, EPI_ISL_476156, EPI_ISL_476157, EPI_ISL_476158, EPI_ISL_476159, EPI_ISL_476160, EPI_ISL_476161, EPI_ISL_476162, EPI_ISL_476163, EPI_ISL_476164, EPI_ISL_476165, EPI_ISL_476167, EPI_ISL_476170, EPI_ISL_476337, EPI_ISL_476338, EPI_ISL_476340, EPI_ISL_476341, EPI_ISL_476343, EPI_ISL_476345, EPI_ISL_476346, EPI_ISL_476347, EPI_ISL_476349, EPI_ISL_476388, EPI_ISL_476389, EPI_ISL_476390, EPI_ISL_476391, EPI_ISL_476394, EPI_ISL_476395, EPI_ISL_476396, EPI_ISL_476397, EPI_ISL_476398, EPI_ISL_476399, EPI_ISL_476400, EPI_ISL_476408, EPI_ISL_476410, EPI_ISL_476411, EPI_ISL_476412, EPI_ISL_476413, EPI_ISL_476414, EPI_ISL_476415, EPI_ISL_476417, EPI_ISL_476418, EPI_ISL_476419, EPI_ISL_476422, EPI_ISL_476423			
see above	Laboratório de Patologia Clínica - UNICAMP	Laboratório de Estudos de Vírus Emergentes - UNICAMP	Angelica Schreiber; Camila Simeoni; Darlan da Silva Candido; Jaqueline Goes Jesus e William Marciel de Souza; José Luiz Proença-Modena; Julia Forato; Julien Theze; Luiz Gonzaga; Magnun Nueldo Nunes dos Santos; Marcilio Jorge Fumagalli; Mariene Ribeiro Amorim; Nuno Rodrigues Faria
EPI_ISL_541397, EPI_ISL_541399	Laboratório de Virologia Comparada e Ambiental- LVCA-IOC	Laboratory of Respiratory Viruses and Measles, Oswaldo Cruz Institute, FIOCRUZ	Ana Carolina Mendonça; Camille Ferreira Mannarino; Fernando Motta; Luciana Appolinario; Marilda Siqueira; Marize Pereira Miagostovich; Paola Resende; Tatiana Prado; Tulio Machado Fumian
EPI_ISL_437090, EPI_ISL_437095	Latvijas Infektoloijas centrs	Latvian Biomedical Research and Study Centre	Ivars Silamielis; Jeena Storoženko; Jnis Kloviš; Kaspars Megnis; Monta Ustinova; Oksana Savicka; Tatjana Kolupajeva; Uga Dumpis; Vita Rovte; ikita Zrelovs
EPI_ISL_538853, EPI_ISL_538946, EPI_ISL_538957, EPI_ISL_538973, EPI_ISL_539089	Leeds Teaching Hospitals NHS Trust and Public Health England, National Infection Service (Leeds laboratory)	Wellcome Sanger Institute for the COVID-19 Genomics UK (COG-UK) consortium	Antony Hale and Alex Alderton; Cordelia Langford; David K. Jackson; Dominic Kwiatkowski; Ewan Harrison; Holli Carden; Ian Johnston; John Sillitoe on behalf of the Wellcome Sanger Institute COVID-19 Surveillance Team; Katherine L. Harper; Louissa Macfarlane-Smith; Roberto Amato; Sonia Goncalves
EPI_ISL_552935, EPI_ISL_553292, EPI_ISL_553452, EPI_ISL_554030, EPI_ISL_554087, EPI_ISL_554093, EPI_ISL_555415, EPI_ISL_555541, EPI_ISL_555721, EPI_ISL_555857, EPI_ISL_555872, EPI_ISL_555889, EPI_ISL_556323, EPI_ISL_557067, EPI_ISL_557083, EPI_ISL_557155, EPI_ISL_557733, EPI_ISL_558660, EPI_ISL_558902, EPI_ISL_558948, EPI_ISL_559219, EPI_ISL_559480, EPI_ISL_589410, EPI_ISL_589470			
see above	Lighthouse Lab in Alderley Park	Wellcome Sanger Institute for the COVID-19 Genomics UK (COG-UK) consortium	Cordelia Langford; David K. Jackson; Dominic Kwiatkowski; Ewan Harrison; Ian Johnston; Jacquelyn Wynn; John Sillitoe on behalf of the Wellcome Sanger Institute COVID-19 Surveillance Team; John Sillitoe on behalf of the Wellcome Sanger Institute COVID-19 Surveillance Team (http://www.sanger.ac.uk/covid-team); Mairead Hyland; Roberto Amato; Sonia Goncalves; The Lighthouse Lab in Alderley Park and Alex Alderton
EPI_ISL_533375, EPI_ISL_537084	Lighthouse Lab in Glasgow	Wellcome Sanger Institute for the COVID-19 Genomics UK (COG-UK) consortium	Anna Dominiczak and Alex Alderton; Carol Clugston; Cordelia Langford; David Gray; David K. Jackson; Dominic Kwiatkowski; Ewan Harrison; Harper VanSteenhouse; Ian Johnston; John Sillitoe; John Sillitoe on behalf of the Wellcome Sanger Institute COVID-19 Surveillance Team; Roberto Amato; Sonia Goncalves; Yumi Kasai
EPI_ISL_820790, EPI_ISL_820800, EPI_ISL_820924	Lighthouse Lab in Milton Keynes	Wellcome Sanger Institute for the COVID-19 Genomics UK (COG-UK) Consortium	Cordelia Langford; David K. Jackson; Dominic Kwiatkowski; Ewan Harrison; Ian Johnston; John Sillitoe on behalf of the Wellcome Sanger Institute COVID-19 Surveillance Team; Roberto Amato; Sonia Goncalves; The Lighthouse Lab in Milton Keynes and Alex Alderton
EPI_ISL_499677, EPI_ISL_517177, EPI_ISL_517232, EPI_ISL_517252, EPI_ISL_517269, EPI_ISL_517312, EPI_ISL_517350, EPI_ISL_517352, EPI_ISL_517361, EPI_ISL_517500, EPI_ISL_534752			
see above	Liverpool Clinical Laboratories	COVID-19 Genomics UK (COG-UK) Consortium	A Alrezaihi; Alessandro Gerada; Alistair Darby; Angela Cowell; Anita Lucaci; Anu Chawla; Cassie Olateju; Catherine Hartley; Charlotte Nelson; Ecaterina Vamos; Elaine O'Toole; Eleanor G Bentley; Ghada T Shawli; Isabel García-Dorival; James Johnson; James P Stewart; Jenifer Manson; Joanne Watts; Jones Benjamin; Jordan J Clark; Julian Hiscox; L Luu; Lucille Rainbow; M Almsaud; Margaret Hughes; Mark Whitehead; Matthew Gemmell; Miren Iturriza-Gomara; Muhannad Alruwaili; N.P Randle; Neil Swainston; PKF Gilmore; Parul Sharma; Rebekah Penrice-Randal; Richard Eccles; Richard Gregory; Sam Haldenby; Steve Paterson; Stuart D Armstrong; Trevor Ian Robinson; Ximeng Han
EPI_ISL_710101	Los Angeles County PHL	Los Angeles County PHL	P. Hemarajata et al.
EPI_ISL_707912	Los Angeles County Public Health Laboratory	Los Angeles County Public Health Laboratory	P. Hemarajata et al.
EPI_ISL_445311, EPI_ISL_445328	MEGASALUD S.A.	Instituto de Salud Publica de Chile	Alejandra Acevedo; Andrés E Castillo; Bárbara Parra; Carolina Tambley; Gabriel Leal; Jaime Lagos; Jorge Fernandez; Loredana Arata; Patricia Bustos; Paz Tapia; Rodrigo Fasce; Winston Andrade
EPI_ISL_445263, EPI_ISL_445358	MEGASALUD SPA.	Instituto de Salud Publica de Chile	Alejandra Acevedo; Andrés E Castillo; Bárbara Parra; Carolina Tambley; Gabriel Leal; Jaime Lagos; Jorge Fernandez; Loredana Arata; Patricia Bustos; Paz Tapia; Rodrigo Fasce; Winston Andrade
EPI_ISL_527683, EPI_ISL_527694, EPI_ISL_527706	MN PHL Division, Minnesota Department of Health	Pathogen Discovery, Respiratory Viruses Branch, Division of Viral Diseases, Centers for Disease Control and Prevention	Anna Montmayeur; Anna Uehara; Clinton R. Paden; Haibin Wang; Jing Zhang; Krista Queen; Rachel Marine; Suxiang Tong; Yan Li; Ying Tao
EPI_ISL_421383, EPI_ISL_422523, EPI_ISL_422550, EPI_ISL_450148, EPI_ISL_801983, EPI_ISL_801992, EPI_ISL_802081, EPI_ISL_802116			
see above	MSHS Clinical Microbiology Laboratories	MSHS Pathogen Surveillance Program	Adolfo Garcia-Sarstre; Adolfo Garcia-Sastre; Adolfo García-Sastre; Adriana van de Guchte; Ajay Obla; Alberto Paniz-Mondolfi; Alberto Paniz-mondolfi; Ana S. Gonzalez-Reiche; Andrew Kasarskis; Angela Amoako; Ashley S. Salimbangon; Betsaida Salom Melo; Bremy Albuquerque; Bremy Alburquerque; Brianne Ciferri; Charles Gleason; Deena R. Altman; Denise Jurczyszak; Elena Hirsch; Emilia Mia Sordillo; Emilia Sordillo; Emily Ferreri; Florian Krammer; Gintaras Deikus; Giulio Kleiner; Gopi Patel; Hala Alshammary; Harm van Bakel; Irina Oussenko; Jayeeta Dutta; Jose Polanco; Juan Soto; Judith Aberg; Katherine Beach; Kathryn Twyman; Kayla Russo; Komal Srivastava; Levy Sominsky; Lisa Miorin; Mahmoud Awawda; Marta Luksza; Matthew Hernandez; Matthew M. Hernandez; Melissa Gitman; Melissa Smith; Michael D. Nowak; Mitchell J. Sullivan; Mitchell Sullivan; Nancy Francoeur; Rachel Chernet; Randy Albrecht; Robert Sebra; Sarah Schaefer; Shelcie Fabre; Shwetha Hara Sridhar; Shwetha Sridhar Hara; Viviana Simon; Wen-chun Liu; Ying-Chih Wang; Zenab Khan

EPI_ISL_445309, EPI_ISL_445355	MUTUAL DE SEGURIDAD C.CH.C.	Instituto de Salud Publica de Chile	Alejandra Acevedo; Andrés E Castillo; Bárbara Parra; Carolina Tambley; Gabriel Leal; Jaime Lagos; Jorge Fernandez; Loredana Arata; Patricia Bustos; Paz Tapia; Rodrigo Fasce; Winston Andrade
EPI_ISL_513452, EPI_ISL_513480	Maine HETL	Tewhey Lab, The Jackson Laboratory	Barter, M.; Dewey, H.; H. and Tewhey, R.; Lynch, R.; Matluk, N.; Munger
EPI_ISL_481270, EPI_ISL_509471	Maryland Department of Health	Maryland Department of Health	Keller, E.
EPI_ISL_460097, EPI_ISL_460109, EPI_ISL_460110, EPI_ISL_460343, EPI_ISL_460396, EPI_ISL_460426, EPI_ISL_460451, EPI_ISL_765843, EPI_ISL_791772, EPI_ISL_791792, EPI_ISL_791872, EPI_ISL_791875, EPI_ISL_791889, EPI_ISL_791906, EPI_ISL_791946			
see above	Massachusetts General Hospital	Infectious Disease Program, Broad Institute of Harvard and MIT	A.E.; Adams, G.; Anahtar, M.; B.L.; B.W.; Bauer, M.; Birren; Branda, J.; Carter, A.; Cerrato, F.; Chaluvadi, S.; Chapman; Cusick, C.; D.J.; DeRuff, K.; E. and Sabeti; Flowers, K.; Gallagher, G.; Gladden-Young, A.; Gnirke, A.; Harris, J.; J.E.; K.J.; LaRocque, R.; Lagerborg, K.; Lemieux; Lin; Loreth, C.; MacInnis; Neumann, A.; Normandin, E.; P.C.; Park; Pierce, V.; Reilly, S.; Rosenberg; Rosenberg, E.; Rudy, M.; Ryan, E.; S.B.; Sabeti; Shaw, B.; Siddle; Slater, D.; Smole, S.; Tomkins-Tinch, C.; Turbett, S.
EPI_ISL_791515, EPI_ISL_791526, EPI_ISL_791532, EPI_ISL_791563, EPI_ISL_791584, EPI_ISL_791633, EPI_ISL_791640, EPI_ISL_791656, EPI_ISL_791659, EPI_ISL_791700			
see above	Massachusetts State Public Health Laboratory	Infectious Disease Program, Broad Institute of Harvard and MIT	A.E.; Adams, G.; Anahtar, M.; B.L.; B.W.; Bauer, M.; Birren; Branda, J.; Carter, A.; Cerrato, F.; Chaluvadi, S.; Chapman; Cusick, C.; D.J.; DeRuff, K.; E. and Sabeti; Flowers, K.; Gallagher, G.; Gladden-Young, A.; Gnirke, A.; Harris, J.; J.E.; K.J.; LaRocque, R.; Lagerborg, K.; Lemieux; Lin; Loreth, C.; MacInnis; Neumann, A.; Normandin, E.; P.C.; Park; Pierce, V.; Reilly, S.; Rosenberg; Rudy, M.; Ryan, E.; S.B.; Shaw, B.; Siddle; Slater, D.; Smole, S.; Tomkins-Tinch, C.; Turbett, S.
EPI_ISL_495599, EPI_ISL_495607	Mayo Clinic & Mayo Clinic Laboratories	Minnesota Department of Health, Public Health Laboratory	Jacob Garfin; Matt Plumb; and Xiong Wang
EPI_ISL_591522, EPI_ISL_591523, EPI_ISL_591524, EPI_ISL_591525, EPI_ISL_591526, EPI_ISL_591527, EPI_ISL_591528, EPI_ISL_591529, EPI_ISL_591530			
see above	Medicina Norte U Chile - Servicio Medico Legal	Center for Mathematical Modeling and Center for Genome Regulation. Santiago, Chile	Allende ML; Ferres M.; Gaete A; Gaggero A; González M; Maass A; Palma R; Travisany D; Urra C; Valiente F; Varas M
EPI_ISL_447091, EPI_ISL_452326, EPI_ISL_507700, EPI_ISL_516270, EPI_ISL_516277, EPI_ISL_516281, EPI_ISL_516282, EPI_ISL_516331, EPI_ISL_516360, EPI_ISL_565961, EPI_ISL_565987			
see above	Michigan Department of Health and Human Services, Bureau of Laboratories	Michigan Department of Health and Human Services, Bureau of Laboratories	Blankenship HM; Riner D; Soehnlen MK
EPI_ISL_426583	Microbial Genomics Laboratory, Institut Pasteur Monteivdeo	Microbial Genomics Laboratory, Institut Pasteur Montevideo, Uruguay	Cecilia Salazar; Florencia Díaz-Viraqué; Gonzalo Moratorio; Gregorio Iraola; Marianoel Pereira; Pilar Moreno
EPI_ISL_426479, EPI_ISL_426480, EPI_ISL_429257	Microbial Genomics Laboratory, Institut Pasteur Montevideo	Microbial Genomics Laboratory, Institut Pasteur Montevideo	Cecilia Salazar; Florencia Díaz-Viraqué; Gonzalo Moratorio; Gregorio Iraola; Marianoel Pereira; Pilar Moreno
EPI_ISL_426476, EPI_ISL_426477, EPI_ISL_426478	Microbial Genomics Laboratory, Institut Pasteur Montevideo	Microbial Genomics Laboratory, Institut Pasteur Montevideo, Uruguay	Cecilia Salazar; Florencia Díaz-Viraqué; Gonzalo Moratorio; Gregorio Iraola; Marianoel Pereira; Pilar Moreno
EPI_ISL_426481, EPI_ISL_426482	Microbial Genomics Laboratory, Institut Pasteur Montevideo, Uruguay	Microbial Genomics Laboratory, Institut Pasteur Montevideo	Cecilia Salazar; Florencia Díaz-Viraqué; Gonzalo Moratorio; Gregorio Iraola; Marianoel Pereira; Pilar Moreno
EPI_ISL_426584	Microbial Genomics Laboratory, Institut Pasteur Montevideo, Uruguay	Microbial Genomics Laboratory, Institut Pasteur Montevideo, Uruguay	Cecilia Salazar; Florencia Díaz-Viraqué; Gonzalo Moratorio; Gregorio Iraola; Marianoel Pereira; Pilar Moreno
EPI_ISL_480331, EPI_ISL_480332, EPI_ISL_480333, EPI_ISL_480334, EPI_ISL_480335, EPI_ISL_480336, EPI_ISL_480337, EPI_ISL_480338, EPI_ISL_480339, EPI_ISL_480340, EPI_ISL_480341, EPI_ISL_480342, EPI_ISL_480343, EPI_ISL_480344, EPI_ISL_480346, EPI_ISL_480347			
see above	Microbial Genomics Laboratory, Institut Pasteur de Montevideo	Microbial Genomics Laboratory, Institut Pasteur de Montevideo	Cecilia Salazar; Gonzalo Moratorio; Gregorio Iraola; Ignacio Ferrés; Marianoel Pereira; Pilar Moreno
EPI_ISL_419728	Microbiological Diagnostic Unit Public Health Laboratory	Microbiological Diagnostic Unit Public Health Laboratory	Sait, M.; Schultz M.; Seemann T.; Sherry, N.
EPI_ISL_482106, EPI_ISL_482109	Microbiology Department, Hereford County Hospital	Wellcome Sanger Institute for the COVID-19 Genomics UK (COG-UK) consortium	Alison Johnson; Cordelia Langford; David K. Jackson; Dominic Kwiatkowski; Ewan Harrison; Fenella Halstead; Ian Johnston; Jane Thomas; John Sillitoe on behalf of the Wellcome Sanger Institute COVID-19 Surveillance Team (http://www.sanger.ac.uk/covid-team); Roberto Amato; Samantha Lamb and Alex Alderton; Sonia Goncalves; Venkat Sivaprakasam; Wendy Hogsden
EPI_ISL_486663	Microbiology, Virology and Biemergency Laboratory-ASST FBF Sacco	Microbiology, Virology and Biemergency Laboratory-ASST FBF Sacco	Comandatore F; Mancon A; Micheli V; Rimoldi SG; Romeri F
EPI_ISL_548124, EPI_ISL_579301	Middlemore Hospital	Institute of Environmental Science and Research (ESR)	Anja Werno; Antje van der Linden; Arlo Upton; Chris Mansell; David Hammer; Dragana Drinkovic; Erasmus Smit; Gary McAuliffe; Hana Sofia Andersson; Hermes Perez; James Ussher; Jill Sherwood; Jing Wang; Joep de Ligt; Josh Freeman; Julia Howard; Juliet Elvy; Lauren Jelly; Mary DeAlmeida; Matt Blakiston; Matt Storey; Matthew Rogers; Max Bloomfield; Michael Addidle; Michelle Balm; Muhammad Faisal; Nikki Freed; Olin Silander; Sally Roberts; Sarah Jefferies; Sharmini Muttaiyah; Susan Morpeth; Susan Taylor; Timothy Blackmore; Vani Sathyendran; Veronica Playle; Virginia Hope; Xiaoyun Ren
EPI_ISL_735413	Militello Centro de Diagnosticos e Biopesquisa Clinica	Instituto Adolfo Lutz, Interdiciplinary Procedures Center, Strategic Laboratory	Claudia Regina Gonçalves; Claudio Tavares Sacchi; Erica Valessa Ramos Gomes; Karoline Rodrigues Campos
EPI_ISL_718145, EPI_ISL_718153, EPI_ISL_718156, EPI_ISL_718157	Ministry of Health Hospitals	Institute of Health and Community Medicine	Chan Chia Jui; Chua Hock Hin; David Perera; Ooi Mong How; Tonnii Sia Loong Loong; Wong Jyn Shan; Wong Kiing Aik
EPI_ISL_437368, EPI_ISL_437386, EPI_ISL_450753, EPI_ISL_450761, EPI_ISL_495615, EPI_ISL_495627, EPI_ISL_530196, EPI_ISL_590768, EPI_ISL_590813			
see above	Minnesota Department of Health, Public Health Laboratory	Minnesota Department of Health, Public Health Laboratory	Alexandra Lorentz; Jacob Garfin; Matt Plumb; and Xiong Wang
EPI_ISL_435130, EPI_ISL_520666, EPI_ISL_520685, EPI_ISL_520738	Mohammed Bin Rashid University of Medicine and Health Sciences	Al Jalila Genomics Center	Abdulmajeed Alkhaja; Abiola Catherine Senok; Ahmad Abou Tayoun; Alawi Alsheikh-Ali; Divinlal Harilal; Hamda Khansaheb; Hanan Al Suwaidi; Mohammed Uddin; Norbert Nowotny; Qutayba Hamid; Rabih Halwani; Rifat Hamoudi; Rupa Murthy Varghese; Sathishkumar Ramaswamy; Tom Loney; Zulfa Omar Deesi
EPI_ISL_467475, EPI_ISL_467497	Molecular Diagnostics Services (MDS)	KRISP, KZN Research Innovation and Sequencing Platform	Chimukangara B; Giandhari J; Khan S; Lessells R; Mdlalose K; Pillay S; Tegally H; Wilkinson E; York D; de Oliveira T
EPI_ISL_681673, EPI_ISL_681674, EPI_ISL_681675, EPI_ISL_681676, EPI_ISL_681677, EPI_ISL_681678, EPI_ISL_681679, EPI_ISL_681680, EPI_ISL_681681, EPI_ISL_681682, EPI_ISL_681683, EPI_ISL_681684, EPI_ISL_681685, EPI_ISL_681686, EPI_ISL_681687, EPI_ISL_681688, EPI_ISL_681689, EPI_ISL_681690, EPI_ISL_681691, EPI_ISL_681692, EPI_ISL_681693, EPI_ISL_681694, EPI_ISL_681695, EPI_ISL_681696, EPI_ISL_681697, EPI_ISL_681698, EPI_ISL_681699, EPI_ISL_681700, EPI_ISL_681701			
see above	Molecular Medicine Laboratory, University of Magallanes	Centro Asistencial Docente y de Investigacion, Universidad de Magallanes	Diego Alvarez; Hermy Alvarez; Jacqueline Aldridge; Jorge González; Marcelo Navarrete; Marco Montes de Oca; Roberto Uribe-Paredes
EPI_ISL_802545, EPI_ISL_802546, EPI_ISL_802547, EPI_ISL_802548, EPI_ISL_802550, EPI_ISL_802551, EPI_ISL_802552, EPI_ISL_802553, EPI_ISL_802554, EPI_ISL_802555, EPI_ISL_802556, EPI_ISL_802558, EPI_ISL_802559, EPI_ISL_802560, EPI_ISL_802561, EPI_ISL_802562, EPI_ISL_802563, EPI_ISL_802564, EPI_ISL_802565			
see above	Molecular Microbiology and Food Research Laboratory (MMFRLAB) - Universidad San Sebastián	Facultad de Ciencias de la Vida, UNAB	Claudio Meneses; César Echeverría; Dayán Sanhueza; Eduardo Castro; Jorge Olivares; Macarena Bastías; Sebastián Wolter; Waldo Díaz
EPI_ISL_510535	Molecular Virology, Instituto Carlos Chagas / Fiocruz Paraná	Universidade Federal do Parana (UFPR)	Duarte dos Santos, C.; Raboni, S.; Suzukawa, A.; Tscha, M.; Zanluca, C.
EPI_ISL_486826	Molecular diagnostic laboratory of Federal Budget Institution of Science "Central Research Institute of Epidemiology" of The Federal Service on Customers' Rights Protection and Human Well-being Surveillance	Group of Genomics and Postgenomic Technologies of Central Research Institute of Epidemiology	Akimkin VG; Bulanenko VP; Kaptelova VV; Korneenko EV; Samoilov AE; Shipulina OY; Speranskaya AS; Tivanova EV; Valdokhina AV
EPI_ISL_631880	Mount Sinai West	New York City Public Health Laboratory	Jade Wang; et al.
EPI_ISL_510812	NA	The Public Health Agency of Sweden	Anna Risberg; Anna-Malin Linde; Karin Tegmark-Wisell; Maria Lind Karlberg; Mattias Haukland; Mia Brytting; Olov Svartstrom; Oskar Karlsson Lindsjo; Petra Edquist; Reza Advani; Sandra Broddesson

EPI_ISL_452126	NC State Laboratory of Public Health	Pathogen Discovery, Respiratory Viruses Branch, Division of Viral Diseases, Centers for Disease Control and Prevention	Alison S. Laufer Halpin; Anna Montmayeur; Anna Uehara; Christopher A. Elkins; Clinton R. Paden; Haibin Wang; Jing Zhang; Krista Queen; Mary S. Keckler; Rachel Marine; Suxiang Tong; Yan Li; Ying Tao; Zachary Weiner
EPI_ISL_509274, EPI_ISL_509344, EPI_ISL_529751	NHLS-IALCH	KRISP, KZN Research Innovation and Sequencing Platform	Giandhari J; Khan S; Lessells R; Mdlalose K; Pillay S; Tegally H; Wilkinson E; York D; de Oliveira T
EPI_ISL_489594	NHSGGC West of Scotland Specialist Virology Centre / MRC-University of Glasgow Centre for Virus Research	Wellcome Sanger Institute for the COVID-19 Genomics UK (COG-UK) consortium	Alasdair MacLean; Alice Broos; Ana da Silva Filipe; Antonia Ho; Cordelia Langford; Daniel Mair; David K. Jackson; David L Robertson; Dominic Kwiatkowski; Elihu Aranday-Cortes; Emma Thomson and Alex Alderton; Ewan Harrison; Ian Johnston; James Shepherd; Jenna Nichols; John Sillitoe on behalf of the Wellcome Sanger Institute COVID-19 Surveillance Team (http://www.sanger.ac.uk/covid-team); Joseph Hughes; Kathy Li; Kathy Smollett; Kirstyn Brunker; Kyriaki Nomikou; Lily Tong; Marc Niebel; Natasha Jesudason; Natasha Johnson; Patawee Asamaphan; Rajiv Shah; Richard Orton; Roberto Amato; Rory Gunson; Sarah McDonald; Sonia Goncalves; Sreenu Vattipally; Stephen Carmichael; Yasmin Parr
EPI_ISL_488003, EPI_ISL_488076, EPI_ISL_488639, EPI_ISL_488654, EPI_ISL_488798, EPI_ISL_488821	NU-OMICS DNA Sequencing research facility, Northumbria University	Wellcome Sanger Institute for the COVID-19 Genomics UK (COG-UK) consortium	Andrew Nelson; Brendan Payne; Chris Duncan; Clive Graham; Cordelia Langford; Darren Smith and Alex Alderton; David K. Jackson; Debra Padgett; Dominic Kwiatkowski; Edward Barton; Emma Swindells; Ewan Harrison; Garren Scott; Gary Black; Gary Eltringham; Greg Young; Ian Johnston; Jane Greenaway; Jennifer Collins; John Allan; John Sillitoe on behalf of the Wellcome Sanger Institute COVID-19 Surveillance Team (http://www.sanger.ac.uk/covid-team); Joshua Loh; Lynn Dover; Matthew Bashton; Paul Baker; Roberto Amato; Sarah Essex; Sheia Waugh; Shirelle Burton-Fanning; Sonia Goncalves; Steve Ligget; Wen Yew; Yusri Taha
EPI_ISL_632062	NYC Department Of Health And Mental Hygiene	New York City Public Health Laboratory	Jade Wang; et al.
EPI_ISL_436059, EPI_ISL_480784	NYC Department of Health and Mental Hygiene	Pathogen Discovery, Respiratory Viruses Branch, Division of Viral Diseases, Centers for Disease Control and Prevention	Anna Uehara; Bettina Bankamp; Christine Mahl; Christy Harrison; Clinton R. Paden; Haibin Wang; Jasmine Padilla; Jennifer Rakeman; Jing Zhang; Justin Lee; Krista Queen; Sally Slavinski; Suxiang Tong; Yan Li; Ying Tao; Zachary Weiner
EPI_ISL_420308, EPI_ISL_420309, EPI_ISL_420570, EPI_ISL_420587, EPI_ISL_421587, EPI_ISL_421724, EPI_ISL_424950, EPI_ISL_424968, EPI_ISL_426619, EPI_ISL_426621, EPI_ISL_426622, EPI_ISL_426625, EPI_ISL_427488, EPI_ISL_427631, EPI_ISL_428762, EPI_ISL_430334, EPI_ISL_430367, EPI_ISL_430381, EPI_ISL_430417, EPI_ISL_435479, EPI_ISL_435480, EPI_ISL_435486, EPI_ISL_435495, EPI_ISL_435515, EPI_ISL_444624, EPI_ISL_444633, EPI_ISL_444644, EPI_ISL_444656, EPI_ISL_444662, EPI_ISL_444686, EPI_ISL_444701, EPI_ISL_444705, EPI_ISL_444724, EPI_ISL_444727, EPI_ISL_444765, EPI_ISL_450393, EPI_ISL_450397, EPI_ISL_451413, EPI_ISL_451442, EPI_ISL_451470, EPI_ISL_456013, EPI_ISL_456047, EPI_ISL_456056, EPI_ISL_458020			
see above	NYU Langone Health	Departments of Pathology and Medicine, New York University School of Medicine	Adriana Heguy; Alireza Khodadadi-Jamayran; Amy Rapkiewicz; Andre M. Ribeiro-dos-Santos; Andrew Lytle; Antonio Serrano; Brendan Belovarac; Christian Marier; Dacia Dimartino; Emily Guzman; Emily Huang; Gael Westby; George Jour; Guomiao Shen; Iman Osman; Jared Pinnell; John Cadley; John Chen; Lawrence H. Lin; Ludovic Boytard; Margaret Black; Maria Aguero-Rosenfeld; Marie Samanovic-Golden; Mark J. Mulligan; Matija Snuderl; Matthew T. Maurano; Megan Hogan; Nick Vulpescu; Paolo Cotzia; Paul Zappile; Peter Meyn; Raquel Ordonez Ciriza; Raven Luther; Sitharam Ramaswami; Tatyana Gindin; Theodore Vougiouklakis; Vanessa Raabe; Xiaojun Feng; Yutong Zhang
EPI_ISL_632076	NYU Langone Health	New York City Public Health Laboratory	Jade Wang; et al.
EPI_ISL_452230	Narhalsan Backa vardcentral	The Public Health Agency of Sweden	Anna Risberg; Anna-Malin Linde; Karin Tegmark-Wisell; Maria Lind Karlberg; Mats Olsson; Mia Brytting; Olov Svartstrom; Oskar Karlsson Lindsjo; Theresa Enkirch
EPI_ISL_420108	National Centre for Infectious Diseases	Programme in Emerging Infectious Diseases, Duke-NUS Medical School	Barnaby E Young; Danielle E Anderson; David CB Lye; Gavin JD Smith; Jayanthi Jayakumar; Martin Linster; Yan Zhuang; Yee Sin Leo; Yvonne CF Su
EPI_ISL_464131, EPI_ISL_464157	National Health Laboratory Service (NHLS), Tygerberg	Division of Medical Virology, Stellenbosch University and National Health Laboratory Service (NHLS)	Bronwyn Kleinhans; Eduan Wilkindon; Gert van Zyl; Houriiyah Tegally; Kayla Delaney; Susan Engelbrecht; Tulio de Oliveira; Wolfgang Preiser
EPI_ISL_455702, EPI_ISL_511891	National Hospital of Tropical Diseases	Oxford University Clinical Research Unit, Hanoi, Vietnam	H. Rogier van Doorn; Le Nguyen Minh Hoa; Nguyen Thi Hong Thuong; Nguyen Thi Ngoc Diep; Nguyen Thi Tam; Nguyen Thu Trang; Pham Ngoc Thach; Van Dinh Trang; Vu Thi Ngoc Bich; on behalf of the OUCRU COVID-19 research group
EPI_ISL_416028, EPI_ISL_416031, EPI_ISL_416032, EPI_ISL_416035, EPI_ISL_416036, EPI_ISL_515525	National Influenza Center - Instituto Adolfo Lutz	Instituto Adolfo Lutz, Interdiciplinary Procedures Center, Strategic Laboratory	Adriana Bugno; Adriano Abbud; Carlos Henrique Camargo; Claudia Regina Gonçalves; Claudio Tavares Sacchi; Daniela Bernardes Borges da Silva; Erica Valessa Ramos Gomes; Fabiana Cristina Pereira dos Santos; Maria do Carmo Sampaio Tavares Timenetsky; Simone Guadagnucci Morillo; Terezinha Maria de Paiva
EPI_ISL_416430	National Influenza Center, National Institute of Hygiene and Epidemiology (NIHE)	National Influenza Center, National Institute of Hygiene and Epidemiology (NIHE)	Dang Duc Anh; Futoshi Hasebe; Hoang Vu Mai Phuong; Kouichi Morita; Le Quynh Mai; Le Thi Thanh; Meng Ling Moi; Nguyen Le Khanh Hang; Nguyen Phuong Anh; Nguyen Vu Son; Pham Hong Quynh Anh; Pham Thi Hien; Taichiro Takemura; Takeshi Nabeshima; Tran Thu Huong; Ung Thi Hong Trang; Vuong Duc Cuong
EPI_ISL_417186	National Institute for Communicable Diseases of the National Health Laboratory Service	National Institute for Communicable Diseases of the National Health Laboratory Service	A; Allam M; Bhiman JN; Ismail A; Khumalo Z; Kwenda S; Mohale T; Subramoney K; van Heusden P; von Gottberg
EPI_ISL_483636, EPI_ISL_498227	National Institute of Laboratory Medicine and Referral Center	Genomic Research Lab, BCSIR	A. K. M. Shamsuzzaman; Abu Sayeed Mohammad Mahmud; Asish Kumar Ghosh; Barna Goswami; Eshrar Osman; Iffat Jahan; Mahmuda Yeasmin; Md. Ahasan Habib; Md. Maruf Ahmed Molla; Md. Murshed Hasan Sarkar; Md. Saddam Hossain; Md. Salim Khan; Mohammad Samir Uzzaman; Salek Ahmed Sajib; Shahina Akter; Sheikh Md. Selim Al Din; Tanjina Akhter Banu; Tasnim Nafisa; Utpal Chandra Ray
EPI_ISL_443218, EPI_ISL_462295, EPI_ISL_479596, EPI_ISL_548985, EPI_ISL_574496	National Public Health Laboratory, National Centre for Infectious Diseases	National Public Health Laboratory, National Centre for Infectious Diseases	Chavatte JM; Chavatte Jean-Marc; Cui L; Cui Lin; Lin Cui; Lin RTP; Lin Raymond Tzer Pin; Mak TM; Mak Tze Minn; Octavia S; Octavia Sophie; Raymond Tzer Pin Lin; Sophie Octavia; Tze Minn Mak; Zhenyang Zhou; Zhou Z
EPI_ISL_681919, EPI_ISL_767736	National Virus Reference Laboratory	Irish Coronavirus Sequencing Consortium - Teagasc Moorepark	Calum Walsh; Fiona Crispie; Genuity Ireland; John Kenny; Matthew McCabe; Paul Cotter
EPI_ISL_444999	Naval Health Research Center	Naval Medical Research Center Biological Defense Research Directorate	Adrian Paskey; Chris Myers; Dessiree Pena-Gomez; Ewell Hollis; Kimberly Bishop-Lilly; Kyle Long; Logan Voegtly; Melinda Balansay-Ames; Nathaniel Christy; Regina Cer; Roger Pan
EPI_ISL_491923	Naval Infectious Diseases Diagnostic Laboratory	Naval Medical Research Center Biological Defense Research Directorate	Adrian Paskey; Dessiree Pena-Gomez; Francisco Malgon Bautista; Hua Wei Chen; Kimberly Bishop-Lilly; Kyle Long; Lindsay Glang; Logan Voegtly; Mark Simons; Megan Schilling; Regina Cer; Victor Sugiharto
EPI_ISL_515327, EPI_ISL_515332, EPI_ISL_515372, EPI_ISL_515373, EPI_ISL_515454	Nevada State Public Health Laboratory	Nevada State Public Health Laboratory	Andrew Gorzalski; Chris Laverdure; Cyprian Rossetto; David Jackson; Heather Kerwin; Joel R. Sevinsky; Natalie Crawford; Paul Hartley; Richard Tillett; Stephanie Van Hooser; Subhash C. Verma; and Mark Pandori
EPI_ISL_631861, EPI_ISL_632049	New York Presbyterian Queens	New York City Public Health Laboratory	Jade Wang; et al.
EPI_ISL_427531, EPI_ISL_427537, EPI_ISL_427542, EPI_ISL_427568, EPI_ISL_427589, EPI_ISL_427614, EPI_ISL_427617			
see above	NewYork-Presbyterian & Mason Lab	Mason Lab	Alon Shaiber; Arryn Craney; Benjamin Young; Cem Meydan; Chandrima Bhattacharya; Christopher E. Mason; Christopher Mozsary; Craig D. Westover; Daniel J. Butler; David Danko; Dmitry Meleshko; Dong Xu; Ebrahim Afshinnekoo; Fritz J. Sedlazeck; Hanna Rennert; Iman Hajirasouliha; Jenny Xiang; Joel Rosiene; John Sipley; Jonathan Foox; Justyna Gawrys; Krista Ryon; Lars F. Westblade; Lin Cong; Marcin Imielinski; Maria Sierra; Massimo Loda; Matthew MacKay; Melissa Cushing; Mirella Salvatore; Nikolay A. Ivanov; Phyllis Ruggiero; Priya Velu; Shawn Levy
EPI_ISL_516831	North West London Pathology, Imperial College Healthcare NHS Trust	Wellcome Sanger Institute for the COVID-19 Genomics UK (COG-UK) consortium	Aileen Rowan; Alison Holmes; Anjna Badhan; Carolina Herrera and Alex Alderton; Cordelia Langford; David K. Jackson; David Muir; Dominic Kwiatkowski; Ewan Harrison; Frankie Bolt; Graham Taylor; Ian Johnston; James Price; John Sillitoe on behalf of the Wellcome Sanger Institute COVID-19 Surveillance Team (http://www.sanger.ac.uk/covid-team); Ling Li; Paul Randell; Roberto Amato; Sonia Goncalves
EPI_ISL_444596	Northwestern Memorial Hospital	Ozer Lab	Alan R. Hauser; Chad J. Achenbach; Chao Qi; Egon A. Ozer; Hannah H. Nam; Judd F. Hultquist; Lacy M. Simons; Lawrence J. Jennings; Michael G. Ison; Ramon Lorenzo-Redondo; Scott C. Roberts
EPI_ISL_534326	Notre Dame Intermedica Saude AS	Instituto Adolfo Lutz, Interdiciplinary Procedures Center, Strategic Laboratory	Claudia Regina Gonçalves; Claudio Tavares Sacchi; Erica Valessa Ramos Gomes
EPI_ISL_700486, EPI_ISL_700511	Nyanga CDC wc NGC	NHLS/UCT	Arash Iranzadeh; Bruna Galvao; Carolyn Williamson; Deelan Doolabh; Diana Hardie; Innocent Mudau; Kruger Marais; Lynn Tyers; Marvin Hsiao; Stephen Korsman
EPI_ISL_512846	O.I.J. MORGUE JUDICIAL	Inciensa, Instituto Costarricense de Investigación y	Adriana Godinez & Melany Calderon; Claudio Soto-Garita; Estela Cordero; Francisco Duarte; Hebleen Porras

		Enseñanza en Nutrición y Salud	
EPI_ISL_631937	OCME Office Of Chief Medical Examiner	New York City Public Health Laboratory	Jade Wang; et al.
EPI_ISL_534316	OS Mun Santana Lauro Ribas Braga	Instituto Adolfo Lutz, Interdiciplinary Procedures Center, Strategic Laboratory	Claudia Regina Gonçalves; Claudio Tavares Sacchi; Erica Valessa Ramos Gomes
EPI_ISL_770776	Ohio Department of Health Laboratory	Ohio Department of Health Laboratory	Caitiln McDonnell; Eric Brandt; Erica Leasure; Glen McGillivary; Heather Blankenship; Holmes; Jennifer; Kelsey Florek; Keoni Omura; Kirtana Ramadugu; Quanta Brown; and Tammy Bannerman
EPI_ISL_491136, EPI_ISL_491162	Oman-National Influenza Center	Biotechnology & OMICs Laboratory	Abdul Latif Khan; Adil Al-Wahaibi; Adil Khan; Ahlam Al-Amri; Ahmed Al-Harrasi; Ahmed Al-Rawahi; Aisha Al-Amri; Aisha Al-Busaidi; Amina Al-Jardani; Hanan Al-Kindi; Intisar Al-Shukri; Sajjad Asaf; Samiha Al-Kharusi; Samira Al-Mahruqi; Seif Al-Abri; Seif Al-Abri.
EPI_ISL_569767, EPI_ISL_569772, EPI_ISL_569811, EPI_ISL_569832, EPI_ISL_569841, EPI_ISL_569848, EPI_ISL_629033			
see above	Omsk Research Institute of Natural Focal Infections	WHO National Influenza Centre Russian Federation	Aleksei Vasilenko; Andrey Komissarov; Artem Fadeev; Daria Nashatyreva; Ekaterina Gradoboeva; Ekaterina Savkina; Elena Poleshchuk; Valery Yakimenko
EPI_ISL_434673, EPI_ISL_475534	Omtanken Grimmered	The Public Health Agency of Sweden	Anna Risberg; Anna-Malin Linde; Bernd Sengpiel; Karin Tegmark-Wisell; Maria Lind Karlberg; Mattias Haukland; Mia Brytting; Olov Svartstrom; Oskar Karlsson Lindsjo; Reza Advani; Sandra Broddesson; Theresa Enkirch
EPI_ISL_486148	Orange County Public Health Laboratory	Chan-Zuckerberg Biohub	CZB Cliahub Consortium
EPI_ISL_660379	Orebro klinisk mikrobiologi	The Public Health Agency of Sweden	Anna Risberg; Anna-Malin Linde; Karin Tegmark-Wisell; Maria Lind Karlberg; Mattias Haukland; Mia Brytting; Olov Svartstrom; Oskar Karlsson Lindsjo; Petra Edquist; Reza Advani; Sandra Broddesson
EPI_ISL_448451, EPI_ISL_448518, EPI_ISL_448776, EPI_ISL_478740, EPI_ISL_478775, EPI_ISL_478848, EPI_ISL_478870, EPI_ISL_478975, EPI_ISL_479022, EPI_ISL_479144, EPI_ISL_534799, EPI_ISL_534848, EPI_ISL_534898, EPI_ISL_534903, EPI_ISL_534926, EPI_ISL_534932, EPI_ISL_534933, EPI_ISL_534972, EPI_ISL_534982, EPI_ISL_576820			
see above	Oxford Viromics, NDM, University of Oxford; Oxford University Hospitals; Basingstoke and North Hampshire Hospital	COVID-19 Genomics UK (COG-UK) Consortium	Alex Mobbs; Amy Trebes; Anita Justice; Catrin Moore; Christophe Fraser; David Bonsall; David Buck; Emma Wise; George Macintyre; Jessica Lynch; John Todd; Mariateresa de Cesare; Matilde Mori; Monique Andersson; Nathan Moore; Nick Cortes; Robert Shaw; Stephen Kidd; Tanya Golubchik; Timothy Peto
EPI_ISL_492580, EPI_ISL_492624, EPI_ISL_492678, EPI_ISL_492727, EPI_ISL_492764	PHE South West Regional Laboratory, National Infection Service	Wellcome Sanger Institute for the COVID-19 Genomics UK (COG-UK) consortium	Barry Vipond; Cordelia Langford; David K. Jackson; Dominic Kwiatkowski; Dr Peter Muir; Ewan Harrison; Hannah Pymont; Ian Johnston; John Sillitoe on behalf of the Wellcome Sanger Institute COVID-19 Surveillance Team (http://www.sanger.ac.uk/covid-team); Rich Hopes; Roberto Amato; Sonia Goncalves; Stephanie Hutchings; and Alex Alderton
EPI_ISL_445252	PONTIFICIA U. CATOLICA FAC. MEDICINA	Instituto de Salud Publica de Chile	Alejandra Acevedo; Andrés E Castillo; Bárbara Parra; Carolina Tambley; Gabriel Leal; Jaime Lagos; Jorge Fernandez; Loredana Arata; Patricia Bustos; Paz Tapia; Rodrigo Fasce; Winston Andrade
EPI_ISL_445356	PONTIFICIA U. CATOLICA SERV. LABORATORIO	Instituto de Salud Publica de Chile	Alejandra Acevedo; Andrés E Castillo; Bárbara Parra; Carolina Tambley; Gabriel Leal; Jaime Lagos; Jorge Fernandez; Loredana Arata; Patricia Bustos; Paz Tapia; Rodrigo Fasce; Winston Andrade
EPI_ISL_445265	PONTIFICIA UNIVERSIDAD CATOLICA DE CHILE	Instituto de Salud Publica de Chile	Alejandra Acevedo; Andrés E Castillo; Bárbara Parra; Carolina Tambley; Gabriel Leal; Jaime Lagos; Jorge Fernandez; Loredana Arata; Patricia Bustos; Paz Tapia; Rodrigo Fasce; Winston Andrade
EPI_ISL_534322	PS Mun Julio Tupy	Instituto Adolfo Lutz, Interdiciplinary Procedures Center, Strategic Laboratory	Claudia Regina Gonçalves; Claudio Tavares Sacchi; Erica Valessa Ramos Gomes
EPI_ISL_523973	PS Municipal Dona Maria Antonieta Ferreira de Barros	Instituto Adolfo Lutz, Interdiciplinary Procedures Center, Strategic Laboratory	Claudia Regina Gonçalves; Claudio Tavares Sacchi; Erica Valessa Ramos Gomes
EPI_ISL_547570	PS Municipal Dr Augusto Gomes de Mattos	Instituto Adolfo Lutz, Interdiciplinary Procedures Center, Strategic Laboratory	Claudia Regina Gonçalves; Claudio Tavares Sacchi; Erica Valessa Ramos Gomes; Karoline Rodrigues Campos
EPI_ISL_515523, EPI_ISL_515524, EPI_ISL_515566, EPI_ISL_524466, EPI_ISL_527866	PS Municipal Dr Lauro Ribas Braga	Instituto Adolfo Lutz, Interdiciplinary Procedures Center, Strategic Laboratory	01246-1301; 355 - Brazil; Av. Dr. Arnaldo; Cerqueira Cesar; Claudia Regina Gonçalves; Claudio Tavares Sacchi; Erica Valessa Ramos Gomes; São Paulo - SP
EPI_ISL_524465	PS Municipal Dr. Caetano Virgílio Neto	Instituto Adolfo Lutz, Interdiciplinary Procedures Center, Strategic Laboratory	Claudia Regina Gonçalves; Claudio Tavares Sacchi; Erica Valessa Ramos Gomes
EPI_ISL_534321	PS e Maternidade Nair Fonseca Leitao Arantes	Instituto Adolfo Lutz, Interdiciplinary Procedures Center, Strategic Laboratory	Claudia Regina Gonçalves; Claudio Tavares Sacchi; Erica Valessa Ramos Gomes
EPI_ISL_654875	Pasteur Institute in Ho Chi Minh city	Department of Microbiology and Immunology - Pasteur Institute in Ho Chi Minh city	Cao Minh Thng; Hoàng Minh; Hoàng Nh ào; Hoàng Quc Cng; Hunh Phng Tho; Hunh Th Kim Loan; Lng Chn Quang; Nguyn Hoàng Anh; Nguyn Hoàng Quân; Nguyn Thanh Long; Nguyn Th Ngc Tho; Nguyn Th Thanh Thng; Nguyn Trung Hiu; Nguyn Vit Thinh; Nguyn V Thng; Phan Trng Lân; Phm Duy Quang; Phm Th Nhung; Phm Th Thu Hng; Trn Th Hng Kim; V Phm Hng Nhung; ào Huy Mnh; ng Thanh Giang
EPI_ISL_470863	PathWest Laboratory Medicine WA	PathWest Laboratory Medicine WA	Avram Levy; Chisha Sikazwe; David Smith and David Speers; Jurissa Lang
EPI_ISL_437489, EPI_ISL_437744, EPI_ISL_437748, EPI_ISL_512898, EPI_ISL_512955, EPI_ISL_512977, EPI_ISL_512988, EPI_ISL_512993, EPI_ISL_512996, EPI_ISL_513000, EPI_ISL_513020, EPI_ISL_513032, EPI_ISL_513045, EPI_ISL_513121, EPI_ISL_513126, EPI_ISL_513134, EPI_ISL_513164, EPI_ISL_513169, EPI_ISL_513228, EPI_ISL_513246, EPI_ISL_513258, EPI_ISL_677923, EPI_ISL_678135, EPI_ISL_678198, EPI_ISL_678208			
see above	Pathogen Genomics Lab King Abdullah University of Science and Technology(KAUST)	Pathogen Genomics Lab King Abdullah University of Science and Technology(KAUST)	Abbas Al Mutair; Abdulaziz Alahmadi; Afrah Alsomali; Ahmad Bakur Mahmoud; Amanda; Amanda Ooi; Amit Kumar Subudhi; Amit Subudhi; Anwar Hashem; Arnab Pain; Asim Khogeer; Awad Al-Omari; Fadwa Alofi; Fathia Ben Rached; Fathia Ben-Rached; Fathia Rached; Jumana Taha; Kahled Algithami; Khaled Algithami; Luke; Luke Esau; Muhammad Shuaib; Naif Almontashiri; Nashwa Al-khotani; Raeece Naeem; Rahul P Salunke; Rahul Salunke; Samer Salih; Sara Mfarrej; Sharif Hala
EPI_ISL_792386, EPI_ISL_792388, EPI_ISL_792389, EPI_ISL_792390, EPI_ISL_792392, EPI_ISL_792393, EPI_ISL_792394, EPI_ISL_792395, EPI_ISL_792396			
see above	Plataforma de Servicios Biotecnólogicos: UTTIPP/PSB , Universidad Nacional de Quilmes.	Área de Secuenciación del Laboratorio de Virología del Hospital de Niños Dr. Ricardo Gutierrez on behalf of 'Proyecto Argentino Interinstitucional de genomica de SARS-CoV-2' (PAIS Consortium)	A; Cardama; Castello; Farina; G; Goya; Goñi; H; LE; Lusso; MI; MS; Nabaes Jodar; Natale; S; Valinotto; Viegas, M.
EPI_ISL_735433	Posto de Atendimento Saude Cidade Pasc Cajati	Instituto Adolfo Lutz, Interdiciplinary Procedures Center, Strategic Laboratory	Claudia Regina Gonçalves; Claudio Tavares Sacchi; Erica Valessa Ramos Gomes; Karoline Rodrigues Campos
EPI_ISL_513513, EPI_ISL_513514, EPI_ISL_513515, EPI_ISL_513516, EPI_ISL_513517, EPI_ISL_513518, EPI_ISL_513519, EPI_ISL_513520, EPI_ISL_513521, EPI_ISL_513522, EPI_ISL_513523, EPI_ISL_513524, EPI_ISL_513525, EPI_ISL_513526, EPI_ISL_513527, EPI_ISL_513528, EPI_ISL_513529, EPI_ISL_513530, EPI_ISL_513531, EPI_ISL_513532, EPI_ISL_513533, EPI_ISL_513534, EPI_ISL_513535, EPI_ISL_513536, EPI_ISL_513537, EPI_ISL_513538, EPI_ISL_513539, EPI_ISL_513540, EPI_ISL_513541, EPI_ISL_513542, EPI_ISL_513543, EPI_ISL_513544, EPI_ISL_513545, EPI_ISL_513546, EPI_ISL_513547, EPI_ISL_513548, EPI_ISL_513549, EPI_ISL_513550, EPI_ISL_513551, EPI_ISL_513552, EPI_ISL_513553, EPI_ISL_513554, EPI_ISL_513555, EPI_ISL_513556, EPI_ISL_513557, EPI_ISL_513558, EPI_ISL_513559, EPI_ISL_513560, EPI_ISL_513561, EPI_ISL_513562, EPI_ISL_513563, EPI_ISL_513564, EPI_ISL_513565, EPI_ISL_513566, EPI_ISL_513567, EPI_ISL_513568, EPI_ISL_513569, EPI_ISL_513570, EPI_ISL_513571, EPI_ISL_513572, EPI_ISL_513573, EPI_ISL_513574, EPI_ISL_513575, EPI_ISL_513576, EPI_ISL_513577, EPI_ISL_513578, EPI_ISL_513579, EPI_ISL_513580, EPI_ISL_513581, EPI_ISL_513582, EPI_ISL_513583			
see above	Programa de Oncovirologia, Instituto Nacional de Câncer	Programa de Oncovirologia, Instituto Nacional de Câncer	Andreia C. de Melo; Brunna M. Alves; Claudia Cicala; James Arthos; João P.B. Viola; Juliana D. Siqueira; Livia R. Goes; Marcelo A. Soares
EPI_ISL_591519, EPI_ISL_591520, EPI_ISL_591521	Programa de Virología, Facultad de Medicina UC	Center for Mathematical Modeling and Center for Genome Regulation. Santiago, Chile	Allende ML; Ferres M.; Gaete A; Gaggero A; González M; Maass A; Palma R; Travisany D; Urra C; Valiente F; Varas M
EPI_ISL_527858	Pronto Atendimento Sancta Maggiore Jardim Paulista	Instituto Adolfo Lutz, Interdiciplinary Procedures Center, Strategic Laboratory	Claudia Regina Gonçalves; Claudio Tavares Sacchi; Erica Valessa Ramos Gomes
EPI_ISL_603021, EPI_ISL_693204	Pronto Socorro Dr. Conrado Cesarino Nuvolini	Instituto Adolfo Lutz, Interdiciplinary Procedures Center, Strategic Laboratory	Claudia Regina Gonçalves; Claudio Tavares Sacchi; Erica Valessa Ramos Gomes; Karoline Rodrigues Campos
EPI_ISL_471556	Pronto Socorro Jose Ibrahin	Instituto Adolfo Lutz, Interdiciplinary Procedures Center,	Claudia Regina Gonçalves; Claudio Tavares Sacchi; Erica Valessa Ramos Gomes

		Strategic Laboratory	
EPI_ISL_527867	Pronto Socorro Municipal - Balneario São José	Instituto Adolfo Lutz, Interdiciplinary Procedures Center, Strategic Laboratory	Claudia Regina Gonçalves; Claudio Tavares Sacchi; Erica Valessa Ramos Gomes
EPI_ISL_523961	Pronto Socorro Municipal 21 de Junho	Instituto Adolfo Lutz, Interdiciplinary Procedures Center, Strategic Laboratory	Claudia Regina Gonçalves; Claudio Tavares Sacchi; Erica Valessa Ramos Gomes
EPI_ISL_515529	Pronto Socorro Municipal Julio Tupy	Instituto Adolfo Lutz, Interdiciplinary Procedures Center, Strategic Laboratory	Claudia Regina Gonçalves; Claudio Tavares Sacchi; Erica Valessa Ramos Gomes
EPI_ISL_693202	Pronto Socorro Municipal Prof. Joao Catarin Mezomo	Instituto Adolfo Lutz, Interdiciplinary Procedures Center, Strategic Laboratory	Claudia Regina Gonçalves; Claudio Tavares Sacchi; Erica Valessa Ramos Gomes; Karoline Rodrigues Campos
EPI_ISL_515554, EPI_ISL_523958, EPI_ISL_523959	Pronto Socorro Municipal de Perus	Instituto Adolfo Lutz, Interdiciplinary Procedures Center, Strategic Laboratory	Claudia Regina Gonçalves; Claudio Tavares Sacchi; Erica Valessa Ramos Gomes
EPI_ISL_693231	Pronto Socorro Municipal de Santa Branca	Instituto Adolfo Lutz, Interdiciplinary Procedures Center, Strategic Laboratory	Claudia Regina Gonçalves; Claudio Tavares Sacchi; Erica Valessa Ramos Gomes; Karoline Rodrigues Campos
EPI_ISL_693210	Pronto-Socorro Dr. Osmar Mesquita	Instituto Adolfo Lutz, Interdiciplinary Procedures Center, Strategic Laboratory	Claudia Regina Gonçalves; Claudio Tavares Sacchi; Erica Valessa Ramos Gomes; Karoline Rodrigues Campos
EPI_ISL_482298, EPI_ISL_482322, EPI_ISL_482324, EPI_ISL_482333, EPI_ISL_482344, EPI_ISL_482372, EPI_ISL_482402, EPI_ISL_482413, EPI_ISL_482422, EPI_ISL_482453, EPI_ISL_482465			
see above	Providence St. Joseph Health Molecular Genomics Laboratory	Providence St. Joseph Health Molecular Genomics Laboratory	Alexa K Dowdell; Brian D Piening; Carlo B Bifulco; Fred L Robinson; Mary Campbell
EPI_ISL_416521	Public Health Laboratory	Public Health Laboratory, Saudi CDC	A; Albarrag
EPI_ISL_513312, EPI_ISL_513313	Public Health, United States Air Force School of Aerospace Medicine	Public Health, United States Air Force School of Aerospace Medicine	A.C.; A.K.; A.W.; B.C.; C.R.; Chapleau; Connors; E.A.; Fries; J.R.; Javorina; Lambert; Macias; Meyer; Purves; R.R. and Starr; S.M.
EPI_ISL_448358, EPI_ISL_448359, EPI_ISL_449046, EPI_ISL_449054, EPI_ISL_449087, EPI_ISL_453608, EPI_ISL_457370, EPI_ISL_457477, EPI_ISL_457523			
see above	Quadram Institute Bioscience	COVID-19 Genomics UK (COG-UK) Consortium	Alexander J Trotter; Alison E. Mather; Alp Aydin; Ana P. Tedim; Anastasia Kolyva; Andrew Bell; Andrew J. Page; Claire Stuart; Dave J. Baker; Gemma L. Kay; John Wain; Justin O'Grady; Leonardo de Oliveira Martins; Lizzie Meadows; Maria Diaz; Mark Webber; Muhammed Yasir; Nabil-Fareed Alikhan; Ngozi Elumogo; Nicholas M. Thomson; Rachael Stanley; Rachel Gilroy; Reenesh Prakash; Samir Dervisevic; Samuel Bloomfield; Steven Rudder; Thanh Le-Viet
EPI_ISL_425570	Queens Medical Centre, Clinical Microbiology Department / DeepSeq Nottingham	COVID-19 Genomics UK (COG-UK) Consortium	Christopher Moore; Fei Sang; Gemma Clark; Hannah Howson-Wells; Johnny Debebe; Jonathan Ball; Joseph Chappell; Manjinder Khakh; Matthew Carlisle; Matthew Loose; Nadine Holmes; Patrick McClure; Theocharis Tsoleridis; Victoria Wright; Wendy Smith
EPI_ISL_530277	Queensland Health Forensic and Scientific Services, Public Health Virology	Public Health Virology Laboratory, Forensic and Scientific Services, Queensland Health	Son Nguyen et al
EPI_ISL_467840, EPI_ISL_571021, EPI_ISL_571116, EPI_ISL_571267, EPI_ISL_571271, EPI_ISL_571383, EPI_ISL_571514, EPI_ISL_571519, EPI_ISL_571715, EPI_ISL_571872, EPI_ISL_572183, EPI_ISL_603862, EPI_ISL_603878, EPI_ISL_603988, EPI_ISL_604046, EPI_ISL_604048, EPI_ISL_604097, EPI_ISL_604099, EPI_ISL_604167, EPI_ISL_604208, EPI_ISL_604223, EPI_ISL_604368, EPI_ISL_604383, EPI_ISL_604403, EPI_ISL_604489, EPI_ISL_604498, EPI_ISL_604506, EPI_ISL_604564, EPI_ISL_604666, EPI_ISL_604674, EPI_ISL_604747, EPI_ISL_604805, EPI_ISL_604842, EPI_ISL_604918, EPI_ISL_604919, EPI_ISL_604949, EPI_ISL_604967			
see above	Quest Diagnostics	Quest Diagnostics	Anderson; Anderson, B.; B.P.; D.F.; Gerasimova, A.; Grover, D.; Hua, M.; K.E.; Kagan; Lacbawan, F.; Liu Y.; Livingston; Owen, R.; R.M.; R.M. and Owen, R.; Rosenthal; S.H.; Shalhout
EPI_ISL_455780	REGIONAL VRDL,ICMR-RMRC BBSR	Immunogenomics lab, Institute of Life Sciences, Bhubaneswar	Ajay Parida; Arup Ghosh; Atimukta Jha; COVID-19 team of ILS & RMRC; DBT's PAN-INDIA 1000 SARS-CoV2 RNA genome sequencing consortium; Debdutta Bhattacharya; Ghulam Hussain Syed; Jaya Singh Khastri; Jyotirmayee Turuk; Manasi Priyadarshini; Orissa COVID-19 study group; Punit Prasad; Rajeeb Swain; Rupesh Dash; Sanghamitra Pati; Shanti Senapati; Shuchi Smita; Soma Chattopadhyay; Sunil Raghav; Swati Madhulika; Tushar K. Beuria; Viplov K. Biswas
EPI_ISL_812203	RI State Health Laboratories	Pathogen Discovery, Respiratory Viruses Branch, Division of Viral Diseases, Centers for Disease Control and Prevention	Anna Montmayeur; Anna Uehara; Brian Lynch; Clinton R. Paden; Haibin Wang; Jing Zhang; Krista Queen; Peter Cook; Rachel Marine; Suxiang Tong; Yan Li; Ying Tao
EPI_ISL_766040	RS Tingkat II Moh. Ridwan Meuraksa	Eijkman Institute for Molecular Biology, Ministry of Research and Technology/National Agency for Research and Innovation	Amin Soebandrio; David H Muljono; Edison Johar; Frilasita A Yudhaputri; Hidayat Trimarsanto; Khin Saw Myint; Safarina G Malik
EPI_ISL_489202	Regional Virus Laboratory, Belfast Health and Social Care Trust	Wellcome Sanger Institute for the COVID-19 Genomics UK (COG-UK) consortium	Alison Watt; Ciara Cox; Conall McCaughey; Cordelia Langford; David K. Jackson; David Simpson; Derek Fairley; Dominic Kwiatkowski; Ewan Harrison; Ian Johnston; James McKenna; John Sillitoe on behalf of the Wellcome Sanger Institute COVID-19 Surveillance Team (http://www.sanger.ac.uk/covid-team); Mairead Connor; Roberto Amato; Sonia Goncalves; Susan Feeney; Tanya Curran; Zoltan Molnar; and Alex Alderton
EPI_ISL_661271	Research platform for Transfusion-transmitted Disease, Institute of Blood Transfusion, Chinese Academy of Medical Sciences	Research platform for Transfusion-transmitted Disease, Institute of Blood Transfusion, Chinese Academy of Medical Sciences	He; M. and Fan, Z.
EPI_ISL_420728, EPI_ISL_421810, EPI_ISL_421921, EPI_ISL_423242, EPI_ISL_423771, EPI_ISL_464217, EPI_ISL_465563, EPI_ISL_465602, EPI_ISL_465884, EPI_ISL_465935, EPI_ISL_466028, EPI_ISL_466533, EPI_ISL_466597, EPI_ISL_526539			
see above	Respiratory Virus Unit, Microbiology Services Colindale, Public Health England	Respiratory Virus Unit, Microbiology Services Colindale, Public Health England	Angie Lackenby; Joanna Ellis; Jonathan Hubb; Kirstin Edwards; Leena Bhaw; Maria Zambon; Monica Galiano; Omolola Akinbami; PHE Covid Sequencing Team; Richard Myers; Shahjahan Miah; Steven Platt; Tiina Talts
EPI_ISL_631920	Richmond University Medical Center	New York City Public Health Laboratory	Jade Wang; et al.
EPI_ISL_450178	Robert Garry lab	Andersen lab at Scripps Research	Allison Smither; Antoinette Bell; Arnaud Drouin; Dahlene Fusco; Gilberto Sabino-Santos; Kaylynn Genemaras; Lilia Melnik; Patricia Snarski; Robert Garry with SEARCH Alliance San Diego
EPI_ISL_514131, EPI_ISL_514132, EPI_ISL_514133, EPI_ISL_514134, EPI_ISL_514135, EPI_ISL_514136, EPI_ISL_514137, EPI_ISL_514138			
see above	Rondônia Central Public Health Laboratory (LACEN/RO), vinculated to State Health Secretariat of Rondônia (SESAU/RO)	Molecular Virology Laboratory of Oswaldo Cruz Foundation of Rondônia	Adriana Cristina Salvador Maia; Alcione de Oliveira dos Santos; Alice Paula Di Sabatino Guimarães; Aline Linhares Ferreira de Melo Mendonça; Caio Henrique Nemeth Santos; Camila Flávia Gomes Azzi; Celina Aparecida Bertoni Lugtenburg; Cicileia Correia da Silva; Felipe Gomes Naveca; Felipe Souza Nogueira-Lima; Fernando Rodrigues Máximo; Jansen Fernandes de Medeiros; Juan Miguel Vilallobos-Salcedo and Deusilene Souza Vieira; Juan Miguel Vilallobos-Salcedo and Deusilene Souza Vieira.; Juan Miguel Vilallobos-Salcedo and Deusilene Souza Vieira1; Juliana Loca Furtado; Luan Felipe Botelho-Souza; Suelen Cavalcante; Tárcio Peixoto Roca; Rita de Cássia Pontello Rampazzo
EPI_ISL_451091, EPI_ISL_451112, EPI_ISL_451117, EPI_ISL_468027, EPI_ISL_483071, EPI_ISL_483089, EPI_ISL_508138, EPI_ISL_508139			
see above	SA Pathology	SA Pathology	Chuan Kok Lim; Geoff Higgins; Ivan Bastian; Lex Leong; Mark Turra
EPI_ISL_648217	SILAB	Laboratorio Mixto de Biotecnología Acuática (LMBA)	Adriana Giri; Agustina Cerri; Ana Cavatorta; Ana Paletta; Diego Chouhy; Elisa Bolatti; Elizabeth Tapia; Federico Remes Lenicov; Flavio Spetale; Gastón Viarengo; Ignacio García Labari; Javier Murillo; Joaquín Ezpeleta; Julian Acosta; Laura Angelone; Leandro Ciappina; María Re; Pablo Casal; Pilar Bulacio; Silvana Spinelli; Silvia Arranz; Sofía Lavista Llanos; Vanina Villanova; Victoria Posner
EPI_ISL_547575	SVO Jundiaí	Instituto Adolfo Lutz, Interdiciplinary Procedures Center, Strategic Laboratory	Claudia Regina Gonçalves; Claudio Tavares Sacchi; Erica Valessa Ramos Gomes; Karoline Rodrigues Campos
EPI_ISL_845627	SYNLAB COLOMBIA S.A.S	Instituto Nacional de Salud - Dirección de Investigación en Salud Pública	Carlos Franco-Muñoz; Diego A. Álvarez-Díaz; Diego Andrés Prada; Gerardo Santamaría; Jonathan Reales; Julian Naizaque; Katherine Laiton-Donato; Magdalena Wiesner; Marcela Mercado-Reyes; Maria T. Herrera-Sepúlveda; Martha Lucia Ospina Martinez; Mauricio Pacheco-Montealegre; Paola Muñoz-Laiton; Sheryll Corchuelo
EPI_ISL_794666	SYNLAB REGIONAL NOROCCIDENTE	Instituto Nacional de Salud - Dirección de Investigación en Salud Pública	Carlos Franco-Muñoz; Diego A. Álvarez-Díaz; Diego Andrés Prada; Gerardo Santamaría; Jonathan Reales; Julian Naizaque; Katherine Laiton-Donato; Magdalena Wiesner; Marcela Mercado-Reyes; Maria T. Herrera; Martha Lucia Ospina Martinez; Mauricio Pacheco-Montealegre; Paola Muñoz-Laiton;

			Sheryl Corchuelo
EPI_ISL_416524	Saitama Medical University Hospital	Saitama Medical University	Kazuo Imai
EPI_ISL_467957, EPI_ISL_483161, EPI_ISL_494416, EPI_ISL_494442, EPI_ISL_494456, EPI_ISL_494467, EPI_ISL_494580, EPI_ISL_494587, EPI_ISL_635342, EPI_ISL_635618, EPI_ISL_636069, EPI_ISL_636194, EPI_ISL_636211, EPI_ISL_648922, EPI_ISL_649018, EPI_ISL_666994, EPI_ISL_667046, EPI_ISL_730088, EPI_ISL_730330			
see above	San Diego County Public Health Laboratory	Andersen lab at Scripps Research	Brett Austin; Jovan Shephard; SEARCH Alliance San Diego with Tracy Basler
EPI_ISL_748667, EPI_ISL_749148, EPI_ISL_749151, EPI_ISL_749155, EPI_ISL_749238, EPI_ISL_749474, EPI_ISL_749906, EPI_ISL_750108, EPI_ISL_750161, EPI_ISL_750162, EPI_ISL_750168, EPI_ISL_750169, EPI_ISL_750170, EPI_ISL_750171, EPI_ISL_750172, EPI_ISL_750173, EPI_ISL_750174, EPI_ISL_750176, EPI_ISL_750177, EPI_ISL_750178, EPI_ISL_750256, EPI_ISL_750430, EPI_ISL_750820, EPI_ISL_751011			
see above	Sanatorio Americano	Institut Pasteur de Montevideo	Ana Carolina Mendonça; Andres Lizasoain; Camila Simoes; Cecilia Alonso; Cecilia Salazar; Daiana Mir; Fernando Lopez-Tort; Fernando Motta; Gonzalo Bello; Ighor Arantes; Ignacio Ferrés; Jose Sotelo; Leticia Maya; Letícia Garay Martins; Luciana Appolinario; Lucia Spangenberg; Mailen Arleo; Mariana Brandes; Marilda Mendonça Siqueira; Marilda Tereza Mar da Rosa; Maria Jose Benitez-Galeano; Martín Graña; Matias Castells; Matias Victoria; Matías Salvo; Natalia Rego; Natalia Reyes; Pablo Smircich; Paola Cristina Resende; Rodney Colina; Tamara Fernandez-Calero; Tania Possi; Tatiana Schäffer Gregianini; Veronica Noya; Yasser Vega
EPI_ISL_583492	Santa Casa Anna Cintra	Instituto Adolfo Lutz, Interdiciplinary Procedures Center, Strategic Laboratory	Claudia Regina Gonçalves; Claudio Tavares Sacchi; Erica Valessa Ramos Gomes; Karoline Rodrigues Campos
EPI_ISL_547580, EPI_ISL_603026, EPI_ISL_603032, EPI_ISL_693219	Santa Casa da Misericórdia de Presidente Prudente	Instituto Adolfo Lutz, Interdiciplinary Procedures Center, Strategic Laboratory	Claudia Regina Gonçalves; Claudio Tavares Sacchi; Erica Valessa Ramos Gomes; Karoline Rodrigues Campos
EPI_ISL_735407	Santa Casa de Marilia	Instituto Adolfo Lutz, Interdiciplinary Procedures Center, Strategic Laboratory	Claudia Regina Gonçalves; Claudio Tavares Sacchi; Erica Valessa Ramos Gomes; Karoline Rodrigues Campos
EPI_ISL_603024, EPI_ISL_603027, EPI_ISL_603038	Santa Casa de Misericordia de Araçatuba	Instituto Adolfo Lutz, Interdiciplinary Procedures Center, Strategic Laboratory	Claudia Regina Gonçalves; Claudio Tavares Sacchi; Erica Valessa Ramos Gomes; Karoline Rodrigues Campos
EPI_ISL_693212	Santa Casa de Misericordia de Braganca Paulista	Instituto Adolfo Lutz, Interdiciplinary Procedures Center, Strategic Laboratory	Claudia Regina Gonçalves; Claudio Tavares Sacchi; Erica Valessa Ramos Gomes; Karoline Rodrigues Campos
EPI_ISL_524469	Santa Casa de Misericordia de Sao Paulo	Instituto Adolfo Lutz, Interdiciplinary Procedures Center, Strategic Laboratory	Claudia Regina Gonçalves; Claudio Tavares Sacchi; Erica Valessa Ramos Gomes
EPI_ISL_693198	Santa Casa de Misericordia de Sao Paulo - Hospital Central	Instituto Adolfo Lutz, Interdiciplinary Procedures Center, Strategic Laboratory	Claudia Regina Gonçalves; Claudio Tavares Sacchi; Erica Valessa Ramos Gomes; Karoline Rodrigues Campos
EPI_ISL_693211	Santa Casa de Misericordia e Maternidade	Instituto Adolfo Lutz, Interdiciplinary Procedures Center, Strategic Laboratory	Claudia Regina Gonçalves; Claudio Tavares Sacchi; Erica Valessa Ramos Gomes; Karoline Rodrigues Campos
EPI_ISL_547579	Santa Casa de Misericórdia de Araçatuba	Instituto Adolfo Lutz, Interdiciplinary Procedures Center, Strategic Laboratory	Claudia Regina Gonçalves; Claudio Tavares Sacchi; Erica Valessa Ramos Gomes; Karoline Rodrigues Campos
EPI_ISL_603031	Santa Casa de Presidente Epitácio	Instituto Adolfo Lutz, Interdiciplinary Procedures Center, Strategic Laboratory	Claudia Regina Gonçalves; Claudio Tavares Sacchi; Erica Valessa Ramos Gomes; Karoline Rodrigues Campos
EPI_ISL_524464	Santa Casa de Santa Isabel	Instituto Adolfo Lutz, Interdiciplinary Procedures Center, Strategic Laboratory	Claudia Regina Gonçalves; Claudio Tavares Sacchi; Erica Valessa Ramos Gomes
EPI_ISL_435663	Santa Clara County Public Health Department	Chiu Laboratory, University of California, San Francisco	Brandon Bonin; Debra A. Wadford; Elsa Villarino; Scot Federman; Wei Gu; Xianding Deng; and Charles Y. Chiu
EPI_ISL_759859	School of Public Health, The University of Hong Kong	School of Public Health, The University of Hong Kong	Barrs; Brackman; C.J.; Chu; D.K.W.; E.M.W.; K.W.S.; Law; P.Y.L.; Peiris, M.; Perera; R.A.P.M. and Sit; T.H.C.; Tam; To; V.R.; V.Y.T.; Yu
EPI_ISL_437567, EPI_ISL_458240, EPI_ISL_458275, EPI_ISL_458276, EPI_ISL_494611, EPI_ISL_494651, EPI_ISL_494674, EPI_ISL_494712			
see above	Scripps Medical Laboratory	Andersen lab at Scripps Research	Ellen Stefanski; Ian Mchardy; SEARCH Alliance San Diego with Michael Quigley
EPI_ISL_525703	Seattle Flu Study	Seattle Flu Study	Amanda Adler; Barry R. Lutz; Benjamin Pelle; Caitlin R. Wolf; Chris D. Frazar; Deborah A. Nickerson; Elisabeth Brandstetter; Helen Y. Chu; Janet A. Englund; Jay Shendure; Jeff Duchin; Jover Lee; Kairsten Fay; Karen Cowgill; Kirsten Lacombe; Lea M. Starita; Mark J. Rieder; Matthew Richardson; Matthew Thompson; Melissa Truong; Michael Boeckh; Michael Famulare; Misja Ilcisin; Peter D. Han; Stephanie Schrag; Thomas R. Sibley; Trevor Bedford
EPI_ISL_693238, EPI_ISL_693239, EPI_ISL_693247	Secao Centro de Diagnostico Secedi	Instituto Adolfo Lutz, Interdiciplinary Procedures Center, Strategic Laboratory	Claudia Regina Gonçalves; Claudio Tavares Sacchi; Erica Valessa Ramos Gomes; Karoline Rodrigues Campos
EPI_ISL_735405	Secretaria Minucipal de Saude de Birigui	Instituto Adolfo Lutz, Interdiciplinary Procedures Center, Strategic Laboratory	Claudia Regina Gonçalves; Claudio Tavares Sacchi; Erica Valessa Ramos Gomes; Karoline Rodrigues Campos
EPI_ISL_708530	Secretaria Municipal de Saude de Fernandópolis	Instituto Adolfo Lutz, Interdiciplinary Procedures Center, Strategic Laboratory	Carlos Henrique Camargo; Claudia Regina Gonçalves; Claudio Tavares Sacchi; Erica Valessa Ramos Gomes; Fernanda Modesto Tolentino Binhardi; Janaina Other Martins Montanha; Karoline Rodrigues Campos; Marcia Maria Costa Nunes Soares; Maricelia Navarro Pinheiro Flores
EPI_ISL_468320	Secretaria Municipal de Saude de Hortolandia	Instituto Adolfo Lutz, Interdiciplinary Procedures Center, Strategic Laboratory	Claudia Regina Gonçalves; Claudio Tavares Sacchi; Erica Valessa Ramos Gomes
EPI_ISL_574583	Secretaria Municipal de Saude de Jandira	Instituto Adolfo Lutz, Interdiciplinary Procedures Center, Strategic Laboratory	Claudia Regina Gonçalves; Claudio Tavares Sacchi; Erica Valessa Ramos Gomes; Karoline Rodrigues Campos
EPI_ISL_574597	Secretaria Municipal de Saude de Jarinu	Instituto Adolfo Lutz, Interdiciplinary Procedures Center, Strategic Laboratory	Claudia Regina Gonçalves; Claudio Tavares Sacchi; Erica Valessa Ramos Gomes; Karoline Rodrigues Campos
EPI_ISL_833164	Secretaria Municipal de Saude de Santa Barbara d'oeste	Instituto Adolfo Lutz, Interdiciplinary Procedures Center, Strategic Laboratory	Claudia Regina Gonçalves; Claudio Tavares Sacchi; Erica Valessa Ramos Gomes; Karoline Rodrigues Campos
EPI_ISL_547576, EPI_ISL_603035	Secretaria Municipal de Saúde	Instituto Adolfo Lutz, Interdiciplinary Procedures Center, Strategic Laboratory	Claudia Regina Gonçalves; Claudio Tavares Sacchi; Erica Valessa Ramos Gomes; Karoline Rodrigues Campos
EPI_ISL_693221, EPI_ISL_693222	Secretaria Municipal de Saúde de Birigui	Instituto Adolfo Lutz, Interdiciplinary Procedures Center, Strategic Laboratory	Claudia Regina Gonçalves; Claudio Tavares Sacchi; Erica Valessa Ramos Gomes; Karoline Rodrigues Campos
EPI_ISL_693215	Secretaria Municipal de Saúde de Iracemapolis	Instituto Adolfo Lutz, Interdiciplinary Procedures Center, Strategic Laboratory	Claudia Regina Gonçalves; Claudio Tavares Sacchi; Erica Valessa Ramos Gomes; Karoline Rodrigues Campos
EPI_ISL_693228	Secretaria Municipal de Sorocaba	Instituto Adolfo Lutz, Interdiciplinary Procedures Center, Strategic Laboratory	Claudia Regina Gonçalves; Claudio Tavares Sacchi; Erica Valessa Ramos Gomes; Karoline Rodrigues Campos
EPI_ISL_471542	Secretaria de Saude de Mogi das Cruzes	Instituto Adolfo Lutz, Interdiciplinary Procedures Center, Strategic Laboratory	Claudia Regina Gonçalves; Claudio Tavares Sacchi; Erica Valessa Ramos Gomes
EPI_ISL_417924	Secretaría de Salud Medellín	Instituto Nacional de Salud, Universidad Cooperativa de Colombia, Instituto Alexander von Humboldt, Imperial College-London, London School of Hygiene & Tropical Medicine	Astrid C. Flórez; Carlos Franco-Muñoz; Christian Julian VillabonaArenas; Diana Marcela Walteros-Acero; Diego A. Álvarez-Díaz; Erika Ospitia; Gloria Puerto; Jose A. Usme-Ciro; Juliana Barbosa; Katherine Laiton-Donato; Liz Villabona-Arenas; Luz Dary Rodriguez; Mailyn A. Gonzalez; Marcela Mercado-Reyes; Martha Lucia Ospina Martinez; Nicolás D. Franco-Sierra; Sergio Gomez Rangel; Sussy Echeverria-Londoño; Zulma M. Cucunubá
EPI_ISL_708529	Secretária Municipal de Saude de Fernandópolis	Instituto Adolfo Lutz, Interdiciplinary Procedures Center,	Carlos Henrique Camargo; Claudia Regina Gonçalves; Claudio Tavares Sacchi; Erica Valessa Ramos Gomes; Fernanda Modesto Tolentino Binhardi;

		Strategic Laboratory	Janaina Other Martins Montanha; Karoline Rodrigues Campos; Marcia Maria Costa Nunes Soares; Maricelia Navarro Pinheiro Flores
EPI_ISL_583502	Serv de Vig Sanitaria Epidemio e CTRL de Zoonoses Guaruja	Instituto Adolfo Lutz, Interdiciplinary Procedures Center, Strategic Laboratory	Claudia Regina Gonçalves; Claudio Tavares Sacchi; Erica Valessa Ramos Gomes; Karoline Rodrigues Campos
EPI_ISL_420598, EPI_ISL_420599, EPI_ISL_420600, EPI_ISL_778843	Servicio Virosis Respiratorias-Departamento Virología-INEI	Instituto Nacional Enfermedades Infecciosas C.G.Malbran	Avaro M.; Baumeister E.; Benedetti E.; Campos J.; Cisterna D.; Dattero ME; Lorenzo F.; Molina V.; Perandones C.; Poklepovich T.; Pontoriero A.; Russo M.; Tuduri E.
EPI_ISL_436311	Servicio de Microbiologia. Hospital Clinico Universitario de Valencia	Sequencing and Bioinformatics Service and Molecular Epidemiology Research Group. FISABIO-Public Health	Beatriz Beamud; David Navarro; Fernando Gonzalez-Candelas; Giuseppe D'Auria; Griselda De Marco; Inma Galán Vendrell; Ivan Ansari; Lidia Ruiz Roldan; Llúcia Martínez-Priego; Loreto Ferrús Abad; Maria Alma Bracho; Mariana Reyes-Prieto; Marta Pla Diaz; Neris Garcia-Gonzalez; Paula Ruiz-Hueso; Sandra Carbo; Vicente Soriano Chirona
EPI_ISL_452567	Servicio de Microbiología y Parasitología clínica. UCEIMP. Hospital Universitario Virgen del Rocío/IBIS/CSIC/US.	SeqCOVID-SPAIN consortium/IBV(CSIC)	Guillermo Martí-n Gutiérrez; Javier Aznar Martí-n and SeqCOVID-SPAIN consortium; Lidia Gálvez Benitez; Verónica González Galán; Ángel Rodrí-guez Villodres
EPI_ISL_510390	Servicio de Microbiología, Hospital Miguel Servet, Zaragoza	SeqCOVID-SPAIN consortium/IBV(CSIC)	Alexander Tristancho Baró; Ana Milagro; Antonio Rezusta López; Nieves Martínez Cameo and SeqCOVID-SPAIN consortium; Yolanda Gracia Grataloup
EPI_ISL_468991, EPI_ISL_819151	Servicio de Microbiología, Hospital Universitario Son Espases	SeqCOVID-SPAIN consortium/IBV(CSIC)	Antonio Oliver and SeqCOVID-SPAIN consortium; Carla López-Causapé; Jordi Reina
EPI_ISL_420112, EPI_ISL_420120, EPI_ISL_420121, EPI_ISL_425217, EPI_ISL_436211	Servicio de Microbiología. Consorcio Hospital General Universitario de Valencia	Sequencing and Bioinformatics Service and Molecular Epidemiology Research Group. FISABIO-Public Health	Beatriz Beamud; Concepcion Gimeno; David Navarro; Fernando Gonzalez-Candelas; Giuseppe D'Auria; Griselda De Marco; Inma Galán Vendrell; Ivan Ansari; Lidia Ruiz Roldan; Llúcia Martínez-Priego; Loreto Ferrús Abad; Maria Alma Bracho; Maria Dolores Ocete; Mariana Reyes-Prieto; Marta Pla Diaz; Neris Garcia-Gonzalez; Paula Ruiz-Hueso; Sandra Carbo; Vicente Soriano Chirona
EPI_ISL_796097	Servicio de Microbiología. Hospital General Universitario de Castellón	SeqCOVID-SPAIN consortium/IBV(CSIC)	María Dolores Tirado Balaguer and SeqCOVID-SPAIN consortium; Rosario Moreno Muñoz
EPI_ISL_509644, EPI_ISL_509649, EPI_ISL_509653, EPI_ISL_510507	Servicio de Microbiología. Hospital Universitario Donostia. OSI Donostialdea. Área de Enfermedades Infecciosas, Grupo de Infección Respiratoria y Resistencia Antimicrobiana. Instituto de Investigación Sanitaria Biodonostia	SeqCOVID-SPAIN consortium/IBV(CSIC)	Gustavo Cilla; Jose Maria Marimón and SeqCOVID-SPAIN consortium; Luis Piñeiro; Milagrosa Montes
EPI_ISL_574598	Servico de Verificacao de Obito SVO	Instituto Adolfo Lutz, Interdiciplinary Procedures Center, Strategic Laboratory	Claudia Regina Gonçalves; Claudio Tavares Sacchi; Erica Valessa Ramos Gomes; Karoline Rodrigues Campos
EPI_ISL_534315, EPI_ISL_583495	Serviço de Verificação de Óbitos SVO Guaruulhos	Instituto Adolfo Lutz, Interdiciplinary Procedures Center, Strategic Laboratory	Claudia Regina Gonçalves; Claudio Tavares Sacchi; Erica Valessa Ramos Gomes; Karoline Rodrigues Campos
EPI_ISL_515543	Serviço de Vigilância Sanitária e Epidemiológica	Instituto Adolfo Lutz, Interdiciplinary Procedures Center, Strategic Laboratory	Claudia Regina Gonçalves; Claudio Tavares Sacchi; Erica Valessa Ramos Gomes
EPI_ISL_636113, EPI_ISL_636114, EPI_ISL_636115, EPI_ISL_636116, EPI_ISL_636117, EPI_ISL_636118, EPI_ISL_636119, EPI_ISL_636120, EPI_ISL_730127, EPI_ISL_730128, EPI_ISL_730129, EPI_ISL_730130, EPI_ISL_730131, EPI_ISL_730132, EPI_ISL_730133, EPI_ISL_730134, EPI_ISL_730135, EPI_ISL_730136			
see above	Sharp HealthCare Laboratory	Andersen lab at Scripps Research	Art Mendoza; Cathy Woerle; Jacquelyn Berumen; Liam McGinnis; Omid Bakhtar; SEARCH Alliance San Diego with Aaron Harding
EPI_ISL_470589, EPI_ISL_470590, EPI_ISL_470591, EPI_ISL_470593, EPI_ISL_470594, EPI_ISL_470595, EPI_ISL_470596			
see above	Simile	Bioinformatics Laboratory / LNCC	Alexandra Gerber; Amilcar Tanuri; Ana Paula Guimarães; CADDE-group; Carolina Voloch; Ester Cerdeira Sabino; Filipe Romero; Ingra Morales Claro; Jaqueline Goes de Jesus; Laboratorio Hermes Pardini; Laboratorio Simile; Luiz Gonzaga Paula de Almeida; Mariane Talon; Nuno Rodrigues Faria; Renato Santana Aguiar e Ana Tereza Vasconcelos; Ronaldo da Silva Francisco Junior; Terezinha Marta Pereira; working group UFMG; working group UFRJ; Átila Duque Rossi
EPI_ISL_623104, EPI_ISL_623105	Simile Medicina Diagnóstica	Bioinformatics Laboratory / LNCC	Alexandra L Gerber; Amilcar Tanuri; Ana Paula de C Guimarães; Ana Tereza R de Vasconcelos; Carolina M Voloch; Covid19-UFRJ Workgroup; Cynthia C Cardoso; Diana Mariani; Luiz G P de Almeida; Luís Cristóvão Pôrto; Orlando C. Ferreira; Otavio J. Brustolini; Renato S Aguiar; Ronaldo S Francisco Jr; Terezinha M P P Castiñeiras
EPI_ISL_579181	Southern Community Labs Dunedin	Institute of Environmental Science and Research (ESR)	Anja Werno; Antje van der Linden; Arlo Upton; Chris Mansell; David Hammer; Dragana Drinkovic; Erasmus Smit; Gary McAuliffe; Hana Sofia Andersson; Hermes Perez; James Ussher; Jill Sherwood; Jing Wang; Joep de Ligt; Josh Freeman; Julia Howard; Juliet Elvy; Lauren Jelly; Mary DeAlmeida; Matt Blakiston; Matt Storey; Matthew Rogers; Max Bloomfield; Michael Addidle; Michelle Balm; Muhammad Faisal; Nikki Freed; Olin Silander; Sally Roberts; Sarah Jefferies; Sharmini Muttaiyah; Susan Morpeth; Susan Taylor; Timothy Blackmore; Vani Sathyendran; Veronica Playle; Virginia Hope; Xiaoyun Ren
EPI_ISL_514646	St. Luke's Hospital	Minnesota Department of Health, Public Health Laboratory	Jacob Garfin; Matt Plumb; and Xiong Wang
EPI_ISL_450474, EPI_ISL_476785	Stanford clinical virology lab	Chan-Zuckerberg Biohub	Benjamin Pinksy; Carlos Bustamante; Euan Ashley; Hannah N. DeJong; Jason Andrews; John Gorzynski; Katharine Walter; Manuel Rivas; Matthew T. Wheeler; Victoria N. Parikh; with CZB Cliahub Consortium
EPI_ISL_752624, EPI_ISL_752627, EPI_ISL_752636	State Laboratories Division, Hawaii State Department of Health	State Laboratories Division, Hawaii State Department of Health	Drew Kuwazaki; Edward Desmond; Pamela O'Brien; Razvan Sultana; Sabrina Diemert
EPI_ISL_428861, EPI_ISL_428875, EPI_ISL_428877, EPI_ISL_428913, EPI_ISL_428915	State Research Center of Virology and Biotechnology VECTOR, Department of Collection of Microorganisms	State Research Center of Virology and Biotechnology VECTOR, Department of Collection of Microorganisms	Alexander N. Shvalov; Anastasiya A. Nazarenko; Anastasiya M. Smirnova; Elena V. Gavrilova; Oleg V. Pyankov; Rinat A. Maksyutov; Sergey A. Bodnev; Tatyana V. Tregubchak
EPI_ISL_682261	TAMIZAJE COMUNITARIO- PASO CANOAS	Inciensa, Instituto Costarricense de Investigación y Enseñanza en Nutrición y Salud	Adriana Godinez & Melany Calderon; Claudio Soto-Garita; Estela Cordero; Francisco Duarte; Hebleen Porras
EPI_ISL_426532	TGen North	TGen North	Darrin Lemmer; Dave Engelthaler; Jolene Bowers; Megan Folkerts
EPI_ISL_427398, EPI_ISL_436099	TSGH-CP molecular lab	TSGH-CP molecular lab	Cherng-Lih Perng; Chien-Wen Chen; Chih-Kai Chang; Feng-Yee Chang; Hsing-Yi Chung; Hung-Sheng Shang; Jung-Chung Lin; Kuo-Ming Yeh; Kuo-Sheng Hung; Ming-Jr JIAN; Ming-Jr Jian; Sheng-Kang Chiu; Shih-Hung Tsai; Tien-Yao Chang
EPI_ISL_672123, EPI_ISL_672140	The Ashley Laboratory, Stanford University	Chan-Zuckerberg Biohub	CZB Cliahub Consortium
EPI_ISL_489708	The National Institute of Public Health	The National Institute of Public Health and State Veterinary Institute Prague	A; D; H; J; Jirincova; L; Nagy; Novakova; Trnka; Vecerova
EPI_ISL_417580, EPI_ISL_417602, EPI_ISL_417678, EPI_ISL_417745, EPI_ISL_417771, EPI_ISL_417799, EPI_ISL_417866, EPI_ISL_424380, EPI_ISL_424382, EPI_ISL_424448, EPI_ISL_424512, EPI_ISL_424558, EPI_ISL_424605			
see above	The National University Hospital of Iceland	deCODE genetics	Agnar Helgason; Alma Moller; Arna B Agustsdottir; Arnaldur Gylfason; Asgeir Sigurdsson; Aslaug Jonasdottir; Berglind Eiriksdottir; Bjarni Thorbjornsson; Brynjar O Jensson; Daniel F Gudbjartsson; Droplaug N Magnusdottir; Elisabet E Gardarsdottir; Emil A Thorarensen; Gardar Sveinbjornsson; Gisli Masson; Gudmundur Georgsson; Gudmundur L Norddahl; Gudrun Sigmundsdottir; Hakon Jonsson; Hilma Holm; Ingileif Jonsdottir; Jona Saemundsdottir; Kamilla S Josefsdottir; Kari Stefansson; Karl G Kristinsson; Kjartan R Gudmundsson; Kristin E Sveinsdottir; Louise le Roux; Maney Sveinsdottir; Olafia S Gretarsdottir; Olafur T Magnusson; Pall Melsted; Patrick Sulem; Run Fridriksdottir; Thora R Gunnarsdottir; Thordur Kristjansson; Thorolfur Gudnason; Unnur Thorsteinsdottir
EPI_ISL_479891	Tokyo Metropolitan Institute of Public Health	Pathogen Genomics Center, National Institute of Infectious Diseases	Hajime Kamiya; Kenji Sadamasu; Kentaro Itokawa; Makoto Kuroda; Mami Nagashima; Masanori Hashino; Motoi Suzuki; Rina Tanaka; Takashi Chiba; Tsuyoshi Sekizuka
EPI_ISL_586398, EPI_ISL_792091	Toronto Invasive Bacterial Diseases Network	McMaster University	Ahmed Draia; Allison McGeer; Andrew G. McArthur; Angel Li; Emily Panousis; Hooman Derakhshani; Jalees Nasir; Kuganya Nirmalarajah; Michael Surette; Patryk Aftanas; Samira Mubareka
EPI_ISL_717692, EPI_ISL_717694, EPI_ISL_717695, EPI_ISL_717696, EPI_ISL_717697, EPI_ISL_717698, EPI_ISL_717699, EPI_ISL_717700, EPI_ISL_756362, EPI_ISL_756363			
see above	Trinidad Public Health Laboratory	Carrington Lab, Department of PreClinical Sciences, Faculty of Medical Sciences, The University of the West Indies	Adesh Ramsubhag; Arianne Brown-Jordan; Avery Hinds; Chinna Chinnadurai; Christine V. F. Carrington; Christopher Oura; Gabriel Escobar; Jaya Jayaraman; Jerome Foster; Karla Georges; Marsha Ivey; Naresh Nandram; Nikita S. D. Sahadeo; Nuno Faria; Oliver Pybus; Rahul Naidu; Rajini

			Haraksingh; Risha Singh; Roshan Parasram; Sarah Hill; Stanley Giddings; SueMin Nathaniel; Vernie Ramkissoon
EPI_ISL_735419	UBS Alvarenga	Instituto Adolfo Lutz, Interdiciplinary Procedures Center, Strategic Laboratory	Claudia Regina Gonçalves; Claudio Tavares Sacchi; Erica Valessa Ramos Gomes; Karoline Rodrigues Campos
EPI_ISL_837053	UBS Darcy Alves e Robalinho	Instituto Adolfo Lutz, Interdiciplinary Procedures Center, Strategic Laboratory	Claudia Regina Gonçalves; Claudio Tavares Sacchi; Erica Valessa Ramos Gomes; Karoline Rodrigues Campos
EPI_ISL_735422	UBS Dematchi	Instituto Adolfo Lutz, Interdiciplinary Procedures Center, Strategic Laboratory	Claudia Regina Gonçalves; Claudio Tavares Sacchi; Erica Valessa Ramos Gomes; Karoline Rodrigues Campos
EPI_ISL_837054	UBS Jose Sabino Ferreira	Instituto Adolfo Lutz, Interdiciplinary Procedures Center, Strategic Laboratory	Claudia Regina Gonçalves; Claudio Tavares Sacchi; Erica Valessa Ramos Gomes; Karoline Rodrigues Campos
EPI_ISL_735420	UBS Riacho Grande	Instituto Adolfo Lutz, Interdiciplinary Procedures Center, Strategic Laboratory	Claudia Regina Gonçalves; Claudio Tavares Sacchi; Erica Valessa Ramos Gomes; Karoline Rodrigues Campos
EPI_ISL_735421	UBS Sta Terezinha	Instituto Adolfo Lutz, Interdiciplinary Procedures Center, Strategic Laboratory	Claudia Regina Gonçalves; Claudio Tavares Sacchi; Erica Valessa Ramos Gomes; Karoline Rodrigues Campos
EPI_ISL_693227	UBS Vila Marchi	Instituto Adolfo Lutz, Interdiciplinary Procedures Center, Strategic Laboratory	Claudia Regina Gonçalves; Claudio Tavares Sacchi; Erica Valessa Ramos Gomes; Karoline Rodrigues Campos
EPI_ISL_523963	UBS Vila Silvia	Instituto Adolfo Lutz, Interdiciplinary Procedures Center, Strategic Laboratory	Claudia Regina Gonçalves; Claudio Tavares Sacchi; Erica Valessa Ramos Gomes
EPI_ISL_471648	UBS e Pronto Socorro Jd. Jacira	Instituto Adolfo Lutz, Interdiciplinary Procedures Center, Strategic Laboratory	Claudia Regina Gonçalves; Claudio Tavares Sacchi; Erica Valessa Ramos Gomes
EPI_ISL_483215, EPI_ISL_483222, EPI_ISL_483237, EPI_ISL_483281, EPI_ISL_483282, EPI_ISL_483290, EPI_ISL_483344, EPI_ISL_483381, EPI_ISL_483467, EPI_ISL_483517			
see above	UC San Diego Center for Advanced Laboratory Medicine	Andersen lab at Scripps Research	Ji H Shin; SEARCH Alliance San Diego with David Pride
EPI_ISL_428996, EPI_ISL_428998, EPI_ISL_428999, EPI_ISL_445182	UCSF Clinical Microbiology Laboratory	Chan-Zuckerberg Biohub	CZB Cliahub Consortium
EPI_ISL_671979, EPI_ISL_671981, EPI_ISL_671982, EPI_ISL_671983, EPI_ISL_671984, EPI_ISL_671985, EPI_ISL_671988, EPI_ISL_671989, EPI_ISL_671990, EPI_ISL_671991, EPI_ISL_671992, EPI_ISL_671994, EPI_ISL_671995, EPI_ISL_671996, EPI_ISL_671997, EPI_ISL_671998, EPI_ISL_671999, EPI_ISL_672000, EPI_ISL_672001, EPI_ISL_672002, EPI_ISL_672003, EPI_ISL_672004, EPI_ISL_672005, EPI_ISL_672007, EPI_ISL_672008, EPI_ISL_672009, EPI_ISL_672010, EPI_ISL_672011			
see above	UEES BioLab	Omics Sciences Laboratory	Darlyn Amaya; Derly Andrade Molina; Edith Lopez Montanero; Fernando Espinoza Fuentes; Gabriel Morey León; Juan Carlos Fernández Cadena; Katheryn Sacheri Viteri; Pedro Barberán; Rubén Armas González
EPI_ISL_794656	UNIDAD HEMATOLOGICA ESPECIALIZADA	Instituto Nacional de Salud - Dirección de Investigación en Salud Pública	Carlos Franco-Muñoz; Diego A. Álvarez-Díaz; Diego Andrés Prada; Gerardo Santamaría; Jonathan Reales; Julian Naizaque; Katherine Laiton-Donato; Magdalena Wiesner; Marcela Mercado-Reyes; Maria T. Herrera; Martha Lucia Ospina Martinez; Mauricio Pacheco-Montealegre; Paola Muñoz-Laiton; Sheryl Corchuelo
EPI_ISL_445317, EPI_ISL_445322	UNIV.DE CHILE HOSP.CLINICO	Instituto de Salud Publica de Chile	Alejandra Acevedo; Andrés E Castillo; Bárbara Parra; Carolina Tambley; Gabriel Leal; Jaime Lagos; Jorge Fernandez; Loredana Arata; Patricia Bustos; Paz Tapia; Rodrigo Fasce; Winston Andrade
EPI_ISL_445247	UNIVERSIDAD DE LOS ANDES	Instituto de Salud Publica de Chile	Alejandra Acevedo; Andrés E Castillo; Bárbara Parra; Carolina Tambley; Gabriel Leal; Jaime Lagos; Jorge Fernandez; Loredana Arata; Patricia Bustos; Paz Tapia; Rodrigo Fasce; Winston Andrade
EPI_ISL_515522	UPA 24HS de Itatiba	Instituto Adolfo Lutz, Interdiciplinary Procedures Center, Strategic Laboratory	Claudia Regina Gonçalves; Claudio Tavares Sacchi; Erica Valessa Ramos Gomes
EPI_ISL_523983, EPI_ISL_523993	UPA Campo Limpo	Instituto Adolfo Lutz, Interdiciplinary Procedures Center, Strategic Laboratory	Claudia Regina Gonçalves; Claudio Tavares Sacchi; Erica Valessa Ramos Gomes
EPI_ISL_603025	UPA Central de Caraguatatuba	Instituto Adolfo Lutz, Interdiciplinary Procedures Center, Strategic Laboratory	Claudia Regina Gonçalves; Claudio Tavares Sacchi; Erica Valessa Ramos Gomes; Karoline Rodrigues Campos
EPI_ISL_534311	UPA III 26 de Agosto	Instituto Adolfo Lutz, Interdiciplinary Procedures Center, Strategic Laboratory	Claudia Regina Gonçalves; Claudio Tavares Sacchi; Erica Valessa Ramos Gomes
EPI_ISL_583496	UPA Jandira	Instituto Adolfo Lutz, Interdiciplinary Procedures Center, Strategic Laboratory	Claudia Regina Gonçalves; Claudio Tavares Sacchi; Erica Valessa Ramos Gomes; Karoline Rodrigues Campos
EPI_ISL_693237, EPI_ISL_693245	UPA Santa Isabel	Instituto Adolfo Lutz, Interdiciplinary Procedures Center, Strategic Laboratory	Claudia Regina Gonçalves; Claudio Tavares Sacchi; Erica Valessa Ramos Gomes; Karoline Rodrigues Campos
EPI_ISL_523975, EPI_ISL_523980	UPA Tito Lopes	Instituto Adolfo Lutz, Interdiciplinary Procedures Center, Strategic Laboratory	Claudia Regina Gonçalves; Claudio Tavares Sacchi; Erica Valessa Ramos Gomes
EPI_ISL_468316	UPA Vila Assis	Instituto Adolfo Lutz, Interdiciplinary Procedures Center, Strategic Laboratory	Claudia Regina Gonçalves; Claudio Tavares Sacchi; Erica Valessa Ramos Gomes
EPI_ISL_515550	UPA Vila Santa Catarina	Instituto Adolfo Lutz, Interdiciplinary Procedures Center, Strategic Laboratory	Claudia Regina Gonçalves; Claudio Tavares Sacchi; Erica Valessa Ramos Gomes
EPI_ISL_436466, EPI_ISL_436488, EPI_ISL_436500, EPI_ISL_436503	UPMC Clinical Laboratory	Microbial Genome Sequencing Center, Microbial Genomic Epidemiological Laboratory	Chinelo Ezeonwuku; Dan Snyder; Jane W. Marsh; Kady D Waggle; Lee H. Harrison; Marissa P Griffith; Mustapha M Mustapha; Stephanie L Mitchell; Vatsala R Srinivasa; Vaughn S. Cooper
EPI_ISL_454375, EPI_ISL_454406	UPMC Clinical Microbiology Laboratory	Microbial Genome Sequencing Center, Microbial Genomic Epidemiological Laboratory	Chinelo Ezeonwuku; Dan Snyder; Jane W. Marsh; Kady D. Waggle; Lee H. Harrison; Marissa P. Griffith; Mustapha M. Mustapha; Stephanie L. Mitchell; Vatsala R. Srinivasa; Vaughn S. Cooper
EPI_ISL_681954, EPI_ISL_681972, EPI_ISL_681982, EPI_ISL_682000	UPMC Clinical Microbiology Laboratory	Microbial Genomic Epidemiology Laboratory, University of Pittsburgh	Chinelo Ezeonwuku; Dan Snyder; Jane W. Marsh; Kady D. Waggle; Lee H. Harrison; Marissa P. Griffith; Mustapha M. Mustapha; Stephanie L. Mitchell; Vatsala R. Srinivasa; Vaughn S. Cooper
EPI_ISL_430919, EPI_ISL_461420, EPI_ISL_477683, EPI_ISL_477693, EPI_ISL_491029, EPI_ISL_570185	UW Virology Lab	UW Virology Lab	Alexander Greninger; Amin Addetia; Hong Xie; Keith Jerome; Keith R Jerome; Lasata Shrestha; Meei-Li Huang; Pavitra Roychoudhury; Truong Nguyen; Victoria M Rachleff
EPI_ISL_734509, EPI_ISL_734593, EPI_ISL_734614, EPI_ISL_734727, EPI_ISL_734827, EPI_ISL_738220	UZ Leuven, National Reference Laboratory for Coronaviruses, Laboratory Medicine, Leuven, Belgium	KU Leuven, Rega Institute, Clinical and Epidemiological Virology	Bert Vanmechelen; Joan Marti-Carerras; Piet Maes; Tony Wawina-Bokalanga
EPI_ISL_693225	Ubs Vila Rosa - Olímpia Gomes De Almeida	Instituto Adolfo Lutz, Interdiciplinary Procedures Center, Strategic Laboratory	Claudia Regina Gonçalves; Claudio Tavares Sacchi; Erica Valessa Ramos Gomes; Karoline Rodrigues Campos
EPI_ISL_792303, EPI_ISL_792304, EPI_ISL_792305	Unidad de Virología, Centro de Educación Médica en Investigaciones Clínicas CEMIC	Área de Secuenciación del Laboratorio de Virología del Hospital de Niños Dr. Ricardo Gutierrez on behalf of 'Proyecto Argentino Interinstitucional de genomica de SARS-CoV-2' (PAIS Consortium)	AS; Echavarría; Goya; LE; Lusso; M; MI; MS; Mistchenko; Nabaes Jodar; Natale; S; Valinotto; Viegas, M.
EPI_ISL_735397	Unidade Respiratória Nova Hortolandia	Instituto Adolfo Lutz, Interdiciplinary Procedures Center,	Claudia Regina Gonçalves; Claudio Tavares Sacchi; Erica Valessa Ramos Gomes; Karoline Rodrigues Campos

		Strategic Laboratory	
EPI_ISL_574590	Unidade de Printo Atendimento UPA I Santa Isabel	Instituto Adolfo Lutz, Interdiciplinary Procedures Center, Strategic Laboratory	Claudia Regina Gonçalves; Claudio Tavares Sacchi; Erica Valessa Ramos Gomes; Karoline Rodrigues Campos
EPI_ISL_735409	Unidade de Pronto Atendimento Carlos Lourenco	Instituto Adolfo Lutz, Interdiciplinary Procedures Center, Strategic Laboratory	Claudia Regina Gonçalves; Claudio Tavares Sacchi; Erica Valessa Ramos Gomes; Karoline Rodrigues Campos
EPI_ISL_693214	Unidade de Pronto Atendimento Central de Caraguatatuba	Instituto Adolfo Lutz, Interdiciplinary Procedures Center, Strategic Laboratory	Claudia Regina Gonçalves; Claudio Tavares Sacchi; Erica Valessa Ramos Gomes; Karoline Rodrigues Campos
EPI_ISL_693226	Unidade de Pronto Atendimento Sao José	Instituto Adolfo Lutz, Interdiciplinary Procedures Center, Strategic Laboratory	Claudia Regina Gonçalves; Claudio Tavares Sacchi; Erica Valessa Ramos Gomes; Karoline Rodrigues Campos
EPI_ISL_735406	Unidade de Pronto Atendimento UPA I Sta Isabel	Instituto Adolfo Lutz, Interdiciplinary Procedures Center, Strategic Laboratory	Claudia Regina Gonçalves; Claudio Tavares Sacchi; Erica Valessa Ramos Gomes; Karoline Rodrigues Campos
EPI_ISL_735414, EPI_ISL_735415, EPI_ISL_735417	Unidade de Pronto Atendimento de Agenor de Campos	Instituto Adolfo Lutz, Interdiciplinary Procedures Center, Strategic Laboratory	Claudia Regina Gonçalves; Claudio Tavares Sacchi; Erica Valessa Ramos Gomes; Karoline Rodrigues Campos
EPI_ISL_534325	Unidade de Vigilancia em Saude de Guarulhos	Instituto Adolfo Lutz, Interdiciplinary Procedures Center, Strategic Laboratory	Claudia Regina Gonçalves; Claudio Tavares Sacchi; Erica Valessa Ramos Gomes
EPI_ISL_693216, EPI_ISL_693217	Unidade de Vigilância Epiudemiológica de Araras	Instituto Adolfo Lutz, Interdiciplinary Procedures Center, Strategic Laboratory	Claudia Regina Gonçalves; Claudio Tavares Sacchi; Erica Valessa Ramos Gomes; Karoline Rodrigues Campos
EPI_ISL_420143	Unilabs Laboratory Medicine	Norwegian Institute of Public Health, Department of Virology	Hilde Elshaug; Kamilla Heddeland Instefjord; Karoline Bragstad; Kathrine Stene-Johansen; Olav Hungnes
EPI_ISL_812324	United States Air Force School of Aerospace Medicine	United States Air Force School of Aerospace Medicine	Amanda Javorina; Anthony Fries; Clarise Starr; Elizabeth Macias; Jennifer Meyer; Sarah Purves; William Gruner
EPI_ISL_525467, EPI_ISL_525468, EPI_ISL_525469	Universidad Iberoamericana	International Centre for Genetic Engineering and Biotechnology (ICGEB) and ARGO Open Lab Platform	Alejandro Vallejo Degaudenzi; Danilo Licastro; Eileen Riego; Leandro Tapia; Patricia Leon; Robert Paulino-Ramirez; Simeone Dal Monego; Sreejith Rajasekharan and Alessandro Marcello.; Victor Virgilio Calderon
EPI_ISL_523811, EPI_ISL_523812	Universidad Iberoamericana, Instituto de Medicina Tropical & Salud Global	International Centre for Genetic Engineering and Biotechnology (ICGEB) and ARGO Open Lab Platform	Alejandro Vallejo Degaudenzi; Danilo Licastro; Eileen Riego; Leandro Tapia; Robert Paulino-Ramirez; Simeone Dal Monego; Sreejith Rajasekharan and Alessandro Marcello.; Victor Virgilio Calderon
EPI_ISL_539783, EPI_ISL_539784, EPI_ISL_697796	Universidad Regional Amazonica IKIAM	Institute of Microbiology, Universidad San Francisco de Quito	Andrea Carrera; Belén Prado-Vivar; Bernardo Gutiérrez; Carolina Proaño-Bolaños; Fabian Aguilar; Gabriel Trueba; Giovanna Moran; Juan José Guadalupe; Katherine Apunte; Marcelo Ortiz; Michelle Grunauer; Monica Becerra-Wong; Nina Espinoza de los Monteros; Patricio Rojas-Silva; Paúl Cárdenas; Sonia Sislema; Sully Márquez; Verónica Barragán; Yeimy Rojas
EPI_ISL_445219	Universidad del Valle, Laboratorio de Microbiologia, VIREM	Universidad del Valle, Universidad Nacional de Colombia-Sede Palmira, International Center for Tropical Agriculture	Beatriz Parra; Diana López-Alvarez; Wilmer J. Cuellar
EPI_ISL_507070, EPI_ISL_665145	University College London Hospital	COVID-19 Genomics UK (COG-UK) Consortium	Catherine Houlihan; Dan Frampton; Judith Heaney; Matthew Byott; Moira Spyer and Eleni Nastouli; Stuart Kirk
EPI_ISL_440983, EPI_ISL_441001, EPI_ISL_441016, EPI_ISL_444099, EPI_ISL_457290, EPI_ISL_478419	University College London, Great Ormond Street Hospital for Children NHS Foundation Trust, Imperial College Healthcare NHS Trust	COVID-19 Genomics UK (COG-UK) Consortium	Alison Holmes; Charlotte Williams; Helena Tutill; Jacqueline Findlay; James Price; Judith Breuer; Julianne Brown; Kathryn Harris; Leysa Forrest; Mark Kristiansen; Paola Niola; Paola Resende Silva; Patricia Dyal; Paul Randell; Rachel Williams; Sam Weeks; Samuel Weeks; Sergi Castellano; Sunando Roy; Tony Brooks; Yasmin Panchbhaya
EPI_ISL_528003, EPI_ISL_528004, EPI_ISL_528067, EPI_ISL_528109, EPI_ISL_528132, EPI_ISL_528142, EPI_ISL_528229, EPI_ISL_528254, EPI_ISL_528258, EPI_ISL_528296, EPI_ISL_528299, EPI_ISL_528317, EPI_ISL_528330, EPI_ISL_528341, EPI_ISL_528365, EPI_ISL_528369, EPI_ISL_581715, EPI_ISL_581731, EPI_ISL_581806			
see above	University Hospital Basel, Clinical Virology	University Hospital Basel, Clinical Bacteriology	Adrian Egli; Alexander Gensch; Alfredo Mari; Christian Nickel; Hans Hirsch; Hans Pargger; Helena MB Seth-Smith; Julia Bielicki; Karoline Leuzinger; Kirstine K. Soegaard; Madlen Stange; Manuel Battegay; Martin Siegemund; Michael Osthoff; Michael Schweitzer; Myrta Brunner; Rita Schneider-Sliwa; Roland Bingisser; Sarah Tschudin-Sutter; Simon Fuchs; Stefano Bassetti; Tim Roloff
EPI_ISL_429206	University Hospitals of Geneva Laboratory of Virology	University Hospitals of Geneva Laboratory of Virology	Laubscher F.
EPI_ISL_775968, EPI_ISL_776197, EPI_ISL_776202, EPI_ISL_776228, EPI_ISL_776241, EPI_ISL_776262, EPI_ISL_776399, EPI_ISL_776548			
see above	University Medical Center Hamburg Eppendorf	Heinrich Pette Institute, Leibniz Institute for Experimental Virology	Adam Grundhoff; Alexis Robitaille; Johannes Knobloch; Martin Aepfelbacher; Nicole Fischer; Thomas Günther
EPI_ISL_479762, EPI_ISL_479771	University of Miami Immunology and Histocompatibility Laboratory	University of Miami Immunology and Histocompatibility Laboratory	Emilio Margolles-Clark; MD; PhD; PhD and Phillip Ruiz
EPI_ISL_578128, EPI_ISL_578140	University of Michigan Clinical Microbiology Laboratory	Lauring Lab, University of Michigan, Department of Microbiology and Immunology	Valesano
EPI_ISL_421321, EPI_ISL_428338, EPI_ISL_434613, EPI_ISL_450706, EPI_ISL_450715, EPI_ISL_484891, EPI_ISL_509818			
see above	University of Wisconsin-Madison AIDS Vaccine Research Laboratories	University of Wisconsin-Madison AIDS Vaccine Research Laboratories	Gage Moreno; Katarina Braun; et al. AIDS Vaccine Research Laboratories
EPI_ISL_693234	Upa Vereador Jose Da Rocha Goncalves	Instituto Adolfo Lutz, Interdiciplinary Procedures Center, Strategic Laboratory	Claudia Regina Gonçalves; Claudio Tavares Sacchi; Erica Valessa Ramos Gomes; Karoline Rodrigues Campos
EPI_ISL_434643, EPI_ISL_445239, EPI_ISL_475526	Uppsala Narakut Aleris	The Public Health Agency of Sweden	Anna Risberg; Anna-Malin Linde; Annika Nilsson; Karin Tegmark-Wisell; Maria Lind Karlberg; Mattias Haukland; Mia Brytting; Olov Svartstrom; Oskar Karlsson Lindsjo; Reza Advani; Sandra Broddesson; Theresa Enkirch
EPI_ISL_449834, EPI_ISL_524134	Utah Public Health Laboratory	Utah Public Health Laboratory	David R. Hillyard; E. Susan Slechta; Erin L. Young; Erin Young; Jeffrey B. Stevenson; Kelly Oakeson; Melanie A. Mallory; Michael T. Pyne; Salika M. Shakir; Tara Gallagher
EPI_ISL_454652	VI-US Virgin Islands Department of Health	Pathogen Discovery, Respiratory Viruses Branch, Division of Viral Diseases, Centers for Disease Control and Prevention	Anna Uehara; Bettina Bankamp; Clinton R. Paden; Haibin Wang; Jing Zhang; Krista Queen; Suxiang Tong; Yan Li; Ying Tao; Zachary Weiner
EPI_ISL_475520	Vardcentralen Brinken	The Public Health Agency of Sweden	Anna Risberg; Anna-Malin Linde; Karin Tegmark-Wisell; Maria Lind Karlberg; Mattias Haukland; Mia Brytting; Olov Svartstrom; Oskar Karlsson Lindsjo; Reza Advani; Sandra Broddesson
EPI_ISL_468478	Ventura County Public Health Lab	Chan-Zuckerberg Biohub	CZB Cliahub Consortium
EPI_ISL_678487	Veterinary Specialized Institute "Sabac", Serbia	Veterinary Specialized Institute "Kraljevo", Serbia	Afonso, C.; Banovic Djeri, B.; Jankovic, M.; Jovanovic, T.; Knezevic, A.; Mrkovacki, S.; Petrovic, T.; Sekler, M.; Tesovic, B.; Vidanovic, D.; Volkening, J.
EPI_ISL_426644, EPI_ISL_426646, EPI_ISL_426647, EPI_ISL_426855, EPI_ISL_426952, EPI_ISL_427103	Victorian Infectious Diseases Reference Laboratory (VIDRL)	Microbiological Diagnostic Unit Public Health Laboratory and Victorian Infectious Diseases Reference Laboratory, Doherty Institute	Caly L.; Druce J.; Sait, M.; Schultz M.; Seemann T.; Sherry, N.
EPI_ISL_419796, EPI_ISL_419798, EPI_ISL_419814, EPI_ISL_419908, EPI_ISL_419936	Victorian Infectious Diseases Reference Laboratory (VIDRL)	Victorian Infectious Diseases Reference Laboratory and Microbiological Diagnostic Unit Public Health Laboratory, Doherty Institute	Caly L.; Druce J.; Sait, M.; Schultz M.; Seemann T.; Sherry, N.
EPI_ISL_468313, EPI_ISL_468319, EPI_ISL_603033	Vigilancia Epidemiologica de São Bernardo do Campo	Instituto Adolfo Lutz, Interdiciplinary Procedures Center, Strategic Laboratory	Claudia Regina Gonçalves; Claudio Tavares Sacchi; Erica Valessa Ramos Gomes; Karoline Rodrigues Campos
EPI_ISL_515542	Vigilância Epidemiológica de Leme	Instituto Adolfo Lutz, Interdiciplinary Procedures Center,	Claudia Regina Gonçalves; Claudio Tavares Sacchi; Erica Valessa Ramos Gomes

		Strategic Laboratory	
EPI_ISL_603023	Vigilância em Saúde Visa Sul	Instituto Adolfo Lutz, Interdiciplinary Procedures Center, Strategic Laboratory	Claudia Regina Gonçalves; Claudio Tavares Sacchi; Erica Valessa Ramos Gomes; Karoline Rodrigues Campos
EPI_ISL_547573, EPI_ISL_583493	Vigilância em Saúde de Cajamar	Instituto Adolfo Lutz, Interdiciplinary Procedures Center, Strategic Laboratory	Claudia Regina Gonçalves; Claudio Tavares Sacchi; Erica Valessa Ramos Gomes; Karoline Rodrigues Campos
EPI_ISL_467002, EPI_ISL_574901, EPI_ISL_574904, EPI_ISL_574951, EPI_ISL_574969, EPI_ISL_574971, EPI_ISL_574989			
see above	Viollier AG	Department of Biosystems Science and Engineering, ETH Zürich	Christian Beisel; Christiane Beckmann; Christoph Noppen; Elodie Burcklen; Ina Nissen; Ivan Topolsky; Maurice Redondo; Natascha Santacroce; Niko Beerenwinkel; Noemie Santamaria de Souza; Olivier Kobel; Pedro Ferreira; Philipp Jablonski; Sarah Nadeau; Sophie Seidel; Susana Posada-Céspedes; Tanja Stadler; Tobias Schär
EPI_ISL_426463, EPI_ISL_426469, EPI_ISL_437413, EPI_ISL_437426, EPI_ISL_437791, EPI_ISL_452802, EPI_ISL_452851, EPI_ISL_467782, EPI_ISL_467928, EPI_ISL_495321, EPI_ISL_516679, EPI_ISL_516692, EPI_ISL_526841, EPI_ISL_572237			
see above	Virginia DCLS	Virginia DCLS	Virginia DCLS
EPI_ISL_435431	Virological Research Group, Szentágothai Research Centre	Bioinformatics Research Group, Szentágothai Research Centre	Attila Gyenesei; Endre Gábor Tóth; Ferenc Jakab; Gábor Kemenesi; Péter Urbán; Róbert Herczeg
EPI_ISL_487902, EPI_ISL_487961, EPI_ISL_488905	Virology Department, Royal Infirmary of Edinburgh, NHS Lothian / School of Biological Sciences, University of Edinburgh	Wellcome Sanger Institute for the COVID-19 Genomics UK (COG-UK) consortium	Colquhoun R; Cordelia Langford; David K. Jackson; Dewar R; Dominic Kwiatkowski; Ewan Harrison; Hill V; Ian Johnston; Jackson B; John Sillitoe on behalf of the Wellcome Sanger Institute COVID-19 Surveillance Team (http://www.sanger.ac.uk/covid-team); McCrone JT; McHugh M; O'Toole Á; Rambaut A; Roberto Amato; Rooke S; Scher E; Sonia Goncalves; Templeton K and Alex Alderton; Yu X
EPI_ISL_425959, EPI_ISL_433196, EPI_ISL_433378, EPI_ISL_433396, EPI_ISL_439308, EPI_ISL_439317, EPI_ISL_439365, EPI_ISL_449323, EPI_ISL_449329, EPI_ISL_461716			
see above	Virology Department, Royal Infirmary of Edinburgh, NHS Lothian / School of Biological Sciences, University of Edinburgh / Institute of Genetics and Molecular Medicine, University of Edinburgh	COVID-19 Genomics UK (COG-UK) Consortium	Balcaza C; Colquhoun R; Dewar R; Gallagher M; Hill V; Jackson B; McCrone JT; McHugh M; O'Toole A; O'Toole Á; O'ÄôToole Å; Rambaut A; Rooke S; Scher E; Templeton K; Williams TC; Yu X
EPI_ISL_441916, EPI_ISL_484469	Virology Department, Sheffield Teaching Hospitals NHS Foundation Trust/Department of Infection, Immunity and Cardiovascular Disease, The Medical School, University of Sheffield	COVID-19 Genomics UK (COG-UK) Consortium	Adri Angyal; Alex Keeley; Benjamin Lindsey; Cariad Evans; Danielle Groves; Dave Partridge; Katie Johnson; Laura Carrilero; Luke Green; Matthew Parker; Matthew Wyles; Mehmet Yavuz; Mohammad Raza; Nikki Smith; Paul Parsons; Rachel Tucker; Rebecca Brown; Thushan de Silva
EPI_ISL_445085	Virology Unit, Agrobiodiversity and Biotechnology Project, CIAT - International Center for Tropical Agriculture	Virology Unit, Agrobiodiversity and Biotechnology Project, CIAT - International Center for Tropical Agriculture	B. and Cuellar; Lopez, D.; Parra; W.J.
EPI_ISL_426440, EPI_ISL_426442, EPI_ISL_426449	WA State Department of Health	Pathogen Discovery, Respiratory Viruses Branch, Division of Viral Diseases, Centers for Disease Control and Prevention	Anna Uehara; Brian Hiatt; Clinton R. Paden; Denny Russell; Haibin Wang; Jesica Jacobs; Jessica Gant; Jing Zhang; Krista Queen; Suxiang Tong; Yan Li; Ying Tao
EPI_ISL_420080, EPI_ISL_427314, EPI_ISL_427320, EPI_ISL_430071, EPI_ISL_430074, EPI_ISL_430084, EPI_ISL_507269			
see above	WHO National Influenza Centre Russian Federation	WHO National Influenza Centre Russian Federation	Andrey Komissarov; Anna Ivanova; Artem Fadeev; Daria Danilenko; Mariia Sergeeva
EPI_ISL_525597, EPI_ISL_525627, EPI_ISL_525632, EPI_ISL_534731	Wadsworth Center, New York State Department of Health	Wadsworth Center, New York State Department of Health	Daryl M. Lamson; Erica Lasek-Nesselquist; Jonathan Plitnick; Kirsten St. George; Matthew D. Shudt; Navjot Singh; Sara Griesemer
EPI_ISL_676734, EPI_ISL_676749, EPI_ISL_676785, EPI_ISL_676836, EPI_ISL_676899	Wadsworth Center, New York State Department.of Health	Wadsworth Center, New York State Department.of Health	Alexis Russel; Daryl M. Lamson; Erasmus Schneider; Erica Lasek-Nesselquist; John Kelly; Jonathan Plitnick; Kirsten St. George; Navjot Singh; Sara Griesemer
EPI_ISL_420988, EPI_ISL_422027, EPI_ISL_422112, EPI_ISL_432178, EPI_ISL_432195, EPI_ISL_432223, EPI_ISL_446270			
see above	Wales Specialist Virology Centre	Public Health Wales Microbiology Cardiff	Alec Birchley; Alexander Adams; Amy Gaskin; Bree Gatica-Wilcox; Catherine Moore; Jason Coombes; Joanne Watkins; Johnathan Evans; Laura Gifford; Lauren Gilbert; Lee Graham; Malorie Perry; Matt Bull; Matthew Bull; Nicole Pacchiarini; Sally Corden; Sara Kumziene-Summerhayes; Sara Rey; Sarah Taylor; Simon Cottrell; Simon Cottrell Sara Rey; Sophie Jones; Tom Connor
EPI_ISL_472942, EPI_ISL_473283, EPI_ISL_474343, EPI_ISL_474356	Wales Specialist Virology Centre Sequencing lab: Pathogen Genomics Unit	COVID-19 Genomics UK (COG-UK) Consortium	Alec Birchley; Alexander Adams; Amy Gaskin; Angela Marchbank; Bree Gatica-Wilcox; Catherine Moore; Jason Coombes; Joanne Watkins; Joel Southgate; Johnathan Evans; Laura Gifford; Lauren Gilbert; Lee Graham; Malorie Perry; Matthew Bull; Nicole Pacchiarini; Sally Corden; Sara Kumziene-Summerhayes; Sara Rey; Sarah Taylor; Simon Cottrell; Sophie Jones; Tom Connor
EPI_ISL_430213, EPI_ISL_434081, EPI_ISL_434089, EPI_ISL_434118, EPI_ISL_434128, EPI_ISL_434129, EPI_ISL_434173, EPI_ISL_434189, EPI_ISL_434213, EPI_ISL_434245, EPI_ISL_434280, EPI_ISL_434332, EPI_ISL_434345, EPI_ISL_449839, EPI_ISL_449875, EPI_ISL_449933, EPI_ISL_449937, EPI_ISL_495653, EPI_ISL_495680, EPI_ISL_497090, EPI_ISL_497472			
see above	Washington State Department of Health	Seattle Flu Study	Amanda Adler; Barry R. Lutz; Benjamin Pelle; Brian Hiatt; Caitlin R. Wolf; Chris D. Frazar; Chu et al; Deborah A. Nickerson; Elisabeth Brandstetter; Geoff; Geoff Melly; Helen Y. Chu; Janet A. Englund; Jay Shendure; Jover Lee; Kairsten Fay; Kirsten Lacombe; Lea M. Starita; Mark J. Rieder; Matthew Richardson; Matthew Thompson; Melissa Truong; Michael Boeckh; Michael Famulare; Misja Ilcisin; Peter D. Han; Philip Dykema; Romesh Gautom; Scott Lindquist; Thomas R. Sibley; Trevor Bedford
EPI_ISL_425669, EPI_ISL_425717, EPI_ISL_425729, EPI_ISL_433554, EPI_ISL_433568, EPI_ISL_438829, EPI_ISL_473513, EPI_ISL_477957			
see above	West of Scotland Specialist Virology Centre, NHSGGC / MRC-University of Glasgow Centre for Virus Research	COVID-19 Genomics UK (COG-UK) Consortium	Alasdair MacLean; Alice Broos; Ana da Silva Filipe; Antonia Ho; Daniel Mair; David L Robertson; Elihu Aranday-Cortes; Emma Thomson; James Shepherd; Jenna Nichols; Joseph Hughes; Kathy Li; Kathy Smollett; Kirstyn Brunker; Kyriaki Nomikou; Lily Tong; Marc Niebel; Natasha Jesudason; Natasha Johnson; Patawee Asamaphan; Rajiv Shah; Richard Orton; Rory Gunson; Rory Gunson.; Sarah McDonald; Sreenu Vattipally; Stephen Carmichael; Yasmin Parr
EPI_ISL_454967, EPI_ISL_454977	Wuhan Chain Medical Labs (CMLabs)	State Key Laboratory of Biotherapy of Sichuan University	Baowen Du; Binwu Ying; Chao Tang; Chuan Chen; Hancheng Wei; Jia Geng; Jing-wen Lin; Lu Chen; Mingxia Yu; Minjin Wang; Weimin Li; Yongzhao Zhou
EPI_ISL_402124	Wuhan Jinyintan Hospital	Wuhan Institute of Virology, Chinese Academy of Sciences	Ding-Yu Zhang; Hao-Rui Si; Lei Zhang; Peng Zhou; Xing-Lou Yang; Yan Zhu; Zhengli Shi
EPI_ISL_462917	Wyoming Public Health Laboratory	Center for Global Health, University of New Mexico Health Sciences Center	Cari Sloma; Darrell Dinwiddie; Daryl Domman; Kurt Schwalm; Noah Hull; Rob Christensen; Wanda Manley
EPI_ISL_660069, EPI_ISL_660070, EPI_ISL_681702, EPI_ISL_681703	Zurita & Zurita Laboratorios	Zurita & Zurita Laboratorios	Camilo Zurita-Salinas; Gabriela Sevillano; Gabriela Sevillano Camilo Zurita-Salinas Karen Loaiza David Ortega-Paredes Jeannete Zurita; Jeannete Zurita
EPI_ISL_417543, EPI_ISL_417549	deCODE genetics	deCODE genetics	Agnar Helgason; Alma Moller; Arna B Agustsdottir; Arnaldur Gylfason; Asgeir Sigurdsson; Aslaug Jonasdottir; Berglind Eiriksdottir; Bjarni Thorbjornsson; Brynjar O Jensson; Daniel F Gudbjartsson; Droplaug N Magnusdottir; Elisabet E Gardarsdottir; Emil A Thorarensen; Gardar Sveinbjornsson; Gisli Masson; Gudmundur Georgsson; Gudmundur L Norddahl; Gudrun Sigmundsdottir; Hakon Jonsson; Hilma Holm; Ingileif Jonsdottir; Jona Saemundsdottir; Kamilla S Josefsdottir; Kari Stefansson; Karl G Kristinsson; Kjartan R Gudmundsson; Kristin E Sveinsdottir; Louise le Roux; Maney Sveinsdottir; Olafia S Gretarsdottir; Olafur T Magnusson; Pall Melsted; Patrick Sulem; Run Fridriksdottir; Thora R Gunnarsdottir; Thordur Kristjansson; Thorolfur Gudnason; Unnur Thorsteinsdottir
EPI_ISL_486427, EPI_ISL_486429	unknown	Clinical Laboratory, Hospital Israelita Albert Einstein	Amgarteb, D.; Amgarten, D.; C.L. and Pinho; F.G.; Guedes; J.R.; Malta, F.; Mangueira; R.A.; R.L.; Santana; de Menezes
EPI_ISL_468063	unknown	Computer Science and Engineering	A.B.; Adcock; Alejandro, B.; Arnold; Carrico, R.; Chariker; Chung, D.; E.C.; F.W.; Hwang; J.H.; J.W.; J.Y.; K.E.; L.A.; Lasnik; Palmer; Park; R.S.; Ramirez, J.; Rouchka; Waigel, S.; Wolf; Zacharias, W.; Zhang, M.
EPI_ISL_447652	unknown	Department of Medicine	Bampali, M.; Dovrolis, N.; Froukala, E.; Gatzidou, E.; Kassela, K.; N. and Karakasiliotis, I.; Spanakis; Stavropoulou, A.; Tsakris, A.; Veletza, S.
EPI_ISL_507018, EPI_ISL_507026	unknown	Infectious Diseases Research, King Abdullah International Medical Research Center (KAIMRC)	Alghoribi; M.F.

EPI_ISL_454036, EPI_ISL_454133, EPI_ISL_454202, EPI_ISL_454215, EPI_ISL_454245, EPI_ISL_454315, EPI_ISL_454333			
see above	unknown	Instituto Nacional de Saude (INSA)	Borges et al
EPI_ISL_593607, EPI_ISL_593608, EPI_ISL_593612, EPI_ISL_593633, EPI_ISL_593634, EPI_ISL_639741, EPI_ISL_639775, EPI_ISL_639783, EPI_ISL_639787, EPI_ISL_639792, EPI_ISL_639804, EPI_ISL_639807, EPI_ISL_693270			
see above	unknown	Public Health Virology Laboratory, Forensic and Scientific Services (PHV-FSS)	Son Nguyen et al.
EPI_ISL_458138, EPI_ISL_458139, EPI_ISL_458140, EPI_ISL_458141, EPI_ISL_458142, EPI_ISL_458143, EPI_ISL_458144, EPI_ISL_458145, EPI_ISL_458146, EPI_ISL_458147, EPI_ISL_458148, EPI_ISL_458149, EPI_ISL_524783, EPI_ISL_524784, EPI_ISL_524785, EPI_ISL_524786, EPI_ISL_524787, EPI_ISL_524788, EPI_ISL_524789, EPI_ISL_524790, EPI_ISL_524791, EPI_ISL_524792, EPI_ISL_524793, EPI_ISL_524794, EPI_ISL_524795, EPI_ISL_524796, EPI_ISL_524797, EPI_ISL_524798, EPI_ISL_524799, EPI_ISL_524800, EPI_ISL_524801			
see above	Evandro Chagas Institute	Evandro Chagas Institute	A.M.; Barbagelata; E.C.; E.M.A.; Ferreira; G.M.R; H.R; J.A.; Junior; L.C.; L.S.; M.C.; Martins; P.S.; Resque; Santos; Silva; Sousa; Sousa Junior; Viana; W.D.C.; da Silva
